# Photoinduced [4 + 2]-cycloaddition reactions of vinyldiazo compounds for the construction of heterocyclic and bicyclic rings†

**DOI:** 10.1039/d4sc03558e

**Published:** 2024-06-28

**Authors:** Ming Bao, Arnold R. Romero Bohórquez, Hadi Arman, Michael P. Doyle

**Affiliations:** a Department of Chemistry, The University of Texas at San Antonio San Antonio Texas 78249 USA michael.doyle@utsa.edu; b Grupo de Investigación en Compuestos Orgánicos de Interés Medicinal (CODEIM), Parque Tecnológico Guatiguará, Universidad Industrial de Santander A. A. 678 Piedecuesta Colombia

## Abstract

Highly selective formal [4 + 2]-cycloaddition of vinyldiazoacetates with azoalkenes from α-halohydrazones, as well as with cyclopentadiene and furan, occurs with light irradiation at room temperature, producing highly functionalized heterocyclic and bicyclic compounds in good yields and excellent diastereoseletivity. Under blue light these vinyldiazoacetate reagents selectively form unstable cyclopropenes that undergo intermolecular cycloaddition reactions at a faster rate than their competitive ene dimerization. [4 + 2]-cycloaddition of vinyldiazoacetates with *in situ* formed azoalkenes produces bicyclo[4.1.0]tetrahydropyridazine derivatives and, together with their cycloaddition using cyclopentadiene and furan that form tricyclic compounds, they occur with high chemoselectivity and diastereocontrol, good functional group tolerance, and excellent scalability. Subsequent transformations portray the synthetic versatility of these structures.

## Abstract 2

Under blue light vinyldiazoacetate reagents selectively form unstable cyclopropenes that undergo intermolecular cycloaddition reactions at a faster rate than their competitive ene dimerization.

## Graphical abstract

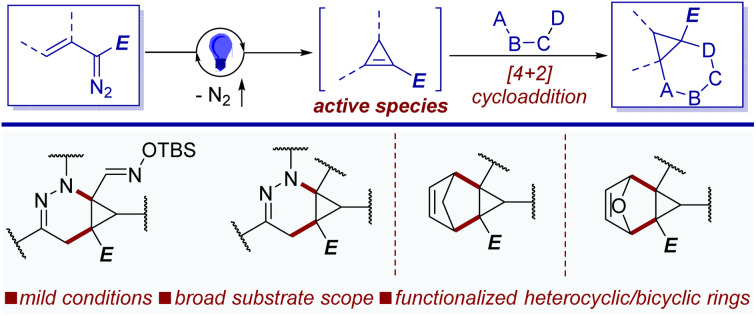

## Introduction

**Scheme 1:**
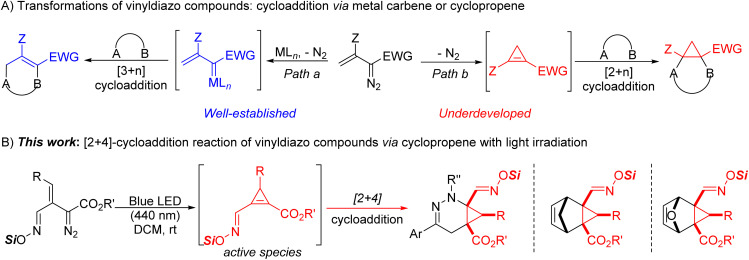
Catalytic transformations of vinyldiazo compounds.

Cycloaddition reactions have become powerful transformations for the construction of carbocyclic and heterocyclic compounds with structural diversity.^1^ Among these, major contributions have come from transition metal catalytic reactions of vinyldiazo compounds which generated metallovinylcarbenes that serve as 1,3-dipoles in various types of [3 + *n*]-cycloadditions or annulations (Scheme 1A, path a).^2^ In the course of these investigations, stable donor–acceptor cyclopropenes were found to be generated from enoldiazocarbonyl compounds under catalytic and thermal conditions, and these cyclopropenes serve as metal carbene precursors^3^ or could undergo cycloaddition reactions (Scheme 1A, path b).^4^ However, cyclopropenes bearing a hydrogen atom at the C-3 position often undergo alder-ene dimerization to give 3-cyclopropylcyclopropenes.^5^ Because cyclopropenes that can undergo the ene reaction are often unstable,^6^ the scope of their cycloaddition applications have been limited to cyclopropenes that can be stabilized.^4^ Limited attempts have been made for *in situ* generation of these unstable intermediates *via* elimination reactions of cyclopropyl halides,^7^ but they are highly restricted in their scope and applications. Thus, the development of a more general methodology for their *in situ* generation and applications for cycloaddition reactions remains a challenge.

We have recently shown that “unstable” cyclopropenes from a broad spectrum of vinyldiazo compounds can be generated photochemically in the presence of 1,3-dipoles to produce [3 + 2]-cycloaddition products in generally high yields and diastereocontrol.^8^ The propensity of stable cyclopropenes to undergo [4 + 2]-cycloaddition reactions is well known,^9^ but these transformations have been limited to cyclopropenes that do not undergo the ene reaction. This manuscript reports how previously inaccessible cyclopropene intermediates from diverse vinyldiazoacetates can be conveniently generated photolytically to undergo [4 + 2]-cycloaddition reactions with α-halohydrazones, cyclopentadiene, and furan for the synthesis of heterocyclic and bicyclic compounds (Scheme 1B). Furthermore, the cycloaddition products formed from oximidovinyldiazoacetates undergo convenient copper(ii) acetate catalyzed oxidative conversion to nitriles that, in some cases, is accompanied with highly structural isomerization under mild thermal conditions.

## Results and discussion

Initially, α-halohydrazone 2a, which is the precursor of azoalkenes in base-catalyzed reactions,^10^ was selected as the model substrate to investigate the viability of its reaction with vinyldiazoacetate 1a.^11^ Tetrahydropyridazines, as an important class of six-membered heterocycles, are widely found in many natural products and pharmaceutically active compounds;^12^ but there has only been one report of the synthesis of bicyclo[4.1.0]tetrahydropyridazines, which occurred with crotonate-derived sulfur ylides and offered only monosubstitution on the cyclopropane ring.^13^ Generally, the intermolecular [4 + 2] reaction between 1,2-diaza-1,3-dienes and alkenes is one of the most effective and economic approaches for the synthesis of tetrahydropyridazines.^14^ Thus, we anticipated that mild base would convert 2a to its azoalkene species, which could then undergo cycloaddition to the active cyclopropene generated *in situ* from oximidovinyldiazo acetates 1a with light irradiation. A variety of common solvents were employed using Cs_2_CO_3_ as the base with blue LED light sources (40 W) (Table 1). Interestingly, the desired [4 + 2]-cycloaddition product 3a was obtained in 67% yield with DCM as the solvent (Table 1, entry 1), while the use of THF and toluene mainly gave the Alder-ene dimer 4a of the cyclopropene (Table 1, entries 2 and 3). Fortunately, reducing the addition rate of vinyldiazo compound 1a lessened the competing ene reaction and provided better results with an isolated yield for 3a of 83% (Table 1, entry 4). Attempts to optimize the yield of 3a by varying the base and using a different wave length of blue light were unsuccessful (Table 1, entries 5–7).

**Table 1:** Optimization of the reaction conditions^a^

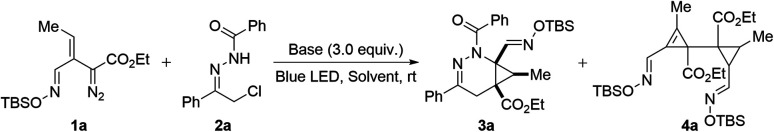				
Entry	Solvent	Base	*hv* [nm]	3a/4a yields^b^ [%]
1	DCM	Cs_2_CO_3_	440	67/25
2	THF	Cs_2_CO_3_	440	<5/86
3	Toluene	Cs_2_CO_3_	440	15/74
4^c^	DCM	Cs_2_CO_3_	440	83/10
5^c^	DCM	K_2_CO_3_	440	79/12
6^c^	DCM	Cs_2_CO_3_	467	40/50
7^c^	DCM	Cs_2_CO_3_	400	70/22

a Unless otherwise noted, the reaction was carried out on a 0.1 mmol scale: 2a (23.6 mg, 0.15 mmol, 1.5 equiv.), base (0.3 mmol, 3.0 equiv.) in 1.0 mL solvent and stirred over 2 h at room temperature, then 1a (31.1 mg, 0.1 mmol) in 1.0 mL solvent was added to the solution *via* a syringe pump over 2.0 h with irradiation by 440 nm blue LED.

b Isolated yields of 3a and 4a.

c 1a in 1.0 mL solvent was added to the solution *via* a syringe pump over 3.0 h.

**Scheme 2:**
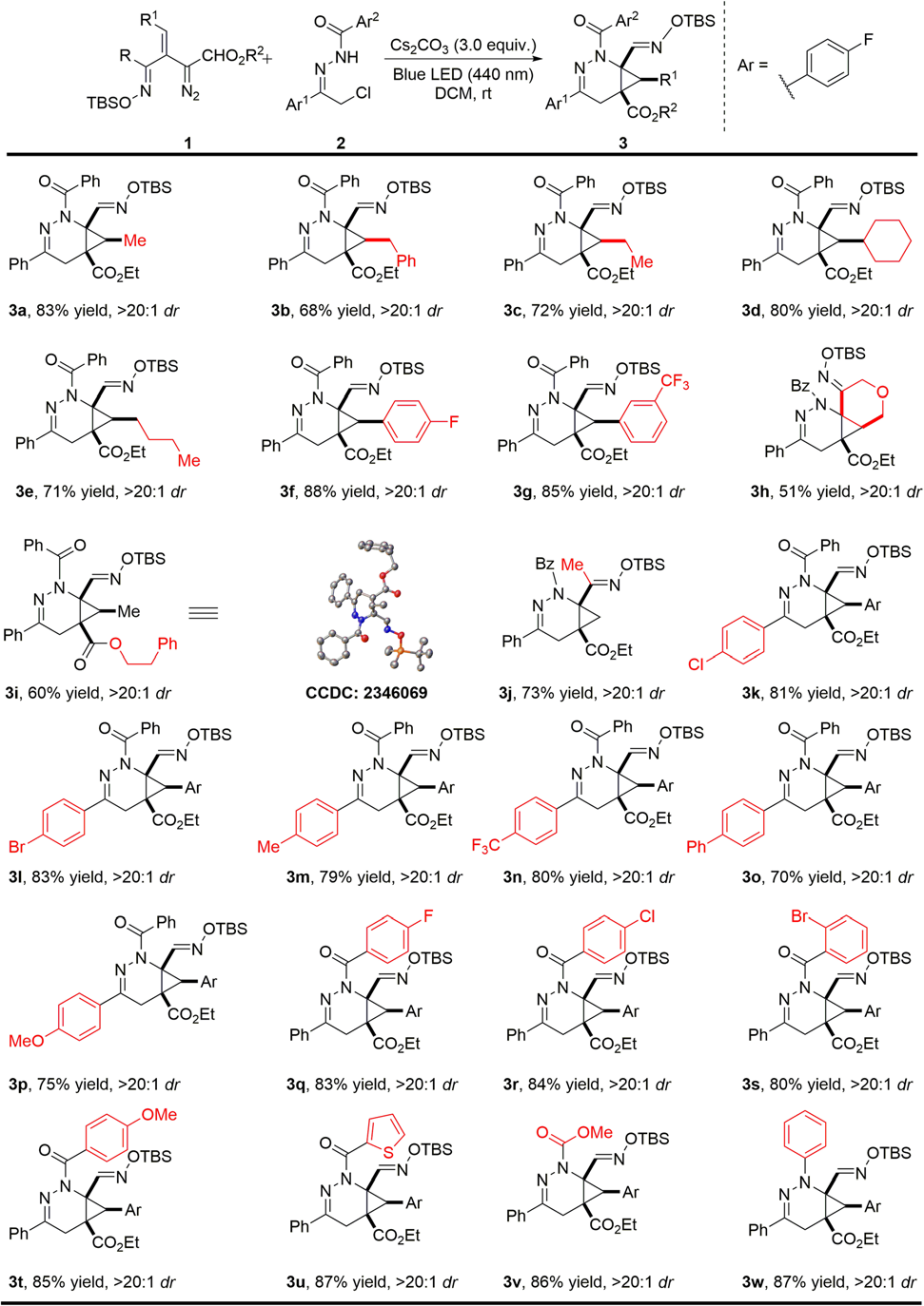
Photoinduced [4 + 2]-cycloaddition of diverse oximidovinyldiazo esters with α-halohydrazones. Reaction conditions: 2 (0.15 mmol, 1.5 equiv.), Cs_2_CO_3_ (0.3 mmol, 3.0 equiv.) in 1.0 mL DCM and stirred over 2 h at room temperature, then 1 (0.1 mmol) in 1.0 mL DCM was added to the solution *via* a syringe pump over 3.0 h with irradiation by 440 nm blue LED. Isolated yields are given.

With the optimal reaction conditions established, the substrate scope with respect to the oximidovinyldiazoacetate was first investigated (Scheme 2). Various alkyl substitutions at the β-position of oximidovinyldiazo compounds 1 were all well tolerated, delivering the bicyclo[4.1.0]tetrahydropyridazine products 3a–3e in 68–83% yields with high diastereoselectivity (>20 : 1 dr). Moreover, these reactions proceeded smoothly with fluoro and trifluoromethyl substituents on the aryl ring of oximidovinyldiazo compounds (3f–3g). The reaction could also be applied to a cyclic vinyldiazoacetate derivative and a phenethyl ester analogue with excellent dr values, although some deterioration in product yield was observed (3h–3i, 51–60% yields). Notably, the methyloximidovinyldiazo compound 1j also performed well under optimal conditions, furnishing the corresponding product 3j in 73% yield with >20 : 1 dr. The impact of aryl group substitution in α-halohydrazones 2 was then evaluated. The electronic effect of the substituents at the *para*-position on the Ar^1^ had little influence on the reaction outcomes, generating 3k–3p in 70–83% yields with >20 : 1 dr. In addition, the effect of the substituents on the Ar^2^ group was also investigated. For Ar^2^ with electron-withdrawing or electron-donating groups at the *para*-position and with the 1-thienyl substituent (3q–3u) product yields were at least 80% with >20 : 1 dr. It is worth mentioning that the *N*-carboxylate and *N*-phenyl derived α-halohydrazones also underwent the reaction smoothly, leading to the products 3v–3w in high yields and diastereocontrol. The structure of product 3i was confirmed by single-crystal X-ray diffraction analysis, and other products were assigned by analogy.

**Scheme 3:**
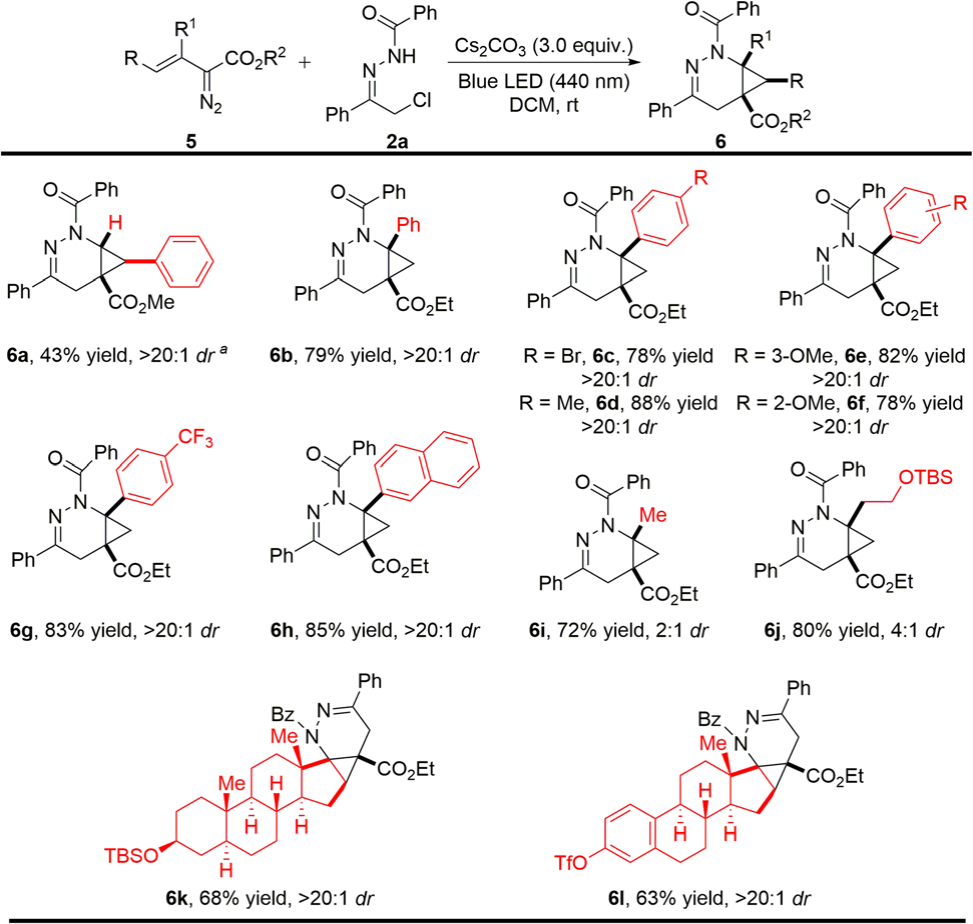
Photoinduced [4 + 2]-cycloaddition of 3-substituted vinyldiazo compounds with α-halohydrazones. Reaction conditions: 2a (23.6 mg, 0.15 mmol, 1.5 equiv.), Cs_2_CO_3_ (0.3 mmol, 3.0 equiv.) in 1.0 mL DCM and stirred over 2 h at room temperature, then 5 (0.1 mmol) in 1.0 mL DCM was added to the solution *via* a syringe pump over 3.0 h with irradiation by 440 nm blue LED. ^a^ Methyl 3-phenyl-1*H*-pyrazole-5-carboxylate was obtained in 40% yield. Isolated yields are given.

Encouraged by the above promising results, we further investigated the reaction scope by employing other types of vinyldiazo compounds beyond oximidovinyldiazoacetates (Scheme 3). In the case of methyl styryldiazoacetate, the cycloaddition product 6a was obtained under the established conditions with a high dr value in moderate yield due to competitive intramolecular pyrazole formation (40% yield).^15^ By contrast, cycloaddition occurred smoothly with β-arylvinyldiazo compounds having different substituents on the aryl ring, producing 6b–6g in high yields with >20 : 1 dr without competing pyrazole formation. In addition, β-alkylvinyldiazo compounds gave the corresponding heterocyclic products 6i and 6j in good yields, but with low diastereocontrol (6i and 6j in 2 : 1 and 4 : 1 dr, respectively). Surprisingly, vinyldiazo compounds (5k and 5l) derived from natural products epiandrosterone and estrone were also suitable, generating cycloaddition products 6k and 6l smoothly and selectively as signal enantiomers in synthetically useful yields.

**Scheme 4:**
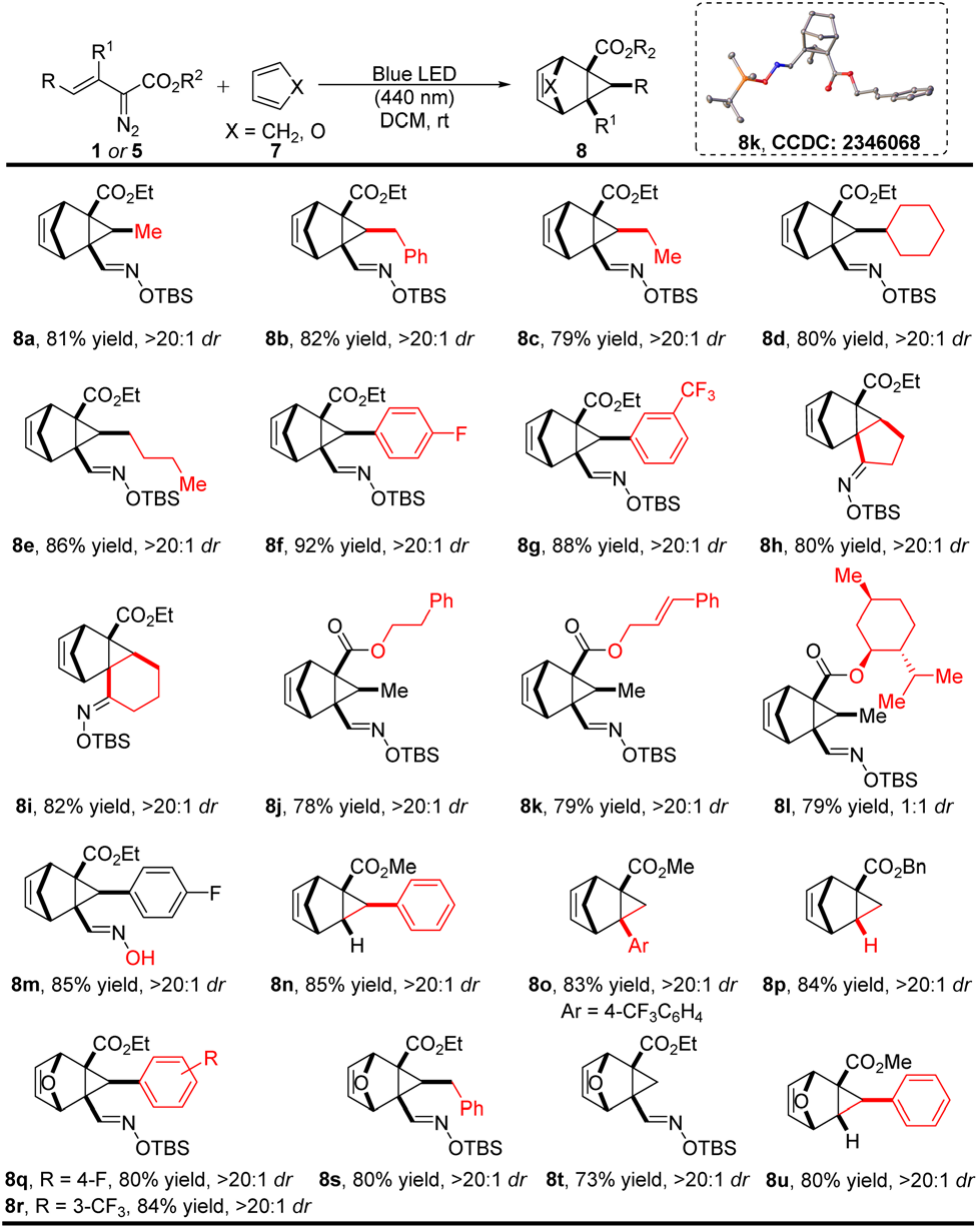
Photoinduced [4 + 2]-cycloaddition of vinyldiazo compounds with cyclopentadiene and furan. Reaction conditions: 1 (0.1 mmol) in 1.0 mL DCM was added to the solution of 7 (0.15 mmol, 1.5 equiv.) in 1.0 mL DCM *via* a syringe pump over 3.0 h with irradiation by 440 nm blue LED. Isolated yields are given.

Considering that cyclopentadiene and furan are relatively reactive C4 synthons that are well known to undergo [4 + 2]-cycloaddition reactions with various dipoles to form diverse bicyclic compounds,^16^ we turned our attention to use cyclopentadiene and furan as carbon synthons for cycloaddition with *in situ* generated unstable cyclopropanes. As anticipated, under photolysis with blue light, in the presence of cyclopentadiene and furan (7), the cyclopropene intermediate undergoes rapid [4 + 2]-cycloaddition effectively using only a 50% molar excess of 7 (Scheme 4). Oximidovinyldiazo acetates with various substitutions, including alkyl (1a–1e), aryl (1f–1g), cyclic (1h–1i), and ester (1j–1l) groups, all reacted with cyclopentadiene smoothly to form corresponding bicyclic products in high yields and diastereoselectivities. Use of the unprotected oximidovinyldiazoacetate also gave the desired product 8m in 85% yield with >20 : 1 dr. Notably, comparably high yields were obtained in the cases of methyl styryldiazoacetate, 3-aryl-2-diazo-3-butenoate, and unsubstituted benzyl 2-diazo-3-butenoate (8n–8p). Furthermore, the reaction preceded smoothly with furan as the C4 synthon, delivering the cycloaddition products 8q–8u in good yields and diastereoselectivities. The structure of these generated bicyclic products was confirmed by single-crystal X-ray diffraction analysis of compound 8k.

**Scheme 5:**
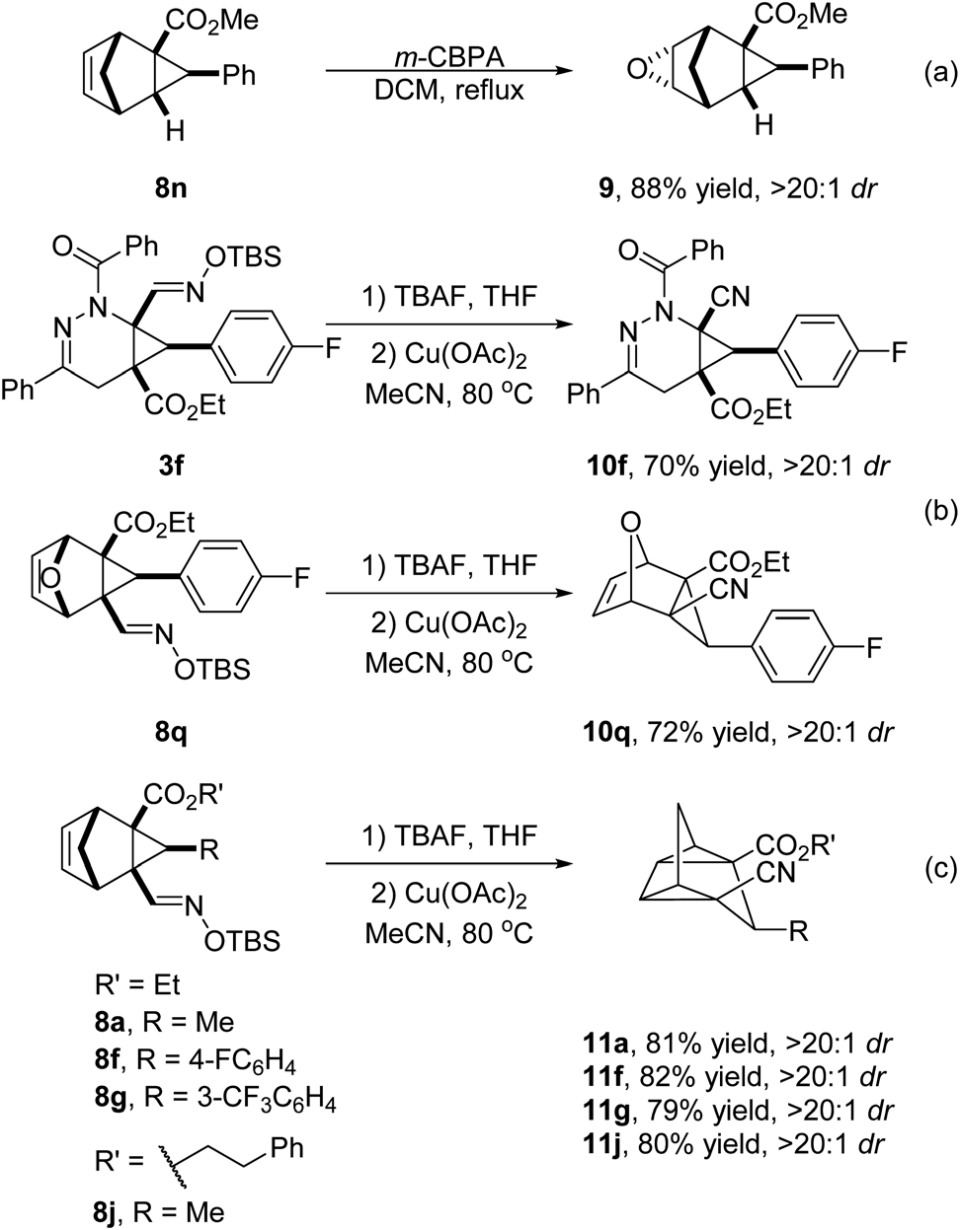
Synthetic transformations.

To demonstrate the synthetic utility of the current method, further transformations with these generated products were conducted (Scheme 5). Epoxidation of 8n with *m*-CPBA in DCM produced 9 as a single isomer in 88% yield (Scheme 5a). Notably, the oximido functional group was conveniently converted to nitriles with catalysis by copper(ii) acetate,^17^ and corresponding products 10f and 10q were obtained in 70% and 72% yields, respectively (Scheme 5b), while with 8a, 8f, 8g and 8j this functional group transformation was accompanied by valence isomerization to tetracyclic derivatives 11 in 79–82% yields (Scheme 5c). In a control experiment without copper(ii) acetate under the same conditions, the reactant was recovered unchanged. Related to the conversion of norbornadiene to quadricyclane,^18^ this homologous vinylcyclopropane rearrangement is rare but has been reported to occur at temperatures as low as 80 °C with a nickel catalyst, suggesting a free radical process.^19^

## Conclusions

In summary, we have developed a photoinduced selective [4 + 2]-cycloaddition of vinyldiazoacetates with azoalkenes from α-halohydrazones, cyclopentadiene, and furan that provides an expeditious synthesis of highly functionalized bicyclo[4.1.0]tetrahydropyridazines and tricyclic compounds in good to high yields with excellent diastereoseletivity at room temperature. The reactive cyclopropene generated selectively from vinyldiazo compounds by blue-light photolysis is the key intermediate in this cycloaddition transformation. This [4 + 2]-cycloaddition features high chemoselectivity and diastereocontrol, good functional group tolerance, and excellent scalability. Further functionalization adds additional value, including catalytic dehydration of the oximido group that can occur with structural isomerization.

## Data availability

Further experimental details, synthetic procedures, characterization data, copies of NMR spectra and X-ray crystallographic data are available in the ESI.†

## Author contributions

MB carried out the experiments. MB and ARRB prepared the starting material. HA performed the X-ray crystallography studies. MPD and MB conceived the project, wrote the manuscript, and co-wrote the ESI.†

## Conflicts of interest

There are no conflicts to declare.

## Supplementary Material

SC-015-D4SC03558E-s001

SC-015-D4SC03558E-s002
