# Supplementary material for: Photoinduced [4 + 2]-cycloaddition reactions of vinyldiazo compounds for the construction of heterocyclic and bicyclic rings
Source: Chem Sci. 2024 Jun 28;15(30):12042–6. doi: 10.1039/d4sc03558e (PMC11290432; doi:10.1039/d4sc03558e)
Supplement: SC-015-D4SC03558E-s001 [file SC-015-D4SC03558E-s001.pdf]

Electronic Supplementary Material (ESI) for Chemical Science.
This journal is © The Royal Society of Chemistry 2024

SUPPORTING INFORMATION

Photoinduced [4+2]-Cycloaddition Reactions of Vinyldiazo Compounds for the Construction of Heterocyclic and Bicyclic Rings

Ming Bao,[a] Arnold R. Romero Bohórquez,[a,b] Hadi Arman,[a] and Michael P. Doyle*[a]

[a] Department of Chemistry, The University of Texas at San Antonio, San Antonio, Texas, 78249, United States

[b] Grupo de Investigación en Compuestos Orgánicos de Interés Medicinal (CODEIM), Parque Tecnológico Guatiguará, Universidad Industrial de Santander, A.A. 678, Piedecuesta, Colombia

E-mail: michael.doyle@utsa.edu

Table of Contents

1. General Information **S2**

2. General Procedure for [4+2]-Cycloaddition with α-Halohydrazones **S3-S20**

3. General Procedure for [4+2]-Cycloaddition with Cyclopentadiene and Furan **S20-S29**

4. Synthetic Transformations and NMR Analysis **S29-S34**

5. NMR Spectra for Compounds 3, 6, 8, 9, 10 and 11 **S35-S97**

6. Single-Crystal X-ray Diffraction of 3i and 8k **S98-S100**

7. References **S100**

General Information:

Unless otherwise noted, all reactions were performed in 10 mL oven-dried (120 °C) glassware under a N_2 atmosphere. Solvents were dried using a *JC Meyer* solvent purification system. Analytical thin-layer chromatography was performed using glass plates pre-coated with 200-300 mesh silica gel impregnated with a fluorescent indicator (254 nm). Column chromatography was performed on CombiFlash® Rf200 and Rf+ purification systems using normal phase silica gel columns (300-400 mesh). High-resolution mass spectra (HRMS) were obtained on a Bruker MicroTOF-ESI mass spectrometer with an ESI resource using CsI or LTQ ESI Positive Ion Calibration Solution as the standard. Accurate masses were reported for the molecular ions $[M+H]^+$ or $[M+Na]^+$. Melting points were obtained uncorrected from an Electro Thermo Mel-Temp DLX 104 device. ^1H NMR spectra were recorded on a Bruker spectrometer (500 MHz and 300 MHz). Chemical shifts were reported in ppm downfield from tetramethylsilane (TMS) with the solvent resonance as the internal standard ($CDCl_3$, δ = 7.26). Spectra were reported as follows: chemical shift (δ ppm), multiplicity (s = singlet, d = doublet, t = triplet, q = quartet, m = multiplet, comp = composite of magnetically non-equivalent protons, dd = doublet of doublets), coupling constants (Hz), integration and assignment. ^{13}C NMR spectra were collected on Bruker instruments (125 MHz and 75 MHz) with complete proton decoupling. Chemical shifts are reported in ppm from the tetramethylsilane with the solvent resonance as internal standard ($CDCl_3$, δ = 77.16). Enantioselectivities were determined by HPLC analysis at 25 °C using an Agilent 1260 Infinity HPLC System equipped with an G1311B quaternary pump, G1315D diode array detector, G1329B auto-sampler, G1316A thermostated column compartment and G1170A valve drive. For instrument control and data processing, Agilent OpenLAB CDS ChemStation Edition for LC & LC/MS Systems (Rev. C.01.07 [26]) software was used. Chiralpak OD-H or (*R,R*-Whelk-O1) columns. The vinyldiazo compound[1] and α-chlorohydrazone[2] were prepared according

to literature procedures.

General Procedure for [4+2]-Cycloaddition with α-Halohydrazones.

To a 10-mL oven-dried vial with a magnetic stirring bar, α-halohydrazone **2** (0.15 mmol, 1.5 equiv.), Cs_2CO_3 (97.7 mg, 0.3 mmol, 3.0 equiv.) in DCM (1.0 mL) was stirring over 2.0 h at room temperature, then vinyldiazo compound **1** (0.1 mmol) in DCM (1.0 mL) was added over 2.0 h to the above solution *via* a syringe pump with irradiation by 440 nm blue LED. When the reaction was complete (monitored by TLC), the reaction mixture was purified by flash column chromatography on silica gel without additional treatment (hexanes:EtOAc = 20:1 to 15:1) to give the pure cycloaddition product.

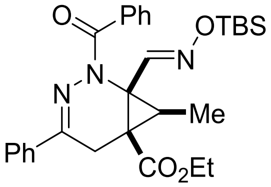

Ethyl (*E*)-2-Benzoyl-1-(*tert*-butyldimethylsilyloxy)iminomethyl-7-methyl-4-phenyl-2,3-diazabicyclo[4.1.0]hept-3-ene-6-carboxylate (3a). 43.1 mg, 83% yield, >20:1 *dr*, colorless oil; ^{1}H NMR (500 MHz, $CDCl_3$) (δ, ppm) 7.82 (s, 1H), 7.76 (d, J = 7.8 Hz, 2H), 7.68 (d, J = 7.5 Hz, 2H), 7.48 – 7.42 (m, 1H), 7.40 – 7.34 (comp, 5H), 4.22 – 4.14 (comp, 2H), 3.92 (d, J = 17.3 Hz, 1H), 2.29 (d, J = 17.3 Hz, 1H), 1.73 (q, J = 6.6 Hz, 1H), 1.62 (d, J = 6.6 Hz, 3H), 1.24 (t, J = 7.0 Hz, 3H), 0.84 (s, 9H), 0.08 (s, 3H), 0.05 (s, 3H); ^{13}C NMR (125 MHz, $CDCl_3$) (δ, ppm) 169.8, 169.2, 152.8, 150.0, 135.9, 135.0, 130.6, 130.1, 130.0, 128.6, 127.5, 126.1, 61.8, 44.2, 35.8, 34.4, 28.0, 26.2, 18.3, 14.3, 9.1, -5.1, -5.2; HRMS (ESI Q-TOF) m/z: $[M+H]^+$ Calcd for $C_{29}H_{38}N_3O_4Si$ 520.2626, found 520.2628.

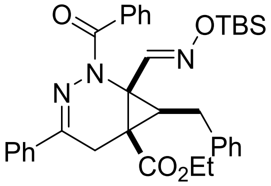

Ethyl 2-Benzoyl-7-benzyl-1-(*E*)-(*tert*-butyldimethylsilyloxy)iminomethyl-4-phenyl-2,3-diazabicyclo[4.1.0]hept-3-ene-6-carboxylate (3b). 40.5 mg, 68% yield, >20:1 *dr*, colorless oil; ^{1}H NMR (500 MHz, $CDCl_3$) (δ, ppm) 7.98 (s, 1H), 7.75 (d, J = 7.3 Hz, 2H), 7.66 (d, J = 7.3 Hz, 2H), 7.49 – 7.43 (m, 1H), 7.42 – 7.28 (comp,

9H), 7.24 – 7.21 (m, 1H), 4.22 – 4.13 (comp, 2H), 3.94 (d, J = 17.4 Hz, 1H), 3.51 – 3.47 (m, 1H), 3.38 – 3.33 (m, 1H), 2.30 (d, J = 17.4 Hz, 1H), 1.95 – 1.92 (m, 1H), 1.21 (t, J = 7.1 Hz, 3H), 0.85 (s, 9H), 0.09 (s, 3H), 0.07 (s, 3H); ^{13}C NMR (125 MHz, $CDCl_3$) (δ, ppm) 170.1, 169.1, 152.0, 150.2, 140.0, 135.8, 135.0, 130.7, 130.1, 130.0, 128.8, 128.64, 128.61, 127.5, 126.5, 126.0, 62.0, 44.5, 41.7, 34.2, 29.7, 27.8, 26.2, 18.3, 14.2, -5.1, -5.2; HRMS (ESI Q-TOF) m/z: $[M+H]^+$ Calcd for $C_{35}H_{42}N_3O_4Si$ 596.2939, found 596.2939.

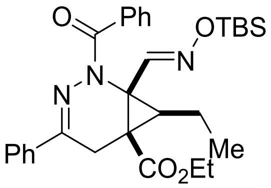

Ethyl 2-Benzoyl-1-(*E*)-(*tert*-butyldimethylsilyloxy)iminomethyl-7-ethyl-4-phenyl-2,3-diazabicyclo[4.1.0]hept-3-ene-6-carboxylate (3c). 38.4 mg, 72% yield, >20:1 *dr*, colorless oil; ^{1}H NMR (500 MHz, $CDCl_3$) (δ, ppm) 7.80 (s, 1H), 7.75 (d, J = 7.5 Hz, 2H), 7.68 (d, J = 7.8 Hz, 2H), 7.46 – 7.41 (comp, 2H), 7.40 – 7.35 (comp, 4H), 4.22 – 4.13 (comp, 2H), 3.92 (d, J = 17.2 Hz, 1H), 2.30 (d, J = 17.2 Hz, 1H), 2.09 – 2.04 (m, 1H), 1.99 – 1.93 (m, 1H), 1.61 (t, J = 7.5 Hz, 1H), 1.24 (t, J = 7.1 Hz, 3H), 1.18 (t, J = 7.3 Hz, 3H), 0.84 (s, 9H), 0.08 (s, 3H), 0.05 (s, 3H); ^{13}C NMR (125 MHz, $CDCl_3$) (δ, ppm) 169.9, 169.3, 152.8, 150.2, 135.9, 135.1, 130.6, 130.1, 130.0, 128.6, 127.5, 126.1, 61.8, 44.3, 42.9, 34.3, 28.3, 26.2, 18.3, 17.6, 14.2, 13.6, -5.1, -5.2; HRMS (ESI Q-TOF) m/z: $[M+H]^+$ Calcd for $C_{30}H_{40}N_3O_4Si$ 534.2783, found 534.2785.

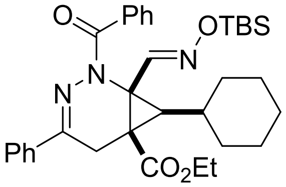

Ethyl 2-Benzoyl-1-(*E*)-(*tert*-butyldimethylsilyloxy)iminomethyl-7-cyclohexyl-4-phenyl-2,3-diazabicyclo[4.1.0]hept-3-ene-6-carboxylate (3d). 47.0 mg, 80% yield, >20:1 *dr*, colorless oil; ^{1}H NMR (500 MHz, $CDCl_3$) (δ, ppm) 7.83 (s, 1H), 7.75 (d, J = 7.6 Hz, 2H), 7.71 (d, J = 7.1 Hz, 2H), 7.48 – 7.45 (m, 1H), 7.41 – 7.37 (comp, 5H), 4.20 (q, J = 6.9 Hz, 2H), 3.97 (d, J = 17.2 Hz, 1H), 2.37 (d, J = 12.4 Hz, 1H), 2.30 (d, J = 17.2 Hz, 1H), 1.98 (q, J = 10.9 Hz, 1H), 1.83 (d, J = 12.5 Hz, 1H), 1.78 – 1.69 (comp, 3H), 1.45 (d, J = 10.9 Hz, 1H), 1.38 – 1.30 (m, 1H), 1.27 – 1.24 (comp, 5H),

1.22 – 1.16 (comp, 2H), 0.86 (s, 9H), 0.10 (s, 3H), 0.08 (s, 3H); ^{13}C NMR (125 MHz, $CDCl_3$) (δ, ppm) 169.8, 169.4, 152.4, 150.4, 135.9, 135.1, 130.6, 130.1, 129.9, 128.6, 127.4, 126.0, 61.8, 47.3, 44.4, 34.0, 33.2, 32.9, 32.8, 28.4, 26.4, 26.2, 26.0, 18.3, 14.3, -5.1, -5.2; HRMS (ESI Q-TOF) m/z: $[M+H]^+$ Calcd for $C_{34}H_{46}N_3O_4Si$ 588.3252, found 588.3255.

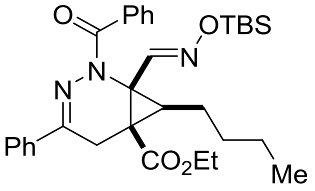

Ethyl 2-Benzoyl-7-butyl-1-(*E*)-(*tert*-butyldimethylsilyloxy)iminomethyl-4-phenyl-2,3-diazabicyclo[4.1.0]hept-3-ene-6-carboxylate (3e). 39.9 mg, 71% yield, >20:1 *dr*, colorless oil; ^{1}H NMR (500 MHz, $CDCl_3$) (δ, ppm) 7.81 (s, 1H), 7.74 (d, J = 7.4 Hz, 2H), 7.68 (d, J = 7.3 Hz, 2H), 7.46 – 7.41 (m, 1H), 7.43 – 7.35 (comp, 4H), 7.27 – 7.26 (m, 1H), 4.25 – 4.11 (comp, 2H), 3.92 (d, J = 17.2 Hz, 1H), 2.30 (d, J = 17.2 Hz, 1H), 2.07 – 2.01 (m, 1H), 1.96 – 1.87 (m, 1H), 1.63 (t, J = 7.1 Hz, 2H), 1.53 – 1.44 (m, 1H), 1.42 – 1.38 (comp, 2H), 1.26 – 1.22 (comp, 3H), 0.94 (t, J = 7.0 Hz, 3H), 0.84 (s, 9H), 0.07 (s, 3H), 0.05 (s, 3H); ^{13}C NMR (125 MHz, $CDCl_3$) (δ, ppm) 169.9, 169.3, 152.6, 150.3, 135.9, 135.1, 130.6, 130.1, 130.0, 128.6, 127.5, 126.1, 61.8, 44.3, 41.4, 34.3, 31.3, 28.2, 26.2, 23.8, 22.7, 18.3, 14.3, 14.2, -5.1, -5.2; HRMS (ESI Q-TOF) m/z: $[M+H]^+$ Calcd for $C_{32}H_{44}N_3O_4Si$ 562.3096, found 562.3100.

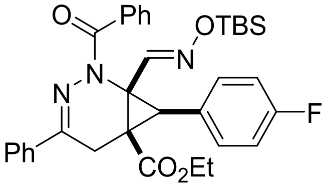

Ethyl 2-Benzoyl-1-(*E*)-(*tert*-butyldimethylsilyloxy)iminomethyl-7-(4-fluoro-phenyl)-4-phenyl-2,3-diazabicyclo[4.1.0]hept-3-ene-6-carboxylate (3f). 51.5 mg, 88% yield, >20:1 *dr*, white solid, mp: 100.0-102.0 °C; ^{1}H NMR (500 MHz, $CDCl_3$) (δ, ppm) 7.84 (d, J = 7.5 Hz, 2H), 7.73 (d, J = 7.4 Hz, 2H), 7.66 (s, 1H), 7.60 – 7.53 (comp, 2H), 7.48 (t, J = 7.4 Hz, 1H), 7.43 (t, J = 7.2 Hz, 2H), 7.40 – 7.38 (comp, 3H), 7.09 – 7.06 (comp, 2H), 4.15 (d, J = 17.5 Hz, 1H), 4.10 – 4.05 (m, 1H), 4.00 – 3.86 (m, 1H), 2.95 (s, 1H), 2.58 (d, J = 17.5 Hz, 1H), 1.02 (t, J = 7.1 Hz, 3H), 0.82 (s, 9H), 0.06 (s, 3H), 0.04 (s, 3H); ^{13}C NMR (125 MHz, $CDCl_3$) (δ, ppm) 170.2, 168.8, 162.4 (d, J = 246.7

Hz), 150.6, 135.8, 134.8, 131.9 (d, *J* = 8.1 Hz), 130.9, 130.2, 130.1, 128.7, 128.6, 127.9 (d, *J* = 3.1 Hz), 127.5, 126.1, 115.5 (d, *J* = 21.5 Hz), 62.0, 44.4, 43.3, 35.0, 27.3, 26.2, 18.3, 13.9, -5.16, -5.20; HRMS (ESI Q-TOF) m/z: $[M+H]^+$ Calcd for $C_{34}H_{39}FN_3O_4Si$ 600.2688, found 600.2691.

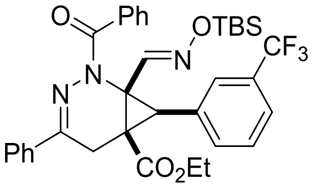

Ethyl 2-Benzoyl-1-(*E*)-(*tert*-butyldimethylsilyloxy)iminomethyl-4-phenyl-7-(3-(trifluoromethyl)phenyl)-2,3-diazabicyclo[4.1.0]hept-3-ene-6-carboxylate (3g). 55.2 mg, 85% yield, >20:1 *dr*, colorless oil; ^{1}H NMR (500 MHz, $CDCl_3$) (δ, ppm) 8.03 (d, *J* = 7.5 Hz, 1H), 7.85 (d, *J* = 7.4 Hz, 2H), 7.77 – 7.71 (comp, 2H), 7.64 (s, 1H), 7.62 (s, 1H), 7.60 – 7.55 (comp, 2H), 7.49 (t, *J* = 7.0 Hz, 1H), 7.43 (t, *J* = 7.5 Hz, 2H), 7.40 – 7.37 (comp, 3H), 4.17 (d, *J* = 17.6 Hz, 1H), 4.12 – 4.04 (m, 1H), 4.02 – 3.89 (m, 1H), 3.02 (s, 1H), 2.61 (d, *J* = 17.6 Hz, 1H), 1.00 (t, *J* = 7.1 Hz, 3H), 0.81 (s, 9H), 0.05 (s, 3H), 0.03 (s, 3H); ^{13}C NMR (125 MHz, $CDCl_3$) (δ, ppm) 170.3, 168.5, 151.3, 150.2, 135.7, 134.7, 134.2, 133.3, 131.0, 130.9, 130.7, 130.2, 129.3, 128.7, 127.6, 126.8 (q, *J* = 3.4 Hz), 126.1, 125.6 (q, *J* = 272.4 Hz), 124.6 (q, *J* = 3.4 Hz), 62.2, 44.3, 43.3, 35.0, 27.2, 26.1, 18.3, 13.8, -5.18, -5.23; HRMS (ESI Q-TOF) m/z: $[M+H]^+$ Calcd for $C_{35}H_{39}F_3N_3O_4Si$ 650.2656, found 650.2658.

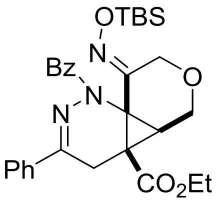

Ethyl (*Z*)-1-Benzoyl-8-(*tert*-butyldimethylsilyloxy)imino-3-phenyl-1,4,4b,5,7,8-hexahydro-4a*H*-pyrano[4',3':1,3]cyclopropa[1,2-*c*]pyridazine-4a-carboxylate (3h). 27.9 mg, 51% yield, >20:1 *dr*, colorless oil; ^{1}H NMR (500 MHz, $CDCl_3$) (δ, ppm) 7.78 (d, *J* = 7.9 Hz, 2H), 7.63 (d, *J* = 7.7 Hz, 2H), 7.50 (t, *J* = 7.1 Hz, 1H), 7.45 – 7.43 (comp, 2H), 7.39 – 7.33 (comp, 3H), 4.78 (d, *J* = 17.2 Hz, 1H), 4.64 (dd, *J* = 12.6, 5.9 Hz, 1H), 4.33 – 4.29 (m, 1H), 4.23 – 4.21 (m, 1H), 4.16 (d, *J* = 17.2 Hz, 1H), 4.08 – 4.04 (m, 1H), 3.67 (d, *J* = 17.9 Hz, 1H), 2.70 (d, *J* = 17.9 Hz, 1H), 2.16 – 2.13 (m, 1H), 1.19 (t, *J* = 7.0 Hz, 3H), 0.86 (s, 9H), 0.12 (s, 3H), 0.07 (s, 3H); ^{13}C NMR (125 MHz,

$CDCl_3$) (δ, ppm) 171.5, 167.5, 158.0, 150.3, 135.9, 134.6, 131.0, 130.0, 129.9, 128.7, 127.7, 126.1, 63.9, 61.8, 59.4, 44.2, 35.5, 29.3, 26.1, 26.0, 18.2, 14.2, -5.25, -5.28; HRMS (ESI Q-TOF) m/z: $[M+H]^+$ Calcd for $C_{30}H_{38}N_3O_5Si$ 548.2575, found 548.2578.

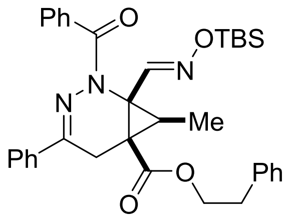

Phenethyl 2-Benzoyl-1-(*E*)-(*tert*-butyldimethylsilyloxy)iminomethyl-7-methyl-4-phenyl-2,3-diazabicyclo[4.1.0]hept-3-ene-6-carboxylate (3i). 35.7 mg, 60% yield, >20:1 *dr*, white solid, mp: 102.0-104.0 °C; ^{1}H NMR (500 MHz, $CDCl_3$) (δ, ppm) 7.81 (s, 1H), 7.76 (d, J = 7.2 Hz, 2H), 7.60 (d, J = 7.2 Hz, 2H), 7.46 – 7.43 (m, 1H), 7.40 – 7.32 (comp, 5H), 7.29 – 7.26 (comp, 2H), 7.23 – 7.19 (comp, 3H), 4.43 – 4.24 (comp, 2H), 3.81 (d, J = 17.4 Hz, 1H), 2.92 (t, J = 6.9 Hz, 2H), 2.24 (d, J = 17.4 Hz, 1H), 1.68 (q, J = 6.6 Hz, 1H), 1.50 (d, J = 6.6 Hz, 3H), 0.85 (s, 9H), 0.09 (s, 3H), 0.06 (s, 3H); ^{13}C NMR (125 MHz, $CDCl_3$) (δ, ppm) 169.8, 169.2, 152.3, 150.1, 137.5, 135.8, 135.0, 130.7, 130.1, 130.0, 128.9, 128.8, 128.6, 127.5, 126.9, 126.1, 66.2, 44.1, 35.9, 35.0, 34.2, 27.7, 26.2, 18.3, 8.9, -5.09, -5.13; HRMS (ESI Q-TOF) m/z: $[M+H]^+$ Calcd for $C_{35}H_{42}N_3O_4Si$ 596.2939, found 596.2944.

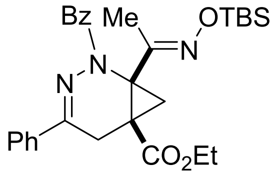

Ethyl 2-Benzoyl-1-(*E*)-1-(*tert*-butyldimethylsilyloxy)iminoethyl-4-phenyl-2,3-diazabicyclo[4.1.0]hept-3-ene-6-carboxylate (3j). 37.9 mg, 73% yield, >20:1 *dr*, colorless oil; ^{1}H NMR (500 MHz, $CDCl_3$) (δ, ppm) 7.74 (d, J = 7.6 Hz, 2H), 7.69 (d, J = 7.6 Hz, 2H), 7.51 – 7.47 (m, 1H), 7.43 (d, J = 7.3 Hz, 2H), 7.40 – 7.37 (comp, 3H), 4.13 (q, J = 7.0 Hz, 2H), 3.89 (d, J = 17.7 Hz, 1H), 2.75 (d, J = 6.2 Hz, 1H), 2.42 (d, J = 17.7 Hz, 1H), 2.03 (s, 3H), 1.27 (d, J = 6.2 Hz, 1H), 1.23 (t, J = 7.0 Hz, 3H), 0.90 (s, 9H), 0.13 (s, 3H), 0.10 (s, 3H); ^{13}C NMR (125 MHz, $CDCl_3$) (δ, ppm) 171.2, 170.0, 157.6, 149.8, 136.0, 135.1, 133.8, 130.7, 129.9, 128.7, 127.6, 126.0, 62.0, 45.2, 30.6, 26.2, 25.8, 23.2, 18.2, 14.2, 13.9, -5.0, -5.1; HRMS (ESI Q-TOF) m/z: $[M+H]^+$ Calcd for $C_{29}H_{38}N_3O_4Si$ 520.2626, found 520.2630.

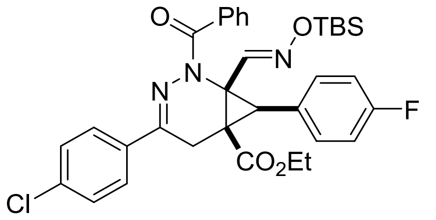

Ethyl 2-Benzoyl-1-(*E*)-(*tert*-butyldimethylsilyloxy)iminomethyl-4-(4-chlorophenyl)-7-(4-fluorophenyl)-2,3-diazabicyclo[4.1.0]hept-3-ene-6-carboxylate (3k). 51.3 mg, 81% yield, >20:1 *dr*, white solid, mp: 101.0-103.0 °C; ^{1}H NMR (300 MHz, $CDCl_3$) (δ, ppm) 7.80 (d, *J* = 7.4 Hz, 2H), 7.65 (s, 1H), 7.60 – 7.53 (comp, 4H), 7.52 – 7.46 (comp, 3H), 7.44 – 7.41 (comp, 2H), 7.07 (t, *J* = 8.6 Hz, 2H), 4.13 – 4.02 (comp, 2H), 3.99 – 3.91 (m, 1H), 2.94 (s, 1H), 2.58 (d, *J* = 17.7 Hz, 1H), 1.01 (t, *J* = 7.1 Hz, 3H), 0.80 (s, 9H), 0.05 (s, 3H), 0.02 (s, 3H); ^{13}C NMR (125 MHz, $CDCl_3$) (δ, ppm) 170.3, 168.7, 162.4 (d, *J* = 246.7 Hz), 150.5, 149.7, 134.7 (d, *J* = 16.1 Hz), 132.1, 132.0, 131.90, 131.85, 131.0, 130.1, 127.7 (d, *J* = 2.9 Hz), 127.6 (d, *J* = 10.2 Hz), 124.5, 115.6 (d, *J* = 21.5 Hz), 62.1, 44.3, 43.2, 34.8, 27.0, 26.1, 18.3, 13.9, -5.17, -5.21; HRMS (ESI Q-TOF) m/z: $[M+H]^+$ Calcd for $C_{34}H_{38}ClFN_3O_4Si$ 634.2299, found 634.2300.

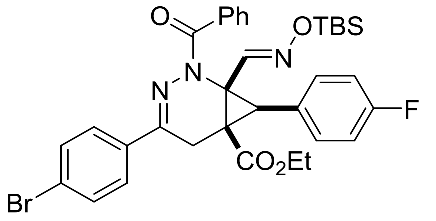

Ethyl 2-Benzoyl-4-(4-bromophenyl)-1-(*E*)-(*tert*-butyldimethylsilyloxy)iminomethyl-7-(4-fluorophenyl)-2,3-diazabicyclo[4.1.0]hept-3-ene-6-carboxylate (3l). 56.2 mg, 83% yield, >20:1 *dr*, white solid, mp: 103.0-105.0 °C; ^{1}H NMR (300 MHz, $CDCl_3$) (δ, ppm) 7.81 (d, *J* = 7.7 Hz, 2H), 7.65 – 7.63 (comp, 3H), 7.59 – 7.52 (comp, 2H), 7.49 (t, *J* = 7.2 Hz, 1H), 7.44 – 7.41 (comp, 2H), 7.34 (d, *J* = 8.4 Hz, 2H), 7.09 – 7.05 (comp, 2H), 4.13 – 4.03 (comp, 2H), 3.98 – 3.91 (m, 1H), 2.94 (s, 1H), 2.57 (d, *J* = 17.6 Hz, 1H), 1.01 (t, *J* = 7.1 Hz, 3H), 0.80 (s, 9H), 0.05 (s, 3H), 0.02 (s, 3H); ^{13}C NMR (125 MHz, $CDCl_3$) (δ, ppm) 170.3, 168.8, 162.4 (d, *J* = 246.9 Hz), 150.5, 149.7, 136.2, 134.7, 134.3, 131.9 (d, *J* = 8.0 Hz), 131.0, 130.1, 129.0, 127.8 (d, *J* = 2.8 Hz), 127.6, 127.3, 115.6 (d, *J* = 21.6 Hz), 62.1, 44.3, 43.2, 34.9, 27.1, 26.1, 18.3, 13.9, -5.16, -5.21; HRMS (ESI Q-TOF) m/z: $[M+H]^+$ Calcd for $C_{34}H_{38}BrFN_3O_4Si$ 678.1794, found 678.1800.

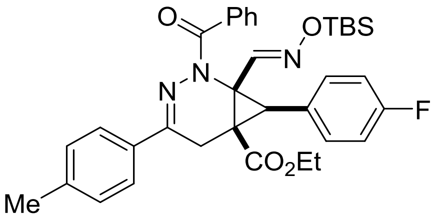

Ethyl 2-Benzoyl-1-(*E*)-(*tert*-butyldimethylsilyloxy)iminomethyl-7-(4-fluoro-phenyl)-4-(*p*-tolyl)-2,3-diazabicyclo[4.1.0]hept-3-ene-6-carboxylate (3m). 48.4 mg, 79% yield, >20:1 *dr*, colorless oil; ^{1}H NMR (500 MHz, $CDCl_3$) (δ, ppm) 7.83 (d, *J* = 7.2 Hz, 2H), 7.67 – 7.60 (comp, 3H), 7.59 – 7.54 (comp, 2H), 7.47 (t, *J* = 7.3 Hz, 1H), 7.41 (t, *J* = 7.5 Hz, 2H), 7.18 (d, *J* = 8.0 Hz, 2H), 7.07 (t, *J* = 8.6 Hz, 2H), 4.14 (d, *J* = 17.5 Hz, 1H), 4.09 – 4.04 (m, 1H), 3.98 – 3.92 (m, 1H), 2.95 (s, 1H), 2.52 (d, *J* = 17.5 Hz, 1H), 2.37 (s, 3H), 1.01 (t, *J* = 7.1 Hz, 3H), 0.81 (s, 9H), 0.05 (s, 3H), 0.03 (s, 3H); ^{13}C NMR (125 MHz, $CDCl_3$) (δ, ppm) 170.1, 168.8, 162.4 (d, *J* = 246.5 Hz), 151.9, 150.5, 140.4, 134.8, 133.0, 131.9 (d, *J* = 8.0 Hz), 130.8, 130.2, 129.4, 127.9 (d, *J* = 2.9 Hz), 127.5, 126.1, 115.5 (d, *J* = 21.5 Hz), 62.0, 44.5, 43.4, 35.2, 27.4, 26.2, 21.5, 18.3, 13.9, -5.16, -5.20; HRMS (ESI Q-TOF) m/z: $[M+H]^+$ Calcd for $C_{35}H_{41}FN_3O_4Si$ 614.2845, found 614.2846.

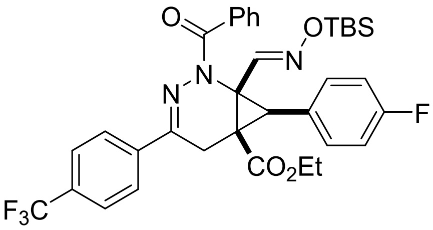

Ethyl 2-Benzoyl-1-(*E*)-(*tert*-butyldimethylsilyloxy)iminomethyl-7-(4-fluoro-phenyl)-4-(4-(trifluoromethyl)phenyl)-2,3-diazabicyclo[4.1.0]hept-3-ene-6-carboxylate (3n). 53.4 mg, 80% yield, >20:1 *dr*, colorless oil; ^{1}H NMR (500 MHz, $CDCl_3$) (δ, ppm) 7.82 – 7.79 (comp, 4H), 7.67 (s, 1H), 7.62 (d, *J* = 8.3 Hz, 2H), 7.57 – 7.55 (comp, 2H), 7.52 – 7.49 (m, 1H), 7.43 (t, *J* = 7.5 Hz, 2H), 7.10 – 7.06 (comp, 2H), 4.16 – 4.02 (comp, 2H), 3.98 – 3.92 (m, 1H), 2.95 (s, 1H), 2.68 (d, *J* = 17.9 Hz, 1H), 1.01 (t, *J* = 7.1 Hz, 3H), 0.81 (s, 9H), 0.04 (s, 3H), 0.02 (s, 3H); ^{13}C NMR (125 MHz, $CDCl_3$) (δ, ppm) 170.6, 168.7, 162.4 (d, *J* = 246.9 Hz), 150.6, 148.3, 139.2, 134.5, 131.9 (d, *J* = 8.1 Hz), 131.6 (q, *J* = 32.6 Hz), 131.1, 130.1, 127.7, 126.2, 125.8 (d, *J* = 2.9 Hz), 125.7 (q, *J* = 3.5 Hz), 124.1 (q, *J* = 272.4 Hz), 115.6 (d, *J* = 21.5 Hz), 62.2, 44.1, 43.0, 34.6, 26.8, 26.1, 18.3, 13.9, -5.17, -5.21; HRMS (ESI Q-TOF) m/z: $[M+H]^+$ Calcd for

$C_{35}H_{38}F_4N_3O_4Si$ 668.2562, found 668.2562.

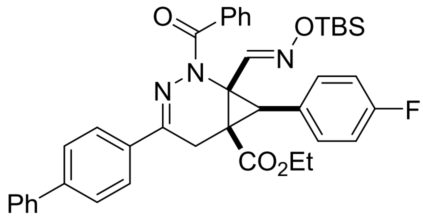

Ethyl 4-([1,1'-Biphenyl]-4-yl)-2-benzoyl-1-(*E*)-(*tert*-butyldimethylsilyloxy)-iminomethyl-7-(4-fluorophenyl)-2,3-diazabicyclo[4.1.0]hept-3-ene-6-carboxylate (3o). 47.3 mg, 70% yield, >20:1 *dr*, colorless oil; ^{1}H NMR (500 MHz, $CDCl_3$) (δ, ppm) 7.85 (d, *J* = 7.8 Hz, 2H), 7.80 (d, *J* = 8.2 Hz, 2H), 7.66 (s, 1H), 7.63 – 7.56 (comp, 6H), 7.51 – 7.47 (comp, 2H), 7.46 – 7.42 (comp, 3H), 7.37 (t, *J* = 7.3 Hz, 1H), 7.08 (t, *J* = 8.5 Hz, 2H), 4.18 (d, *J* = 17.5 Hz, 1H), 4.12 – 4.06 (m, 1H), 3.99 – 3.93 (m, 1H), 2.97 (s, 1H), 2.61 (d, *J* = 17.5 Hz, 1H), 1.02 (t, *J* = 7.1 Hz, 3H), 0.82 (s, 9H), 0.06 (s, 3H), 0.04 (s, 3H); ^{13}C NMR (125 MHz, $CDCl_3$) (δ, ppm) 170.2, 168.9, 162.4 (d, *J* = 246.7 Hz), 151.0, 150.6, 142.8, 140.3, 134.7 (d, *J* = 12.2 Hz), 131.9 (d, *J* = 8.1 Hz), 130.9, 130.2, 129.0, 127.91, 127.88, 127.6, 127.4, 127.2, 127.1, 126.5, 115.6 (d, *J* = 21.5 Hz), 62.1, 44.5, 43.3 35.0, 27.3, 26.2, 18.3, 13.9, -5.1, -5.2; HRMS (ESI Q-TOF) m/z: $[M+H]^+$ Calcd for $C_{40}H_{43}FN_3O_4Si$ 676.3001, found 676.3002.

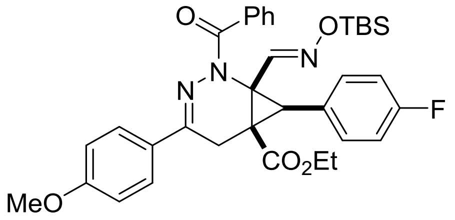

Ethyl 2-Benzoyl-1-(*E*)-(*tert*-butyldimethylsilyloxy)iminomethyl-7-(4-fluoro-phenyl)-4-(4-methoxyphenyl)-2,3-diazabicyclo[4.1.0]hept-3-ene-6-carboxylate (3p). 47.2 mg, 75% yield, >20:1 *dr*, white solid; ^{1}H NMR (500 MHz, $CDCl_3$) (δ, ppm) 7.82 (d, *J* = 7.7 Hz, 2H), 7.69 (d, *J* = 8.2 Hz, 2H), 7.63 (s, 1H), 7.60 – 7.52 (comp, 2H), 7.47 (t, *J* = 7.2 Hz, 1H), 7.41 (t, *J* = 7.5 Hz, 2H), 7.08 – 7.05 (comp, 2H), 6.90 (d, *J* = 8.2 Hz, 2H), 4.15 (d, *J* = 17.3 Hz, 1H), 4.08 – 4.03 (m, 1H), 3.98 – 3.90 (m, 1H), 3.83 (s, 3H), 2.97 (s, 1H), 2.47 (d, *J* = 17.3 Hz, 1H), 1.01 (t, *J* = 7.1 Hz, 3H), 0.81 (s, 9H), 0.05 (s, 3H), 0.03 (s, 3H); ^{13}C NMR (125 MHz, $CDCl_3$) (δ, ppm) 169.9, 168.9, 162.4 (d, *J* = 246.5 Hz), 161.3, 152.3, 150.5, 134.9, 131.9 (d, *J* = 8.0 Hz), 130.8, 130.1, 128.3, 128.0 (d, *J* = 2.9 Hz), 127.7, 127.5, 115.5 (d, *J* = 21.5 Hz), 114.1, 62.0, 55.5, 44.8, 43.5, 35.4, 27.6, 26.2, 18.3, 13.9, -5.15, -5.19; HRMS (ESI Q-TOF) m/z: $[M+H]^+$ Calcd for

$C_{35}H_{41}FN_3O_5Si$ 630.2794, found 630.2797.

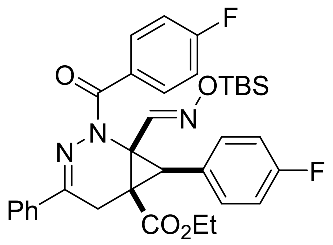

Ethyl 1-(*E*)-(*tert*-Butyldimethylsilyloxy)iminomethyl-2-(4-fluorobenzoyl)-7-(4-fluorophenyl)-4-phenyl-2,3-diazabicyclo[4.1.0]hept-3-ene-6-carboxylate (3q). 51.2 mg, 83% yield, >20:1 *dr*, colorless oil; ^{1}H NMR (500 MHz, $CDCl_3$) (δ, ppm) 7.91 – 7.88 (comp, 2H), 7.80 – 7.70 (comp, 2H), 7.64 (s, 1H), 7.57 – 7.54 (comp, 2H), 7.44 – 7.35 (comp, 3H), 7.12 – 7.05 (comp, 4H), 4.14 (d, J = 17.6 Hz, 1H), 4.11 – 4.05 (m, 1H), 3.99 – 3.92 (m, 1H), 2.95 (s, 1H), 2.58 (d, J = 17.6 Hz, 1H), 1.02 (t, J = 7.1 Hz, 3H), 0.80 (s, 9H), 0.04 (s, 3H), 0.02 (s, 3H); ^{13}C NMR (125 MHz, $CDCl_3$) (δ, ppm) 169.1, 168.7, 164.4 (d, J = 247.9 Hz), 162.4 (d, J = 243.3 Hz), 151.5, 150.5, 135.7, 132.7 (d, J = 8.7 Hz), 131.9 (d, J = 8.1 Hz), 130.7 (d, J = 3.0 Hz), 130.2, 128.8, 127.8 (d, J = 2.9 Hz), 126.0, 115.6 (d, J = 21.5 Hz), 114.7 (d, J = 21.7 Hz), 62.1, 44.4, 43.2, 34.9, 27.3, 26.1, 18.3, 13.9, -5.18, -5.22; HRMS (ESI Q-TOF) m/z: $[M+H]^+$ Calcd for $C_{34}H_{38}F_2N_3O_4Si$ 618.2594, found 618.2596.

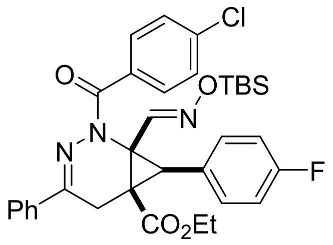

Ethyl 1-(*E*)-(*tert*-Butyldimethylsilyloxy)iminomethyl-2-(4-chlorobenzoyl)-7-(4-fluorophenyl)-4-phenyl-2,3-diazabicyclo[4.1.0]hept-3-ene-6-carboxylate (3r). 53.2 mg, 84% yield, >20:1 *dr*, white solid; ^{1}H NMR (500 MHz, $CDCl_3$) (δ, ppm) 7.81 (d, J = 8.3 Hz, 2H), 7.75 – 7.69 (comp, 2H), 7.63 (s, 1H), 7.56 – 7.54 (comp, 2H), 7.41 – 7.40 (comp, 5H), 7.07 (t, J = 8.5 Hz, 2H), 4.15 (d, J = 17.6 Hz, 1H), 4.11 – 4.05 (m, 1H), 3.99 – 3.92 (m, 1H), 2.95 (s, 1H), 2.57 (d, J = 17.6 Hz, 1H), 1.02 (t, J = 7.1 Hz, 3H), 0.81 (s, 9H), 0.04 (s, 3H), 0.02 (s, 3H); ^{13}C NMR (125 MHz, $CDCl_3$) (δ, ppm) 169.1, 168.7, 162.4 (d, J = 246.8 Hz), 151.8, 150.4, 137.0, 135.6, 133.1, 131.9 (d, J = 8.1 Hz), 131.7, 130.3, 128.8, 127.9, 127.8 (d, J = 2.9 Hz), 126.1, 115.6 (d, J = 21.6 Hz), 62.1, 44.4, 43.3, 34.9, 27.3, 26.2, 18.3, 13.9, -5.15, -5.20; HRMS (ESI Q-TOF) m/z: $[M+H]^+$ Calcd for $C_{34}H_{38}ClFN_3O_4Si$ 634.2299, found 634.2300.

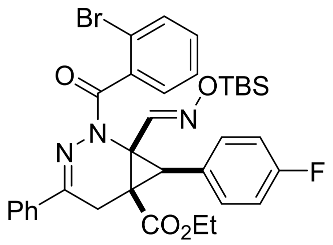

Ethyl 2-(2-Bromobenzoyl)-1-(*E*)-(*tert*-butyldimethylsilyloxy)iminomethyl-7-(4-fluorophenyl)-4-phenyl-2,3-diazabicyclo[4.1.0]hept-3-ene-6-carboxylate (3s). 54.2 mg, 80% yield, >20:1 *dr*, white solid; ^{1}H NMR (500 MHz, $CDCl_3$) (δ, ppm) 7.67 (s, 1H), 7.57 – 7.54 (comp, 4H), 7.53 – 7.49 (comp, 2H), 7.42 (t, *J* = 7.4 Hz, 1H), 7.37 – 7.26 (comp, 4H), 7.08 (t, *J* = 8.4 Hz, 2H), 4.20 (d, *J* = 17.5 Hz, 1H), 4.10 – 4.04 (m, 1H), 3.99 – 3.93 (m, 1H), 3.07 (s, 1H), 2.52 (d, *J* = 17.5 Hz, 1H), 1.02 (t, *J* = 7.1 Hz, 3H), 0.86 (s, 9H), 0.15 (s, 3H), 0.14 (s, 3H); ^{13}C NMR (125 MHz, $CDCl_3$) (δ, ppm) 168.7, 168.4, 162.4 (d, *J* = 246.9 Hz), 154.7, 150.2, 138.6, 135.6, 132.1 (d, *J* = 3.3 Hz), 132.0, 130.3 (d, *J* = 10.1 Hz), 129.3, 128.7, 128.6, 127.7 (d, *J* = 2.7 Hz), 127.2, 126.3, 120.5, 115.5 (d, *J* = 21.5 Hz), 62.1, 43.8, 43.7, 35.4, 27.9, 26.2, 18.3, 13.9, -5.00, -5.02; HRMS (ESI Q-TOF) m/z: $[M+H]^+$ Calcd for $C_{34}H_{38}BrFN_3O_4Si$ 678.1794, found 678.1794.

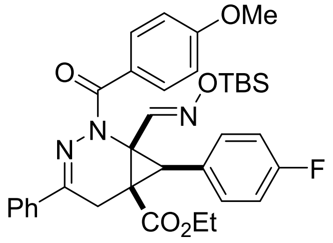

Ethyl 1-(*E*)-(*tert*-Butyldimethylsilyloxy)iminomethyl-7-(4-fluorophenyl)-2-(4-methoxybenzoyl)-4-phenyl-2,3-diazabicyclo[4.1.0]hept-3-ene-6-carboxylate (3t). 53.5 mg, 85% yield, >20:1 *dr*, colorless oil; ^{1}H NMR (500 MHz, $CDCl_3$) (δ, ppm) 7.88 (d, *J* = 8.6 Hz, 2H), 7.80 – 7.73 (comp, 2H), 7.64 (s, 1H), 7.58 – 7.55 (comp, 2H), 7.44 – 7.37 (comp, 3H), 7.06 (t, *J* = 8.5 Hz, 2H), 6.93 (d, *J* = 8.6 Hz, 2H), 4.12 (d, *J* = 17.5 Hz, 1H), 4.09 – 4.02 (m, 1H), 3.98 – 3.91 (m, 1H), 3.88 (s, 3H), 2.94 (s, 1H), 2.59 (d, *J* = 17.5 Hz, 1H), 1.01 (t, *J* = 7.1 Hz, 3H), 0.80 (s, 9H), 0.03 (s, 3H), 0.01 (s, 3H); ^{13}C NMR (125 MHz, $CDCl_3$) (δ, ppm) 169.7, 168.9, 162.3 (d, *J* = 246.6 Hz), 161.8, 150.8, 150.7, 136.0, 132.5, 131.9 (d, *J* = 8.1 Hz), 130.0, 128.7, 128.0 (d, *J* = 2.9 Hz), 126.8, 126.1, 115.5 (d, *J* = 21.5 Hz), 112.9, 62.0, 55.5, 44.6, 43.1, 35.1, 27.2, 26.2, 18.3, 13.9, -5.18, -5.22; HRMS (ESI Q-TOF) m/z: $[M+H]^+$ Calcd for $C_{35}H_{41}FN_3O_4Si$ 630.2794,

found 630.2798.

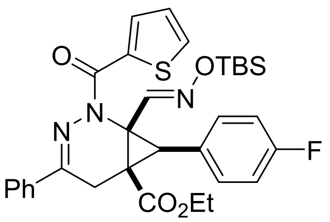

Ethyl 1-(*E*)-(*tert*-Butyldimethylsilyloxy)iminomethyl-7-(4-fluorophenyl)-4-phenyl-2-(thiophene-2-carbonyl)-2,3-diazabicyclo[4.1.0]hept-3-ene-6-carboxylate (3u). 52.6 mg, 87% yield, >20:1 *dr*, colorless oil; ^{1}H NMR (500 MHz, $CDCl_3$) (δ, ppm) 8.16 (d, J = 3.7 Hz, 1H), 8.03 – 7.95 (comp, 2H), 7.62 (d, J = 4.9 Hz, 1H), 7.57 – 7.55 (comp, 3H), 7.53 – 7.48 (comp, 3H), 7.12 (t, J = 4.3 Hz, 1H), 7.06 (t, J = 8.4 Hz, 2H), 4.31 (d, J = 16.9 Hz, 1H), 4.13 – 4.01 (m, 1H), 3.99 – 3.85 (m, 1H), 3.00 (s, 1H), 2.42 (d, J = 16.9 Hz, 1H), 0.98 (t, J = 7.1 Hz, 3H), 0.77 (s, 9H), -0.02 (s, 3H), -0.03 (s, 3H); ^{13}C NMR (125 MHz, $CDCl_3$) (δ, ppm) 168.7, 162.4 (d, J = 246.8 Hz), 162.2, 155.7, 149.5, 135.7, 135.6, 134.6, 133.8, 131.9 (d, J = 8.0 Hz), 130.5, 128.9, 127.8 (d, J = 3.0 Hz), 126.8, 126.4, 115.5 (d, J = 21.6 Hz), 62.0, 45.3, 43.9, 35.2, 28.8, 26.1, 18.3, 13.9, -5.36, -5.42; HRMS (ESI Q-TOF) m/z: $[M+H]^+$ Calcd for $C_{32}H_{37}FN_3O_4SSi$ 606.2253, found 606.2255.

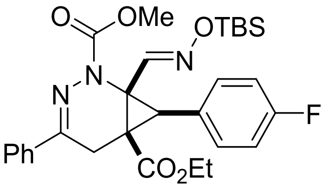

6-Ethyl 2-Methyl 1-(*E*)-(*tert*-Butyldimethylsilyl)oxy)imino)methyl)-7-(4-fluoro-phenyl)-4-phenyl-2,3-diazabicyclo[4.1.0]hept-3-ene-2,6-dicarboxylate (3v). 47.6 mg, 86% yield, >20:1 *dr*, colorless oil; ^{1}H NMR (500 MHz, $CDCl_3$) (δ, ppm) 7.85 – 7.83 (comp, 2H), 7.45 – 7.36 (comp, 6H), 7.04 (t, J = 8.6 Hz, 2H), 4.21 (d, J = 16.1 Hz, 1H), 4.07 – 3.98 (m, 1H), 3.96 – 3.88 (comp, 4H), 3.02 (s, 1H), 2.25 (d, J = 16.1 Hz, 1H), 0.97 (t, J = 7.1 Hz, 3H), 0.89 (s, 9H), 0.15 (s, 3H), 0.12 (s, 3H); ^{13}C NMR (125 MHz, $CDCl_3$) (δ, ppm) 168.4, 162.3 (d, J = 246.8 Hz), 159.4, 155.2, 149.5, 135.9, 131.7 (d, J = 8.0 Hz), 130.3, 128.7, 127.8 (d, J = 3.0 Hz), 126.6, 115.5 (d, J = 21.5 Hz), 62.0, 53.8, 44.1, 37.2, 30.1, 26.2, 18.4, 13.8, -5.2; HRMS (ESI Q-TOF) m/z: $[M+H]^+$ Calcd for $C_{29}H_{37}FN_3O_5Si$ 554.2481, found 554.2487.

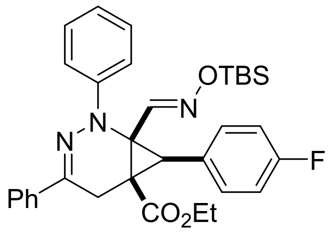

Ethyl 1-(*E*)-(*tert*-Butyldimethylsilyloxy)iminomethyl-7-(4-fluorophenyl)-2,4-diphenyl-2,3-diazabicyclo[4.1.0]hept-3-ene-6-carboxylate (3w). 52.1 mg, 87% yield, >20:1 *dr*, colorless oil; ^{1}H NMR (500 MHz, $CDCl_3$) (δ, ppm) 7.84 (d, J = 7.2 Hz, 2H), 7.74 – 7.72 (comp, 2H), 7.65 (s, 1H), 7.57 – 7.55 (comp, 2H), 7.48 (t, J = 7.3 Hz, 1H), 7.45 – 7.34 (comp, 5H), 7.07 (t, J = 8.6 Hz, 2H), 4.14 (d, J = 17.6 Hz, 1H), 4.11 – 4.05 (m, 1H), 3.98 – 3.92 (m, 1H), 2.95 (s, 1H), 2.58 (d, J = 17.6 Hz, 1H), 1.01 (t, J = 7.1 Hz, 3H), 0.81 (s, 9H), 0.05 (s, 3H), 0.03 (s, 3H); ^{13}C NMR (125 MHz, $CDCl_3$) (δ, ppm) 170.2, 168.8, 162.4 (d, J = 246.7 Hz), 151.2, 150.6, 135.8, 134.8, 131.9 (d, J = 8.1 Hz), 130.9, 130.2, 128.7, 127.9 (d, J = 3.0 Hz), 127.6, 126.1, 115.5 (d, J = 21.5 Hz), 62.0, 44.4, 43.3, 35.0, 27.3, 26.2, 18.3, 13.9, -5.16, -5.20; HRMS (ESI Q-TOF) m/z: $[M+H]^+$ Calcd for $C_{33}H_{39}FN_3O_3Si$ 572.2739, found 572.2742.

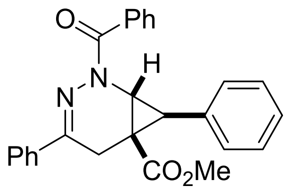

Methyl 2-Benzoyl-4,7-diphenyl-2,3-diazabicyclo[4.1.0]hept-3-ene-6-carboxylate (6a). 17.6 mg, 43% yield, >20:1 *dr*, colorless oil; ^{1}H NMR (500 MHz, $CDCl_3$) (δ, ppm) 7.83 (d, J = 7.9 Hz, 2H), 7.70 – 7.63 (comp, 2H), 7.51 – 7.43 (comp, 5H), 7.36 – 7.32 (comp, 6H), 5.04 (d, J = 5.4 Hz, 1H), 3.41 (s, 3H), 3.41 – 3.27 (comp, 2H), 2.72 (d, J = 5.4 Hz, 1H); ^{13}C NMR (125 MHz, $CDCl_3$) (δ, ppm) 171.6, 170.1, 143.5, 136.6, 134.6, 134.3, 130.9, 130.3, 129.7, 129.5, 128.9, 128.7, 128.5, 127.6, 125.6, 52.1, 36.8, 36.4, 29.1, 23.6; HRMS (ESI Q-TOF) m/z: $[M+H]^+$ Calcd for $C_{26}H_{23}N_2O_3$ 411.1703, found 411.1707.

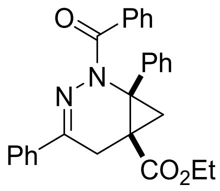

Ethyl 2-Benzoyl-1,4-diphenyl-2,3-diazabicyclo[4.1.0]hept-3-ene-6-carboxylate

(6b). 33.5 mg, 79% yield, >20:1 *dr*, colorless oil; ^{1}H NMR (300 MHz, $CDCl_3$) (δ, ppm) 7.87 – 7.76 (comp, 2H), 7.72 – 7.60 (comp, 2H), 7.47 – 7.31 (comp, 8H), 7.27 – 7.16 (comp, 3H), 4.11 (d, *J* = 17.2 Hz, 1H), 3.92 – 3.46 (comp, 2H), 2.92 (d, *J* = 6.3 Hz, 1H), 2.28 (d, *J* = 17.2 Hz, 1H), 1.47 (d, *J* = 6.3 Hz, 1H), 0.74 (t, *J* = 7.1 Hz, 3H); ^{13}C NMR (125 MHz, $CDCl_3$) (δ, ppm) 170.2, 169.6, 155.6, 136.2, 135.8, 135.1, 130.7, 130.3, 129.9, 128.8, 128.7, 127.9, 127.8, 127.5, 126.4, 61.6, 46.8, 31.9, 27.4, 23.7, 13.6; HRMS (ESI Q-TOF) m/z: $[M+H]^+$ Calcd for $C_{27}H_{25}N_2O_3$ 425.1860, found 425.1862.

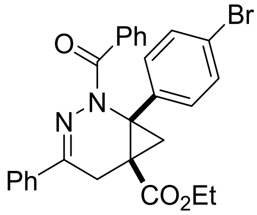

Ethyl 2-Benzoyl-1-(4-bromophenyl)-4-phenyl-2,3-diazabicyclo[4.1.0]hept-3-ene-6-carboxylate (6c). 39.2 mg, 78% yield, >20:1 *dr*, colorless oil; ^{1}H NMR (300 MHz, $CDCl_3$) (δ, ppm) 7.87 – 7.78 (comp, 2H), 7.69 – 7.60 (comp, 2H), 7.45 – 7.32 (comp, 8H), 7.25 – 7.18 (comp, 2H), 4.11 (d, *J* = 17.2 Hz, 1H), 3.84 – 3.54 (comp, 2H), 2.92 (d, *J* = 6.3 Hz, 1H), 2.28 (d, *J* = 17.2 Hz, 1H), 1.47 (d, *J* = 6.3 Hz, 1H), 0.74 (t, *J* = 7.1 Hz, 3H); ^{13}C NMR (125 MHz, $CDCl_3$) (δ, ppm) 170.2, 169.6, 155.5, 136.2, 135.8, 135.2, 130.7, 130.3, 129.9, 128.8, 128.7, 127.9, 127.8, 127.5, 126.4, 61.6, 46.8, 31.9, 27.4, 23.7, 13.6; HRMS (ESI Q-TOF) m/z: $[M+H]^+$ Calcd for $C_{27}H_{24}BrN_2O_3$ 503.0965, found 503.0966.

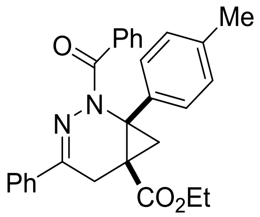

Ethyl 2-Benzoyl-4-phenyl-1-(*p*-tolyl)-2,3-diazabicyclo[4.1.0]hept-3-ene-6-carboxylate (6d). 38.6 mg, 88% yield, >20:1 *dr*, colorless oil; ^{1}H NMR (500 MHz, $CDCl_3$) (δ, ppm) 7.95 – 7.76 (comp, 2H), 7.66 (d, *J* = 8.0 Hz, 2H), 7.46 – 7.38 (comp, 4H), 7.36 (t, *J* = 7.6 Hz, 2H), 7.30 (d, *J* = 7.8 Hz, 2H), 7.04 (d, *J* = 7.8 Hz, 2H), 4.09 (d, *J* = 17.0 Hz, 1H), 3.84 – 3.71 (m, 1H), 3.71 – 3.65 (m, 1H), 2.89 (d, *J* = 6.3 Hz, 1H), 2.28 (d, *J* = 17.0 Hz, 1H), 2.27 (s, 3H), 1.45 (d, *J* = 6.3 Hz, 1H), 0.78 (t, *J* = 7.1 Hz, 3H); ^{13}C NMR (125 MHz, $CDCl_3$) (δ, ppm) 170.2, 169.6, 155.3, 137.4, 135.8, 135.2,

133.2, 130.6, 130.2, 129.9, 128.8, 128.6, 128.5, 127.5, 126.4, 61.5, 46.7, 31.8, 27.4, 23.7, 21.3, 13.6; HRMS (ESI Q-TOF) m/z: $[M+H]^+$ Calcd for $C_{28}H_{27}N_2O_3$ 439.2016, found 439.2018.

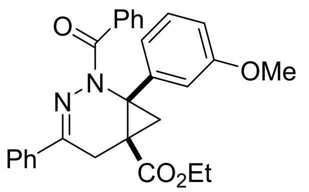

Ethyl 2-Benzoyl-1-(3-methoxyphenyl)-4-phenyl-2,3-diazabicyclo[4.1.0]hept-3-ene-6-carboxylate (6e). 37.2 mg, 82% yield, >20:1 *dr*, colorless oil; ^1H NMR (500 MHz, $CDCl_3$) (δ, ppm) 7.85 – 7.72 (comp, 3H), 7.66 (d, J = 7.4 Hz, 2H), 7.40 – 7.37 (comp, 4H), 7.33 (t, J = 7.5 Hz, 2H), 7.20 – 7.17 (m, 1H), 6.96 (t, J = 7.7 Hz, 1H), 6.70 (d, J = 8.1 Hz, 1H), 4.21 (d, J = 17.8 Hz, 1H), 3.78 – 3.71 (m, 1H), 3.66 – 3.60 (m, 1H), 3.55 (s, 3H), 2.76 (d, J = 5.8 Hz, 1H), 2.37 (d, J = 17.8 Hz, 1H), 1.42 (d, J = 5.8 Hz, 1H), 0.73 (t, J = 7.1 Hz, 3H); ^{13}C NMR (125 MHz, $CDCl_3$) (δ, ppm) 170.7, 169.5, 158.0, 150.2, 136.7, 135.6, 134.1, 130.4, 130.1, 129.6, 129.1, 128.7, 127.4, 126.1, 124.6, 119.9, 109.9, 61.3, 55.4, 42.9, 28.8, 25.72, 25.68, 13.6; HRMS (ESI Q-TOF) m/z: $[M+H]^+$ Calcd for $C_{28}H_{27}N_2O_4$ 455.1965, found 455.1967.

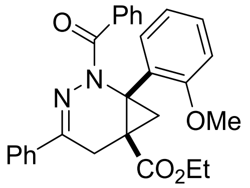

Ethyl 2-Benzoyl-1-(2-methoxyphenyl)-4-phenyl-2,3-diazabicyclo[4.1.0]hept-3-ene-6-carboxylate (6f). 35.4 mg, 78% yield, >20:1 *dr*, colorless oil; ^1H NMR (500 MHz, $CDCl_3$) (δ, ppm) 7.87 – 7.74 (comp, 3H), 7.68 (d, J = 7.6 Hz, 2H), 7.43 – 7.40 (comp, 4H), 7.35 (t, J = 7.4 Hz, 2H), 7.21 (t, J = 7.7 Hz, 1H), 6.98 (t, J = 7.6 Hz, 1H), 6.72 (d, J = 8.1 Hz, 1H), 4.23 (d, J = 17.8 Hz, 1H), 3.80 – 3.74 (m, 1H), 3.69 – 3.62 (m, 1H), 3.58 (s, 3H), 2.78 (d, J = 5.7 Hz, 1H), 2.39 (d, J = 17.8 Hz, 1H), 1.45 (d, J = 5.7 Hz, 1H), 0.76 (t, J = 7.1 Hz, 3H); ^{13}C NMR (125 MHz, $CDCl_3$) (δ, ppm) 170.7, 169.5, 158.0, 150.2, 136.7, 135.6, 134.1, 130.4, 130.1, 129.6, 129.1, 128.7, 127.4, 126.1, 124.6, 119.9, 109.9, 61.3, 55.4, 42.9, 28.8, 25.8, 25.7, 13.6; HRMS (ESI Q-TOF) m/z: $[M+H]^+$ Calcd for $C_{28}H_{27}N_2O_4$ 455.1965, found 455.1966.

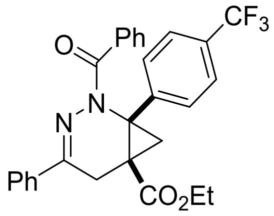

Ethyl 2-Benzoyl-4-phenyl-1-(4-(trifluoromethyl)phenyl)-2,3-diazabicyclo[4.1.0]-hept-3-ene-6-carboxylate (6g). 40.8 mg, 83% yield, >20:1 *dr*, colorless oil; ^{1}H NMR (500 MHz, $CDCl_3$) (δ, ppm) 7.82 (d, *J* = 7.1 Hz, 2H), 7.65 (d, *J* = 7.6 Hz, 2H), 7.55 – 7.50 (comp, 4H), 7.47 – 7.42 (comp, 4H), 7.39 – 7.36 (comp, 2H), 4.13 (d, *J* = 17.2 Hz, 1H), 3.78 – 3.67 (comp, 2H), 2.94 (d, *J* = 6.4 Hz, 1H), 2.32 (d, *J* = 17.2 Hz, 1H), 1.53 (d, *J* = 6.4 Hz, 1H), 0.75 (t, *J* = 7.0 Hz, 3H); ^{13}C NMR (125 MHz, $CDCl_3$) (δ, ppm) 169.8, 169.7, 155.6, 140.4, 135.5, 134.7, 131.0, 130.5, 129.93, 129.91 (q, *J* = 32.5 Hz), 129.0, 128.9, 127.7, 126.4, 125.0 (q, *J* = 3.5 Hz), 124.1 (q, *J* = 272.1 Hz), 61.8, 46.3, 32.1, 27.2, 23.8, 13.6; HRMS (ESI Q-TOF) m/z: $[M+Na]^+$ Calcd for $C_{28}H_{24}F_3N_2O_3$ 493.1734, found 493.1732.

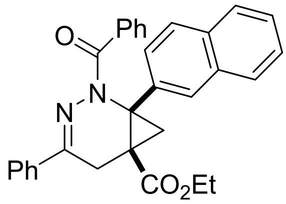

Ethyl 2-Benzoyl-1-(naphthalen-2-yl)-4-phenyl-2,3-diazabicyclo[4.1.0]hept-3-ene-6-carboxylate (6h). 40.3 mg, 85% yield, >20:1 *dr*, colorless oil; ^{1}H NMR (500 MHz, $CDCl_3$) (δ, ppm) 7.90 (s, 1H), 7.88 – 7.86 (comp, 2H), 7.81 – 7.68 (comp, 3H), 7.65 (d, *J* = 7.7 Hz, 2H), 7.51 (d, *J* = 8.3 Hz, 1H), 7.45 – 7.41 (comp, 6H), 7.37 – 7.34 (comp, 2H), 4.16 (d, *J* = 17.2 Hz, 1H), 3.74 – 3.60 (m, 1H), 3.60 – 3.47 (m, 1H), 3.06 (d, *J* = 6.2 Hz, 1H), 2.34 (d, *J* = 17.2 Hz, 1H), 1.56 (d, *J* = 6.2 Hz, 1H), 0.57 (t, *J* = 7.0 Hz, 3H); ^{13}C NMR (125 MHz, $CDCl_3$) (δ, ppm) 170.1, 169.6, 155.4, 135.8, 135.1, 133.6, 133.0, 130.7, 130.3, 129.9, 128.8, 128.3, 128.0, 127.6, 127.5, 126.5, 126.3, 126.1, 126.0, 61.5, 47.0, 32.0, 27.4, 23.9, 13.5; HRMS (ESI Q-TOF) m/z: $[M+H]^+$ Calcd for $C_{31}H_{27}N_2O_3$ 475.2016, found 475.2017.

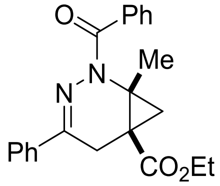

Ethyl 2-Benzoyl-1-methyl-4-phenyl-2,3-diazabicyclo[4.1.0]hept-3-ene-6-carboxylate (6i). 26.1 mg, 72% yield, 2:1 *dr*, colorless oil; composite NMR signals of two diastereoisomers: ^{1}H NMR (500 MHz, $CDCl_3$) (δ, ppm) 7.81 – 7.71 (comp, 3H), 7.67 (d, J = 7.3 Hz, 2H), 7.62 – 7.59 (m, 1H), 7.49 – 7.36 (comp, 8H), 7.36 – 7.30 (m, 1H), 4.34 – 4.14 (comp, 3.5H), 3.99 (d, J = 16.8 Hz, 1H), 3.00 – 2.81 (m, 0.5H), 2.76 – 2.60 (m, 1H), 2.11 (d, J = 5.9 Hz, 1H), 2.01 – 1.95 (comp, 1.5H), 1.65 (s, 3H), 1.63 (s, 1.5H), 1.31 (t, J = 7.1 Hz, 1.5H), 1.27 (t, J = 7.1 Hz, 3H); ^{13}C NMR (125 MHz, $CDCl_3$) (δ, ppm) 171.3, 171.0, 169.6, 160.1, 159.0, 146.5, 136.7, 135.6, 135.4, 133.9, 131.0, 130.7, 130.6, 130.4, 130.0, 129.7, 128.8, 128.7, 127.6, 126.5, 125.7, 125.6, 62.3, 61.9, 61.2, 41.9, 30.4, 29.9, 28.3, 27.0, 26.5, 19.5, 18.7, 15.7, 14.4, 14.1; HRMS (ESI Q-TOF) m/z: $[M+H]^+$ Calcd for $C_{22}H_{23}N_2O_3$ 363.1703, found 363.1706.

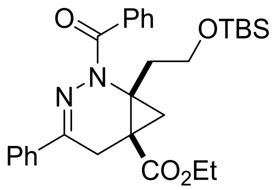

Ethyl 2-Benzoyl-1-2-(*tert*-butyldimethylsilyloxy)ethyl-4-phenyl-2,3-diazabicyclo-[4.1.0]hept-3-ene-6-carboxylate (6j). 40.5 mg, 80% yield, 4:1 *dr*, colorless oil; composite NMR signals of two diastereoisomers: ^{1}H NMR (500 MHz, $CDCl_3$) (δ, ppm) 7.81 – 7.77 (comp, 2.4H), 7.71 (d, J = 7.5 Hz, 2H), 7.64 (d, J = 6.8 Hz, 0.5H), 7.50 – 7.45 (comp, 1.6H), 7.44 – 7.34 (comp, 6H), 4.35 – 4.12 (comp, 4H), 3.81 (t, J = 6.0 Hz, 0.6H), 3.66 – 3.56 (m, 1H), 3.53 – 3.48 (m, 1H), 3.36 (d, J = 18.1 Hz, 0.3H), 3.17 – 3.03 (m, 1H), 2.58 (d, J = 18.1 Hz, 0.3H), 2.16 – 2.12 (comp, 1.3H), 2.05 – 1.91 (comp, 2H), 1.87 – 1.84 (comp, 0.6H), 1.32 (t, J = 7.1 Hz, 3H), 1.27 (t, J = 7.1 Hz, 0.7H), 0.92 (s, 2.3H), 0.75 (s, 9H), 0.08 (s, 0.7H), 0.06 (s, 0.7H), -0.11 (s, 3H), -0.16 (s, 3H); ^{13}C NMR (125 MHz, $CDCl_3$) (δ, ppm) 171.5, 171.3, 169.2, 168.6, 158.4, 136.3, 135.7, 135.5, 134.9, 133.6, 130.7, 130.5, 130.3, 130.0, 129.8, 129.7, 128.62, 128.60, 127.6, 126.6, 125.8, 61.87, 61.85, 61.3, 59.9, 43.7, 36.3, 31.3, 29.9, 29.7, 28.7, 27.9, 26.9, 26.1, 25.3, 18.5, 18.4, 14.4, 14.3, -5.25, -5.34, -5.5; HRMS (ESI Q-TOF) m/z: $[M+H]^+$ Calcd for $C_{29}H_{39}N_2O_4Si$ 507.2674, found 507.2675.

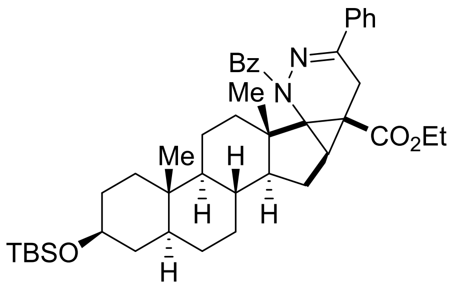

Ethyl (2a*S*,4*S*,6a*S*,6b*S*,8a*S*,13a*S*,13b*R*)-9-Benzoyl-4-(*tert*-butyldimethylsilyloxy)-6a,8a-dimethyl-11-phenyl-1,2,2a,3,4,5,6,6a,6b,7,8,8a,9,12,12b,13,13a,13b-octadecahydro-12a*H*-naphtho[2'',1'':4',5']indeno[1',2':1,3]cyclopropa[1,2-*c*]pyridazine-12a-carboxylate (6k). 48.2 mg, 68% yield, >20:1 *dr*, colorless oil; ^{1}H NMR (500 MHz, $CDCl_3$) (δ, ppm) 7.68 (d, *J* = 7.8 Hz, 2H), 7.63 (d, *J* = 6.9 Hz, 2H), 7.49 – 7.40 (comp, 2H), 7.39 – 7.37 (comp, 2H), 7.36 – 7.33 (comp, 2H), 4.23 (q, *J* = 7.1 Hz, 2H), 3.62 – 3.46 (m, 1H), 3.16 (d, *J* = 18.7 Hz, 1H), 2.63 – 2.44 (comp, 2H), 1.79 – 1.48 (comp, 9H), 1.48 – 1.38 (comp, 3H), 1.35 – 1.20 (comp, 9H), 1.05 (s, 3H), 0.97 – 0.92 (m, 1H), 0.88 (s, 9H), 0.83 (s, 3H), 0.60 – 0.55 (m, 1H), 0.05 (s, 6H); ^{13}C NMR (125 MHz, $CDCl_3$) (δ, ppm) 171.0, 169.5, 149.5, 136.3, 135.7, 130.4, 129.8, 129.7, 128.6, 127.5, 125.7, 72.2, 61.3, 55.2, 47.3, 46.5, 45.6, 45.4, 41.0, 38.7, 37.2, 36.3, 35.9, 35.1, 32.1, 32.0, 28.8, 27.3, 26.1, 22.4, 21.0, 18.4, 18.0, 14.5, 12.5, -4.4; HRMS (ESI Q-TOF) m/z: $[M+H]^+$ Calcd for $C_{44}H_{61}N_2O_4Si$ 709.4395, found 709.4396.

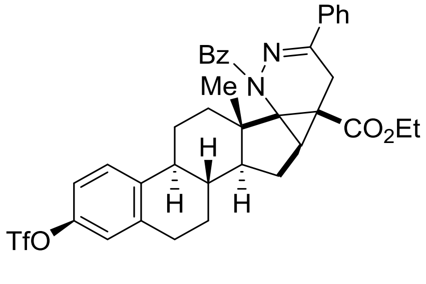

Ethyl (6b*S*,8a*S*,13a*S*,13b*R*)-9-Benzoyl-8a-methyl-11-phenyl-4-(trifluoromethyl-sulfonyloxy)-1,2,6b,7,8,8a,9,12,12b,13,13a,13b-dodecahydro-12a*H*-naphtho[2'',1'':4',5']indeno[1',2':1,3]cyclopropa[1,2-*c*]pyridazine-12a-carboxylate (6l). 44.5 mg, 63% yield, >20:1 *dr*, colorless oil; ^{1}H NMR (500 MHz, $CDCl_3$) (δ, ppm) 7.70 (d, *J* = 7.5 Hz, 2H), 7.65 (d, *J* = 7.1 Hz, 2H), 7.48 – 7.42 (m, 1H), 7.40 – 7.35 (comp, 4H), 7.28 (d, *J* = 8.7 Hz, 1H), 7.26 (s, 1H), 7.02 (d, *J* = 8.7 Hz, 1H), 6.98 (s, 1H), 4.27 – 4.18 (comp, 2H), 3.23 (d, *J* = 18.7 Hz, 1H), 2.95 – 2.92 (comp, 2H), 2.69 (dd, *J* = 12.0, 6.0 Hz, 1H), 2.58 (d, *J* = 18.7 Hz, 1H), 2.33 (d, *J* = 11.3 Hz, 1H), 2.21 – 2.17 (m, 1H), 2.00 (d, *J* = 11.8 Hz, 1H), 1.90 (d, *J* = 11.5 Hz, 1H), 1.84 – 1.78

(m, 1H), 1.76 – 1.66 (comp, 2H), 1.65 – 1.56 (comp, 2H), 1.56 – 1.44 (comp, 2H), 1.29 (t, J = 7.0 Hz, 3H), 1.13 (s, 3H); ^{13}C NMR (125 MHz, $CDCl_3$) (δ, ppm) 171.0, 169.4, 149.4, 147.7, 140.7, 139.6, 136.2, 135.6, 130.5, 129.9, 128.7, 127.5, 126.9, 125.7, 121.4, 118.3, 61.4, 46.7, 46.6, 45.6, 44.6, 41.1, 37.6, 36.4, 32.1, 29.5, 27.5, 27.1, 26.0, 22.4, 18.0, 14.4; HRMS (ESI Q-TOF) m/z: $[M+H]^+$ Calcd for $C_{38}H_{38}F_3N_2O_6S$ 707.2397, found 707.2400.

General Procedure for [4+2]-Cycloaddition with Cyclopentadiene and Furan.

To a 10-mL oven-dried vial with a magnetic stirring bar, diazo compound **1** (0.1 mmol) in DCM (1.0 mL) was added over 3.0 h *via* a syringe pump to a solution of cyclopentadiene/furan (0.2 mmol, 2.0 equiv.) in the DCM (1.0 mL) at room temperature with irradiation by 440 nm blue LED. When the reaction was complete (monitored by TLC), the reaction mixture was purified by flash column chromatography on silica gel without additional treatment (hexanes:EtOAc = 20:1 to 15:1) to give the pure cycloaddition product.

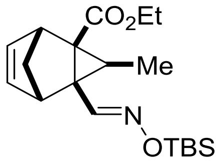

Ethyl (*E*)-4-(*tert*-Butyldimethylsilyloxy)iminomethyl-3-methyltricyclo[3.2.1.0^{2,4}]-oct-6-ene-2-carboxylate (8a). 28.3 mg, 81% yield, >20:1 *dr*, colorless oil; ^{1}H NMR (500 MHz, $CDCl_3$) (δ, ppm) 7.87 (s, 1H), 6.06 – 6.01 (m, 1H), 5.99 – 5.97 (m, 1H), 4.17 (q, J = 7.1 Hz, 2H), 3.48 – 3.46 (comp, 2H), 1.87 (d, J = 7.4 Hz, 1H), 1.77 (q, J = 6.7 Hz, 1H), 1.70 (d, J = 7.4 Hz, 1H), 1.30 (d, J = 6.7 Hz, 3H), 1.26 (t, J = 7.1 Hz, 3H), 0.94 (s, 9H), 0.17 (s, 3H), 0.15 (s, 3H); ^{13}C NMR (125 MHz, $CDCl_3$) (δ, ppm) 172.1, 154.2, 134.5, 134.3, 61.3, 60.7, 48.2, 46.9, 40.0, 39.5, 37.9, 26.4, 18.5, 14.5, 10.2, -5.1; HRMS (ESI Q-TOF) m/z: $[M+H]^+$ Calcd for $C_{19}H_{32}NO_3Si$ 350.2146, found 350.2147.

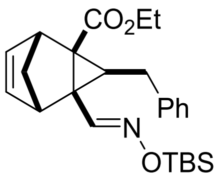

Ethyl (*E*)-3-Benzyl-4-((*tert*-butyldimethylsilyloxy)iminomethyl)tricyclo[3.2.1.0^{2,4}]-oct-6-ene-2-carboxylate (8b). 34.9 mg, 82% yield, >20:1 *dr*, colorless oil; ^{1}H NMR (500 MHz, $CDCl_3$) (δ, ppm) 8.00 (s, 1H), 7.24 (d, *J* = 7.4 Hz, 2H), 7.17 (t, *J* = 7.2 Hz, 1H), 7.10 (d, *J* = 7.4 Hz, 2H), 6.06 – 6.02 (m, 1H), 6.01 – 5.99 (m, 1H), 4.18 (q, *J* = 7.0 Hz, 2H), 3.54 – 3.53 (comp, 2H), 3.20 (dd, *J* = 15.4, 7.4 Hz, 1H), 3.09 (dd, *J* = 15.4, 7.4 Hz, 1H), 1.99 (t, *J* = 7.3 Hz, 1H), 1.92 (d, *J* = 7.3 Hz, 1H), 1.74 (d, *J* = 7.3 Hz, 1H), 1.25 (t, *J* = 7.0 Hz, 3H), 0.94 (s, 9H), 0.18 (s, 3H), 0.16 (s, 3H); ^{13}C NMR (125 MHz, $CDCl_3$) (δ, ppm) 172.0, 154.0, 140.9, 134.7, 134.4, 128.5, 128.3, 126.1, 61.4, 60.9, 48.2, 47.1, 43.9, 39.9, 39.5, 30.4, 26.4, 18.5, 14.4, -5.1; HRMS (ESI Q-TOF) m/z: $[M+H]^+$ Calcd for $C_{25}H_{36}NO_3Si$ 426.2459, found 426.2455.

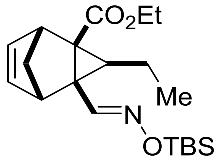

Ethyl (*E*)-4-(*tert*-Butyldimethylsilyloxy)iminomethyl-3-ethyltricyclo[3.2.1.0^{2,4}]-oct-6-ene-2-carboxylate (8c). 28.7 mg, 79% yield, >20:1 *dr*, colorless oil; ^{1}H NMR (500 MHz, $CDCl_3$) (δ, ppm) 7.85 (s, 1H), 6.04 – 6.01 (m, 1H), 6.01 – 5.96 (m, 1H), 4.16 (q, *J* = 7.1 Hz, 2H), 3.47 – 3.46 (comp, 2H), 1.87 (d, *J* = 7.3 Hz, 1H), 1.83 – 1.72 (m, 2H), 1.70 (d, *J* = 7.5 Hz, 1H), 1.65 (t, *J* = 7.5 Hz, 1H), 1.26 (t, *J* = 7.1 Hz, 3H), 0.94 (s, 9H), 0.86 (t, *J* = 7.3 Hz, 3H), 0.17 (s, 3H), 0.15 (s, 3H); ^{13}C NMR (125 MHz, $CDCl_3$) (δ, ppm) 172.2, 154.4, 134.6, 134.3, 61.3, 60.7, 48.3, 46.9, 45.6, 39.6, 39.4, 26.4, 18.5, 18.1, 14.5, 13.8, -5.1; HRMS (ESI Q-TOF) m/z: $[M+H]^+$ Calcd for $C_{20}H_{34}NO_3Si$ 364.2302, found 364.2296.

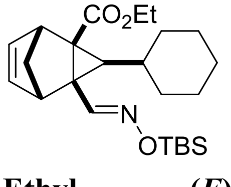

Ethyl (*E*)-4-(*tert*-Butyldimethylsilyloxy)iminomethyl-3-cyclohexyltricyclo-[3.2.1.0^{2,4}]oct-6-ene-2-carboxylate (8d). 33.4 mg, 80% yield, >20:1 *dr*, colorless oil; ^{1}H NMR (500 MHz, $CDCl_3$) 7.84 (s, 1H), 6.03 – 6.01 (m, 1H), 5.98 – 5.93 (m, 1H), 4.26 – 4.04 (comp, 2H), 3.48 – 3.47 (comp, 2H), 1.99 – 1.92 (m, 1H), 1.85 (d, *J* = 7.4 Hz, 1H), 1.70 (d, *J* = 7.4 Hz, 1H), 1.66 – 1.62 (comp, 3H), 1.57 – 1.54 (m, 1H), 1.49 – 1.47 (comp, 2H), 1.27 – 1.24 (comp, 4H), 1.22 – 1.06 (comp, 3H), 0.93 (s, 9H), 0.91 – 0.85 (m, 1H), 0.17 (s, 3H), 0.15 (s, 3H); ^{13}C NMR (125 MHz, $CDCl_3$) (δ, ppm) 172.3,

154.6, 134.6, 134.3, 61.3, 60.7, 50.5, 48.3, 47.0, 39.2, 39.1, 33.1, 33.0, 32.9, 26.5, 26.4, 26.1, 18.6, 14.5, -5.10, -5.12; HRMS (ESI Q-TOF) m/z: $[M+H]^+$ Calcd for $C_{24}H_{40}NO_3Si$ 418.2772, found 418.2765.

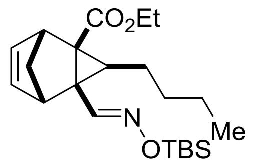

Ethyl (*E*)-3-Butyl-4-(*tert*-butyldimethylsilyloxy)iminomethyltricyclo[3.2.1.0^{2,4}]-oct-6-ene-2-carboxylate (8e). 33.6 mg, 86% yield, >20:1 *dr*, colorless oil; ^{1}H NMR (500 MHz, $CDCl_3$) (δ, ppm) 7.86 (s, 1H), 6.04 – 6.03 (m, 1H), 5.99 – 5.98 (m, 1H), 4.16 (q, J = 7.1 Hz, 2H), 3.48 – 3.47 (comp, 2H), 1.87 (d, J = 7.3 Hz, 1H), 1.80 – 1.76 (m, 1H), 1.73 – 1.64 (comp, 3H), 1.28 – 1.19 (comp, 7H), 0.94 (s, 9H), 0.85 (t, J = 6.9 Hz, 3H), 0.17 (s, 3H), 0.15 (s, 3H); ^{13}C NMR (125 MHz, $CDCl_3$) (δ, ppm) 172.2, 154.5, 134.5, 134.3, 61.3, 60.7, 48.2, 46.9, 44.0, 39.6, 39.3, 31.7, 26.4, 24.4, 22.6, 18.5, 14.5, 14.2, -5.1; HRMS (ESI Q-TOF) m/z: $[M+H]^+$ Calcd for $C_{22}H_{38}NO_3Si$ 392.2615, found 392.2610.

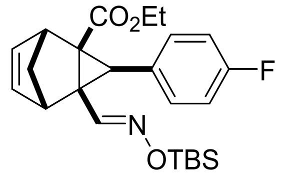

Ethyl (*E*)-4-(*tert*-Butyldimethylsilyloxy)iminomethyl-3-(4-fluorophenyl)tricyclo-[3.2.1.0^{2,4}]oct-6-ene-2-carboxylate (8f). 39.5 mg, 92% yield, >20:1 *dr*, white solid, mp: 105.0-106.0 °C; ^{1}H NMR (500 MHz, $CDCl_3$) (δ, ppm) 7.74 (s, 1H), 6.98 – 6.92 (comp, 4H), 6.19 (s, 1H), 6.11 (s, 1H), 4.19 – 3.91 (comp, 2H), 3.71 (s, 1H), 3.67 (s, 1H), 2.95 (s, 1H), 1.92 (d, J = 7.5 Hz, 1H), 1.78 (d, J = 7.5 Hz, 1H), 1.03 (t, J = 7.1 Hz, 3H), 0.93 (s, 9H), 0.18 (s, 3H), 0.16 (s, 3H); ^{13}C NMR (125 MHz, $CDCl_3$) (δ, ppm) 171.3, 161.6 (d, J = 245.1 Hz), 154.6, 135.0, 134.8, 131.2 (d, J = 3.1 Hz), 131.0 (d, J = 7.9 Hz), 115.1 (d, J = 21.4 Hz), 61.4, 60.9, 48.2, 47.0, 46.0, 40.1, 39.3, 26.3, 18.5, 14.1, -5.11, -5.13; HRMS (ESI Q-TOF) m/z: $[M+H]^+$ Calcd for $C_{24}H_{33}FNO_3Si$ 430.2208, found 430.2212.

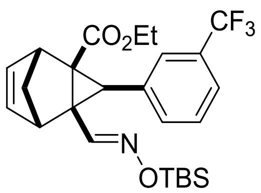

Ethyl (*E*)-4-(*tert*-Butyldimethylsilyloxy)iminomethyl-3-(3-(trifluoromethyl)-phenyl)tricyclo[3.2.1.0^{2,4}]oct-6-ene-2-carboxylate (8g). 42.2 mg, 88% yield, >20:1 *dr*, colorless oil; ^{1}H NMR (500 MHz, $CDCl_3$) (δ, ppm) 7.70 (s, 1H), 7.45 (d, *J* = 7.7 Hz, 1H), 7.37 (t, *J* = 7.7 Hz, 1H), 7.27 (s, 1H), 7.21 (d, *J* = 7.6 Hz, 1H), 6.27 – 6.18 (m, 1H), 6.18 – 6.08 (m, 1H), 4.11 – 3.92 (comp, 2H), 3.74 (s, 1H), 3.70 (s, 1H), 3.02 (s, 1H), 1.94 (d, *J* = 7.5 Hz, 1H), 1.81 (d, *J* = 7.5 Hz, 1H), 1.01 (t, *J* = 7.1 Hz, 3H), 0.93 (s, 9H), 0.18 (s, 3H), 0.17 (s, 3H); ^{13}C NMR (125 MHz, $CDCl_3$) (δ, ppm) 171.0, 154.0, 136.8, 135.2, 135.0, 132.9, 130.5 (q, *J* = 32.1 Hz), 128.6, 126.5 (q, *J* = 3.5 Hz), 124.2 (q, *J* = 272.3 Hz), 123.4 (q, *J* = 3.5 Hz), 61.5, 61.0, 48.2, 47.1, 46.1, 39.9, 39.5, 26.3, 18.5, 14.0, -5.1; HRMS (ESI Q-TOF) m/z: $[M+H]^+$ Calcd for $C_{25}H_{33}F_3NO_3Si$ 480.2176, found 480.2177.

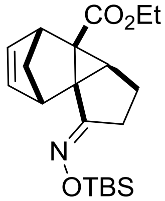

Ethyl (*E*)-1-(*tert*-Butyldimethylsilyloxy)imino-1,2,3,3a,4,7-hexahydro-3b*H*-4,7-methanocyclopenta[1,3]cyclopropa[1,2]benzene-3b-carboxylate (8h). 28.9 mg, 80% yield, >20:1 *dr*, colorless oil; ^{1}H NMR (500 MHz, $CDCl_3$) (δ, ppm) 6.24 – 6.02 (m, 1H), 6.01 – 5.84 (m, 1H), 4.15 (q, *J* = 7.1 Hz, 2H), 3.41 (s, 1H), 3.24 (s, 1H), 2.89 – 2.71 (m, 1H), 2.52 – 2.37 (m, 1H), 2.26 (d, *J* = 7.2 Hz, 1H), 2.12 – 1.94 (comp, 2H), 1.76 (d, *J* = 7.2 Hz, 1H), 1.26 (t, *J* = 7.1 Hz, 3H), 0.92 (s, 9H), 0.15 (s, 3H), 0.14 (s, 3H); ^{13}C NMR (125 MHz, $CDCl_3$) (δ, ppm) 172.5, 167.9, 137.0, 132.1, 62.9, 60.8, 51.5, 49.8, 47.1, 43.8, 39.5, 30.3, 26.3, 22.5, 18.5, 14.4, -5.1, -5.2; HRMS (ESI Q-TOF) m/z: $[M+H]^+$ Calcd for $C_{20}H_{32}NO_3Si$ 362.2146, found 362.2137.

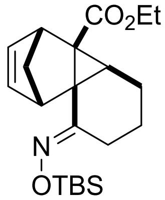

Ethyl (*E*)-8-(*tert*-Butyldimethylsilyloxy)imino-1,4,5,6,7,8-hexahydro-1,4-

methanocyclopropa[1,2:1,3]dibenzene-4a(4b*H*)-carboxylate (8i). 30.8 mg, 82% yield, >20:1 *dr*, colorless oil; ^{1}H NMR (500 MHz, $CDCl_3$) (δ, ppm) 6.04 – 6.00 (m, 1H), 6.00 – 5.96 (m, 1H), 4.12 (q, *J* = 7.0 Hz, 2H), 3.35 (s, 1H), 3.22 (s, 1H), 2.74 – 2.69 (m, 1H), 2.31 – 2.17 (comp, 2H), 1.93 – 1.76 (comp, 3H), 1.73 (d, *J* = 7.1 Hz, 1H), 1.55 – 1.53 (m, 1H), 1.30 – 1.19 (comp, 4H), 0.93 (s, 9H), 0.14 (s, 6H); ^{13}C NMR (125 MHz, $CDCl_3$) (δ, ppm) 172.4, 161.1, 135.4, 133.8, 62.1, 60.6, 49.8, 48.6, 40.1, 37.7, 35.7, 26.4, 23.6, 20.9, 19.5, 18.4, 14.4, -5.0, -5.1; HRMS (ESI Q-TOF) m/z: $[M+H]^+$ Calcd for $C_{21}H_{34}NO_3Si$ 376.2302, found 376.2296.

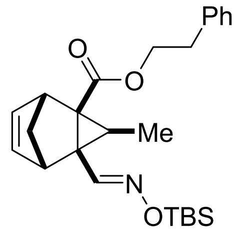

Phenethyl (*E*)-4-(*tert*-Butyldimethylsilyloxy)iminomethyl-3-methyltricyclo-[3.2.1.0^{2,4}]oct-6-ene-2-carboxylate (8j). 33.2 mg, 78% yield, >20:1 *dr*, colorless oil; ^{1}H NMR (500 MHz, $CDCl_3$) (δ, ppm) 7.84 (s, 1H), 7.31 – 7.28 (comp, 2H), 7.24 – 7.20 (comp, 3H), 6.04 – 5.99 (m, 1H), 5.99 – 5.95 (m, 1H), 4.41 – 4.26 (comp, 2H), 3.46 (s, 1H), 3.42 (s, 1H), 2.96 (t, *J* = 7.0 Hz, 2H), 1.81 (d, *J* = 7.3 Hz, 1H), 1.75 (q, *J* = 6.7 Hz, 1H), 1.67 (d, *J* = 7.3 Hz, 1H), 1.23 (d, *J* = 6.7 Hz, 3H), 0.95 (s, 9H), 0.18 (s, 3H), 0.16 (s, 3H); ^{13}C NMR (125 MHz, $CDCl_3$) (δ, ppm) 172.0, 154.1, 139.0, 134.6, 134.2, 129.0, 128.6, 126.7, 65.3, 61.3, 48.1, 46.8, 40.0, 39.8, 38.0, 35.3, 26.4, 18.5, 10.1, -5.1; HRMS (ESI Q-TOF) m/z: $[M+H]^+$ Calcd for $C_{25}H_{36}NO_3Si$ 426.2459, found 426.2453.

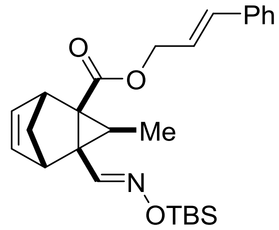

Cinnamyl 4-(*E*)-(*tert*-Butyldimethylsilyloxy)iminomethyl-3-methyltricyclo-[3.2.1.0^{2,4}]oct-6-ene-2-carboxylate (8k). 34.5 mg, 79% yield, >20:1 *dr*, white solid, mp: 105.0-107.0 °C; ^{1}H NMR (500 MHz, $CDCl_3$) (δ, ppm) 7.91 (s, 1H), 7.39 (d, *J* = 7.5 Hz, 2H), 7.34 – 7.31 (comp, 2H), 7.27 (d, *J* = 7.5 Hz, 1H), 6.65 (d, *J* = 15.9 Hz, 1H), 6.36 – 6.23 (m, 1H), 6.07 – 6.02 (m, 1H), 6.02 – 5.96 (m, 1H), 4.78 (d, *J* = 6.3 Hz, 2H), 3.52 (s, 1H), 3.50 (s, 1H), 1.91 (d, *J* = 7.3 Hz, 1H), 1.81 (q, *J* = 6.7 Hz, 1H), 1.73

(d, J = 7.3 Hz, 1H), 1.33 (d, J = 6.7 Hz, 3H), 0.93 (s, 9H), 0.17 (s, 3H), 0.15 (s, 3H); ^{13}C NMR (125 MHz, $CDCl_3$) (δ, ppm) 171.8, 154.0, 136.4, 134.6, 134.2, 134.1, 128.7, 128.2, 126.8, 123.5, 65.4, 61.3, 48.2, 46.9, 40.0, 39.9, 38.1, 26.4, 18.5, 10.2, -5.1; HRMS (ESI Q-TOF) m/z: $[M+H]^+$ Calcd for $C_{26}H_{36}NO_3Si$ 438.2459, found 438.2461.

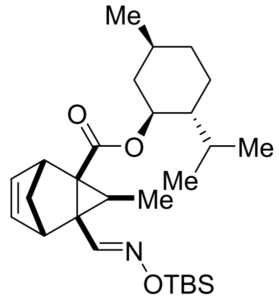

(1*S*,2*R*,5*S*)-2-Isopropyl-5-methylcyclohexyl 4-(*E*)-(*tert*-Butyldimethylsilyloxy)-iminomethyl-3-methyltricyclo[3.2.1.0^{2,4}]oct-6-ene-2-carboxylate (8l). 36.3 mg, 79% yield, 1:1 *dr*, colorless oil; composite NMR signals of two diastereoisomers: ^{1}H NMR (500 MHz, $CDCl_3$) (δ, ppm) 7.86 (s, 1H), 6.08 – 6.00 (m, 1H), 6.01 – 5.94 (m, 1H), 4.77 – 4.72 (m, 1H), 3.49 – 3.44 (comp, 2H), 1.96 (d, J = 11.8 Hz, 1H), 1.91 – 1.82 (comp, 2H), 1.80 – 1.73 (m, 1H), 1.72 – 1.67 (comp, 3H), 1.43 – 1.39 (m, 1H), 1.33 – 1.29 (comp, 3H), 1.11 – 0.96 (m, 1H), 0.95 – 0.94 (comp, 9H), 0.92 – 0.85 (comp, 8H), 0.74 (t, J = 6.8 Hz, 3H), 0.17 – 0.16 (comp, 3H), 0.15 – 0.14 (comp, 3H); ^{13}C NMR (125 MHz, $CDCl_3$) (δ, ppm) 171.72, 171.69, 154.14, 154.09, 134.6, 134.5, 134.4, 134.3, 74.72, 74.69, 61.3, 61.2, 48.4, 48.3, 47.3, 47.1, 46.8, 46.7, 41.3, 41.2, 40.2, 39.7, 39.3, 37.9, 37.7, 34.4, 31.62, 31.58, 26.43, 26.38, 26.3, 26.2, 23.4, 23.2, 22.2, 21.12, 21.08, 18.54, 18.47, 16.3, 16.0, 10.3, -5.08, -5.10; HRMS (ESI Q-TOF) m/z: $[M+H]^+$ Calcd for $C_{27}H_{46}NO_3Si$ 460.3241, found 460.3233.

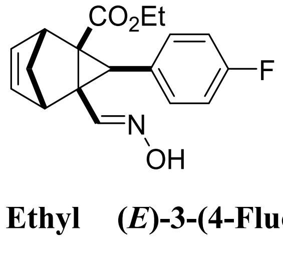

Ethyl (*E*)-3-(4-Fluorophenyl)-4-((hydroxyimino)methyl)tricyclo[3.2.1.0^{2,4}]oct-6-ene-2-carboxylate (8m). 26.8 mg, 85% yield, >20:1 *dr*, colorless oil; ^{1}H NMR (500 MHz, $CDCl_3$) (δ, ppm) 7.72 (s, 1H), 6.99 – 6.93 (comp, 4H), 6.34 – 6.15 (m, 1H), 6.13 – 5.98 (m, 1H), 4.16 – 3.88 (comp, 2H), 3.69 (s, 1H), 3.64 (s, 1H), 2.97 (s, 1H), 1.93

(d, J = 7.5 Hz, 1H), 1.80 (d, J = 7.5 Hz, 1H), 1.06 (t, J = 7.1 Hz, 3H); ^{13}C NMR (125 MHz, $CDCl_3$) (δ, ppm) 171.2, 161.7 (d, J = 245.4 Hz), 151.7, 135.1, 134.6, 131.0 (d, J = 3.4 Hz), 130.9 (d, J = 7.9 Hz), 115.2 (d, J = 21.4 Hz), 61.3, 61.1, 48.2, 46.7, 46.2, 40.2, 38.9, 14.1; HRMS (ESI Q-TOF) m/z: $[M+H]^+$ Calcd for $C_{18}H_{19}FNO_3$ 316.1343, found 316.1334.

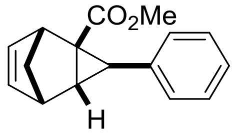

Methyl 3-Phenyltricyclo[3.2.1.0^{2,4}]oct-6-ene-2-carboxylate (8n). 20.4 mg, 85% yield, >20:1 *dr*, colorless oil; ^{1}H NMR (500 MHz, $CDCl_3$) (δ, ppm) 7.24 – 7.21 (comp, 2H), 7.16 – 7.12 (comp, 3H), 6.17 – 6.06 (m, 1H), 6.01 – 5.91 (m, 1H), 3.50 (s, 1H), 3.36 (s, 3H), 3.11 (s, 1H), 2.60 (t, J = 4.5 Hz, 1H), 2.53 (d, J = 4.5 Hz, 1H), 1.83 (d, J = 7.2 Hz, 1H), 1.76 (d, J = 7.2 Hz, 1H); ^{13}C NMR (125 MHz, $CDCl_3$) (δ, ppm) 173.4, 137.6, 133.6, 132.9, 128.9, 127.9, 126.4, 62.3, 51.5, 46.4, 43.93, 43.89, 36.2, 28.5; HRMS (ESI Q-TOF) m/z: $[M+H]^+$ Calcd for $C_{16}H_{17}O_2$ 241.1223, found 241.1217.

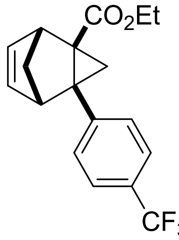

Ethyl 4-(4-(Trifluoromethyl)phenyl)tricyclo[3.2.1.0^{2,4}]oct-6-ene-2-carboxylate (8o). 25.6 mg, 83% yield, >20:1 *dr*, colorless oil; ^{1}H NMR (500 MHz, $CDCl_3$) (δ, ppm) 7.53 (d, J = 8.2 Hz, 2H), 7.45 (d, J = 8.2 Hz, 2H), 6.19 – 6.13 (m, 1H), 6.13 – 6.05 (m, 1H), 3.98 – 3.78 (comp, 2H), 3.48 (s, 1H), 3.02 (s, 1H), 2.53 (d, J = 7.4 Hz, 1H), 2.28 – 2.27 (m, 1H), 2.00 (d, J = 6.4 Hz, 1H), 1.61 (d, J = 5.5 Hz, 1H), 0.87 (t, J = 7.1 Hz, 3H); ^{13}C NMR (125 MHz, $CDCl_3$) (δ, ppm) 172.4, 144.1, 135.4, 135.1, 129.0 (q, J = 3.5 Hz), 128.9, 126.2 (q, J = 272.3 Hz), 125.1 (q, J = 3.5 Hz), 63.7, 60.5, 51.7, 45.7, 42.3, 38.2, 28.7, 14.1; HRMS (ESI Q-TOF) m/z: $[M+H]^+$ Calcd for $C_{18}H_{18}F_3O_2$ 323.1253, found 323,1248.

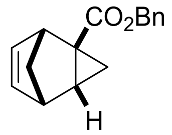

Benzyl Tricyclo[3.2.1.0^{2,4}]oct-6-ene-2-carboxylate (8p). 20.2 mg, 84% yield, >20:1 *dr*, colorless oil; ^{1}H NMR (500 MHz, $CDCl_3$) (δ, ppm) 7.43 – 7.28 (comp, 5H), 6.06 – 5.90 (m, 1H), 5.82 – 5.80 (m, 1H), 5.28 – 5.02 (comp, 2H), 3.36 (s, 1H), 2.92 (s, 1H), 2.20 – 2.05 (m, 1H), 1.95 (d, *J* = 7.2 Hz, 1H), 1.81 (d, *J* = 7.2 Hz, 1H), 1.77 – 1.74 (m, 1H), 1.15 (t, *J* = 4.7 Hz, 1H); ^{13}C NMR (125 MHz, $CDCl_3$) (δ, ppm) 175.6, 136.7, 133.0, 132.7, 128.6, 128.1, 127.9, 66.2, 63.9, 43.9, 43.5, 29.7, 28.9, 27.2; HRMS (ESI Q-TOF) m/z: $[M+H]^+$ Calcd for $C_{16}H_{17}O_2$ 241.1223, found 241.1219.

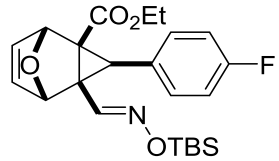

Ethyl (*E*)-4-(*tert*-Butyldimethylsilyloxy)iminomethyl-3-(4-fluorophenyl)-8-oxatricyclo[3.2.1.0^{2,4}]oct-6-ene-2-carboxylate (8q). 34.5 mg, 80% yield, >20:1 *dr*, colorless oil; ^{1}H NMR (500 MHz, $CDCl_3$) (δ, ppm) 7.54 (s, 1H), 7.10 – 7.08 (comp, 2H), 6.99 (t, *J* = 8.6 Hz, 2H), 6.80 – 6.70 (m, 1H), 6.61 – 6.50 (m, 1H), 5.12 (s, 1H), 5.07 (s, 1H), 4.05 – 3.99 (comp, 2H), 3.83 (s, 1H), 1.06 (t, *J* = 7.1 Hz, 3H), 0.95 (s, 9H), 0.20 (s, 3H), 0.16 (s, 3H); ^{13}C NMR (125 MHz, $CDCl_3$) (δ, ppm) 169.1, 161.8 (d, *J* = 245.8 Hz), 152.2, 140.6, 137.2, 131.1 (d, *J* = 8.0 Hz), 129.9 (d, *J* = 3.2 Hz), 115.3 (d, *J* = 21.5 Hz), 79.8, 78.9, 61.0, 46.2, 43.5, 40.7, 26.3, 18.5, 14.1, -5.06, -5.11; HRMS (ESI Q-TOF) m/z: $[M+H]^+$ Calcd for $C_{23}H_{31}FNO_4Si$ 432.2001, found 432.2002.

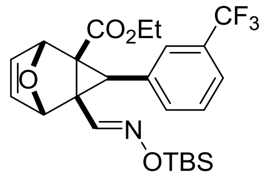

Ethyl (*E*)-4-(*tert*-Butyldimethylsilyloxy)iminomethyl-3-(3-(trifluoromethyl)-phenyl)-8-oxatricyclo[3.2.1.0^{2,4}]oct-6-ene-2-carboxylate (8r). 40.4 mg, 84% yield, >20:1 *dr*, colorless oil; ^{1}H NMR (500 MHz, $CDCl_3$) (δ, ppm) 7.52 – 7.50 (comp, 2H), 7.43 (t, *J* = 7.7 Hz, 1H), 7.40 – 7.30 (comp, 2H), 6.76 (d, *J* = 5.5 Hz, 1H), 6.59 (d, *J* = 5.5 Hz, 1H), 5.14 (s, 1H), 5.10 (s, 1H), 4.01 (q, *J* = 7.1 Hz, 2H), 3.89 (s, 1H), 1.03

(t, J = 7.1 Hz, 3H), 0.95 (s, 9H), 0.20 (s, 3H), 0.17 (s, 3H); ^{13}C NMR (125 MHz, $CDCl_3$) (δ, ppm) 168.9, 151.8, 140.4, 137.3, 135.4, 132.9, 130.8 (q, J = 32.1 Hz), 128.9, 126.5 (q, J = 3.5 Hz), 124.1 (q, J = 272.2 Hz), 123.9 (q, J = 3.6 Hz), 79.8, 78.9, 61.2, 46.1, 43.4, 40.7, 26.2, 18.5, 14.0, -5.1, -5.2; HRMS (ESI Q-TOF) m/z: $[M+H]^+$ Calcd for $C_{24}H_{31}F_3NO_4Si$ 482.1969, found 482.1962.

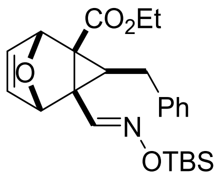

Ethyl (*E*)-3-Benzyl-4-(*tert*-butyldimethylsilyloxy)iminomethyl-8-oxatricyclo-[3.2.1.0^{2,4}]oct-6-ene-2-carboxylate (8s). 34.2 mg, 80% yield, >20:1 *dr*, colorless oil; ^{1}H NMR (500 MHz, $CDCl_3$) (δ, ppm) 7.85 (s, 1H), 7.33 – 7.29 (comp, 2H), 7.24 – 7.20 (comp, 3H), 6.74 (d, J = 5.5 Hz, 1H), 6.56 (d, J = 5.5 Hz, 1H), 4.95 (s, 1H), 4.92 (s, 1H), 4.29 – 4.09 (comp, 2H), 3.24 (dd, J = 15.1, 8.5 Hz, 1H), 3.17 (dd, J = 15.1, 6.8 Hz, 1H), 2.97 – 2.90 (m, 1H), 1.26 (t, J = 7.2 Hz, 3H), 0.97 (s, 9H), 0.20 (s, 3H), 0.18 (s, 3H); ^{13}C NMR (125 MHz, $CDCl_3$) (δ, ppm) 170.0, 151.6, 148.8, 140.3, 137.6, 128.7, 128.4, 126.4, 79.6, 78.9, 61.0, 46.8, 43.0, 39.5, 29.7, 26.3, 18.5, 14.4, -5.09, -5.13; HRMS (ESI Q-TOF) m/z: $[M+H]^+$ Calcd for $C_{24}H_{34}NO_4Si$ 428.2252, found 428.2248.

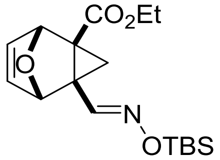

Ethyl (*E*)-4-(*tert*-Butyldimethylsilyloxy)iminomethyl-8-oxatricyclo[3.2.1.0^{2,4}]oct-6-ene-2-carboxylate (8t). 24.6 mg, 73% yield, >20:1 *dr*, colorless oil; ^{1}H NMR (500 MHz, $CDCl_3$) (δ, ppm) 7.47 (s, 1H), 6.72 (d, J = 5.5 Hz, 1H), 6.65 (d, J = 5.5 Hz, 1H), 4.92 (s, 1H), 4.87 (s, 1H), 4.13 (q, J = 7.0 Hz, 2H), 2.33 (d, J = 5.0 Hz, 1H), 1.95 (d, J = 5.0 Hz, 1H), 1.24 (t, J = 7.0 Hz, 3H), 0.93 (s, 9H), 0.17 (s, 3H), 0.15 (s, 3H); ^{13}C NMR (125 MHz, $CDCl_3$) (δ, ppm) 170.7, 153.0, 139.8, 138.6, 79.3, 78.3, 61.2, 46.0, 40.9, 26.5, 26.2, 18.4, 14.4, -5.1, -5.2; HRMS (ESI Q-TOF) m/z: $[M+H]^+$ Calcd for $C_{17}H_{28}NO_4Si$ 338.1782, found 338.1777.

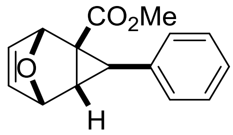

Methyl 3-Phenyl-8-oxatricyclo[3.2.1.0^{2,4}]oct-6-ene-2-carboxylate (8u). 19.3 mg, 80% yield, >20:1 *dr*, colorless oil; ^{1}H NMR (300 MHz, $CDCl_3$) (δ, ppm) 7.33 – 7.16 (comp, 5H), 6.65 – 6.61 (comp, 2H), 5.09 (d, *J* = 1.4 Hz, 1H), 4.91 (d, *J* = 1.4 Hz, 1H), 3.67 (d, *J* = 5.0 Hz, 1H), 3.40 (s, 3H), 2.36 (d, *J* = 5.0 Hz, 1H); ^{13}C NMR (75 MHz, $CDCl_3$) (δ, ppm) 170.6, 138.7, 138.2, 136.0, 129.0, 128.0, 126.9, 78.3, 78.2, 51.5, 42.8, 42.6, 34.8; HRMS (ESI Q-TOF) m/z: $[M+H]^+$ Calcd for $C_{15}H_{15}O_3$ 243.1016, found 243.1018.

Synthetic Transformations:

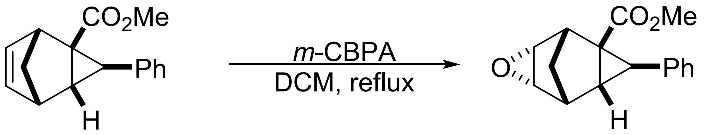

8n **9**, 88% yield, >20:1 *dr*

Synthesis of 9: To a 10-mL oven-dried round-bottom flask with a magnetic stirring bar, **8n** (25.4 mg, 0.1 mmol), *m*-CPBA (35.0 mg, 0.2 mmol, 2.0 equiv.), and DCM (2.0 mL) were added in sequence. Then the reaction mixture was stirred overnight at reflux. When the reaction was complete (monitored by TLC), the reaction mixture was purified by flash column chromatography on silica gel without additional treatment (hexanes:EtOAc = 10:1) to give 23.8 mg pure product **9** as colorless oil, 88% yield, >20:1 *dr*; ^{1}H NMR (300 MHz, $CDCl_3$) (δ, ppm) 7.28 – 7.22 (comp, 2H), 7.21 – 7.11 (comp, 2H), 3.75 (d, *J* = 4.9 Hz, 1H), 3.33 (s, 3H), 3.26 (dd, *J* = 3.0, 1.6 Hz, 1H), 3.25 – 3.22 (m, 1H), 3.17 (dd, *J* = 3.0, 1.6 Hz, 1H), 2.78 – 2.77 (m, 1H), 2.58 (t, *J* = 4.8 Hz, 1H), 1.68 (d, *J* = 9.2 Hz, 1H), 1.32 (d, *J* = 9.2 Hz, 1H); ^{13}C NMR (75 MHz, $CDCl_3$) (δ, ppm) 172.0, 136.9, 128.8, 128.1, 126.8, 51.6, 49.8, 49.7, 41.2, 40.0, 39.6, 39.3, 37.0, 34.1; HRMS (ESI Q-TOF) m/z: $[M+H]^+$ Calcd for $C_{16}H_{17}O_3$ 257.1172, found 257.1173.

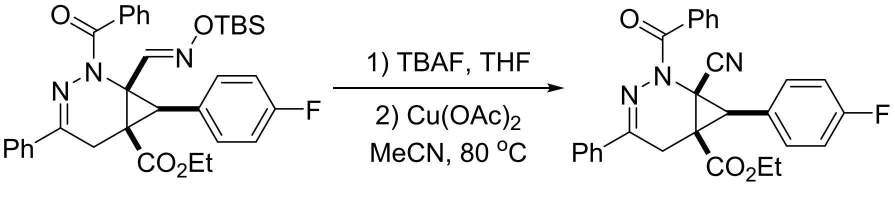

3f **10f**

Synthesis of 10f: To a 10-mL oven-dried round-bottom flask with a magnetic stirring bar, TBAF (31.4 mg, 1.2 equiv.) in THF was added to the solution of **3f** (46.7 mg, 0.1 mmol) in THF (1.0 mL), and the reaction mixture was stirred for 10 min at 0 °C. When the reaction was complete (monitored by TLC), the reaction mixture was purified by flash column chromatography on silica gel without additional treatment (hexanes:EtOAc = 5:1) to give deprotected products. Then, $Cu(OAc)_2$ (1.0 mg, 5.0 mol %), deprotected products, and MeCN (2.0 mL) were added in sequence, and the reaction mixture was stirred at 80 °C overnight. When the reaction was complete (monitored by TLC), the reaction mixture was purified by flash column chromatography on silica gel without additional treatment (hexanes:EtOAc = 10:1) to give pure product

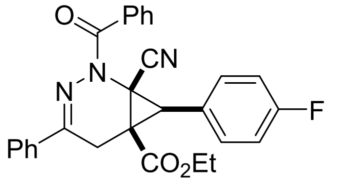

Ethyl 2-Benzoyl-1-cyano-7-(4-fluorophenyl)-4-phenyl-2,3-diazabicyclo[4.1.0]-hept-3-ene-6-carboxylate (10f). 32.7 mg, 70% yield, >20:1 *dr*, colorless oil; ^1H NMR (300 MHz, $CDCl_3$) (δ, ppm) 7.88 – 7.85 (comp, 2H), 7.78 – 7.65 (comp, 4H), 7.57 – 7.52 (m, 1H), 7.50 – 7.33 (comp, 5H), 7.12 (t, J = 8.7 Hz, 2H), 4.21 (d, J = 17.9 Hz, 1H), 4.15 – 3.89 (comp, 2H), 2.98 (s, 1H), 2.61 (d, J = 17.9 Hz, 1H), 0.96 (t, J = 7.2 Hz, 3H); ^{13}C NMR (75 MHz, $CDCl_3$) (δ, ppm) 170.3, 166.8, 162.8 (d, J = 247.7 Hz), 152.8, 134.7, 133.1, 131.9, 131.3 (d, J = 8.4 Hz), 130.9, 130.5, 129.0, 127.9, 126.9 (d, J = 3.1 Hz), 126.3, 115.9 (d, J = 21.7 Hz), 113.8, 62.9, 43.0, 36.3, 35.8, 26.5, 13.4; HRMS (ESI Q-TOF) m/z: $[M+H]^+$ Calcd for $C_{28}H_{23}FN_3O_3$ 468.1718, found 468.1719.

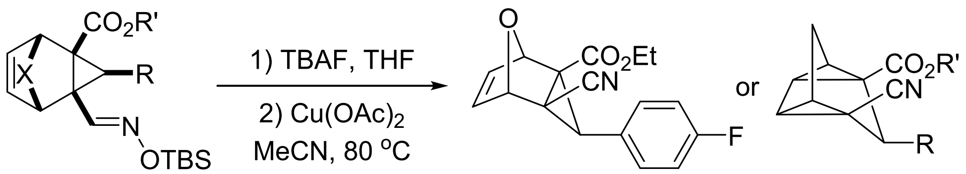

8 10q 11

Synthesis of 10q or 11: To a 10-mL oven-dried round-bottom flask with a magnetic stirring bar, TBAF (31.4 mg, 1.2 equiv.) in THF was added to the solution of **8** (0.1 mmol) in THF (1.0 mL), and the reaction mixture was stirred for 10 min at 0 °C. When

the reaction was complete (monitored by TLC), the reaction mixture was purified by flash column chromatography on silica gel without additional treatment (hexanes:EtOAc = 5:1) to give deprotected products. Then, $Cu(OAc)_2$ (1.0 mg, 5.0 mol %), deprotected products, and MeCN (2.0 mL) were added in sequence, and the reaction mixture was stirred at 80 °C overnight. When the reaction was complete (monitored by TLC), the reaction mixture was purified by flash column chromatography on silica gel without additional treatment (hexanes:EtOAc = 10:1) to give pure product

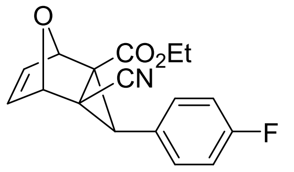

Ethyl 4-Cyano-3-(4-fluorophenyl)-8-oxatricyclo[3.2.1.02,4]oct-6-ene-2-carboxylate (10q). 21.5 mg, 72% yield, >20:1 *dr*, colorless oil; ^{1}H NMR (300 MHz, $CDCl_3$) (δ, ppm) 7.36 – 7.33 (comp, 2H), 7.04 (t, J = 8.6 Hz, 2H), 6.93 – 6.67 (comp, 2H), 5.21 (s, 1H), 5.11 (s, 1H), 4.00 (q, J = 7.1 Hz, 2H), 3.88 (s, 1H), 0.98 (t, J = 7.1 Hz, 3H); ^{13}C NMR (75 MHz, $CDCl_3$) (δ, ppm) 166.6, 162.4 (d, J = 247.2 Hz), 140.2, 137.7, 130.3 (d, J = 8.2 Hz), 128.5 (d, J = 3.2 Hz), 116.7, 115.6 (d, J = 21.7 Hz), 79.9, 79.2, 61.8, 48.7, 42.3, 33.4, 13.8; HRMS (ESI Q-TOF) m/z: $[M+H]^+$ Calcd for $C_{17}H_{15}FNO_3$ 300.1030, found 300.1029.

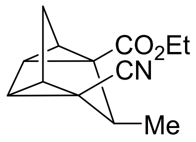

Ethyl 2b-Cyano-1-methylhexahydrodicyclopropa[*cd*,*gh*]pentalene-1a(1*H*)-carboxylate (11a). 18.9 mg, 81% yield, >20:1 *dr*, colorless oil; ^{1}H NMR (300 MHz, $CDCl_3$) (δ, ppm) 4.22 – 3.91 (comp, 2H), 3.02 (q, J = 6.9 Hz, 1H), 2.48 (dd, J = 6.2, 3.8 Hz, 1H), 2.37 (dd, J = 6.2, 3.8 Hz, 1H), 2.32 – 2.24 (m, 1H), 2.19 (d, J = 13.5 Hz, 1H), 2.10 – 1.93 (comp, 2H), 1.29 – 1.21 (comp, 6H); ^{13}C NMR (75 MHz, $CDCl_3$) (δ, ppm) 170.9, 120.4, 60.9, 40.6, 36.7, 33.6, 33.5, 32.3, 31.0, 26.5, 26.1, 14.4, 13.5; HRMS (ESI Q-TOF) m/z: $[M+H]^+$ Calcd for $C_{13}H_{16}NO_2$ 218.1176, found 218.1177.

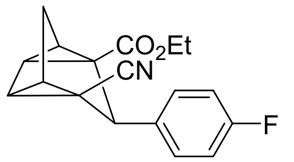

Ethyl 2b-Cyano-1-(4-fluorophenyl)hexahydrodicyclopropa[*cd,gh*]pentalene-1a(1*H*)-carboxylate (11f). 25.7 mg, 82% yield, >20:1 *dr*, colorless oil; ^{1}H NMR (300 MHz, $CDCl_3$) (δ, ppm) 7.50 – 7.41 (comp, 2H), 7.07 – 6.97 (comp, 2H), 4.33 (s, 1H), 4.17 – 4.04 (comp, 2H), 2.63 (dd, *J* = 6.3, 3.9 Hz, 1H), 2.59 – 2.53 (m, 1H), 2.50 (dd, *J* = 6.3, 3.9 Hz, 1H), 2.19 – 2.09 (m, 1H), 1.98 (t, *J* = 2.5 Hz, 1H), 1.16 (t, *J* = 7.1 Hz, 3H); ^{13}C NMR (75 MHz, $CDCl_3$) (δ, ppm) 170.4, 162.0 (d, *J* = 245.6 Hz), 133.1 (d, *J* = 3.2 Hz), 129.1 (d, *J* = 7.9 Hz), 120.7, 115.5 (d, *J* = 21.3 Hz), 61.3, 44.0, 40.5, 34.0, 33.7, 33.5, 32.1, 26.3, 26.0, 14.3; HRMS (ESI Q-TOF) m/z: $[M+H]^+$ Calcd for $C_{18}H_{17}FNO_2$ 298.1238, found 298.1242.

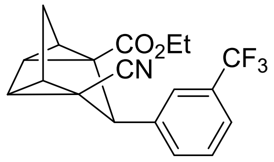

Ethyl 2b-Cyano-1-(3-(trifluoromethyl)phenyl)hexahydrodicyclopropa[*cd,gh*]-pentalene-1a(1*H*)-carboxylate (11g). 28.7 mg, 79% yield, >20:1 *dr*, colorless oil; ^{1}H NMR (300 MHz, $CDCl_3$) (δ, ppm) 7.77 – 7.74 (comp, 2H), 7.59 – 7.48 (comp, 2H), 4.42 (s, 1H), 4.22 – 4.08 (comp, 1H), 2.71 – 2.67 (m, 1H), 2.64 – 2.54 (comp, 2H), 2.21 (t, *J* = 5.2 Hz, 1H), 2.03 (t, *J* = 4.2 Hz, 1H), 2.00 – 1.95 (m, 1H), 1.19 (t, *J* = 7.1 Hz, 3H); ^{13}C NMR (75 MHz, $CDCl_3$) (δ, ppm) 170.2, 138.5, 131.2, 129.2, 126.2 (q, *J* = 272.3 Hz), 126.0, 124.3 (q, *J* = 3.7 Hz), 120.4, 61.4, 44.5, 40.3, 34.0, 33.8, 33.5, 32.1, 26.4, 25.8, 14.2; HRMS (ESI Q-TOF) m/z: $[M+H]^+$ Calcd for $C_{19}H_{17}F_3NO_2$ 348.1206, found 348.1205.

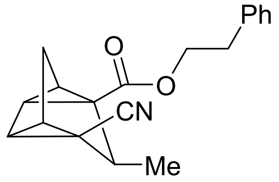

Phenethyl 2b-Cyano-1-methylhexahydrodicyclopropa[*cd,gh*]pentalene-1a(1*H*)-carboxylate (11j). 24.7 mg, 80% yield, >20:1 *dr*, colorless oil; ^{1}H NMR (300 MHz, $CDCl_3$) (δ, ppm) 7.37 – 7.28 (comp, 2H), 7.25 – 7.14 (comp, 3H), 4.38 – 4.16 (comp, 2H), 3.00 – 2.96 (m, 1H), 2.91 (t, *J* = 6.8 Hz, 2H), 2.46 (dd, *J* = 6.1, 3.8 Hz, 1H), 2.31

(dd, J = 6.3, 3.8 Hz, 1H), 2.27 – 2.20 (m, 1H), 2.17 (d, J = 13.6 Hz, 1H), 2.09 – 1.92 (comp, 1H), 1.20 (d, J = 6.9 Hz, 3H); ^{13}C NMR (75 MHz, $CDCl_3$) (δ, ppm) 170.8, 137.8, 129.0, 128.7, 126.8, 120.3, 65.4, 40.5, 36.6, 35.2, 33.7, 32.3, 31.0, 26.5, 26.1, 13.4; HRMS (ESI Q-TOF) m/z: $[M+H]^+$ Calcd for $C_{19}H_{20}NO_2$ 294.1489, found 294.1490.

NMR Analysis of Compound 11f:

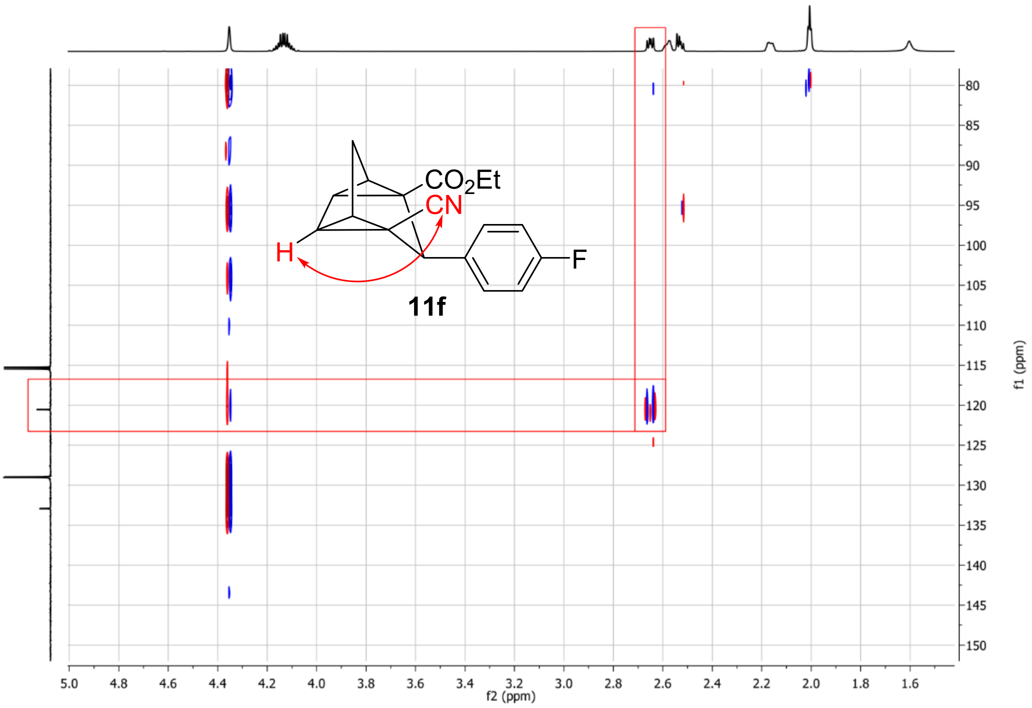

Figure S1. HMBC NMR spectra of **11f**.

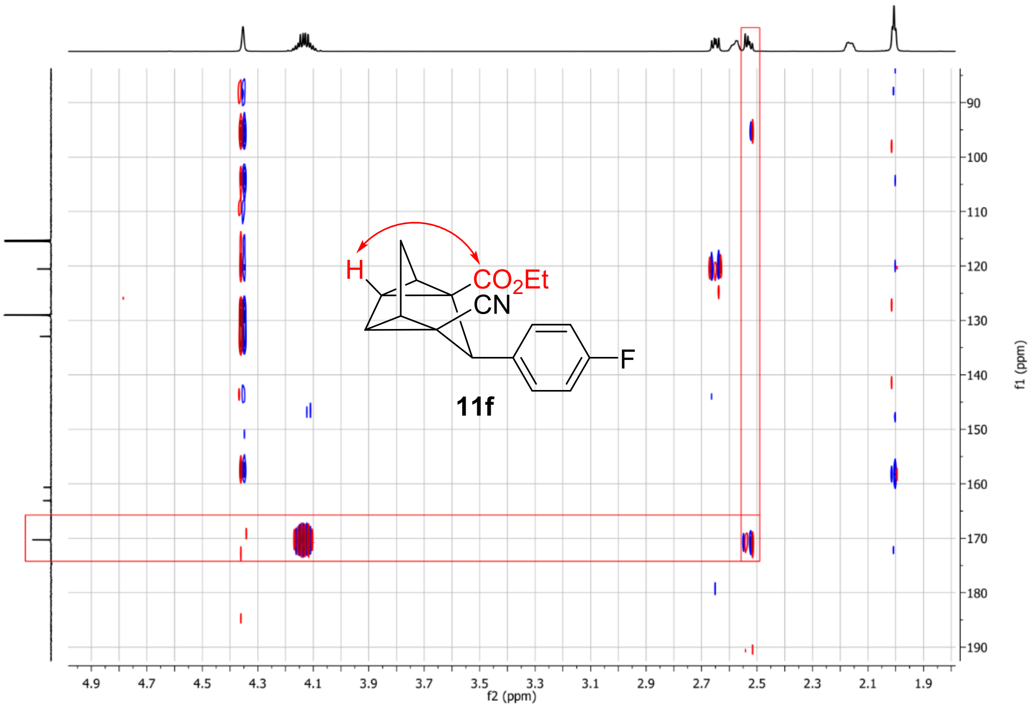

Figure S2. HMBC NMR spectra of **11f**.

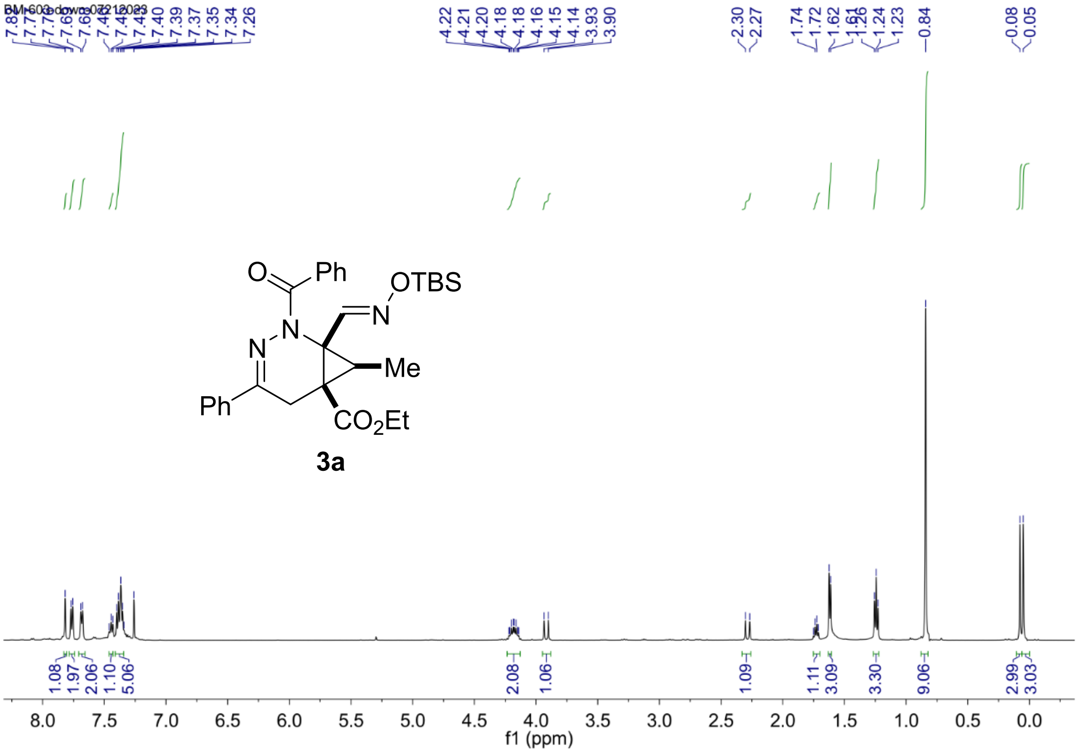
BM-603-down-07212023
4.22 4.21 4.20 4.18 4.18 4.16 4.15 4.14 3.93 3.90
2.30 2.27
1.74 1.72 1.62 1.26 1.24 1.23
0.84
0.08 0.05
O
Ph
OTBS
N
N
N
Me
Ph
CO_2Et
3a
1.08 1.97 2.06 1.10 5.06
2.08
1.06
1.09
1.11 3.09
3.30
9.06
2.99 3.03
f1 (ppm)

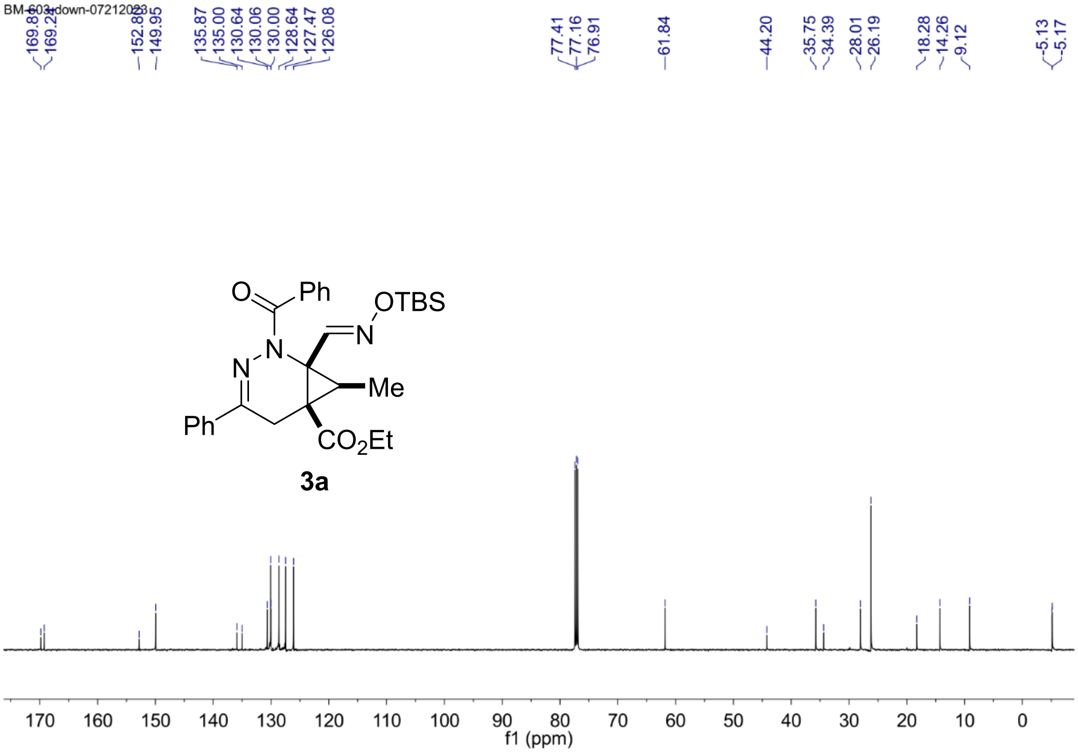
BM-603-down-07212023
135.87 135.00 130.64 130.06 130.00 128.64 127.47 126.08
149.95
77.41 77.16 76.91
61.84
44.20
35.75 34.39 28.01 26.19
18.28 14.26 9.12
-5.13 -5.17
O
Ph
OTBS
N
N
N
Me
Ph
CO_2Et
3a
f1 (ppm)

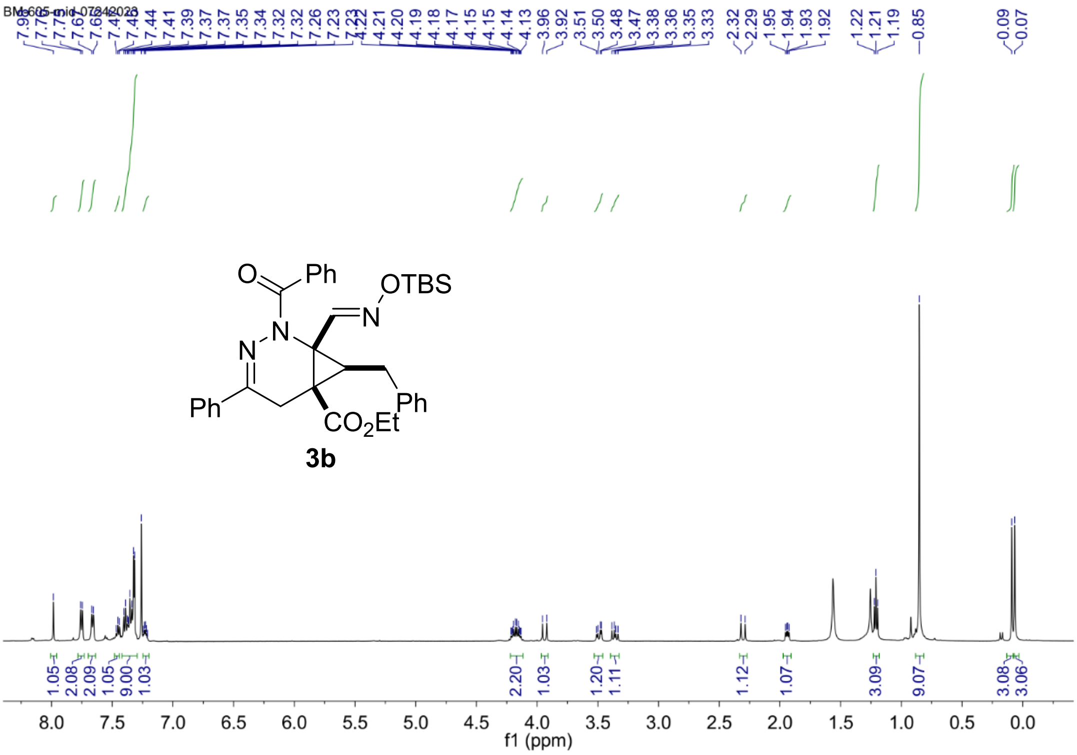
7.90
7.77
7.44
7.41
7.39
7.37
7.37
7.35
7.34
7.32
7.32
7.26
7.23
4.21
4.20
4.19
4.18
4.17
4.15
4.15
4.14
4.13
3.96
3.92
3.51
3.50
3.48
3.47
3.38
3.36
3.35
3.33
2.32
2.29
1.95
1.94
1.93
1.92
1.22
1.21
1.19
0.85
0.09
0.07
O
Ph
OTBS
N
N
N
Ph
Ph
CO_2Et
3b
1.05
2.08
2.09
1.05
9.00
1.03
2.20
1.03
1.20
1.11
1.12
1.07
3.09
9.07
3.08
3.06
8.0 7.5 7.0 6.5 6.0 5.5 5.0 4.5 4.0 3.5 3.0 2.5 2.0 1.5 1.0 0.5 0.0
f1 (ppm)

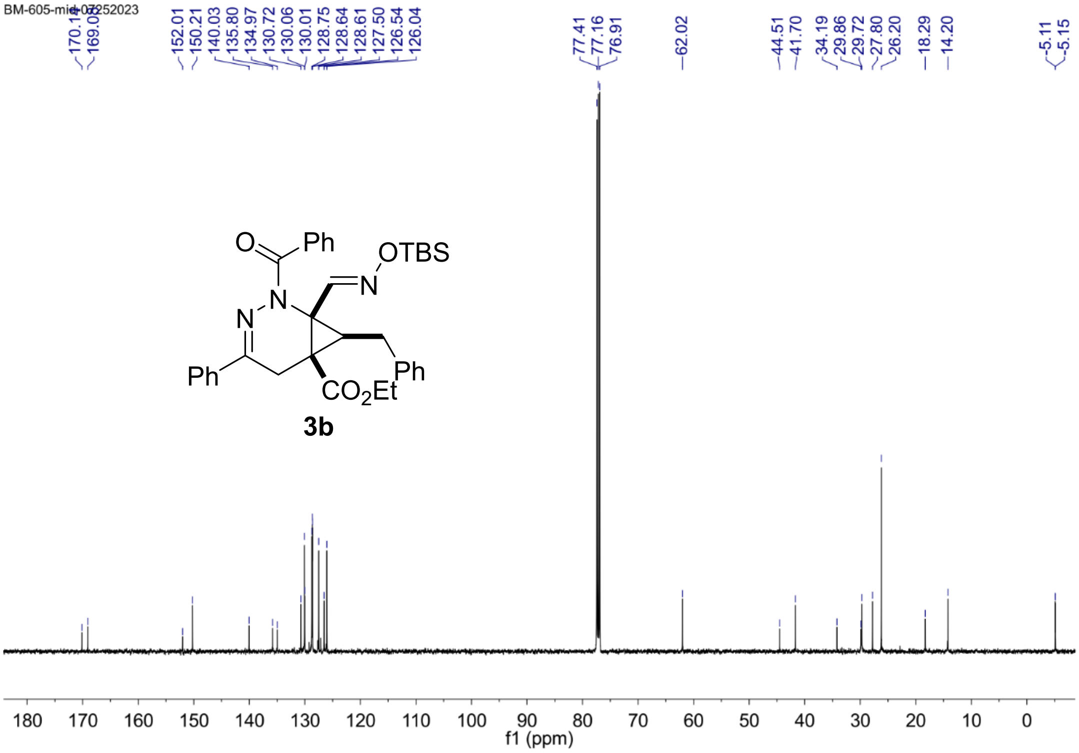
BM-605-mid-07252023
152.01
150.21
140.03
135.80
134.97
130.72
130.06
130.01
128.75
128.64
128.61
127.50
126.54
126.04
77.41
77.16
76.91
62.02
44.51
41.70
34.19
29.86
29.72
27.80
26.20
18.29
14.20
-5.11
-5.15
O
Ph
OTBS
N
N
N
Ph
Ph
CO_2Et
3b
180 170 160 150 140 130 120 110 100 90 80 70 60 50 40 30 20 10 0
f1 (ppm)

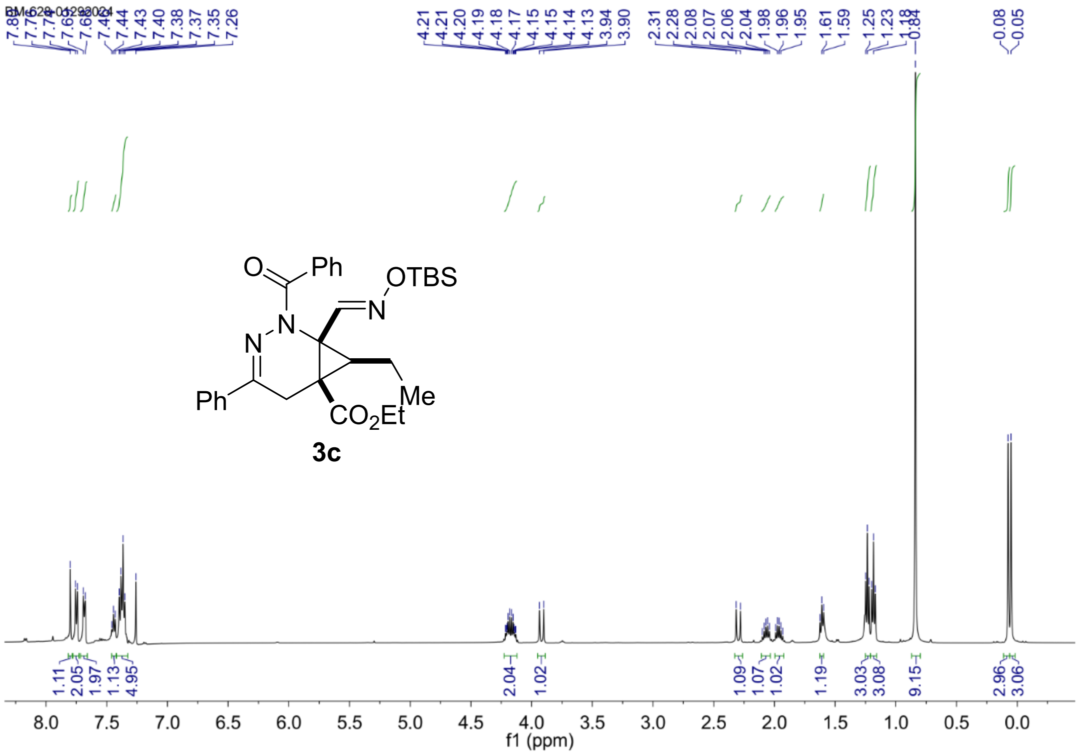

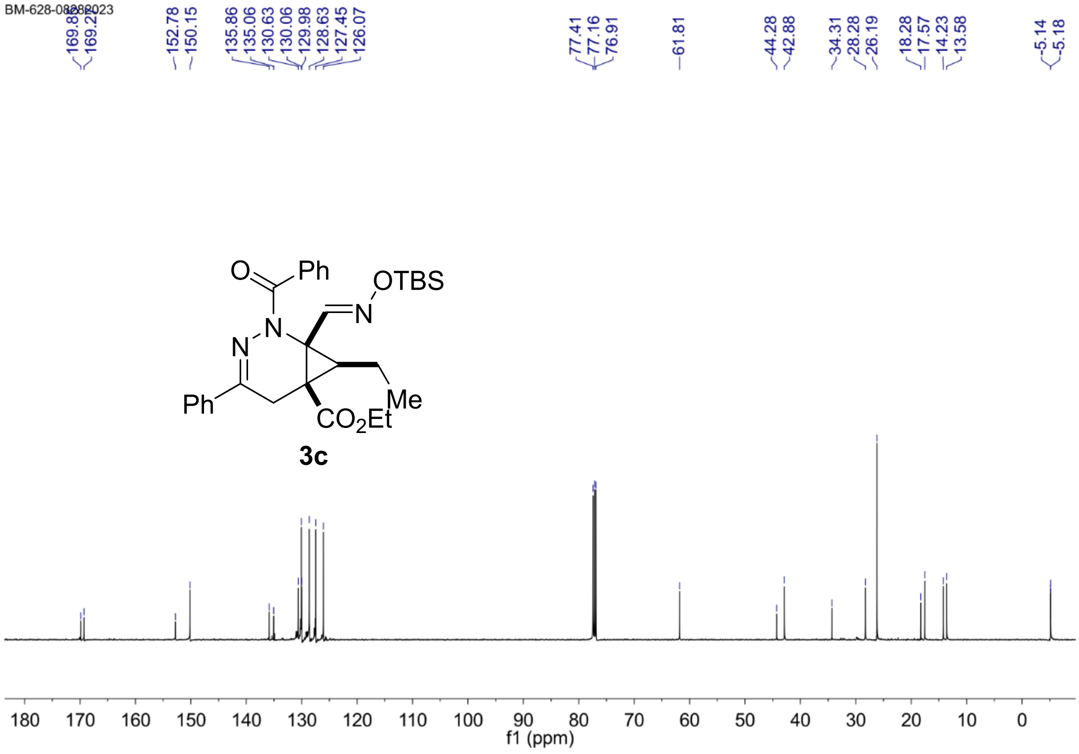

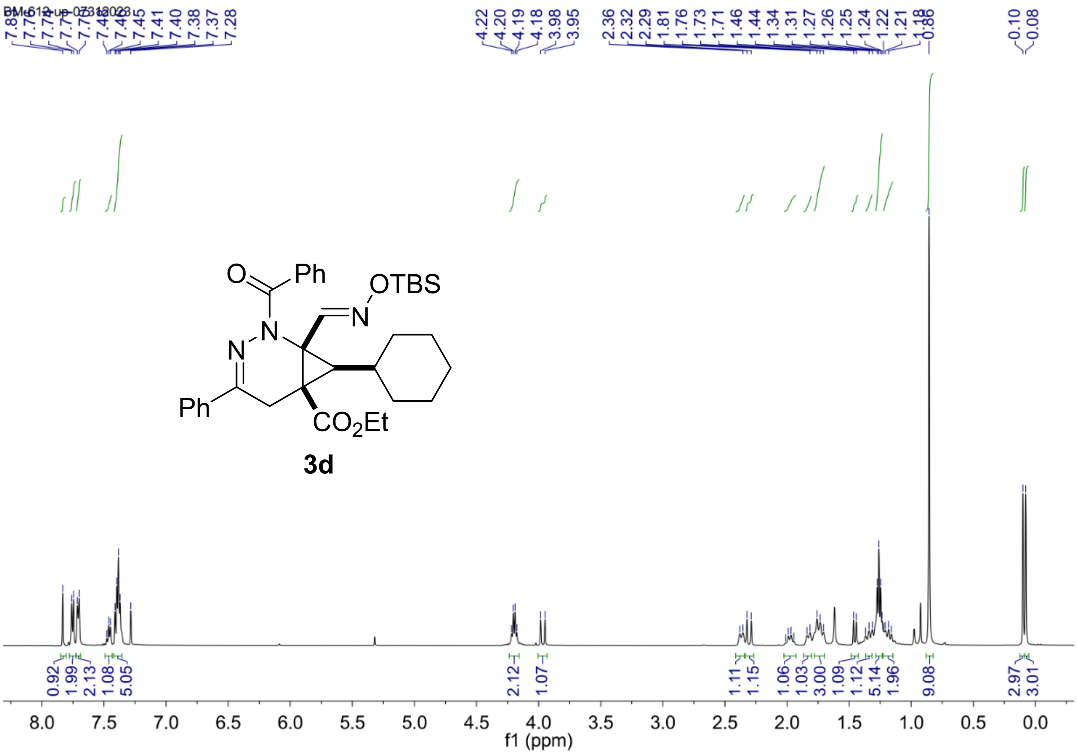

BM-612-up-07312023
O
Ph
OTBS
N
N
N
Ph
CO2Et
3d
f1 (ppm)

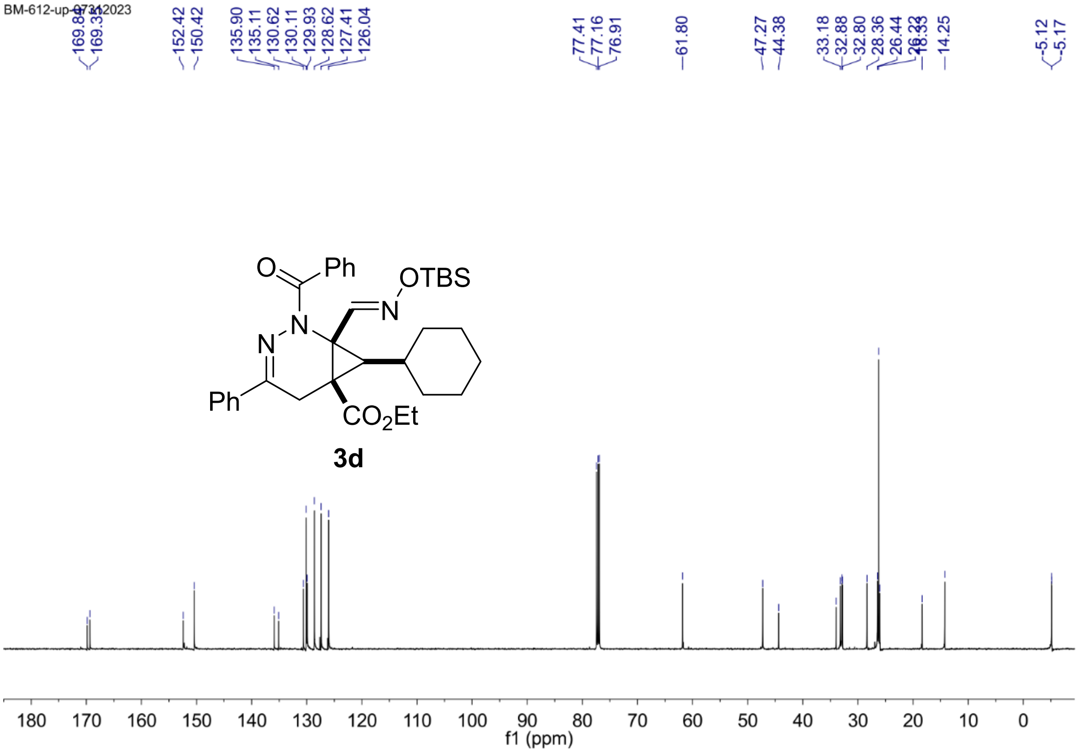

BM-612-up-07312023
O
Ph
OTBS
N
N
N
Ph
CO2Et
3d
f1 (ppm)

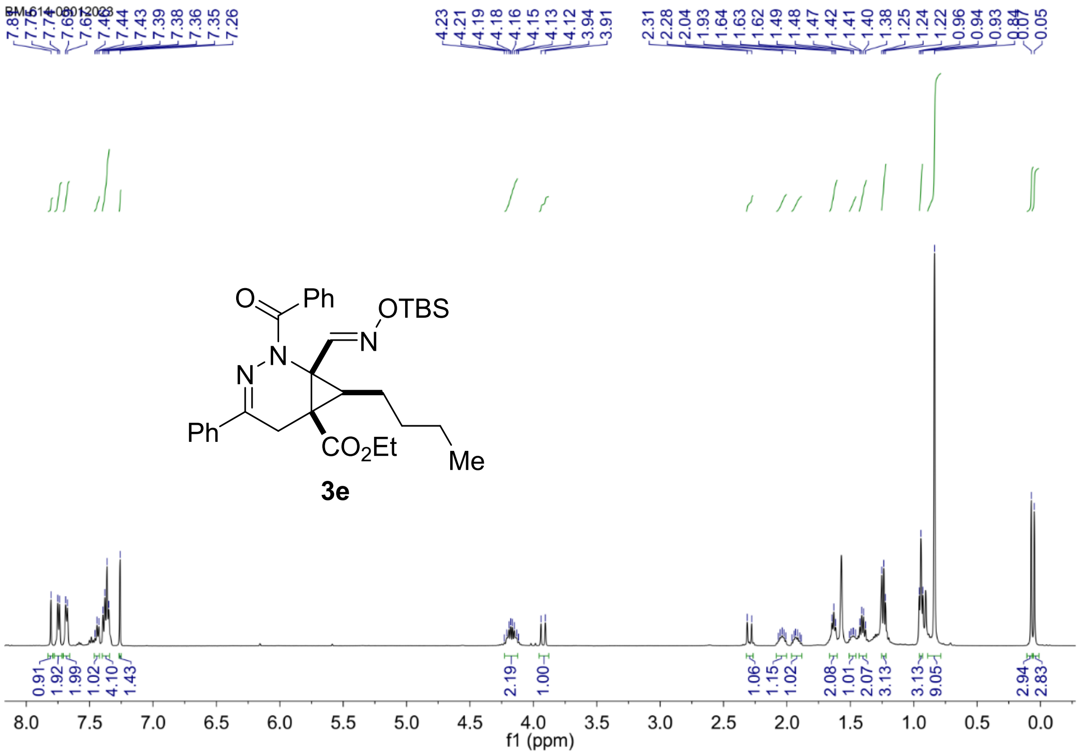

BM-614-08012023
4.23
4.21
4.19
4.18
4.16
4.15
4.13
4.12
3.94
3.91
2.31
2.28
2.04
1.93
1.64
1.63
1.62
1.49
1.48
1.47
1.42
1.41
1.40
1.38
1.25
1.24
1.22
0.96
0.94
0.93
0.05
O
Ph
OTBS
N
N
N
Ph
CO2Et
Me
3e
0.91
1.92
1.99
1.02
4.10
1.43
2.19
1.00
1.06
1.15
1.02
2.08
1.01
2.07
3.13
3.13
9.05
2.94
2.83
f1 (ppm)

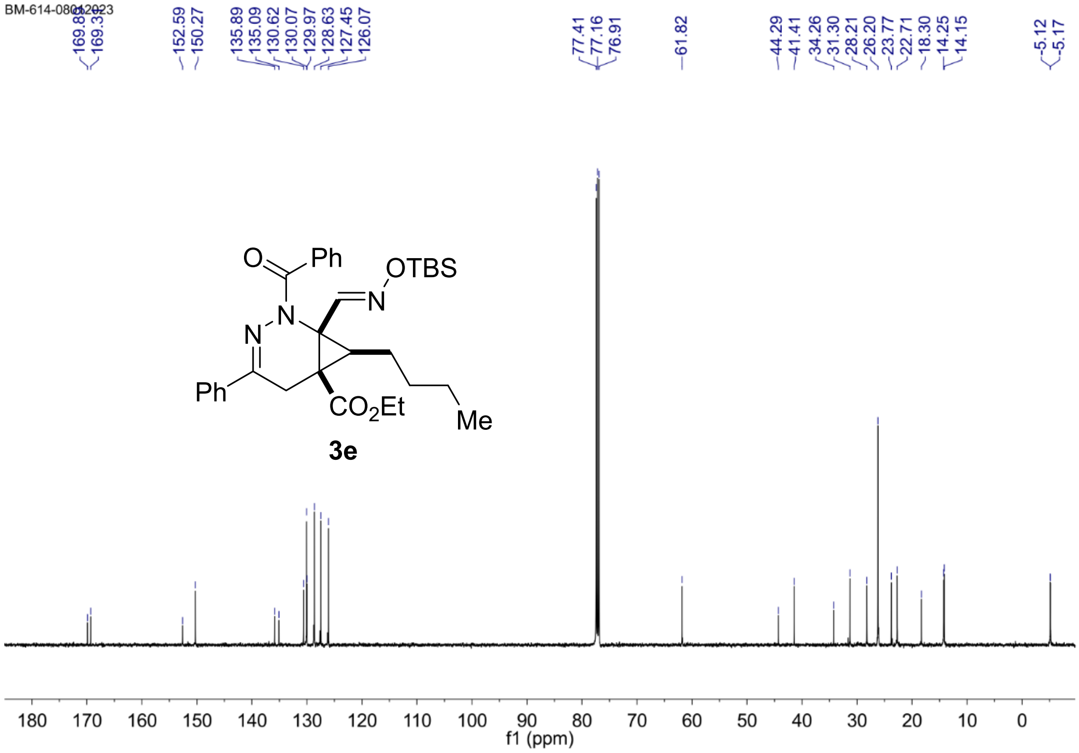

152.59
150.27
135.89
135.09
130.62
130.07
129.97
128.63
127.45
126.07
77.41
77.16
76.91
61.82
44.29
41.41
34.26
31.30
28.21
26.20
23.77
22.71
18.30
14.25
14.15
-5.12
-5.17
O
Ph
OTBS
N
N
N
Ph
CO2Et
Me
3e
f1 (ppm)

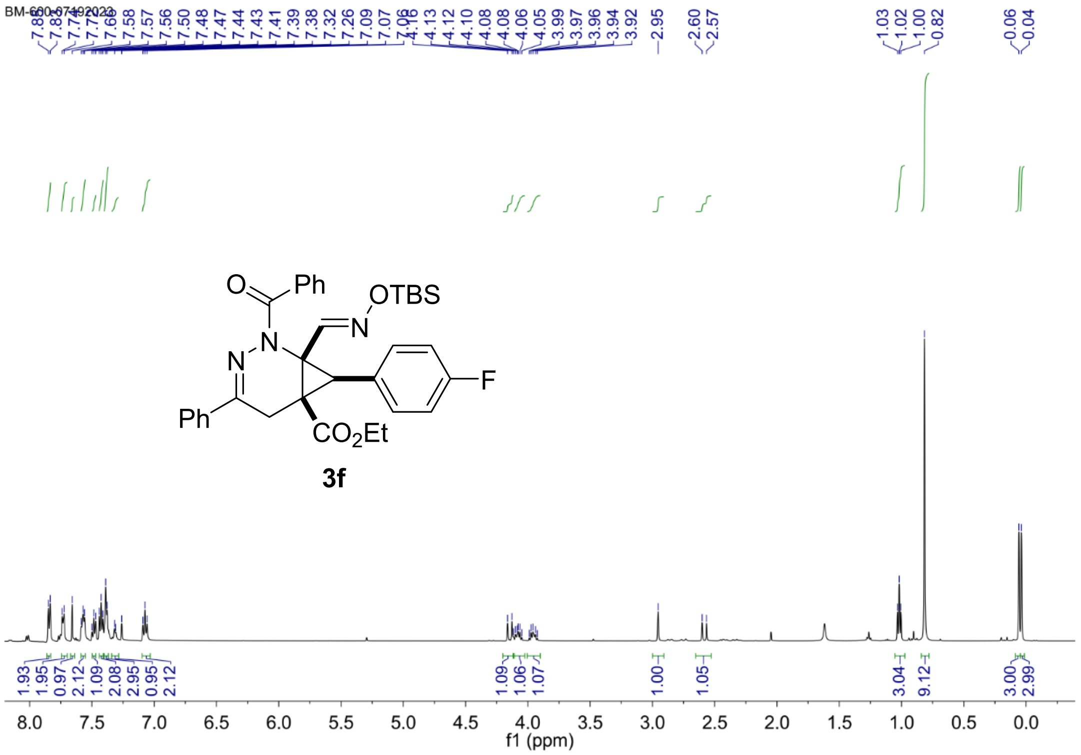

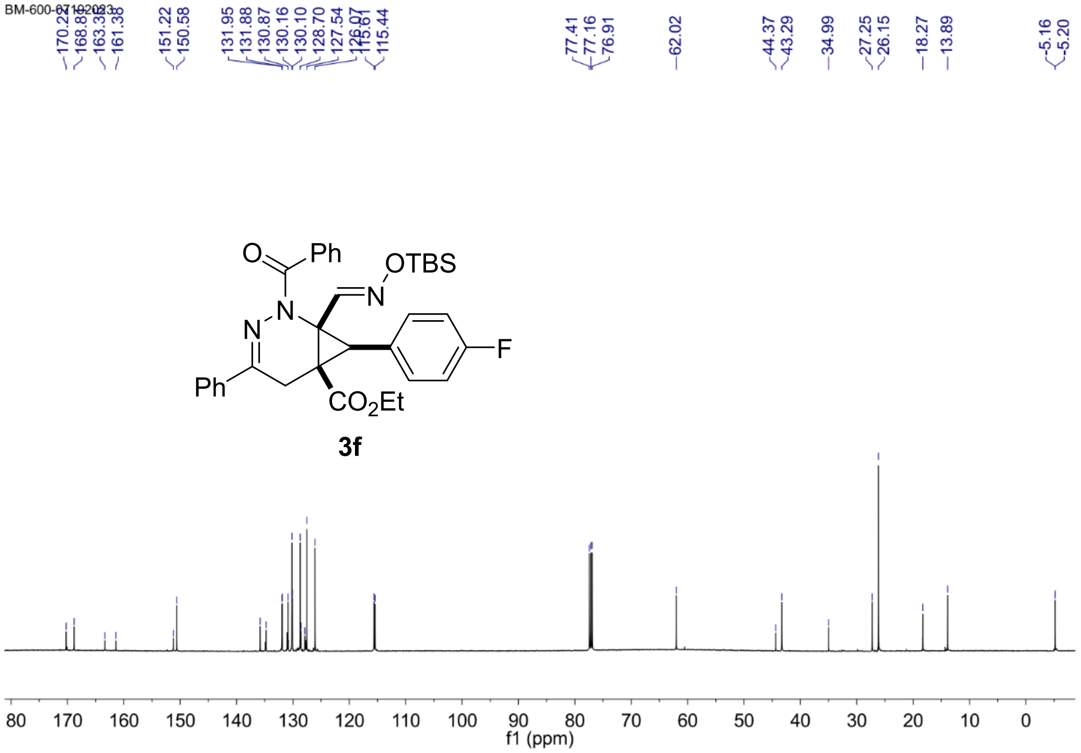

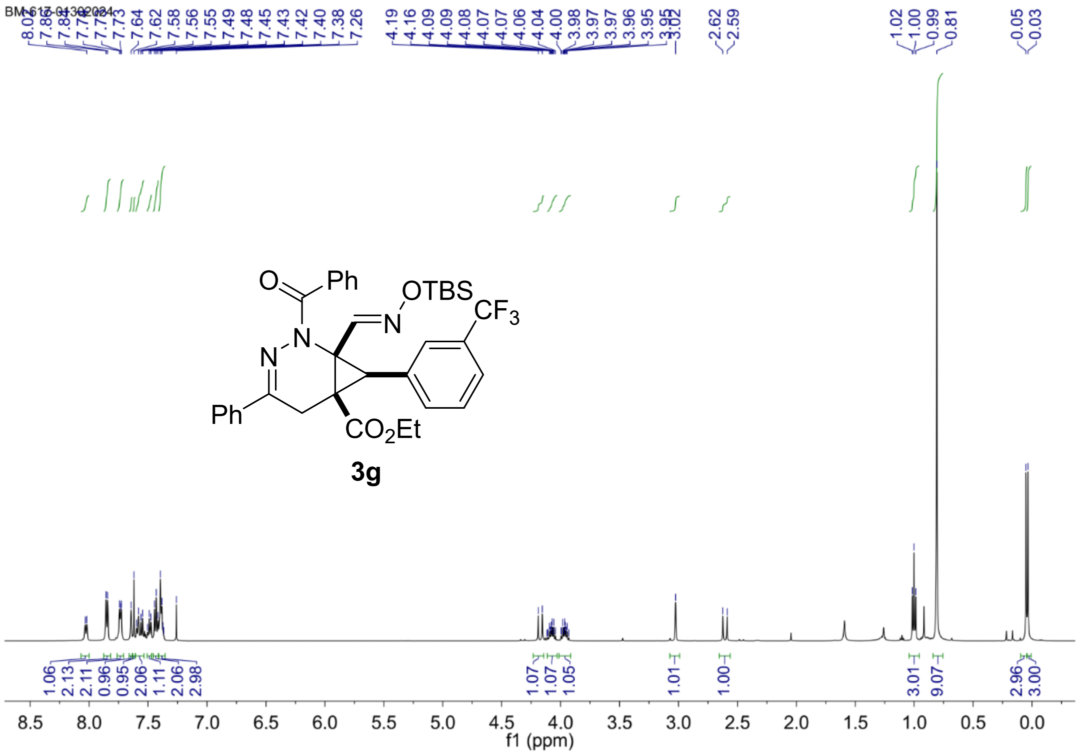
BM-617-01302024
7.73 7.64 7.62 7.58 7.56 7.55 7.49 7.48 7.45 7.43 7.42 7.40 7.38 7.26
4.19 4.16 4.09 4.09 4.08 4.07 4.07 4.06 4.04 4.00 3.98 3.97 3.97 3.96 3.95
3.02
2.62 2.59
1.02 1.00 0.99 0.81
0.05 0.03
O Ph OTBS N CF3 N N Ph CO2Et
3g
1.06 2.13 2.11 0.96 0.95 2.06 1.11 2.06 2.98 1.07 1.07 1.05 1.01 1.00 3.01 9.07 2.96 3.00
8.5 8.0 7.5 7.0 6.5 6.0 5.5 5.0 4.5 4.0 3.5 3.0 2.5 2.0 1.5 1.0 0.5 0.0
f1 (ppm)

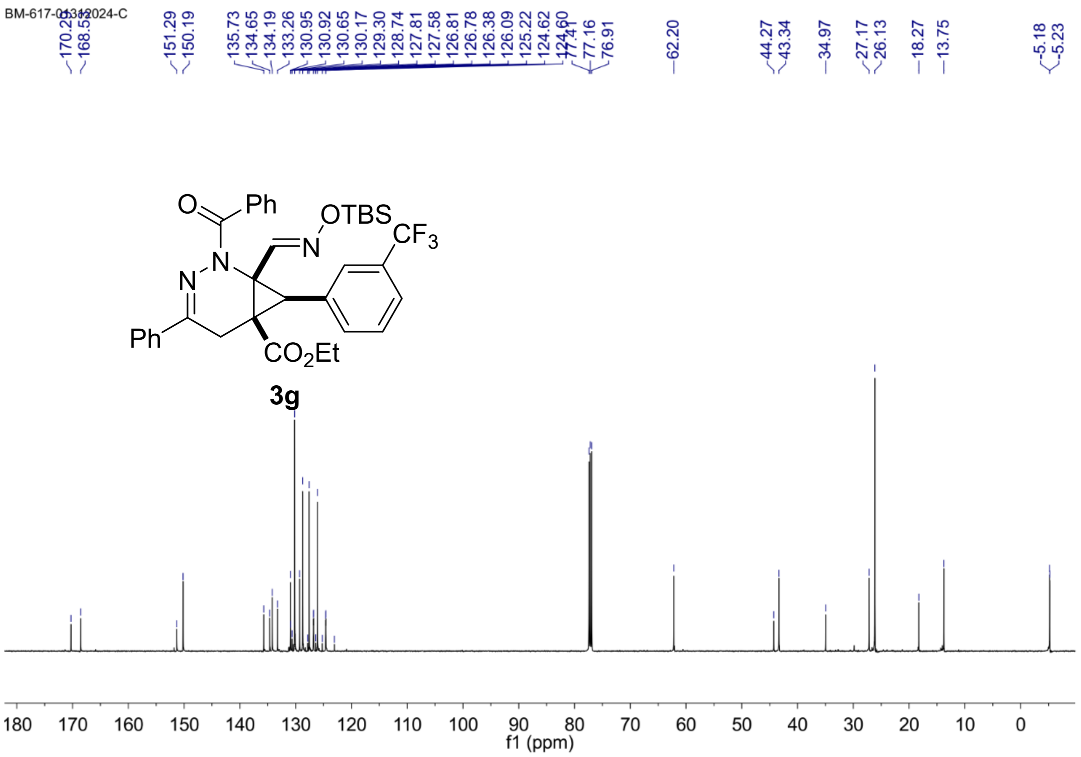
BM-617-01312024-C
151.29 150.19
135.73 134.65 134.19 133.26 130.95 130.92 130.65 130.17 129.30 128.74 127.81 127.58 126.81 126.78 126.38 126.09 125.22 124.62
77.41 77.16 76.91
62.20
44.27 43.34
34.97
27.17 26.13
18.27
13.75
-5.18 -5.23
O Ph OTBS N CF3 N N Ph CO2Et
3g
180 170 160 150 140 130 120 110 100 90 80 70 60 50 40 30 20 10 0
f1 (ppm)

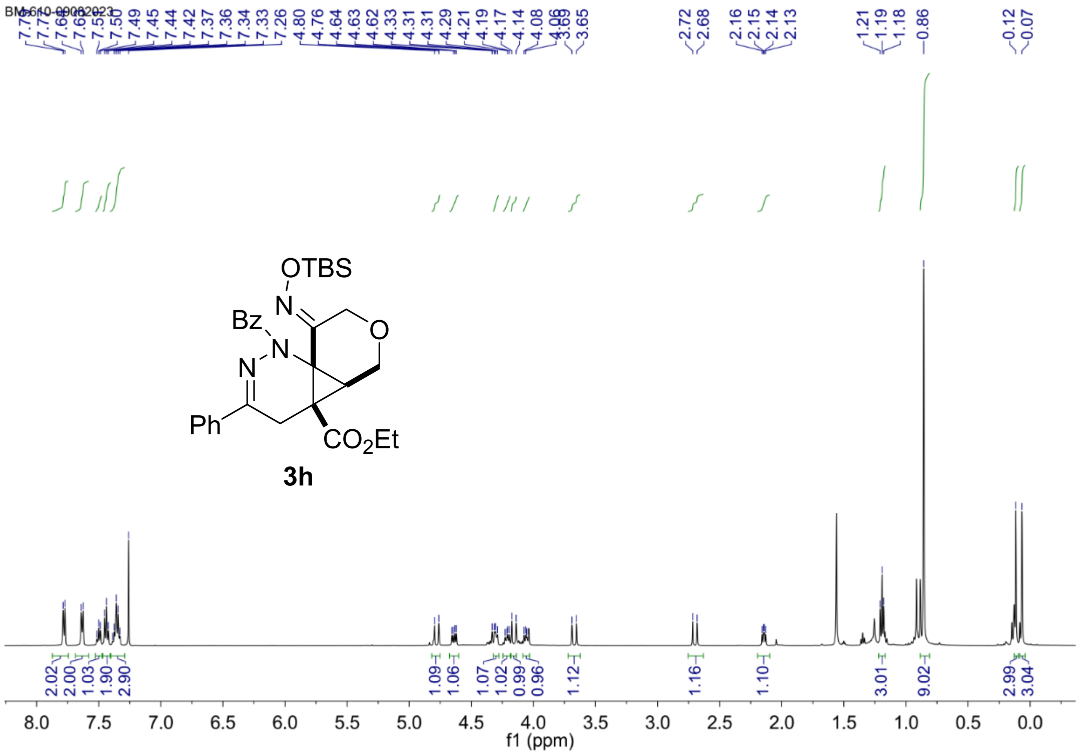

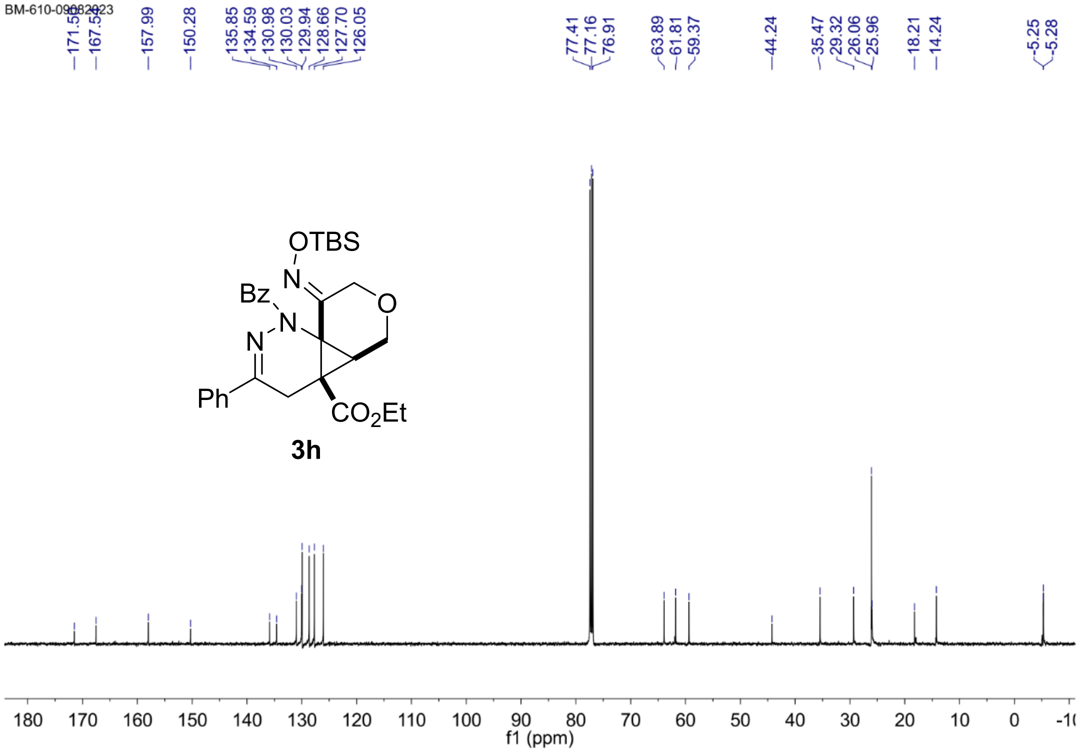

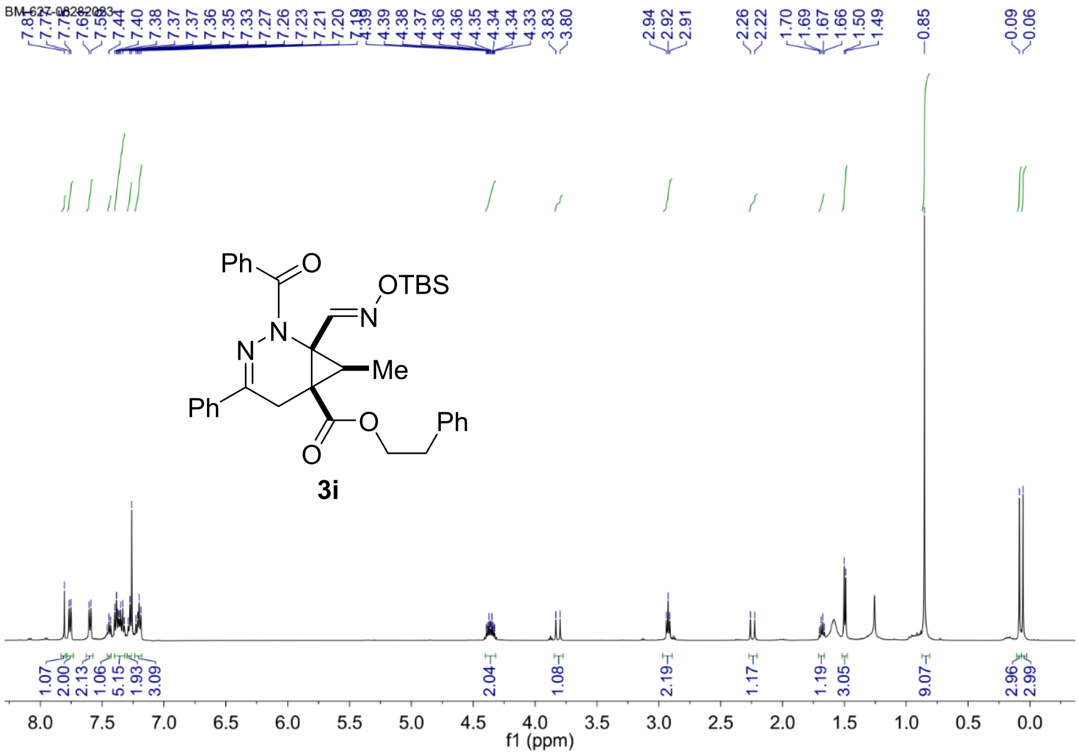
BM-627-08282023
Ph
O
OTBS
N
N
N
Me
Ph
O
Ph
O
3i
f1 (ppm)

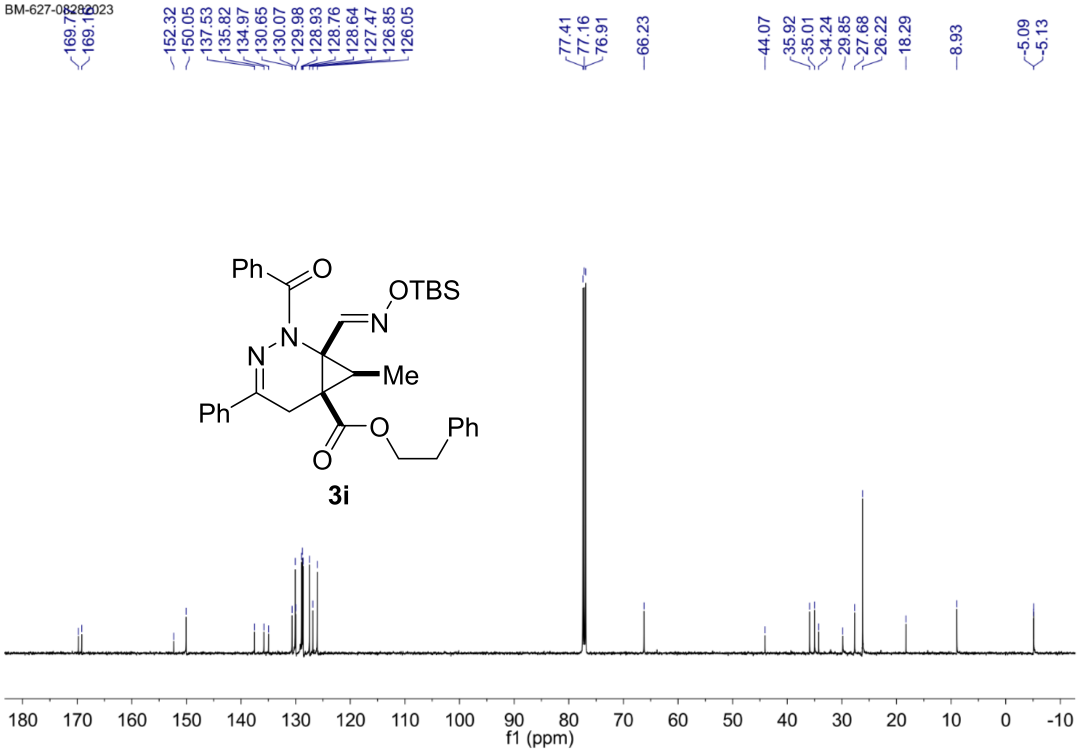
BM-627-08282023
Ph
O
OTBS
N
N
N
Me
Ph
O
Ph
O
3i
f1 (ppm)

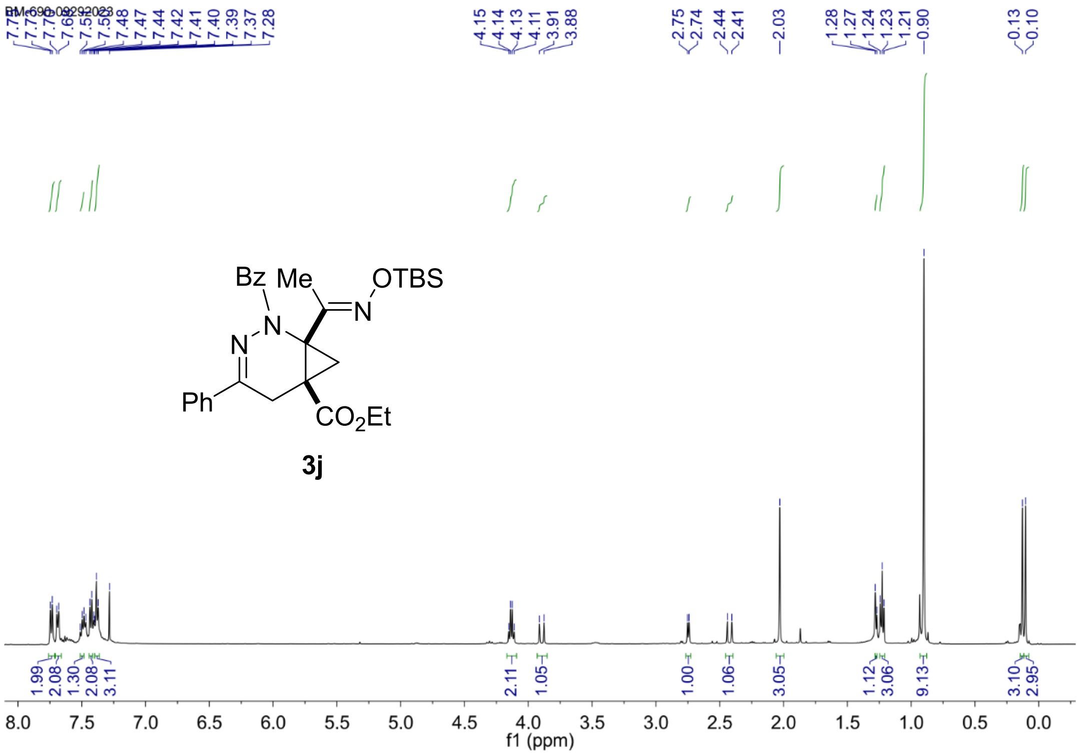

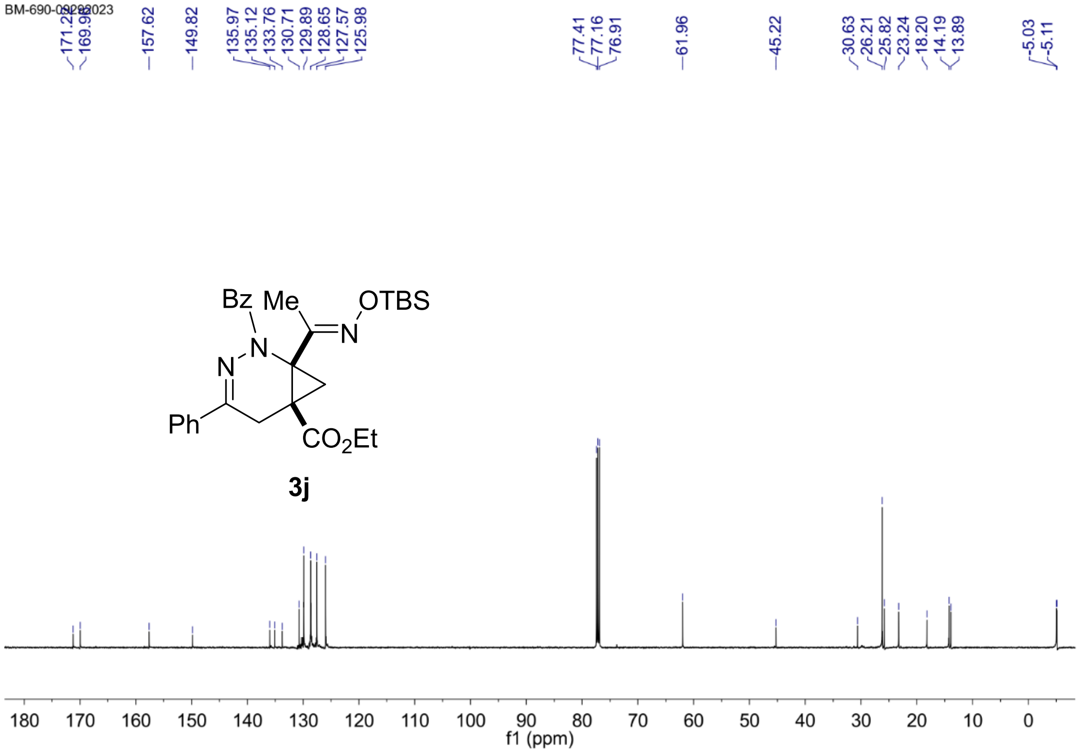

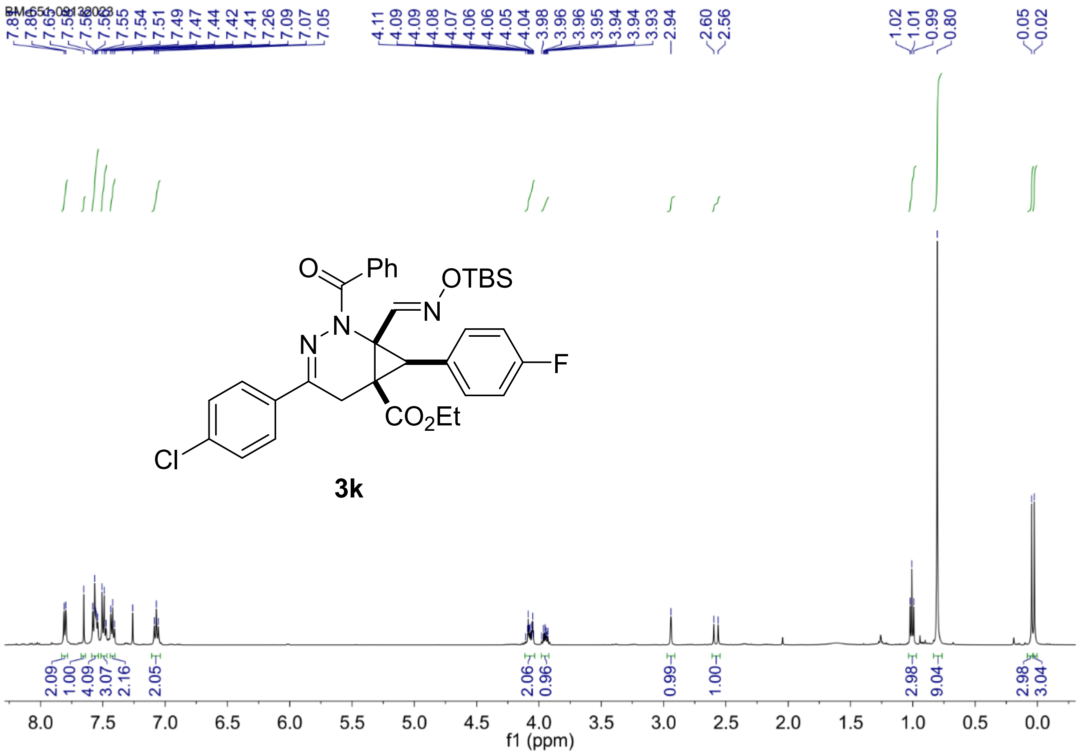
BM-651-09132023
O
Ph
OTBS
N
N
N
F
CO2Et
Cl
3k
f1 (ppm)

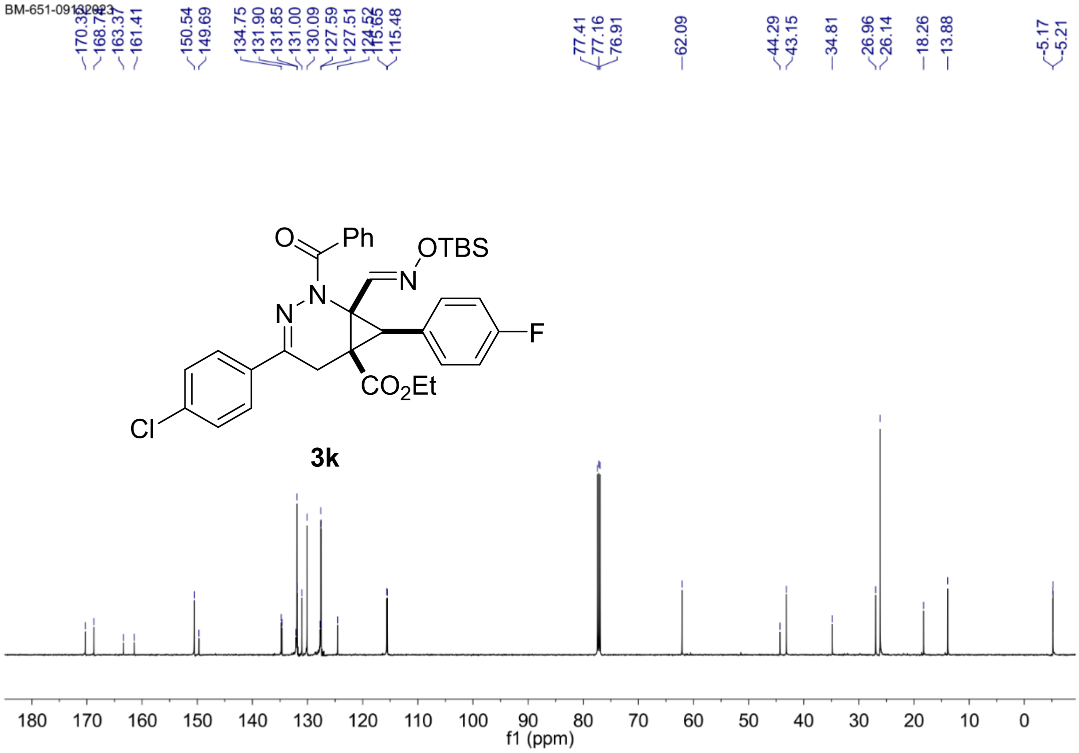
BM-651-09132023
O
Ph
OTBS
N
N
N
F
CO2Et
Cl
3k
f1 (ppm)

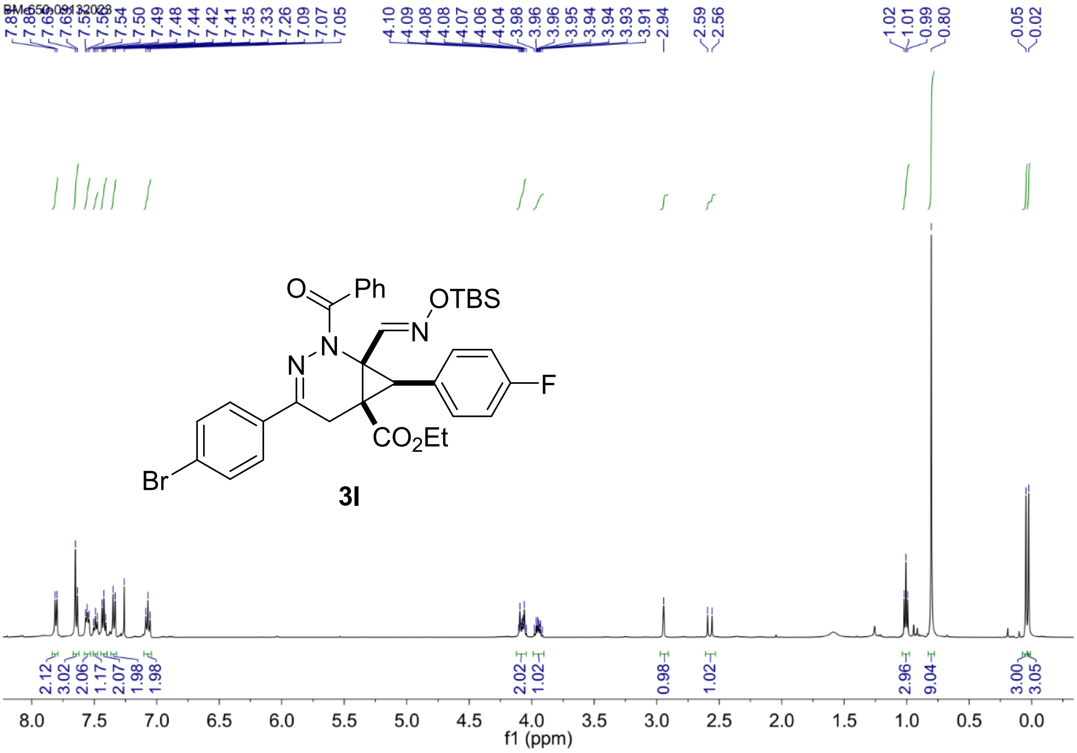

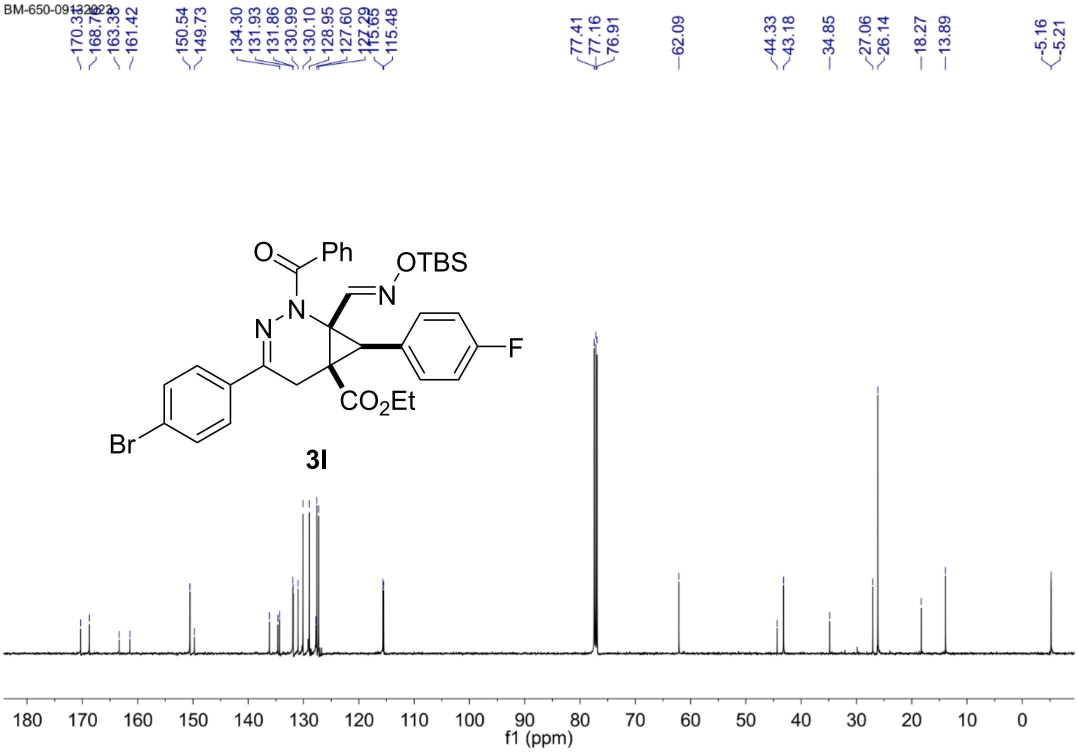

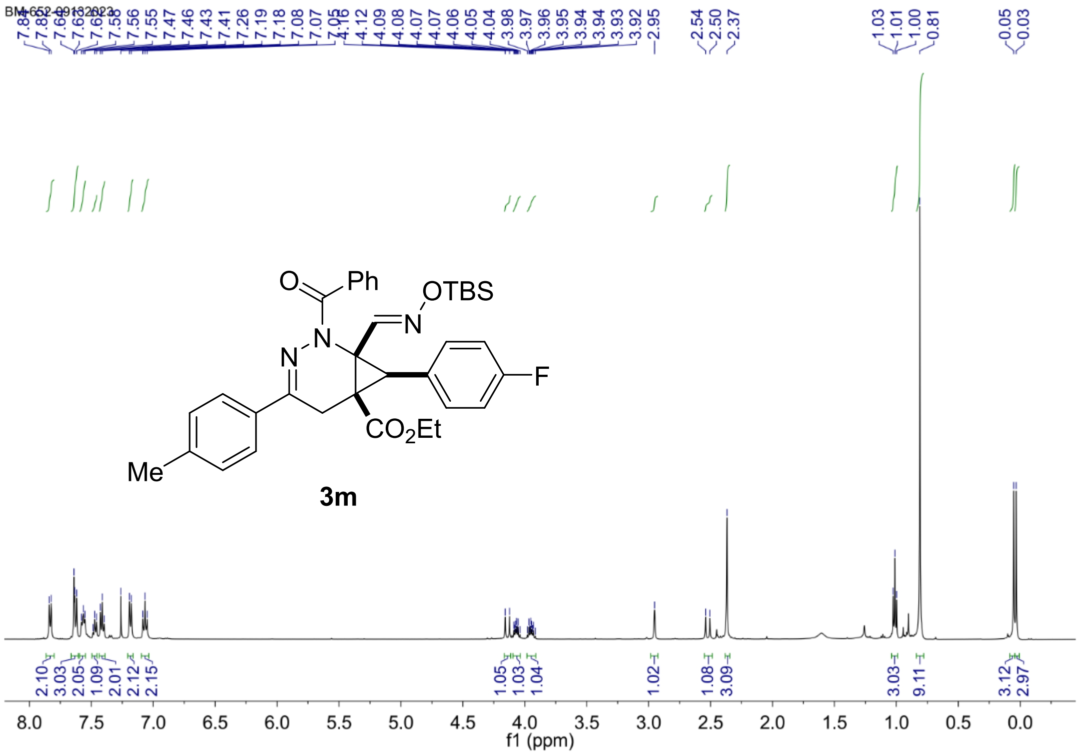

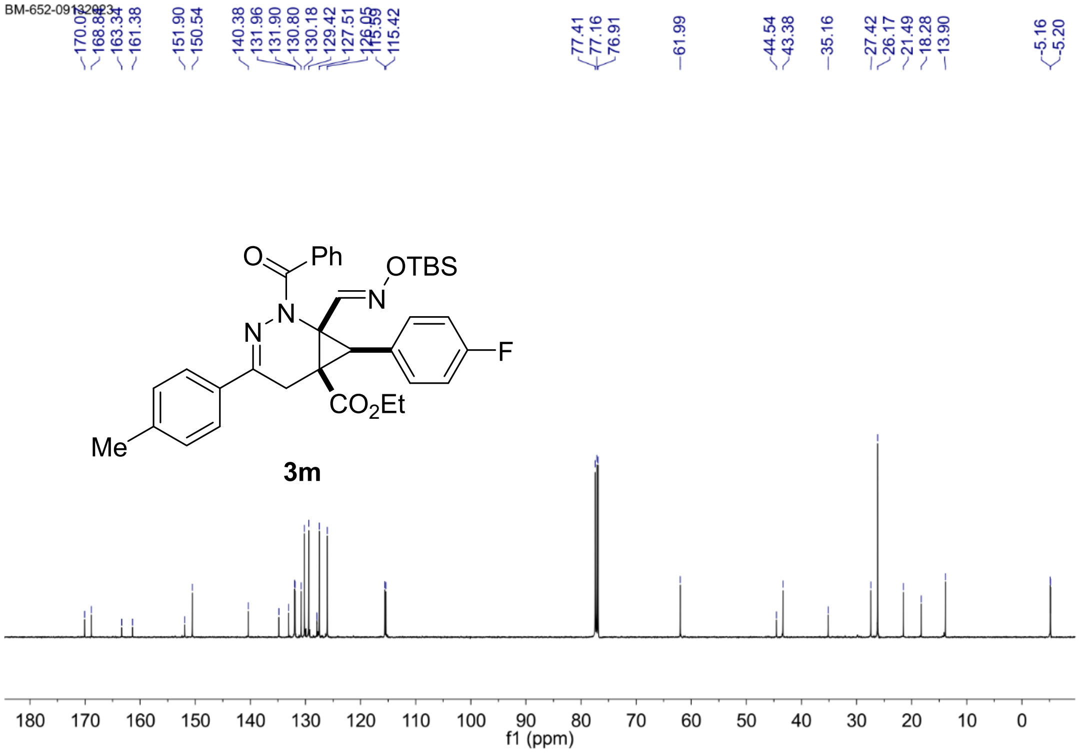

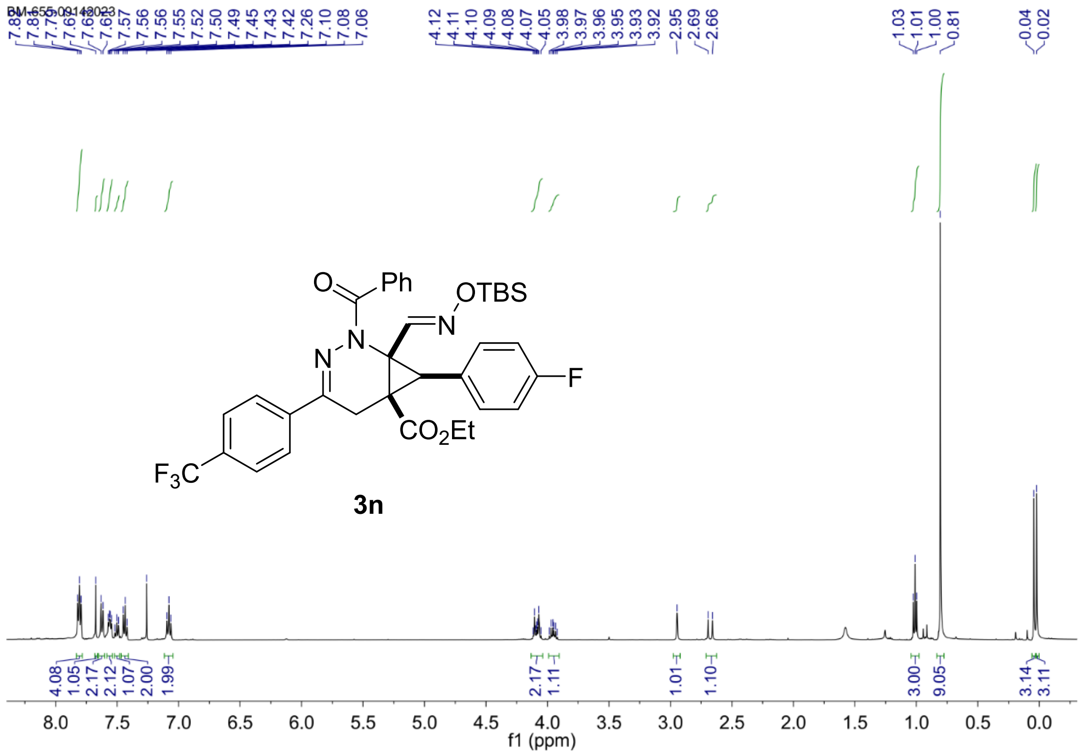

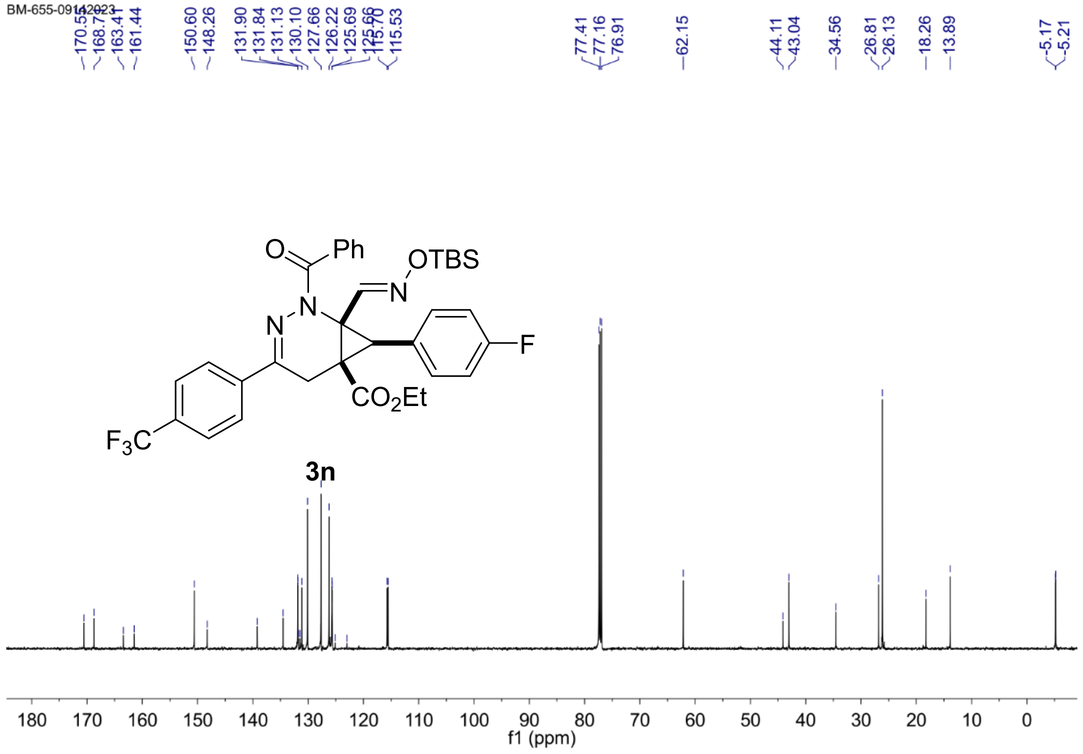

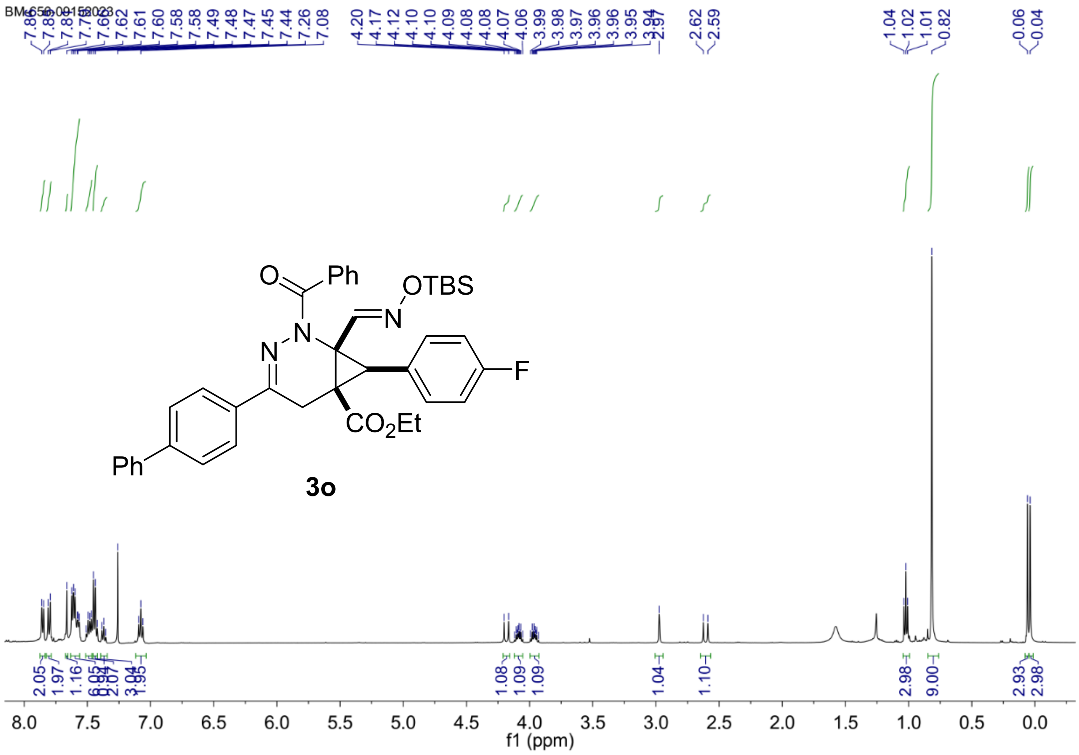

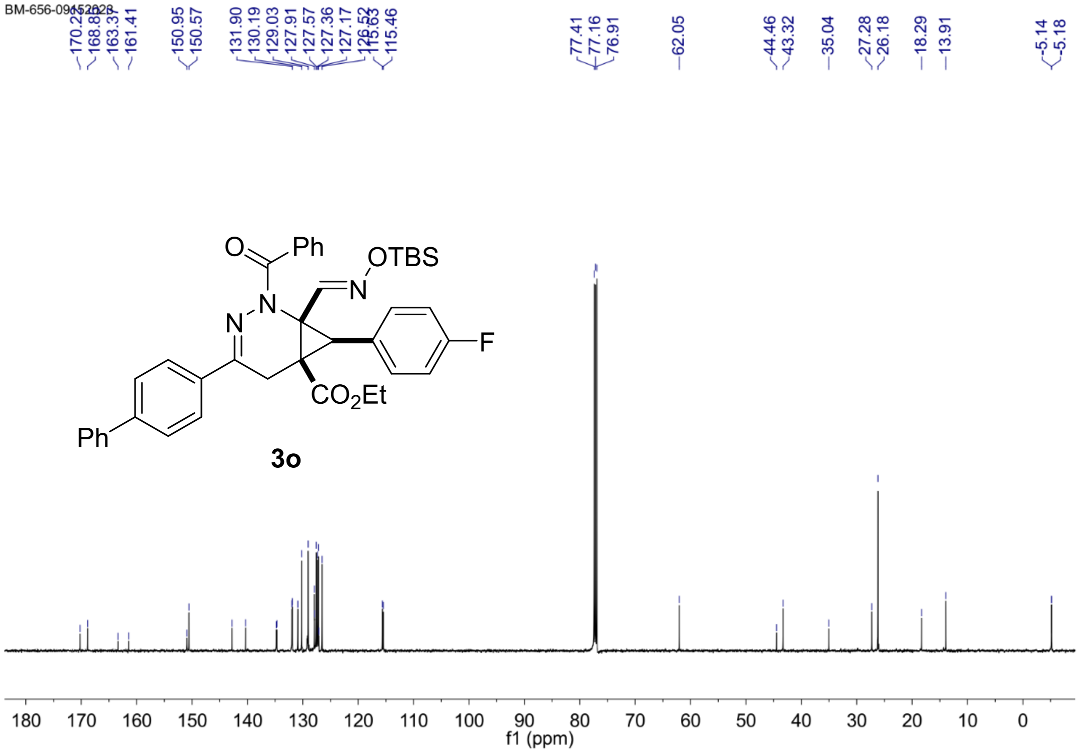

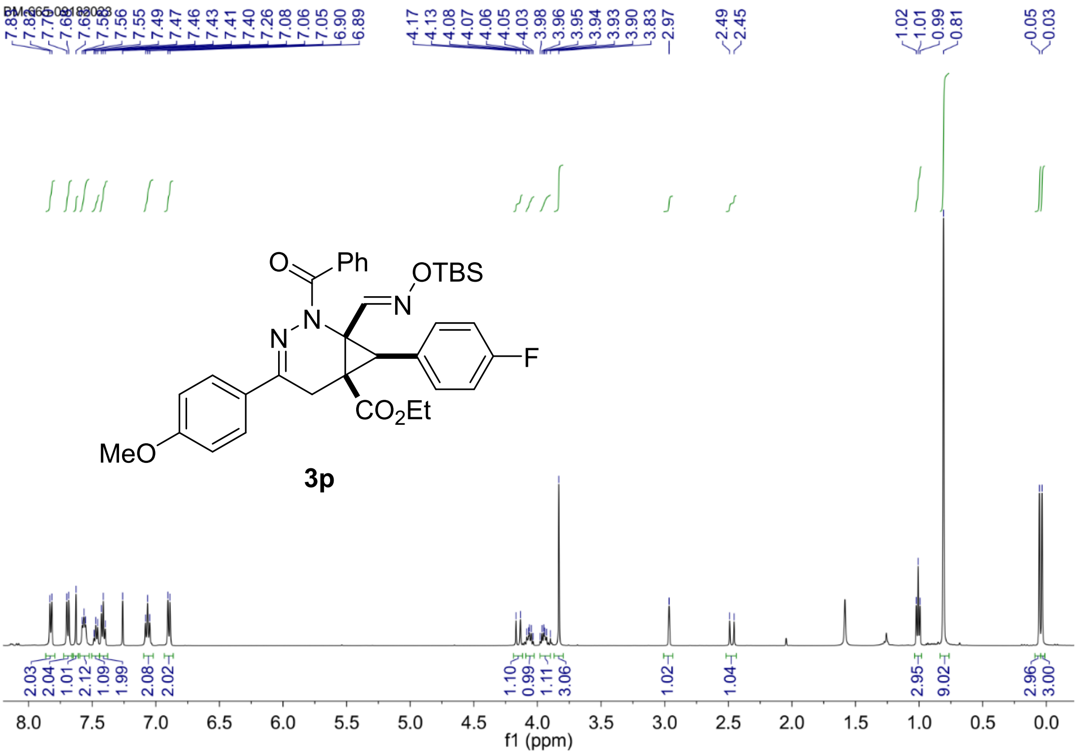
3p

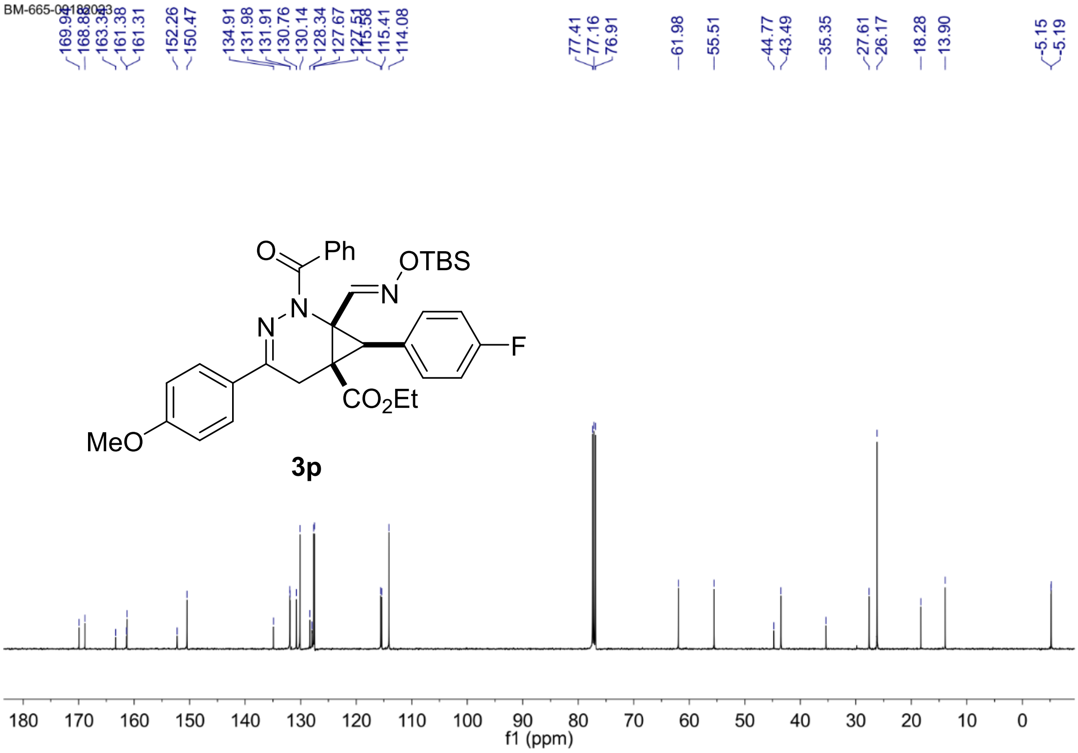
3p

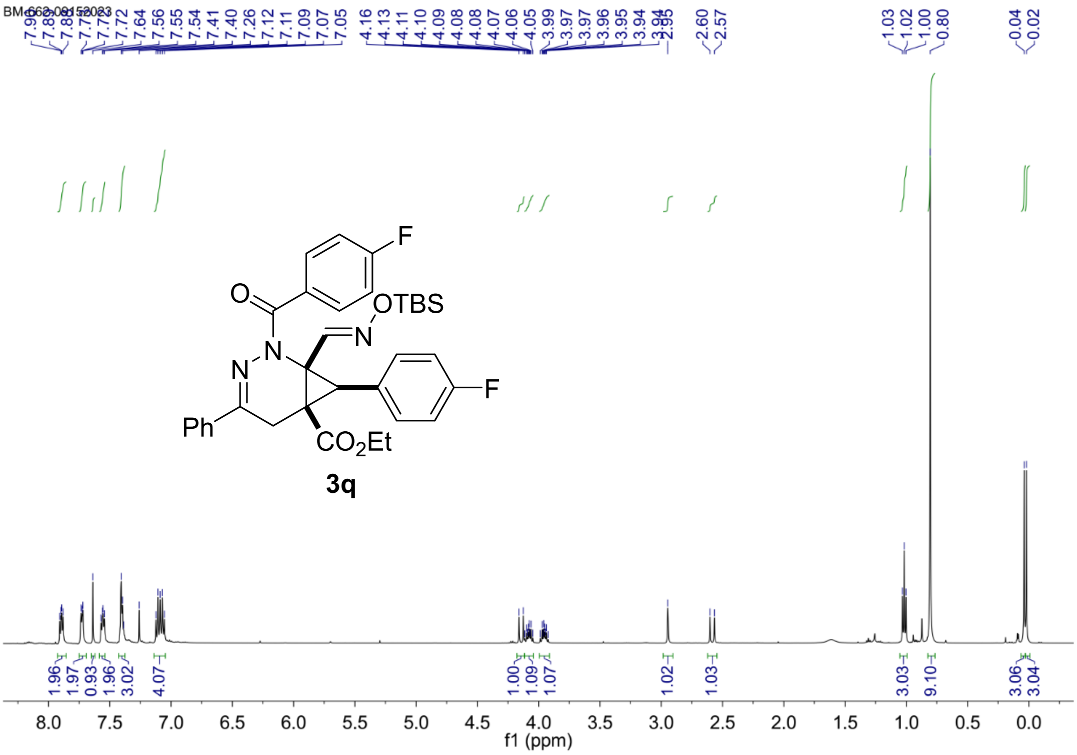
OTBS
F
O
N
N
Ph
CO2Et
3q
1.96
1.97
0.93
1.96
3.02
4.07
1.00
1.09
1.07
1.02
1.03
3.03
9.10
3.06
3.04
f1 (ppm)

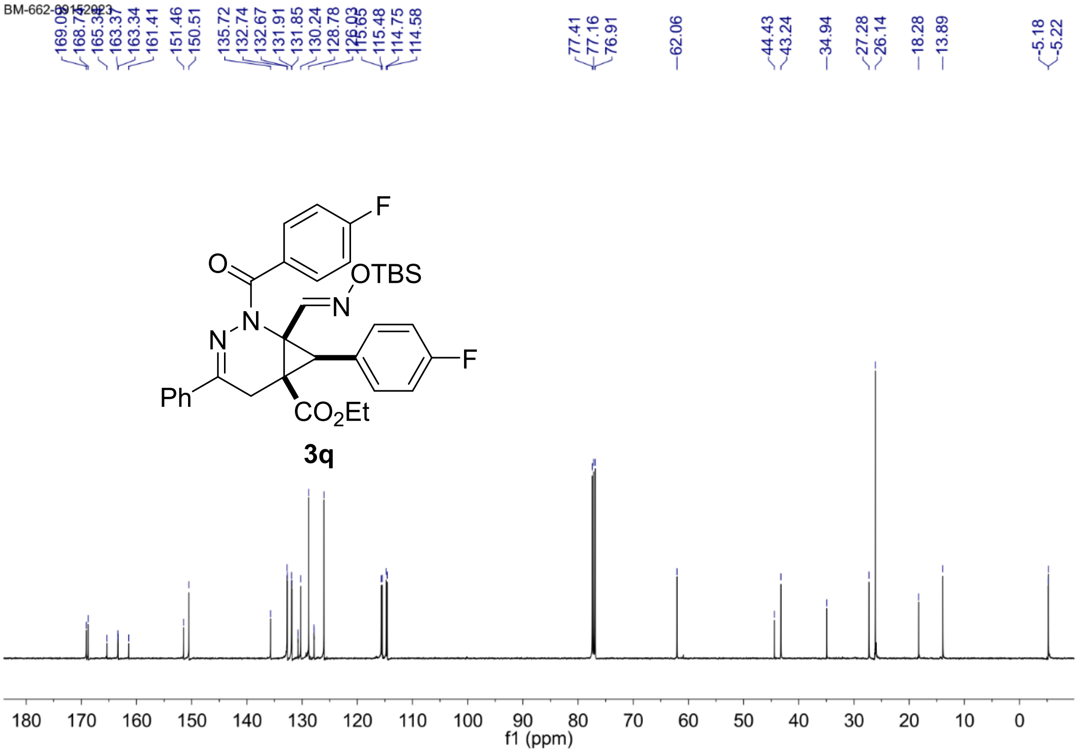
OTBS
F
O
N
N
Ph
CO2Et
3q
77.41
77.16
76.91
62.06
44.43
43.24
34.94
27.28
26.14
18.28
13.89
-5.18
-5.22
f1 (ppm)

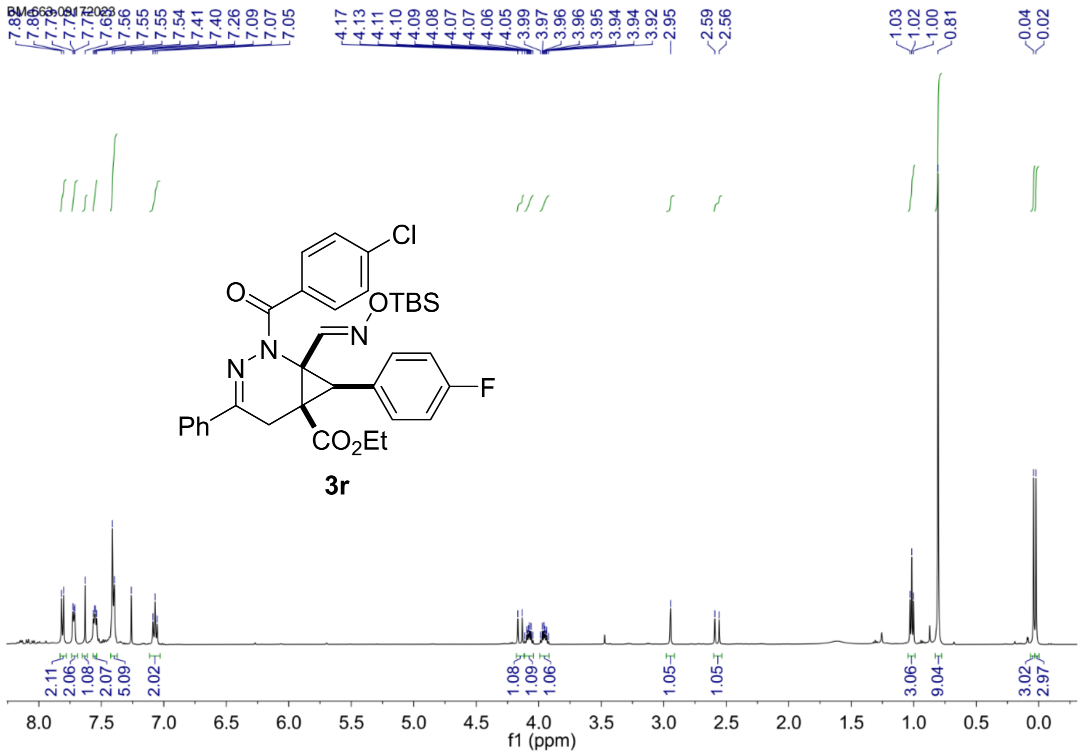
BM-663-09172023
OTBS
Cl
O
N
N
N
F
Ph
CO_2Et
3r
f1 (ppm)

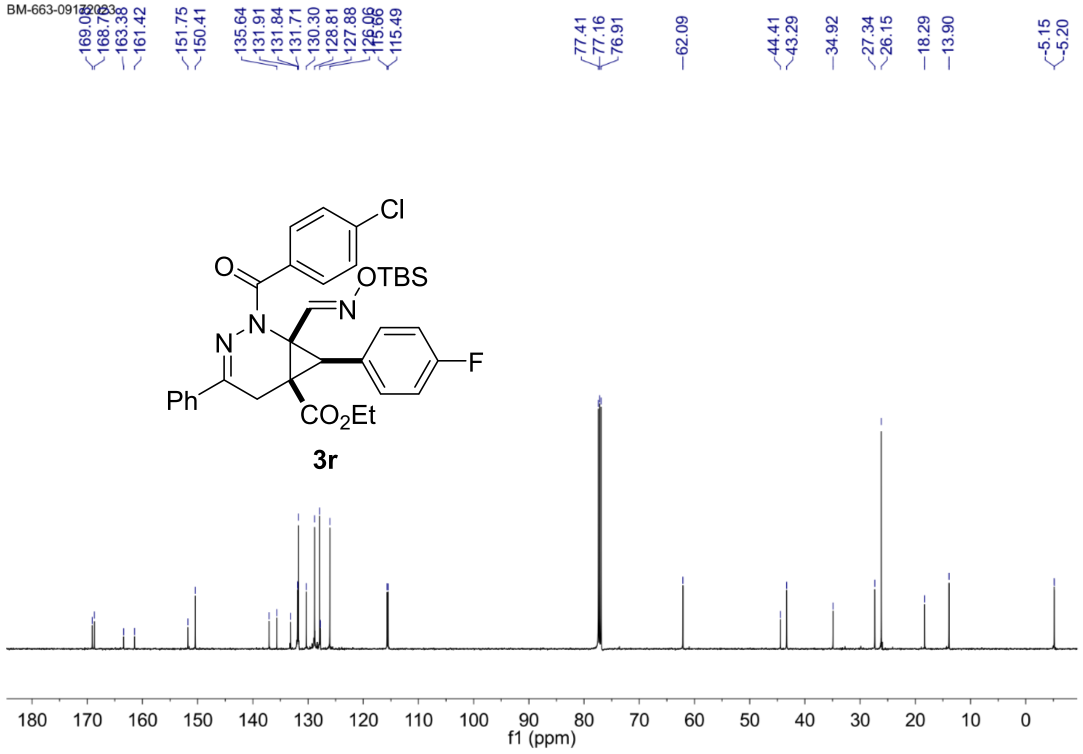
BM-663-09172023
OTBS
Cl
O
N
N
N
F
Ph
CO_2Et
3r
f1 (ppm)

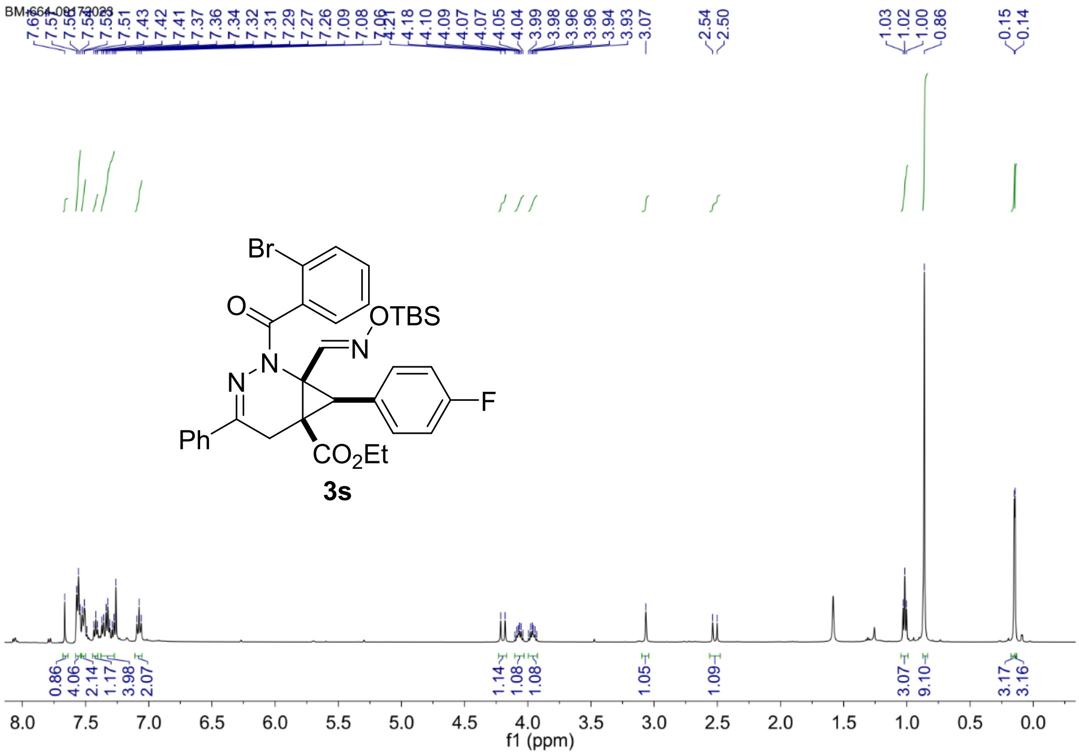

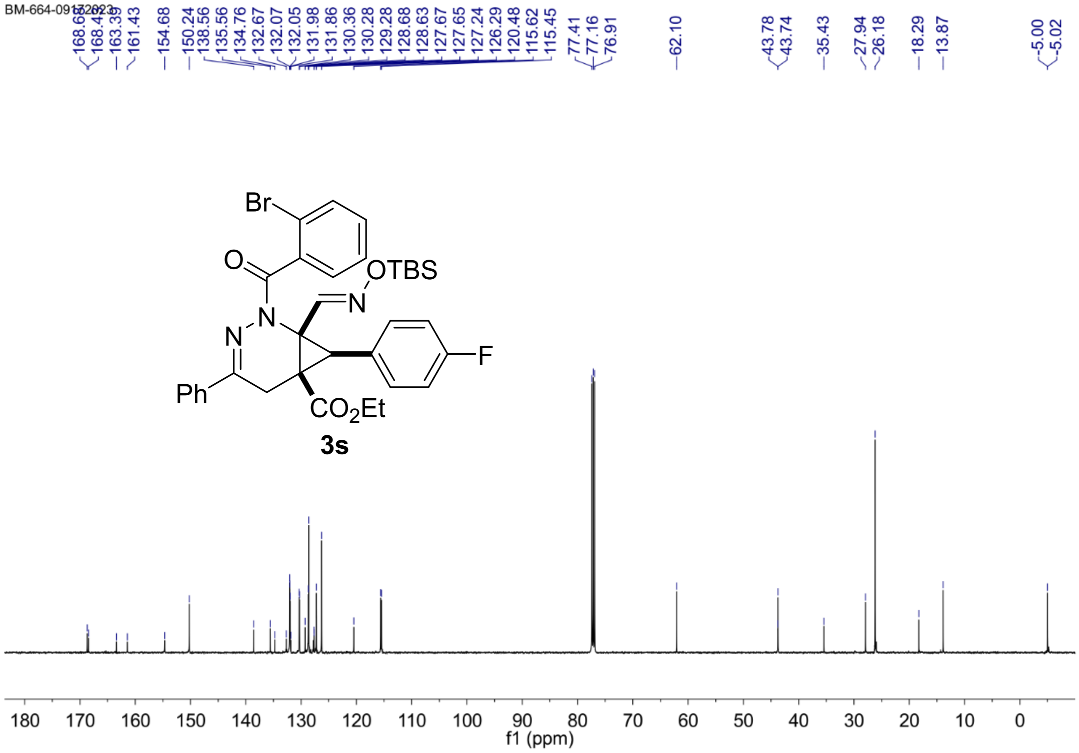

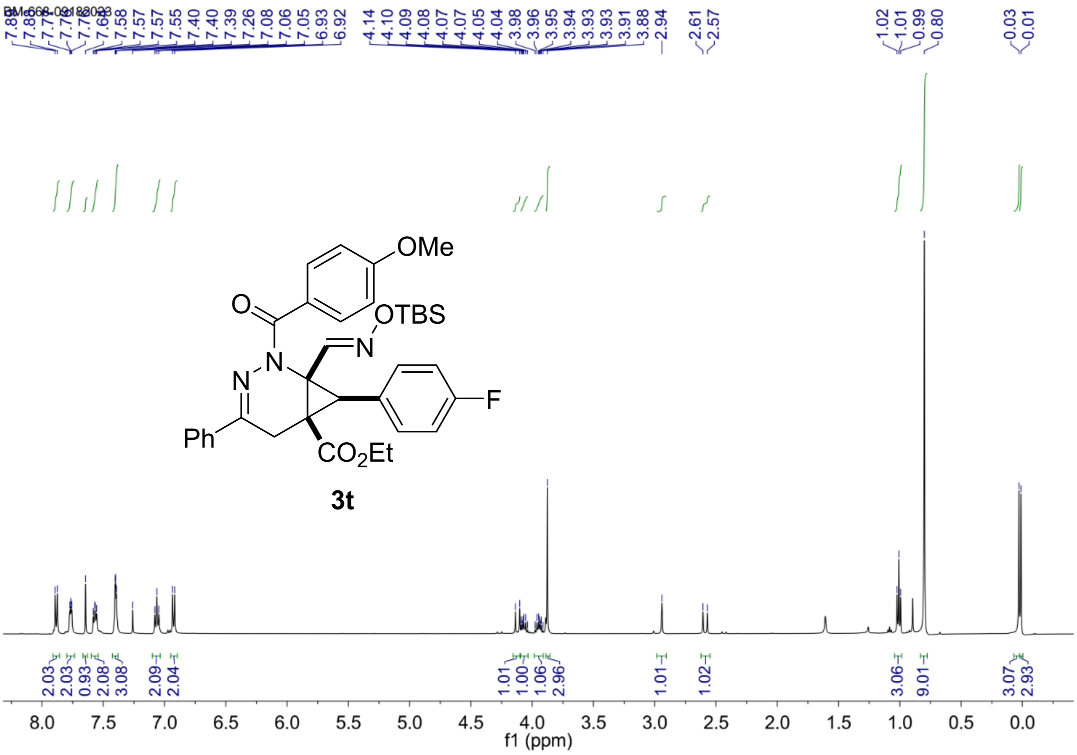
OMe
O
OTBS
N
N
N
F
Ph
CO2Et
3t
f1 (ppm)

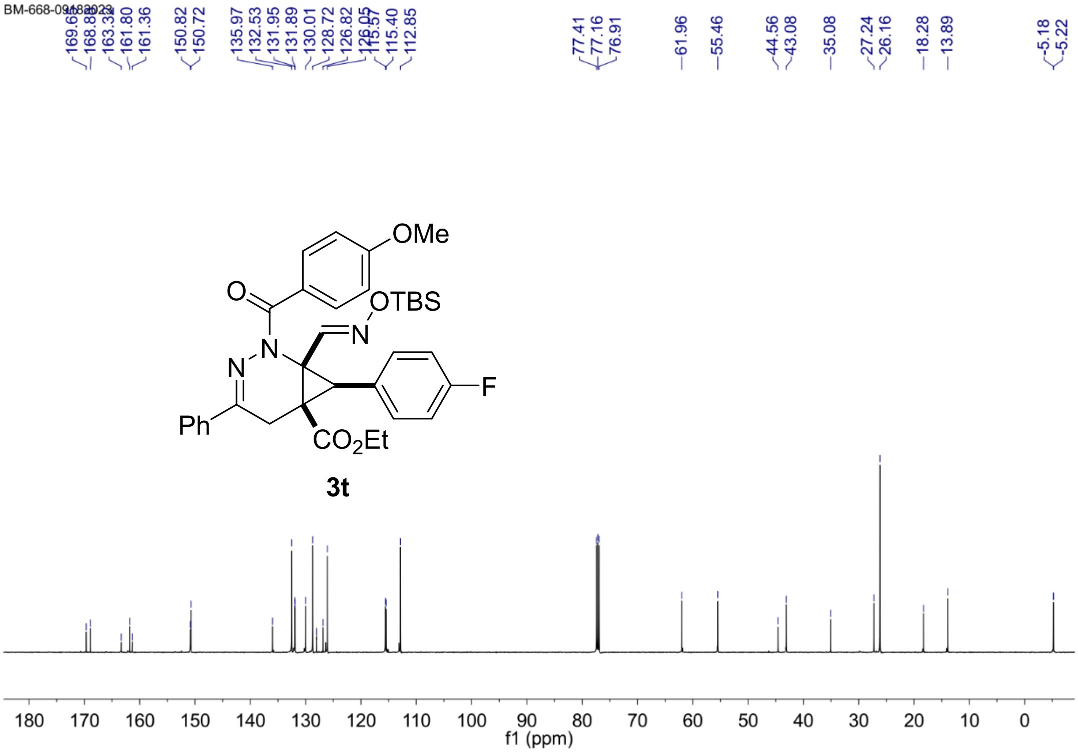
OMe
O
OTBS
N
N
N
F
Ph
CO2Et
3t
f1 (ppm)

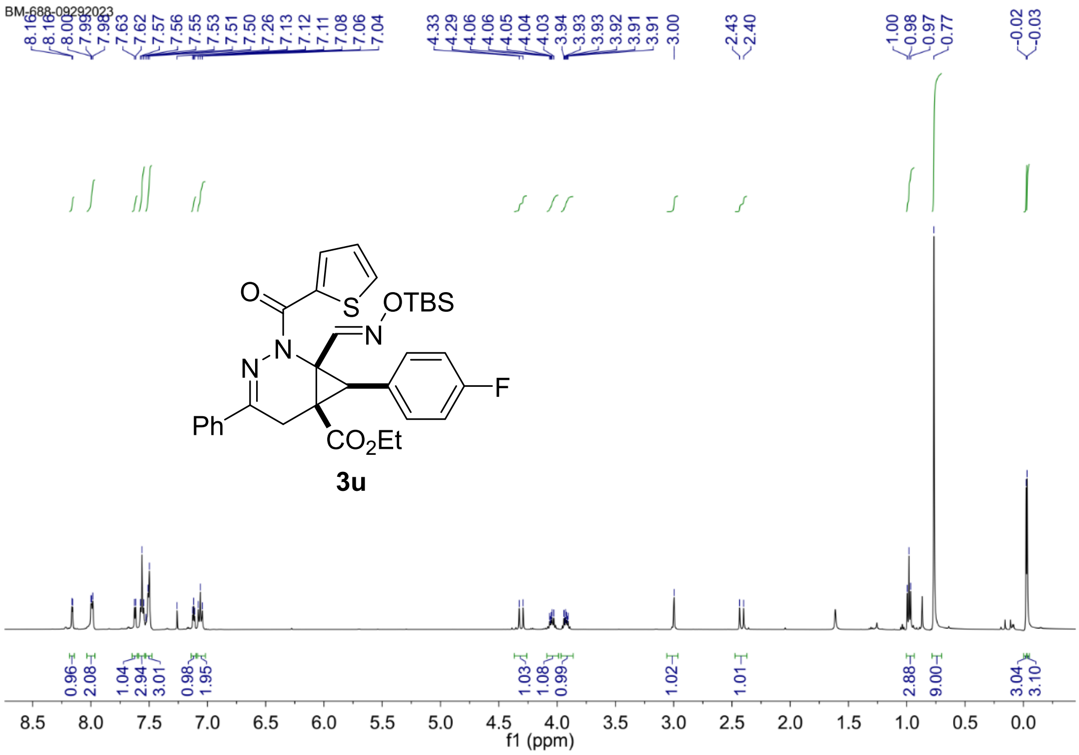

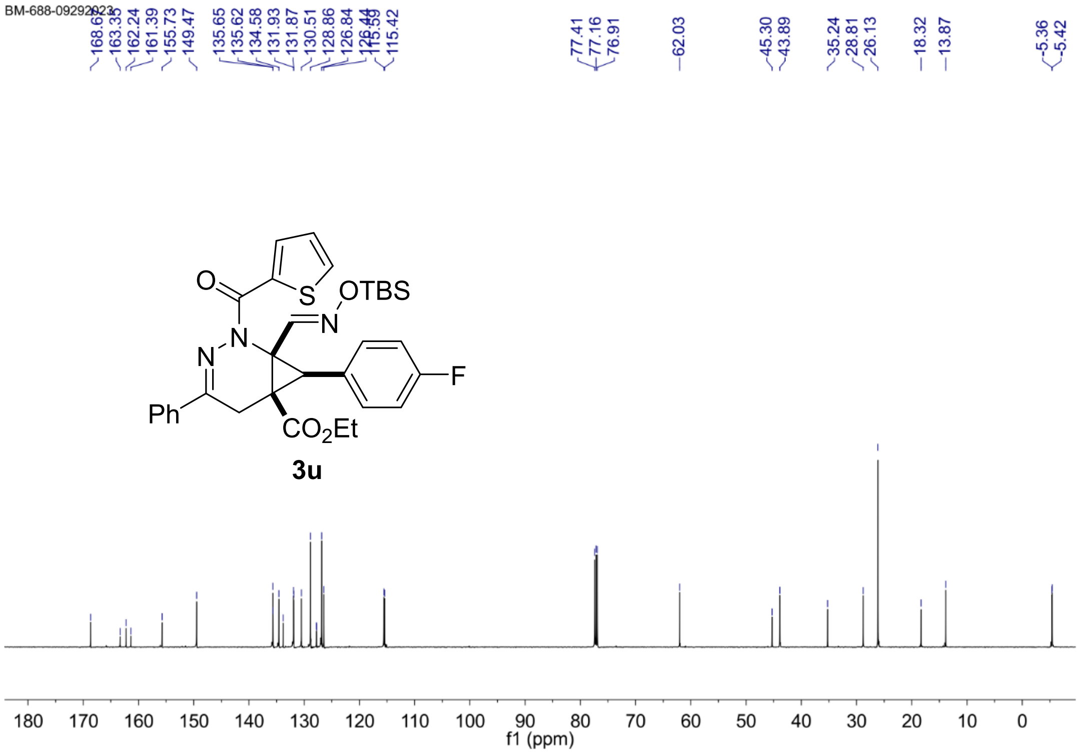

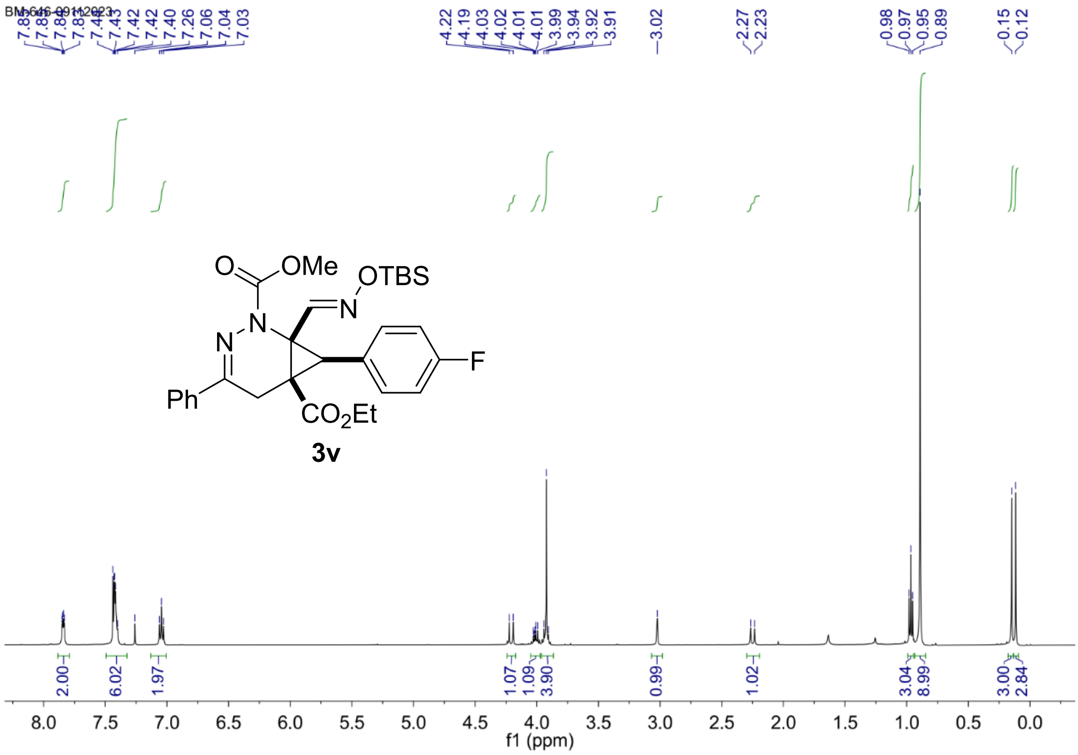

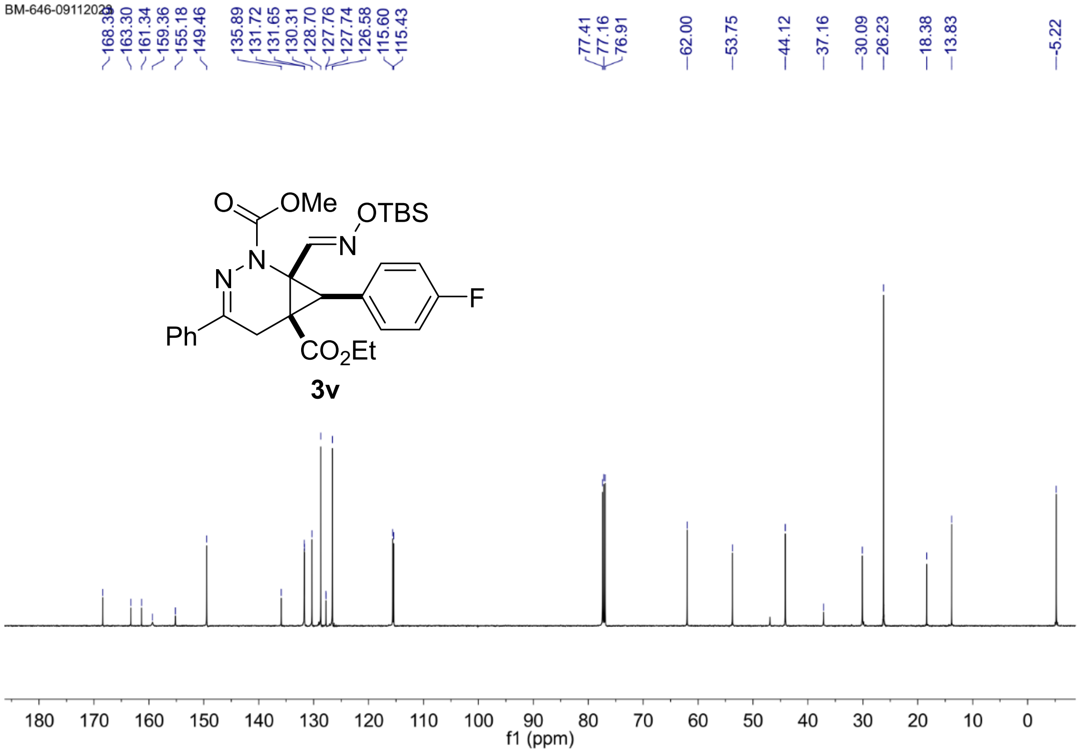

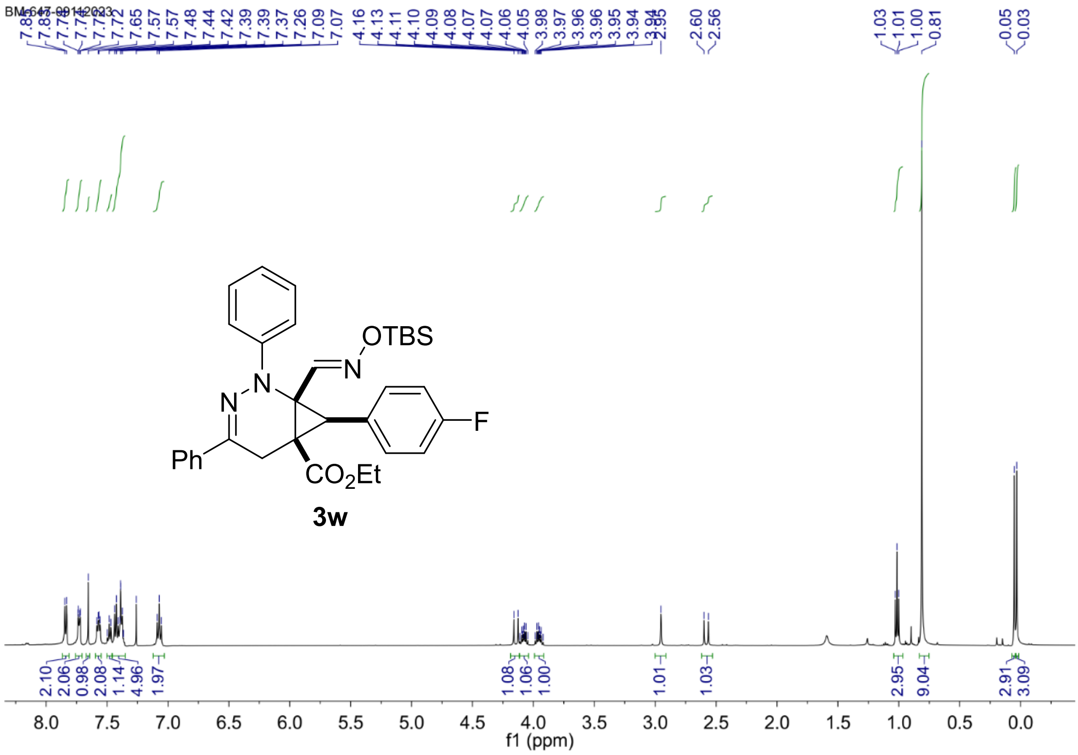
OTBS
N
N
N
F
Ph
CO2Et
3w
f1 (ppm)

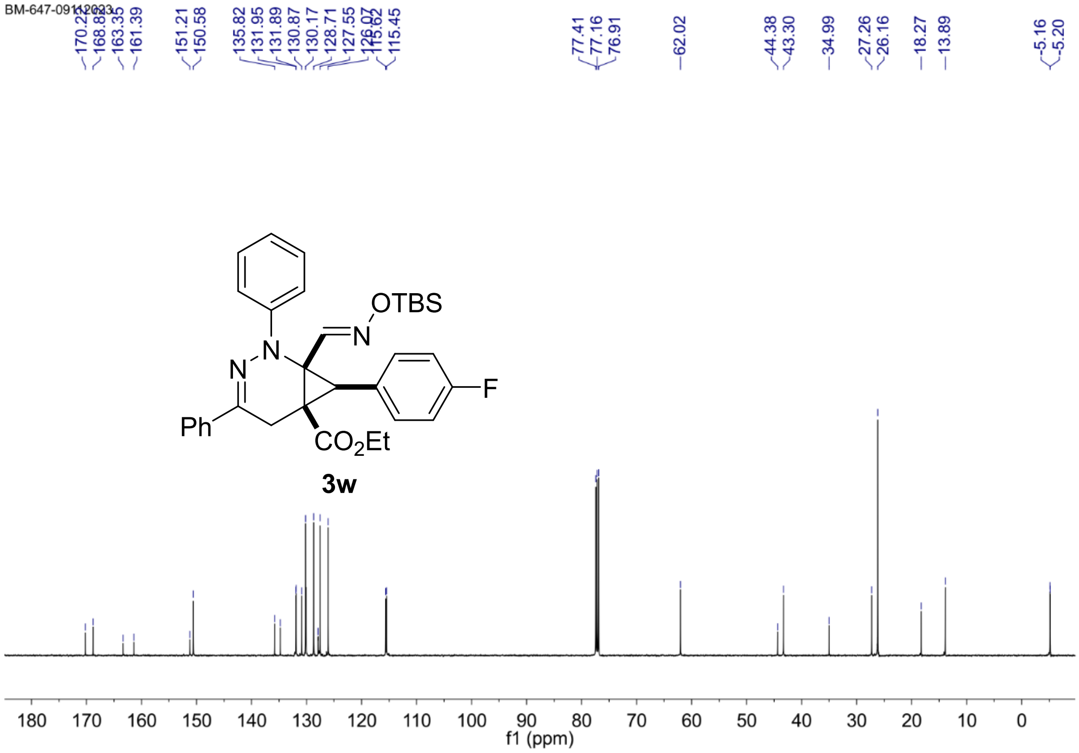
OTBS
N
N
N
F
Ph
CO2Et
3w
f1 (ppm)

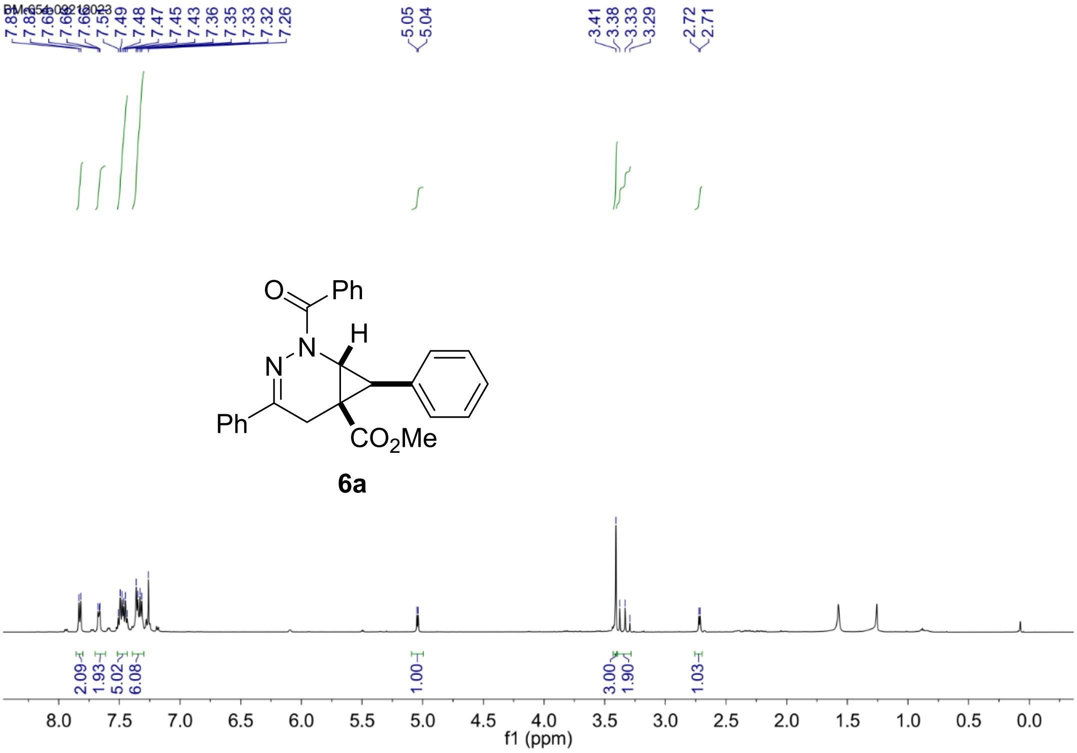
BM-654-09212023
6a
f1 (ppm)

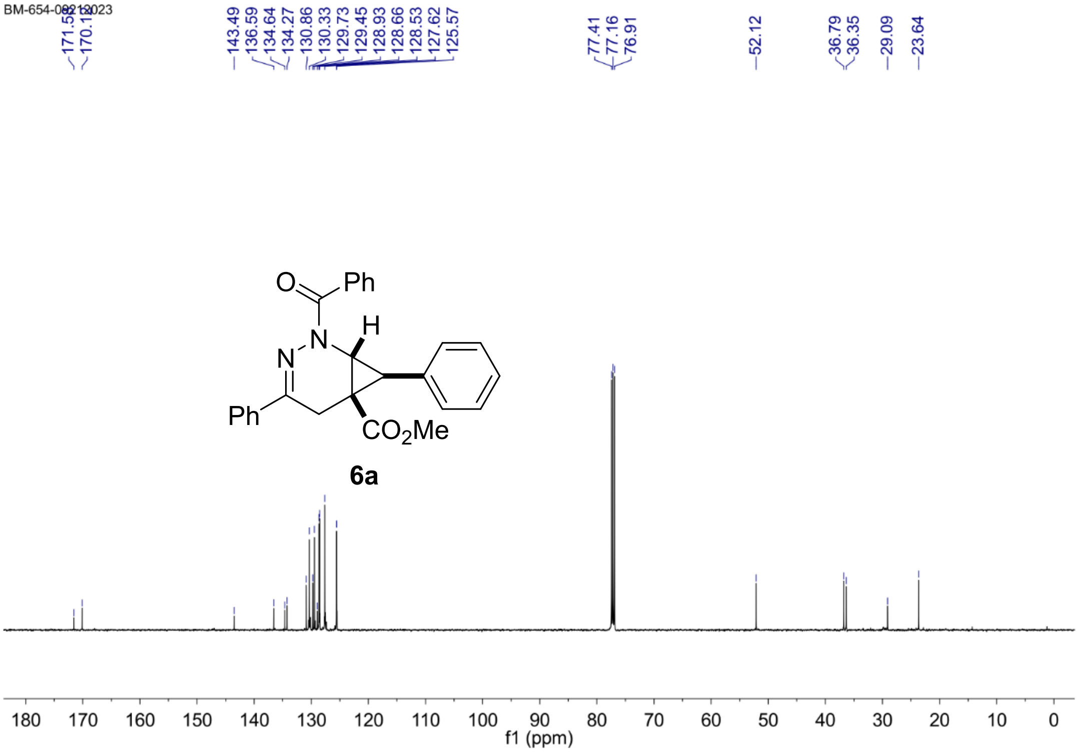
BM-654-09212023
6a
f1 (ppm)

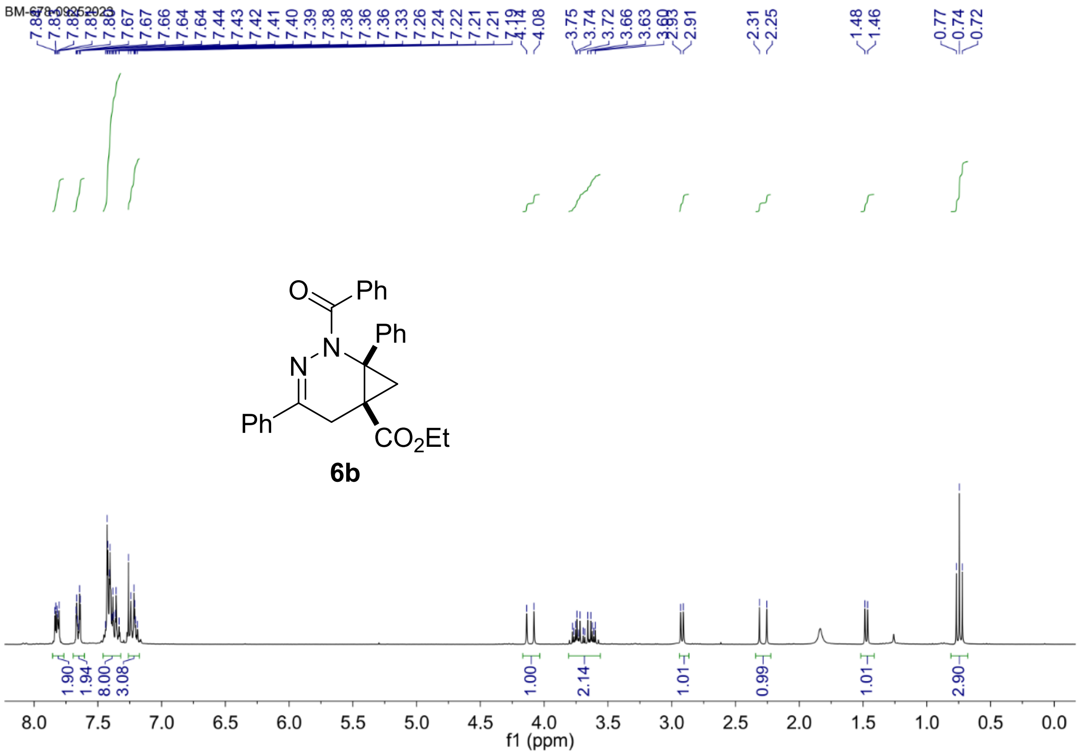
O
Ph
N
N
Ph
Ph
CO2Et
6b
f1 (ppm)

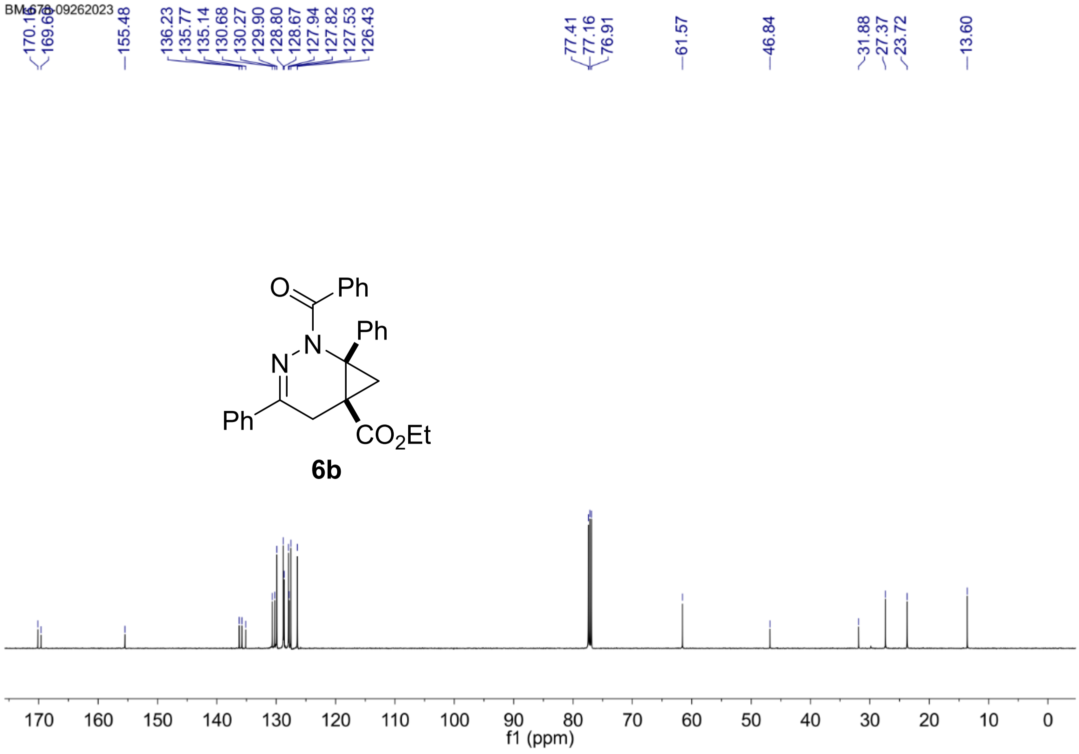
O
Ph
N
N
Ph
Ph
CO2Et
6b
f1 (ppm)

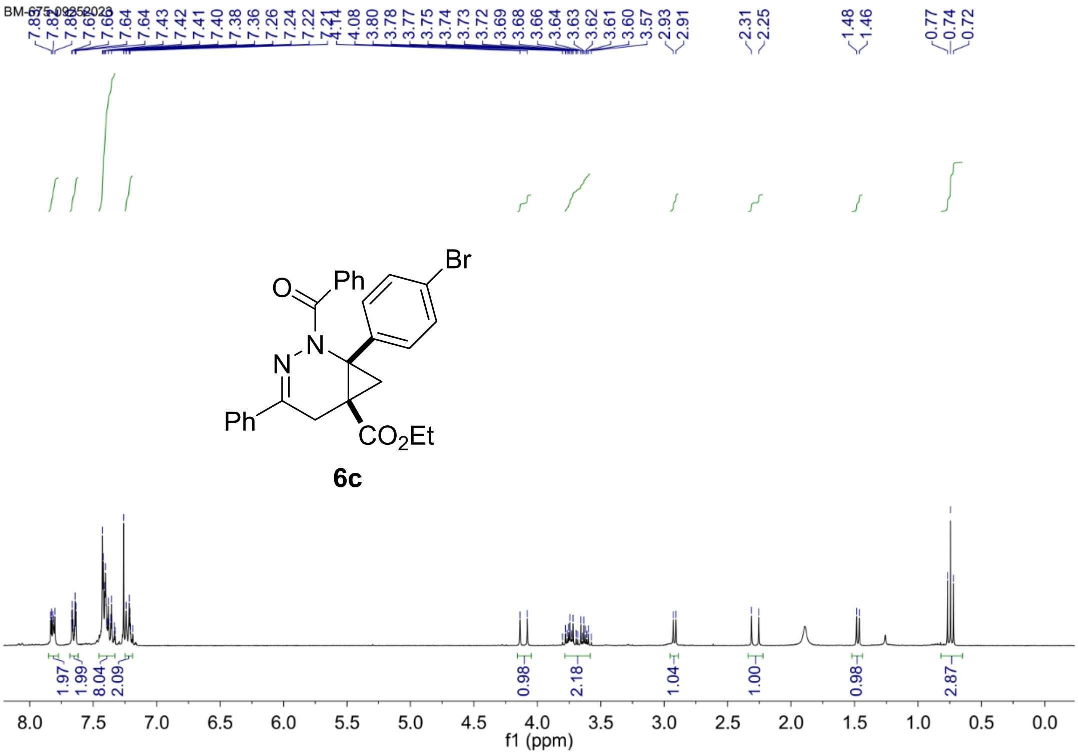
Br
O
Ph
N
N
Ph
CO2Et
6c
f1 (ppm)

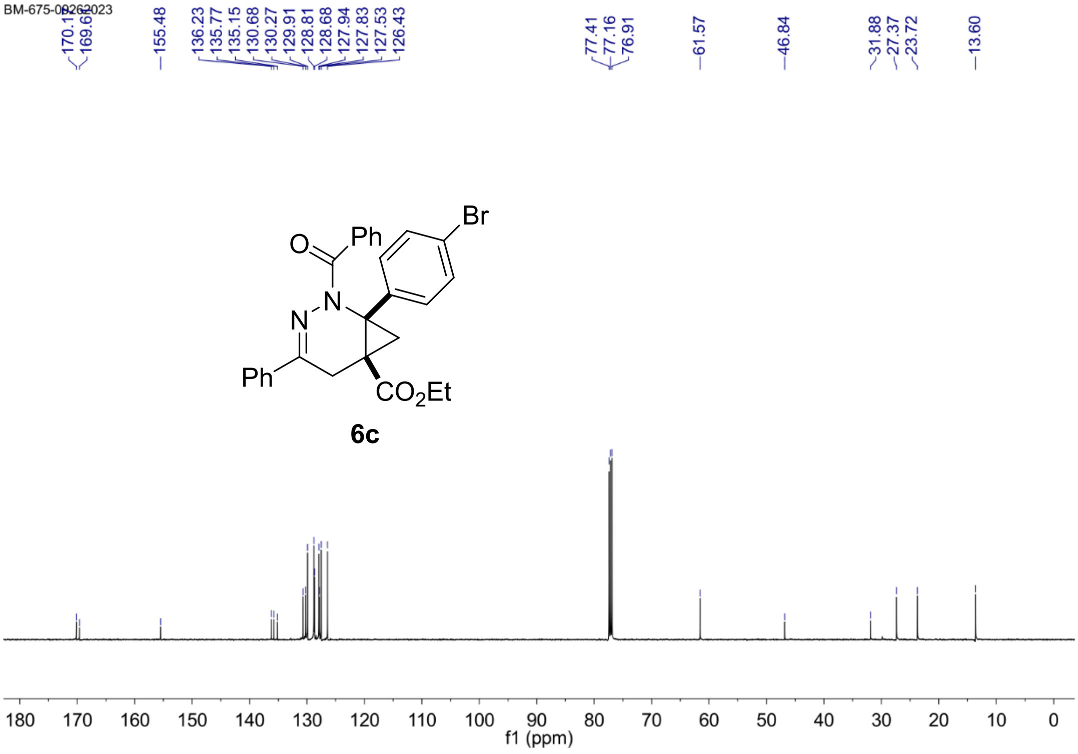
BM-675-09262023
Br
O
Ph
N
N
Ph
CO2Et
6c
f1 (ppm)

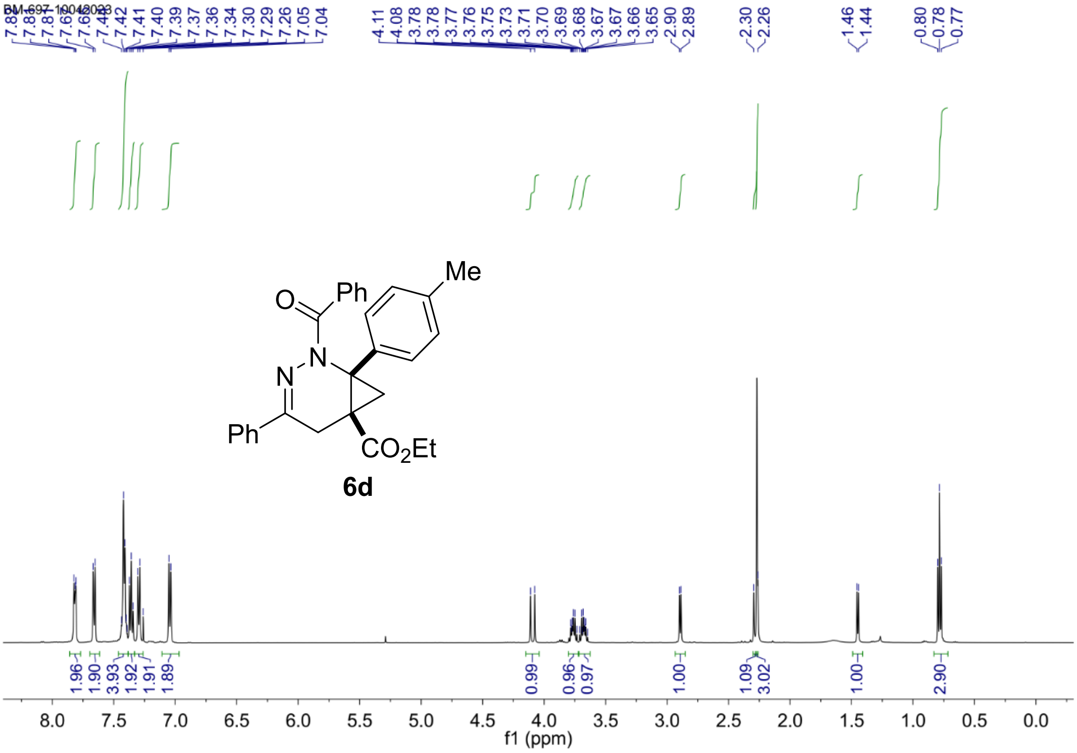
Me
O
Ph
N
N
Ph
CO2Et
6d
f1 (ppm)

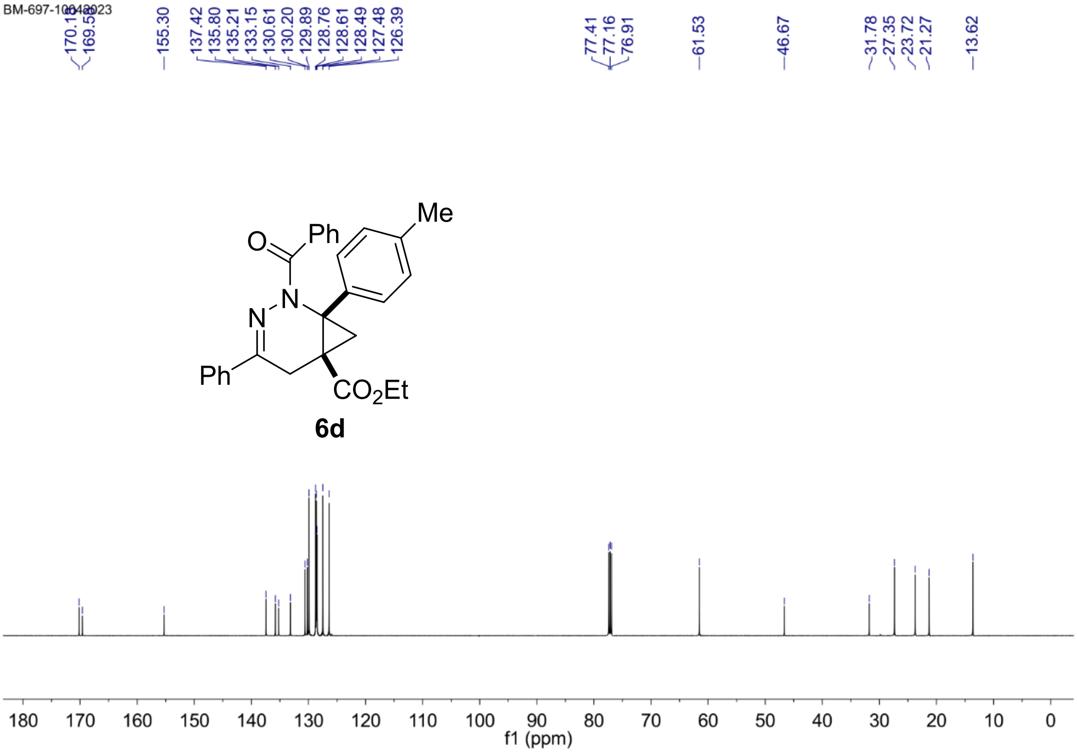
Me
O
Ph
N
N
Ph
CO2Et
6d
f1 (ppm)

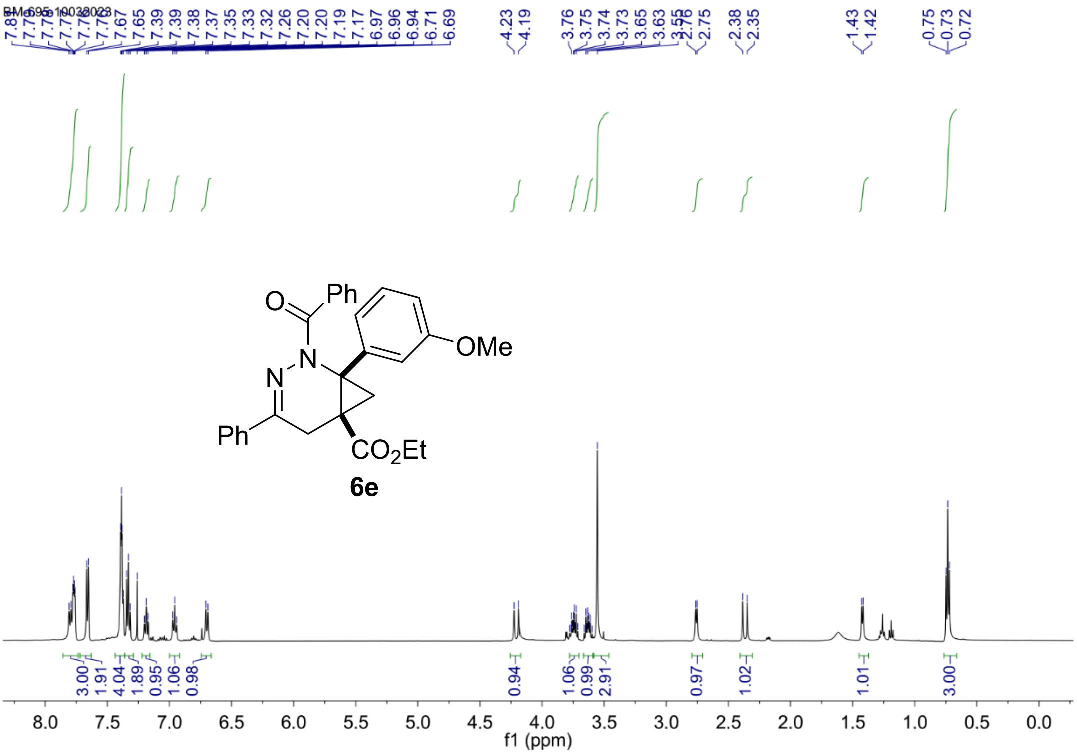
6e
f1 (ppm)

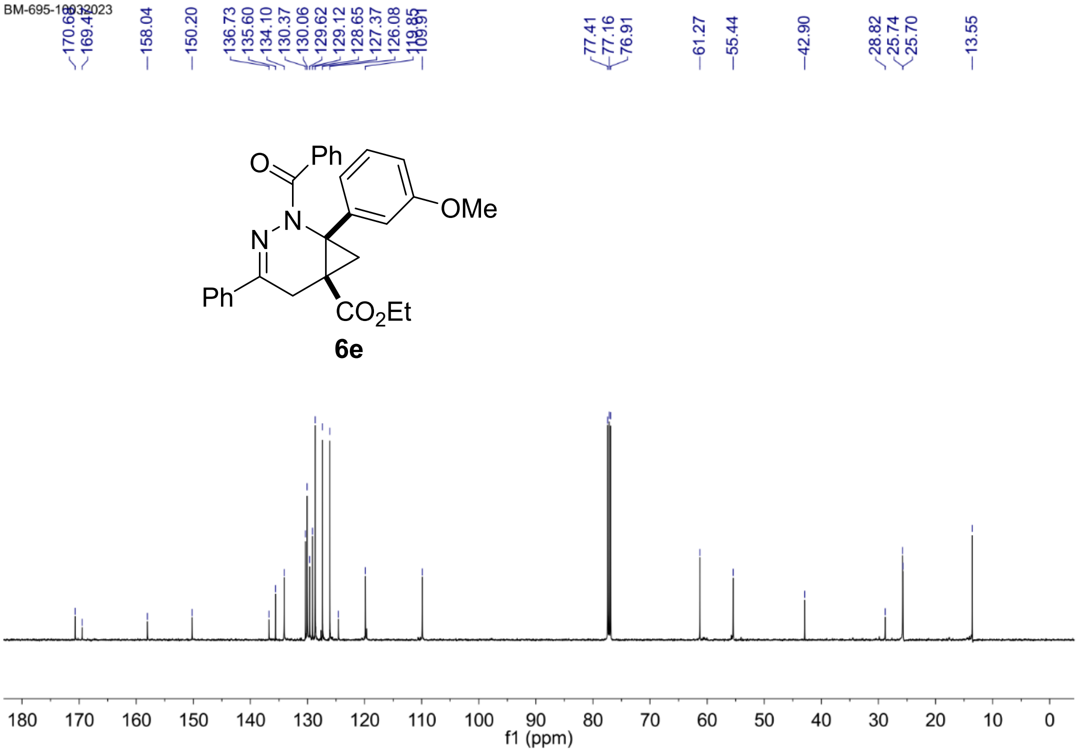
6e
f1 (ppm)

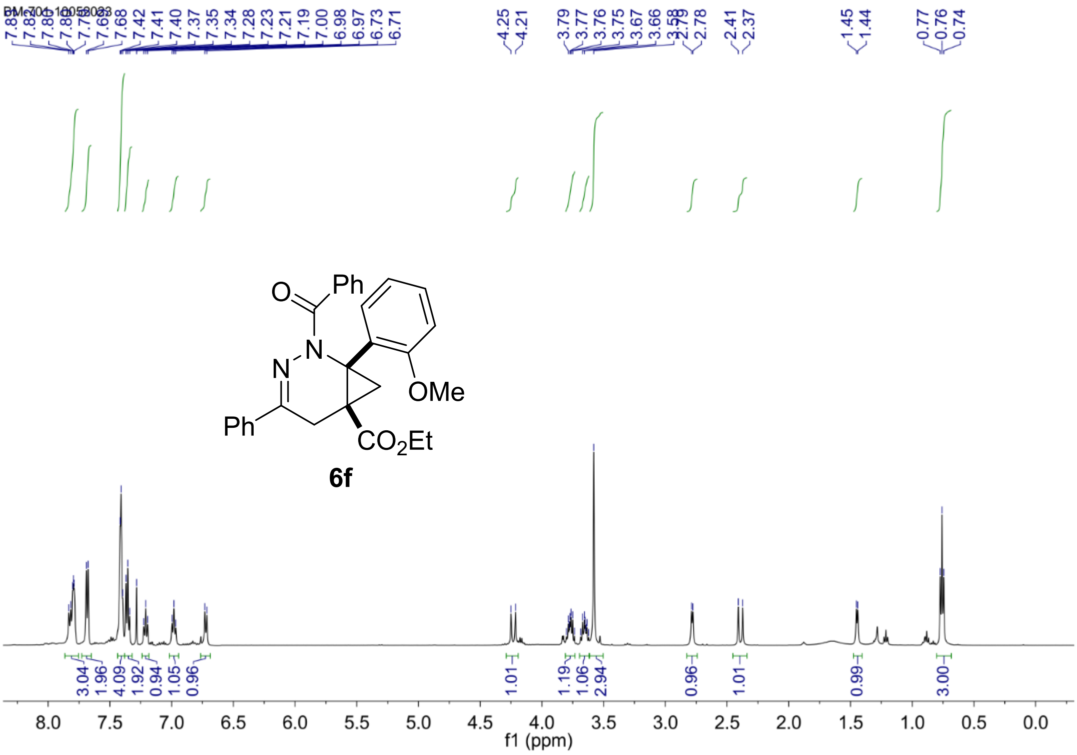
BM-701-10052023
6f
OMe
CO2Et
Ph
f1 (ppm)

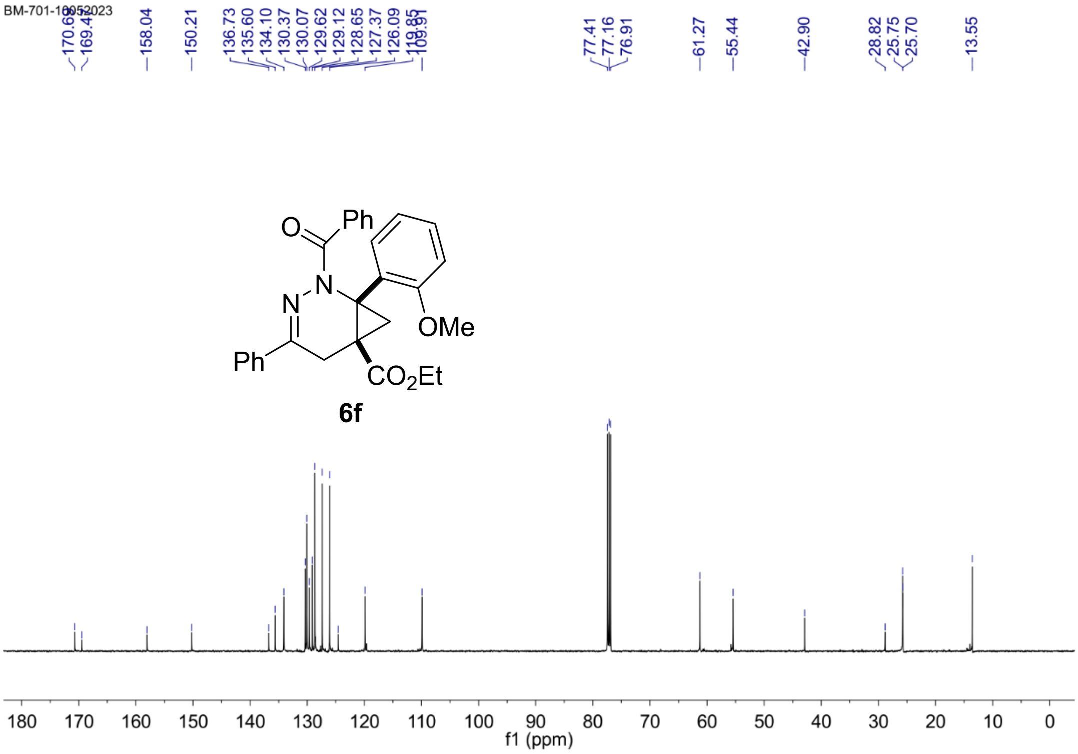
BM-701-10052023
6f
OMe
CO2Et
Ph
f1 (ppm)

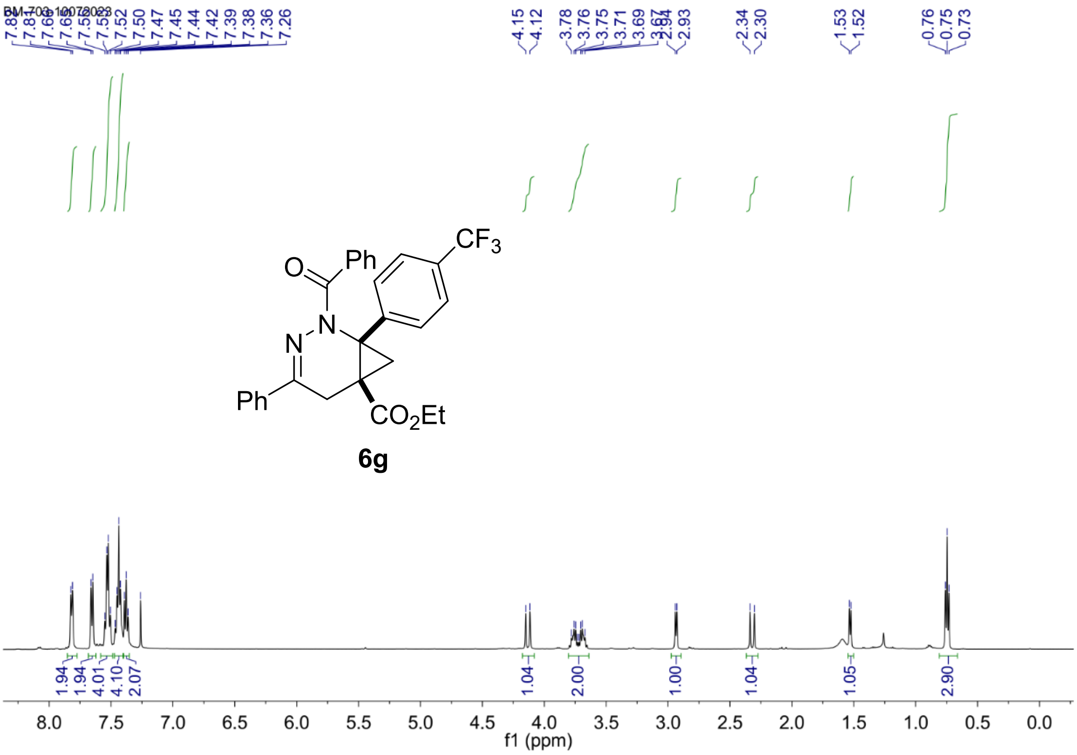

4.15
4.12
3.78
3.76
3.75
3.71
3.69
2.93
2.34
2.30
1.53
1.52
0.76
0.75
0.73
7.52
7.50
7.47
7.45
7.44
7.42
7.39
7.38
7.36
7.26
O
Ph
CF3
N
N
Ph
CO2Et
6g
1.94
1.94
4.01
4.10
2.07
1.04
2.00
1.00
1.04
1.05
2.90
f1 (ppm)

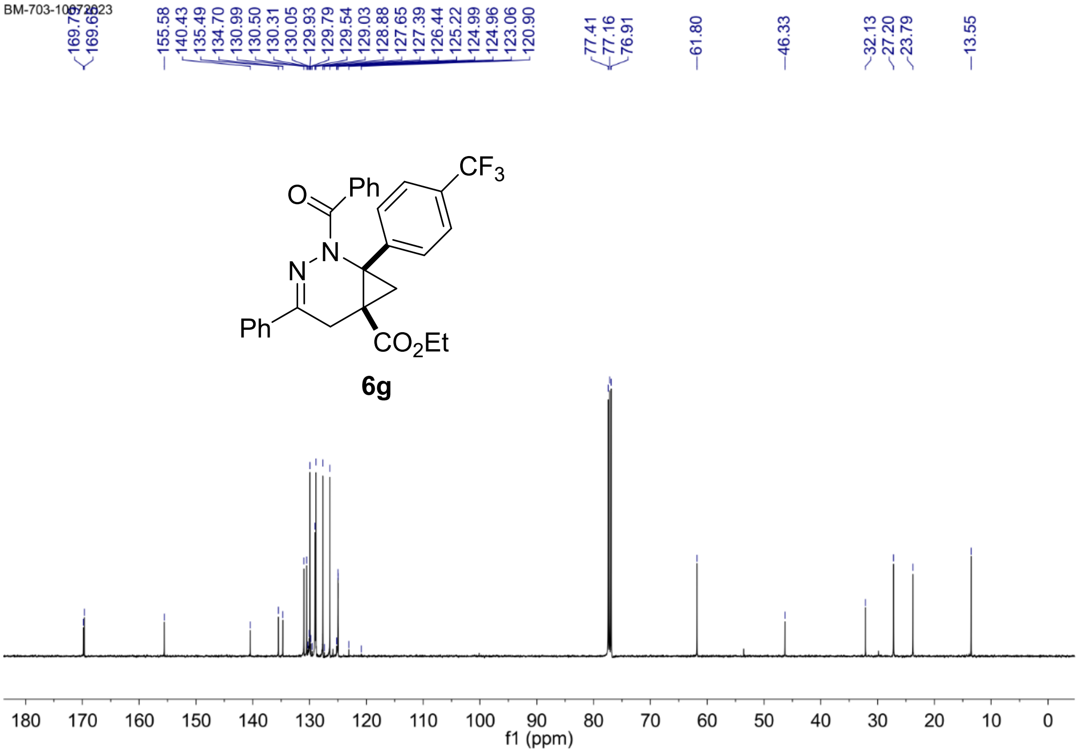

155.58
140.43
135.49
134.70
130.99
130.50
130.31
130.05
129.93
129.79
129.54
129.03
128.88
127.65
127.39
126.44
125.22
124.99
124.96
123.06
120.90
77.41
77.16
76.91
61.80
46.33
32.13
27.20
23.79
13.55
O
Ph
CF3
N
N
Ph
CO2Et
6g
f1 (ppm)

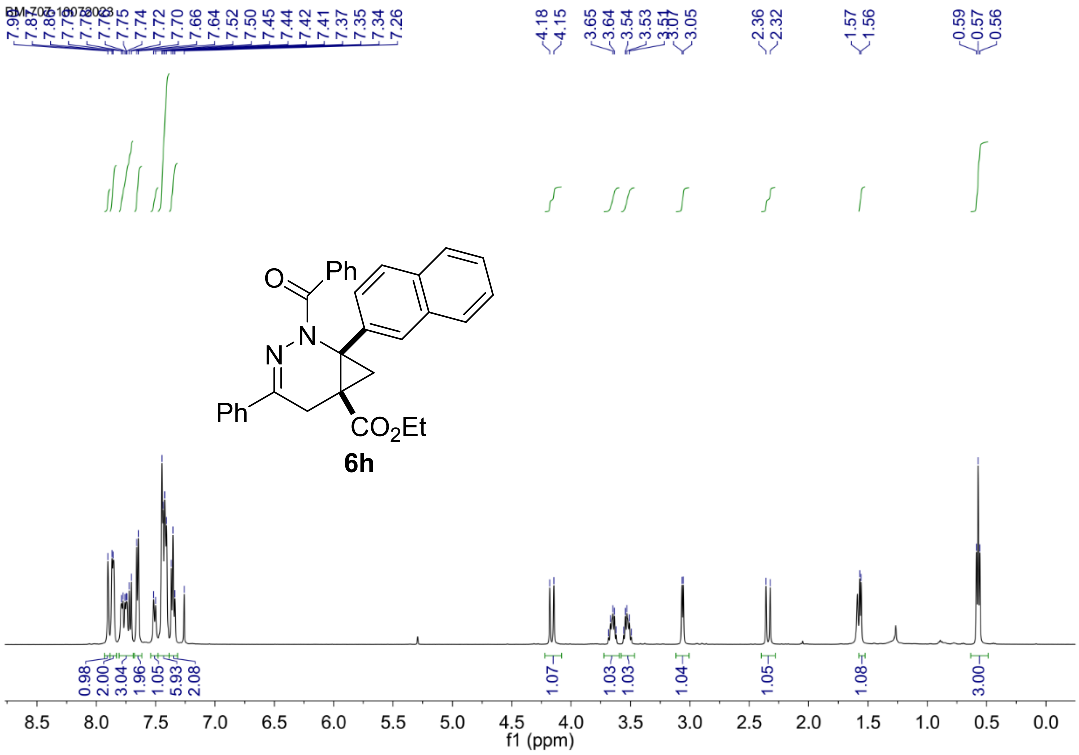

6h

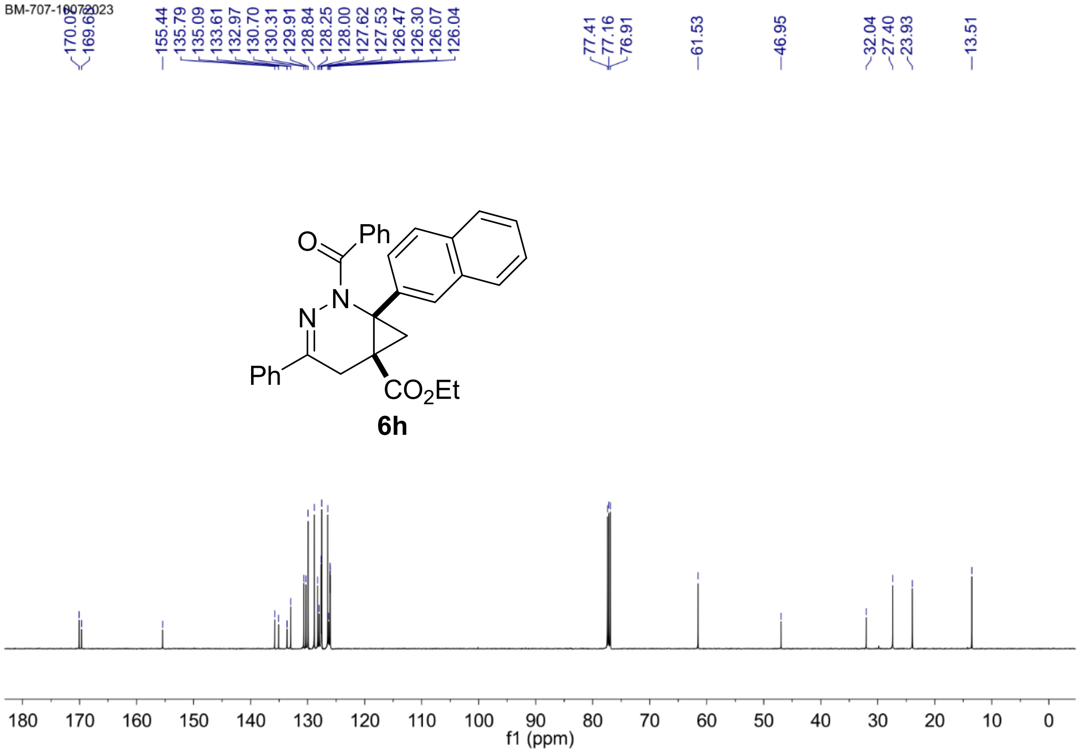

6h

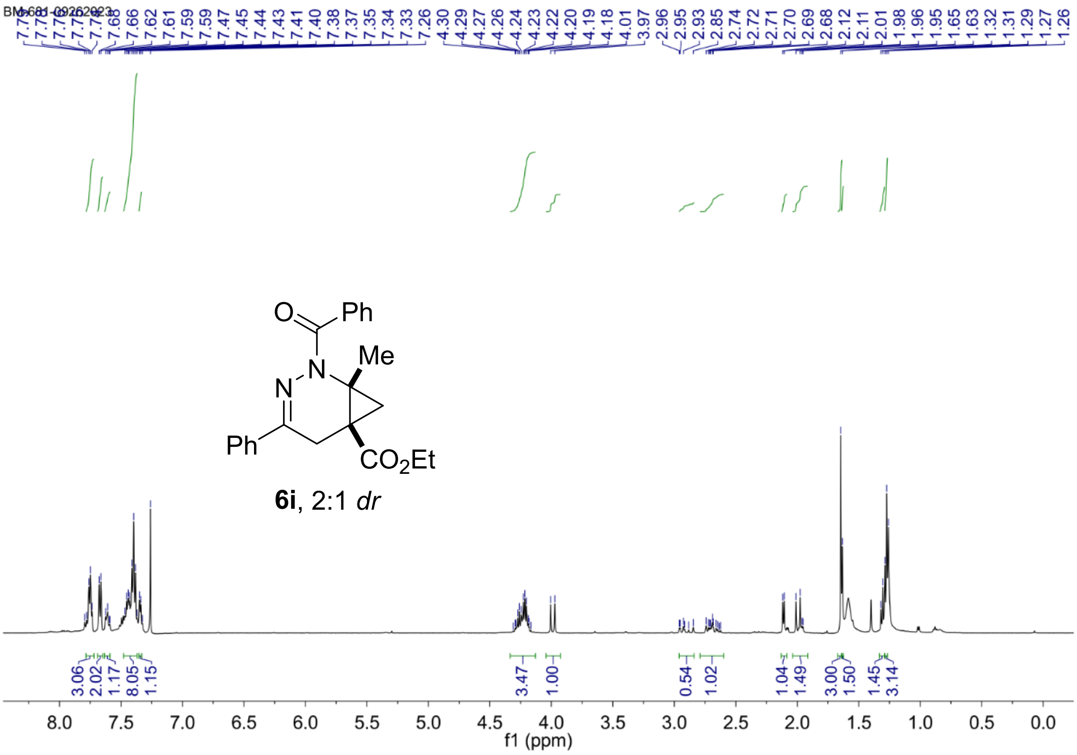
BM-681-09262023
7.68 7.66 7.62 7.61 7.59 7.59 7.47 7.45 7.44 7.43 7.41 7.40 7.38 7.37 7.35 7.34 7.33 7.26 4.30 4.29 4.27 4.26 4.24 4.23 4.22 4.20 4.19 4.18 4.01 3.97 2.96 2.95 2.93 2.85 2.74 2.72 2.71 2.70 2.69 2.68 2.12 2.11 2.01 1.98 1.96 1.95 1.65 1.63 1.32 1.31 1.29 1.27 1.26
O
Ph
N
Me
N
Ph
CO2Et
6i, 2:1 dr
3.06 2.02 1.17 8.05 1.15 3.47 1.00 0.54 1.02 1.04 1.49 3.00 1.50 1.45 3.14
8.0 7.5 7.0 6.5 6.0 5.5 5.0 4.5 4.0 3.5 3.0 2.5 2.0 1.5 1.0 0.5 0.0
f1 (ppm)

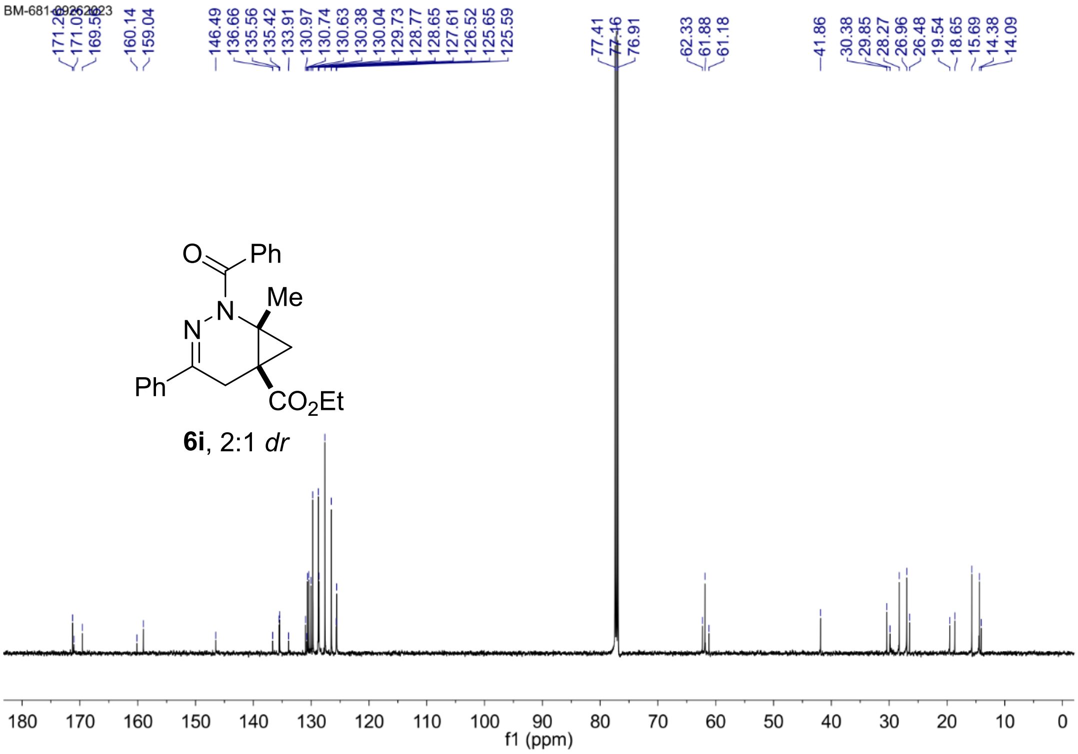
BM-681-09262023
171.20 171.03 169.58 160.14 159.04 146.49 136.66 135.56 135.42 133.91 130.97 130.74 130.63 130.38 130.04 129.73 128.77 128.65 127.61 126.52 125.65 125.59 77.41 77.16 76.91 62.33 61.88 61.18 41.86 30.38 29.85 28.27 26.96 26.48 19.54 18.65 15.69 14.38 14.09
O
Ph
N
Me
N
Ph
CO2Et
6i, 2:1 dr
180 170 160 150 140 130 120 110 100 90 80 70 60 50 40 30 20 10 0
f1 (ppm)

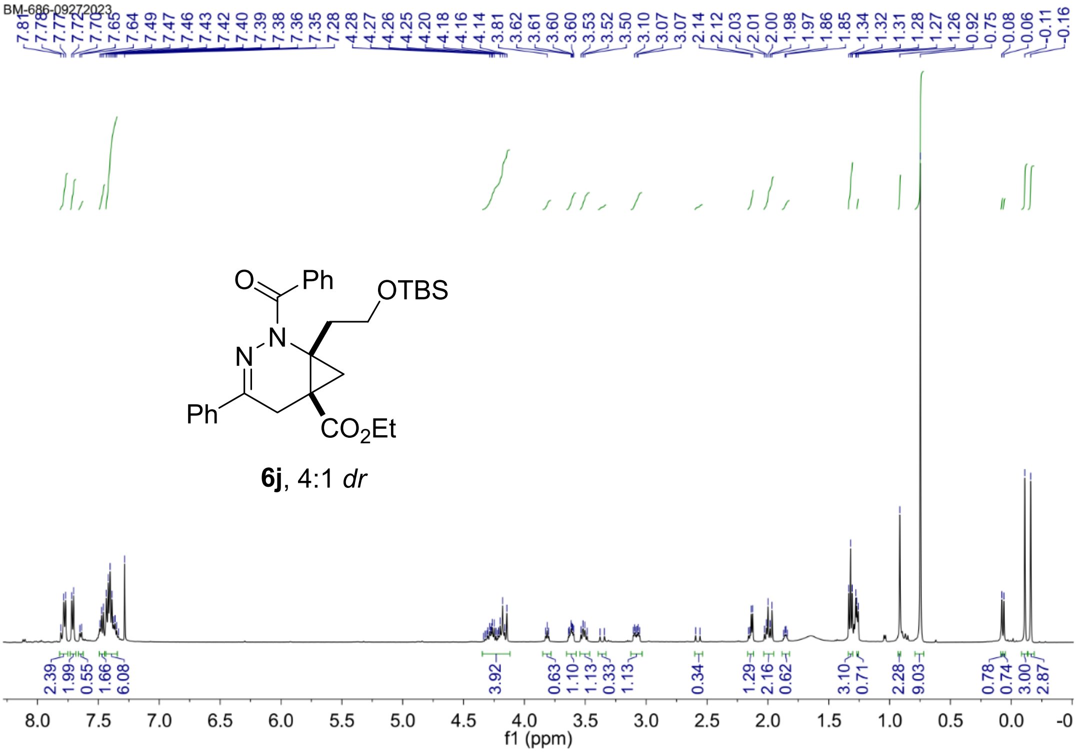
BM-686-09272023
7.81 7.78 7.77 7.72 7.70 7.65 7.64 7.49 7.47 7.46 7.43 7.42 7.40 7.39 7.38 7.36 7.35 7.28 4.28 4.27 4.26 4.25 4.20 4.18 4.16 4.14 3.81 3.62 3.61 3.60 3.60 3.53 3.52 3.50 3.10 3.07 3.07 2.14 2.12 2.03 2.01 2.00 1.98 1.97 1.86 1.85 1.34 1.32 1.31 1.28 1.27 1.26 0.92 0.75 0.08 0.06 -0.11 -0.16
O
Ph
OTBS
N
N
Ph
CO2Et
6j, 4:1 dr
2.39 1.99 0.55 1.66 6.08 3.92 0.63 1.10 1.13 0.33 1.13 0.34 1.29 2.16 0.62 3.10 0.71 2.28 9.03 0.78 0.74 3.00 2.87
f1 (ppm)

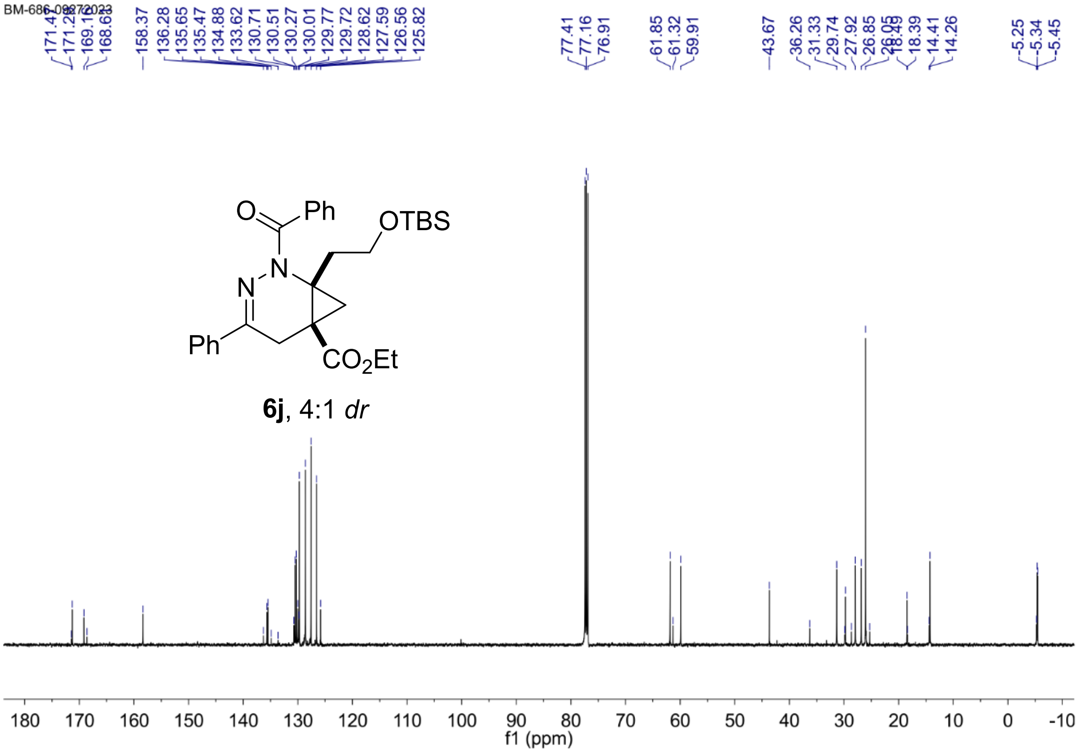
BM-686-09272023
171.41 171.22 169.16 168.63 158.37 136.28 135.65 135.47 134.88 133.62 130.71 130.51 130.27 130.01 129.77 129.72 128.62 127.59 126.56 125.82 77.41 77.16 76.91 61.85 61.32 59.91 43.67 36.26 31.33 29.74 27.92 26.85 26.05 18.49 18.39 14.41 14.26 -5.25 -5.34 -5.45
O
Ph
OTBS
N
N
Ph
CO2Et
6j, 4:1 dr
f1 (ppm)

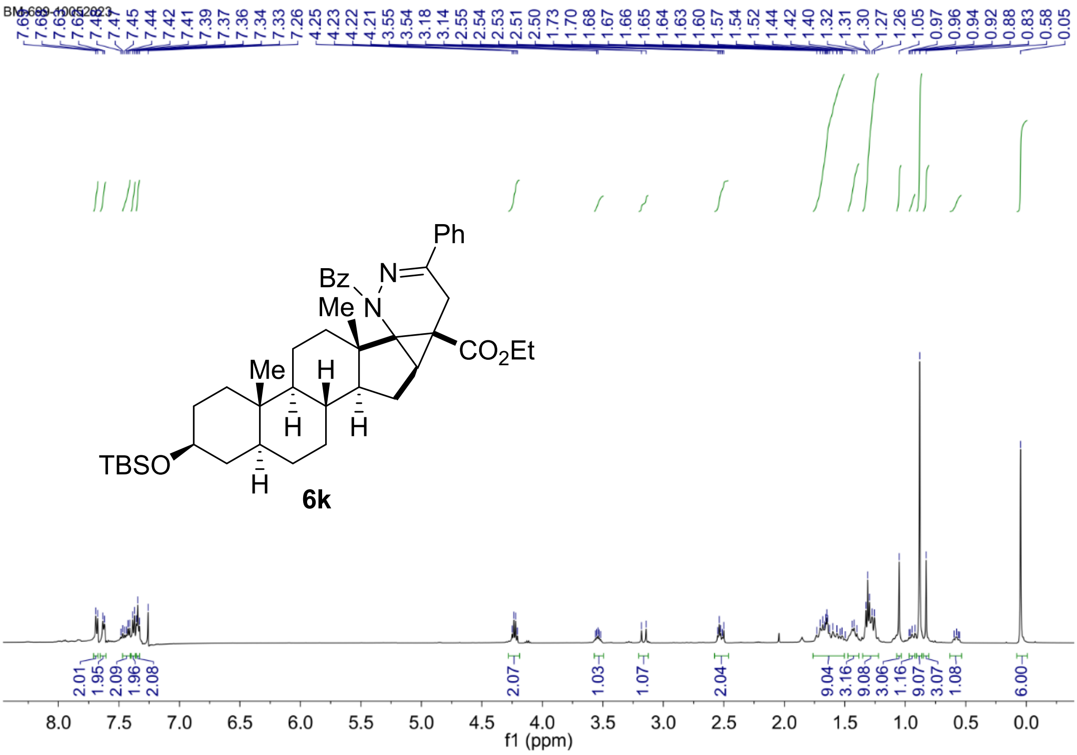
BM-699-10052023
7.69 7.68 7.63 7.62 7.48 7.47 7.45 7.44 7.42 7.41 7.39 7.37 7.36 7.34 7.33 7.26 4.25 4.23 4.22 4.21 3.55 3.54 3.18 3.14 2.55 2.54 2.53 2.51 2.50 1.73 1.70 1.68 1.67 1.66 1.65 1.64 1.63 1.60 1.57 1.54 1.52 1.44 1.42 1.40 1.32 1.31 1.30 1.27 1.26 1.05 0.97 0.96 0.94 0.92 0.88 0.83 0.58 0.05
Ph
Bz
N
Me
N
CO2Et
Me
H
H
H
TBSO
H
6k
2.01 1.95 2.09 1.96 2.08 2.07 1.03 1.07 2.04 9.04 3.16 9.08 3.06 1.16 9.07 3.07 1.08 6.00
8.0 7.5 7.0 6.5 6.0 5.5 5.0 4.5 4.0 3.5 3.0 2.5 2.0 1.5 1.0 0.5 0.0
f1 (ppm)

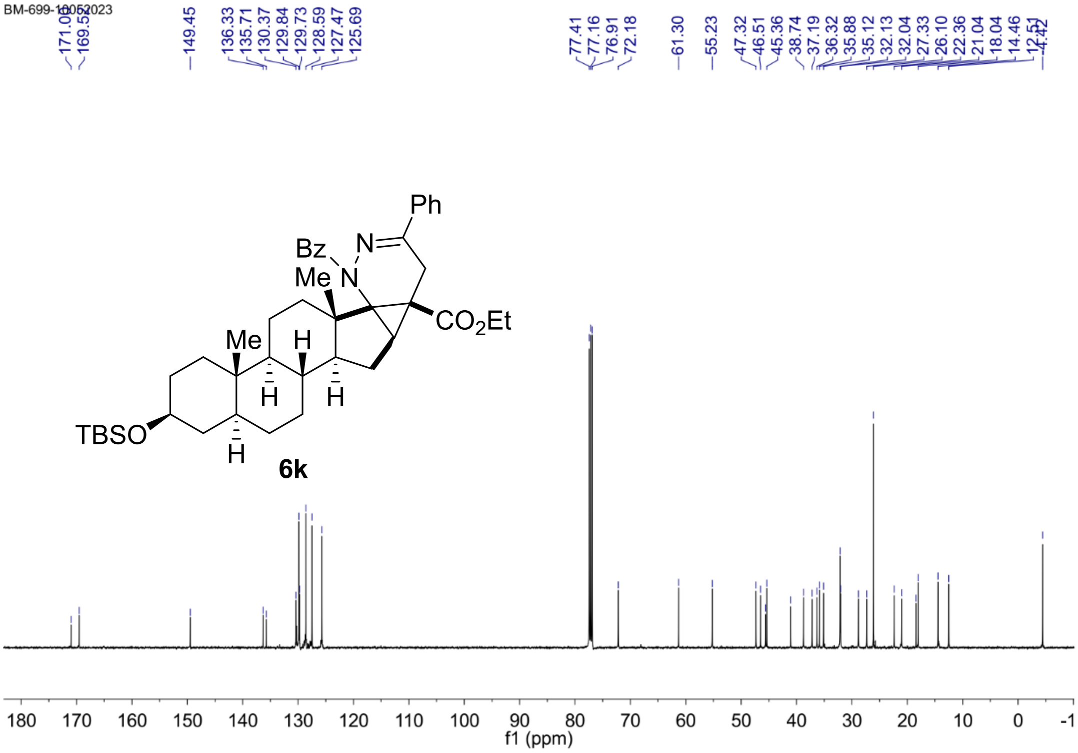
BM-699-10052023
171.00 169.52 149.45 136.33 135.71 130.37 129.84 129.73 128.59 127.47 125.69 77.41 77.16 76.91 72.18 61.30 55.23 47.32 46.51 45.36 38.74 37.19 36.32 35.88 35.12 32.13 32.04 27.33 26.10 22.36 21.04 18.04 14.46 12.51 -4.42
Ph
Bz
N
Me
N
CO2Et
Me
H
H
H
TBSO
H
6k
180 170 160 150 140 130 120 110 100 90 80 70 60 50 40 30 20 10 0 -1
f1 (ppm)

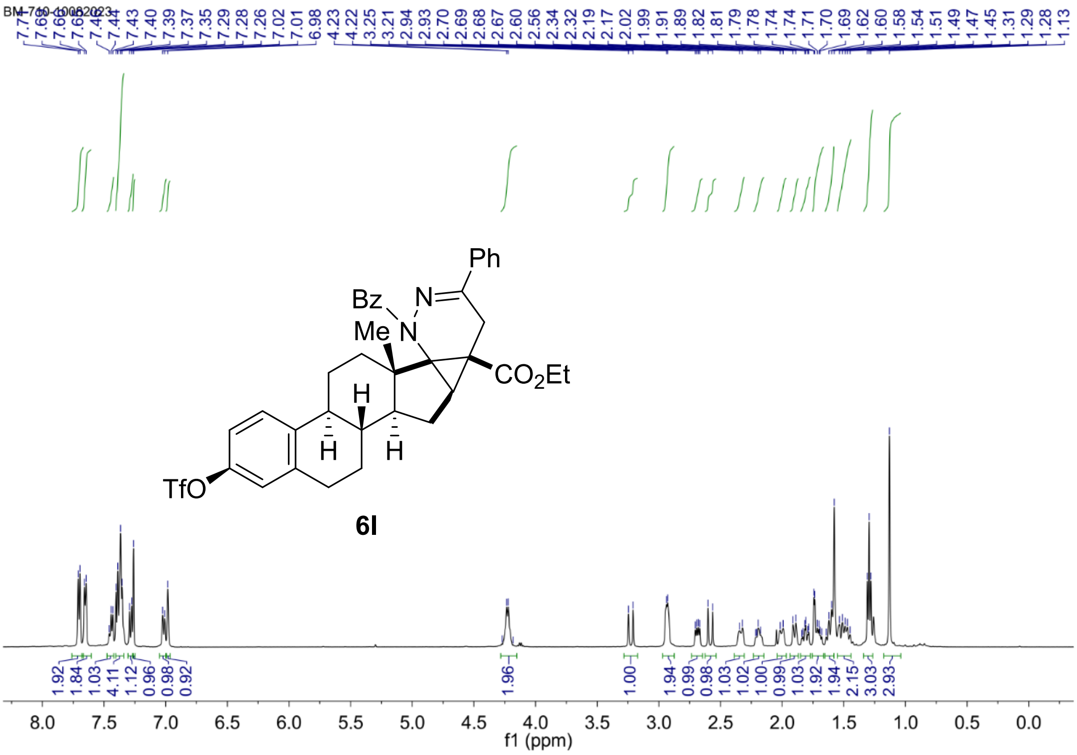

Ph
Bz
N
Me
N
CO2Et
H
H
H
TfO
6l
f1 (ppm)

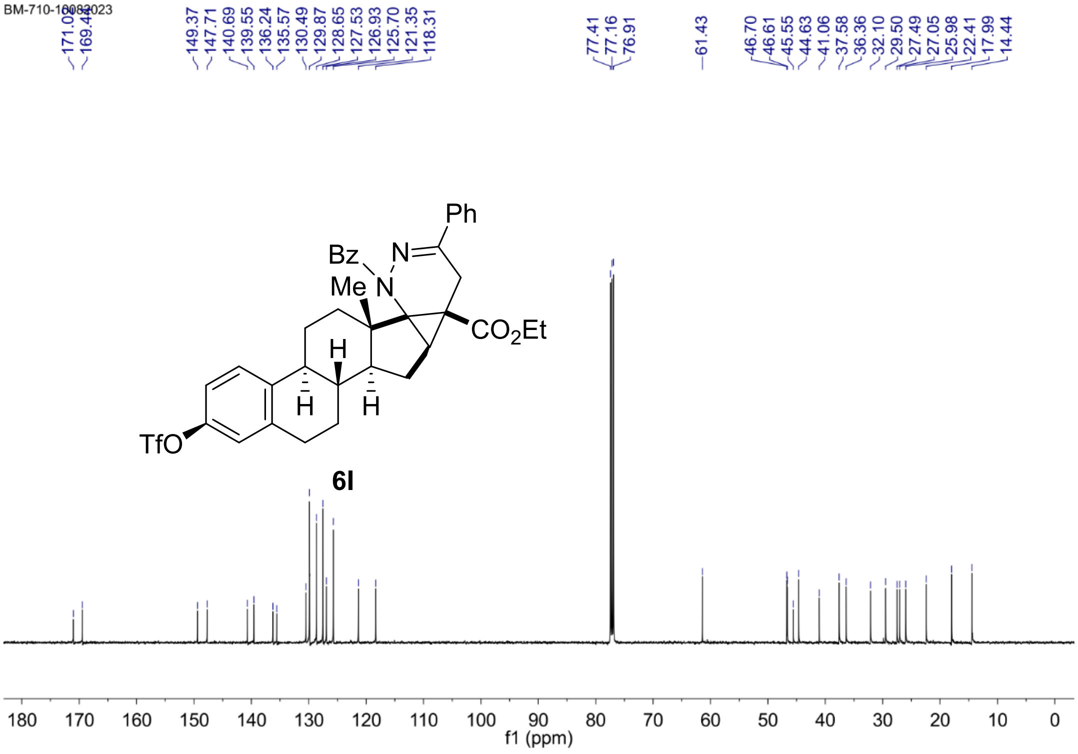

BM-710-10082023
Ph
Bz
N
Me
N
CO2Et
H
H
H
TfO
6l
f1 (ppm)

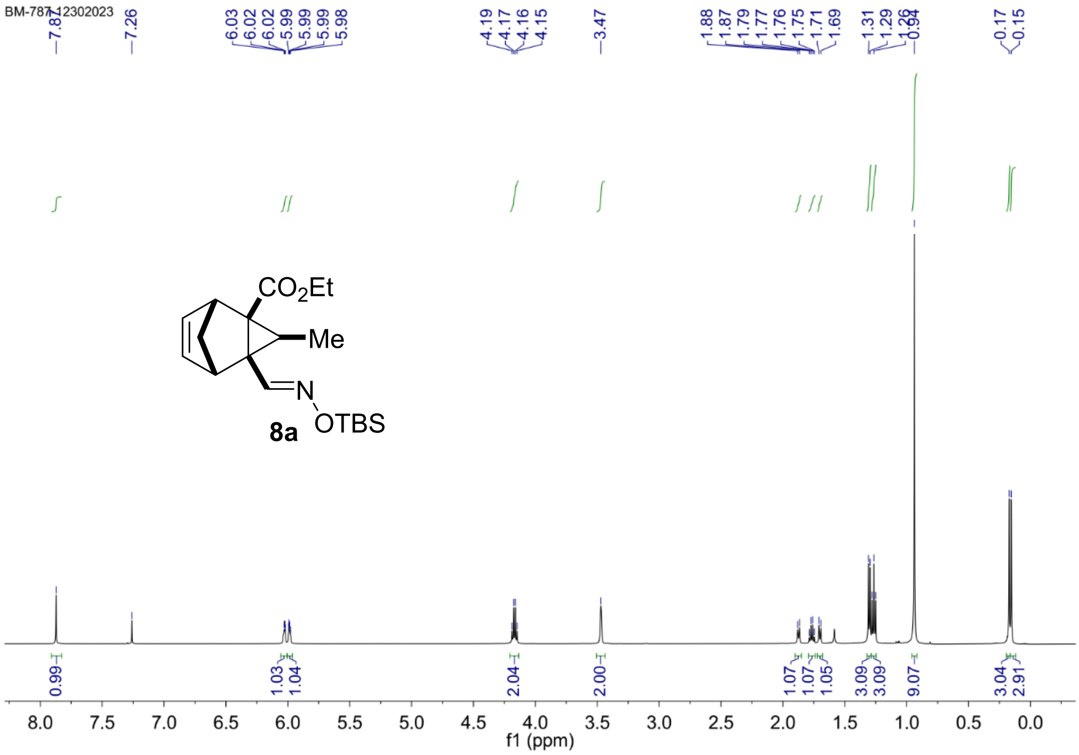
BM-787-12302023
7.87
7.26
6.03
6.02
6.02
5.99
5.99
5.99
5.98
4.19
4.17
4.16
4.15
3.47
1.88
1.87
1.79
1.77
1.76
1.75
1.71
1.69
1.31
1.29
1.26
0.94
0.17
0.15
CO2Et
Me
N
OTBS
8a
0.99
1.03
1.04
2.04
2.00
1.07
1.07
1.05
3.09
3.09
9.07
3.04
2.91
f1 (ppm)

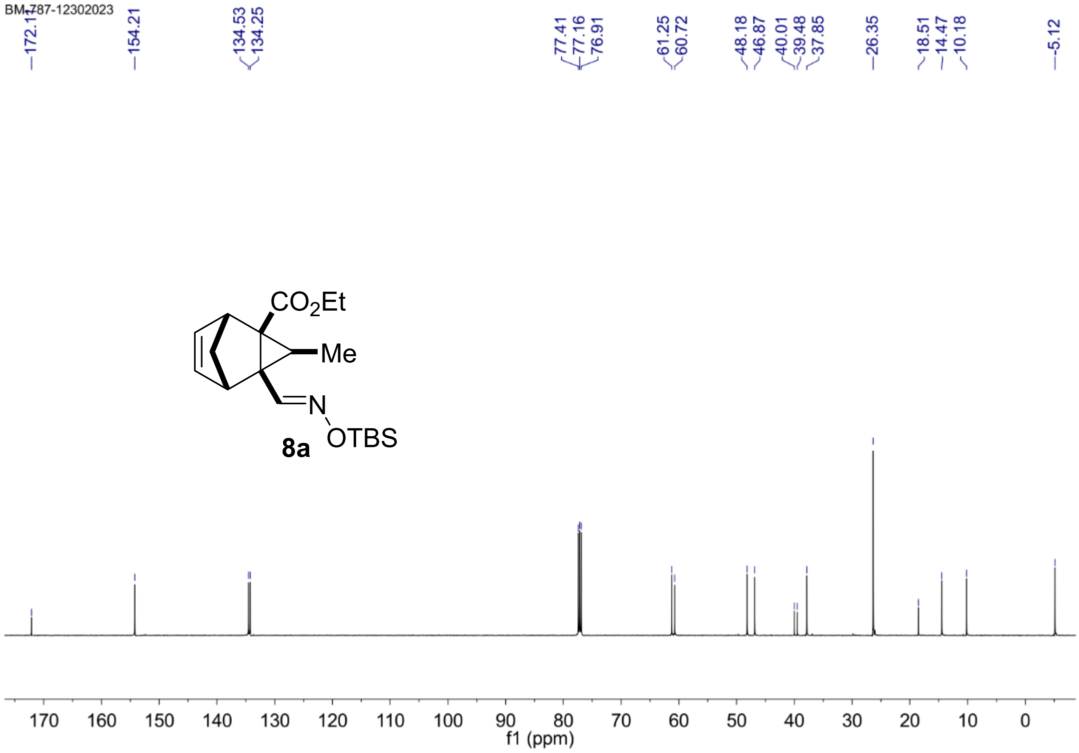
BM-787-12302023
172.11
154.21
134.53
134.25
77.41
77.16
76.91
61.25
60.72
48.18
46.87
40.01
39.48
37.85
26.35
18.51
14.47
10.18
-5.12
CO2Et
Me
N
OTBS
8a
f1 (ppm)

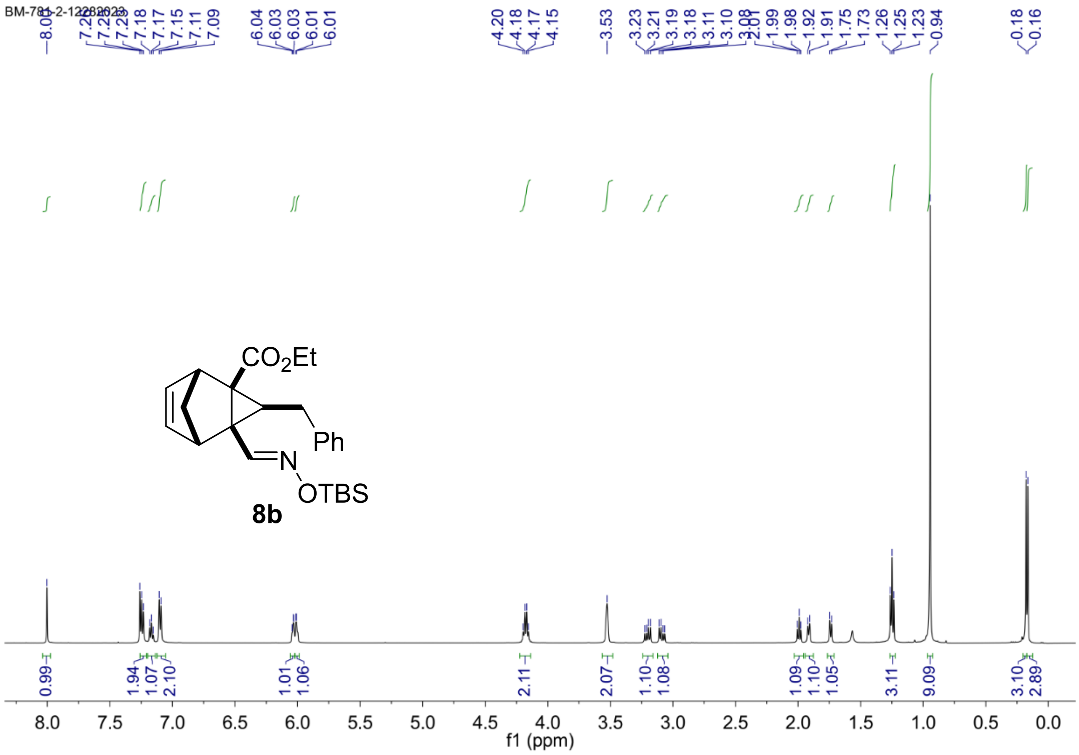

BM-781-2-12282023
CO2Et
Ph
N
OTBS
8b
f1 (ppm)

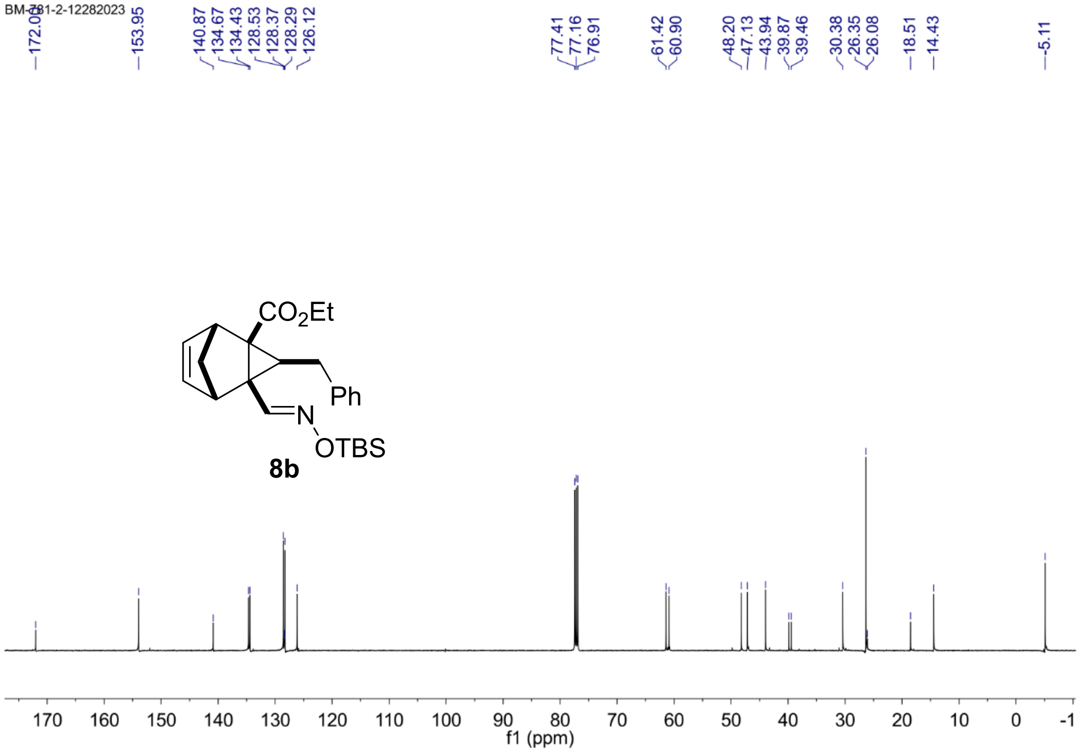

BM-781-2-12282023
172.08
153.95
140.87
134.67
134.43
128.53
128.37
128.29
126.12
77.41
77.16
76.91
61.42
60.90
48.20
47.13
43.94
39.87
39.46
30.38
26.35
26.08
18.51
14.43
-5.11
CO2Et
Ph
N
OTBS
8b
f1 (ppm)

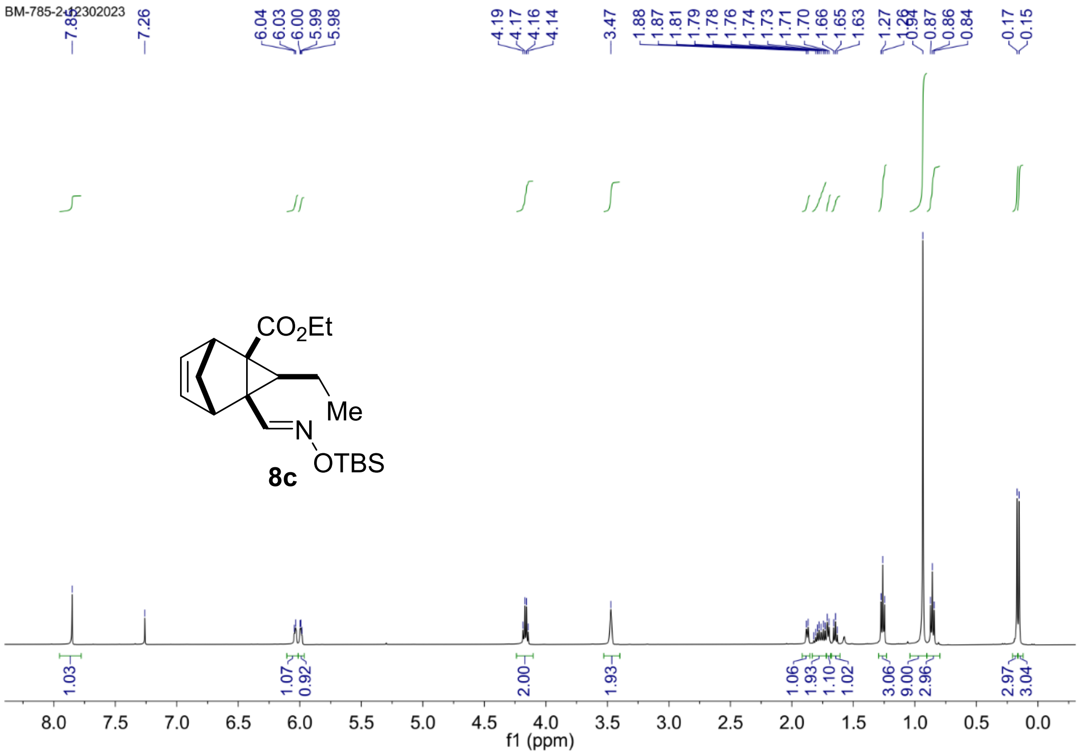
BM-785-2-12302023
CO2Et
Me
N
OTBS
8c
f1 (ppm)

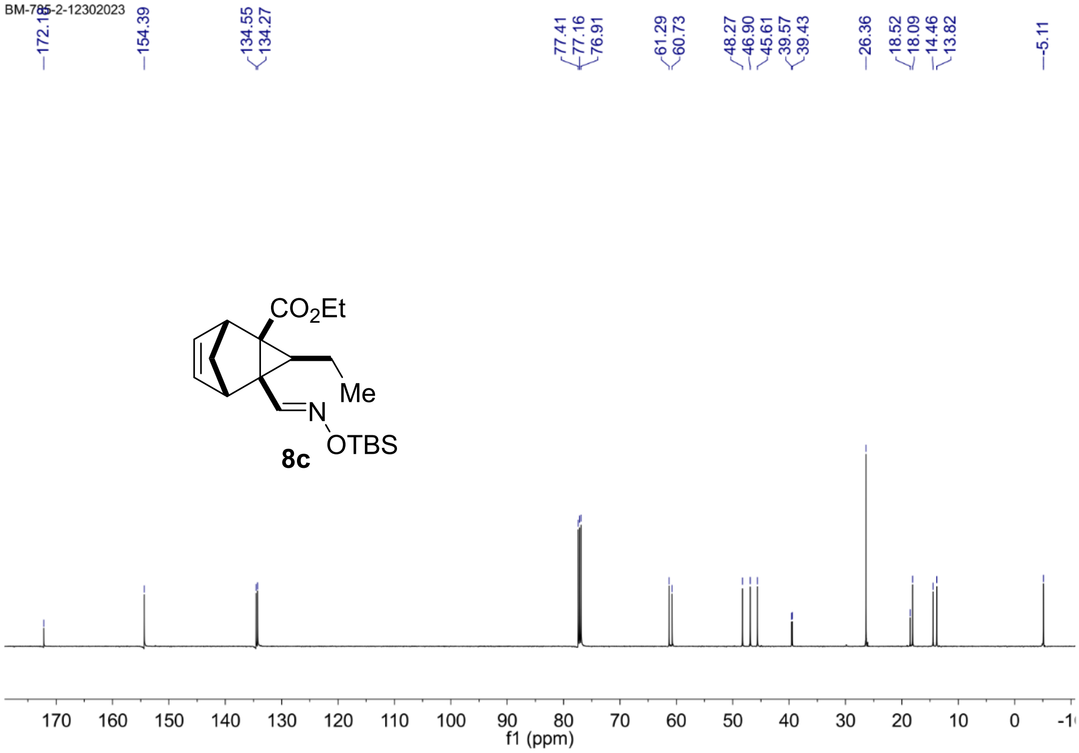
BM-785-2-12302023
CO2Et
Me
N
OTBS
8c
f1 (ppm)

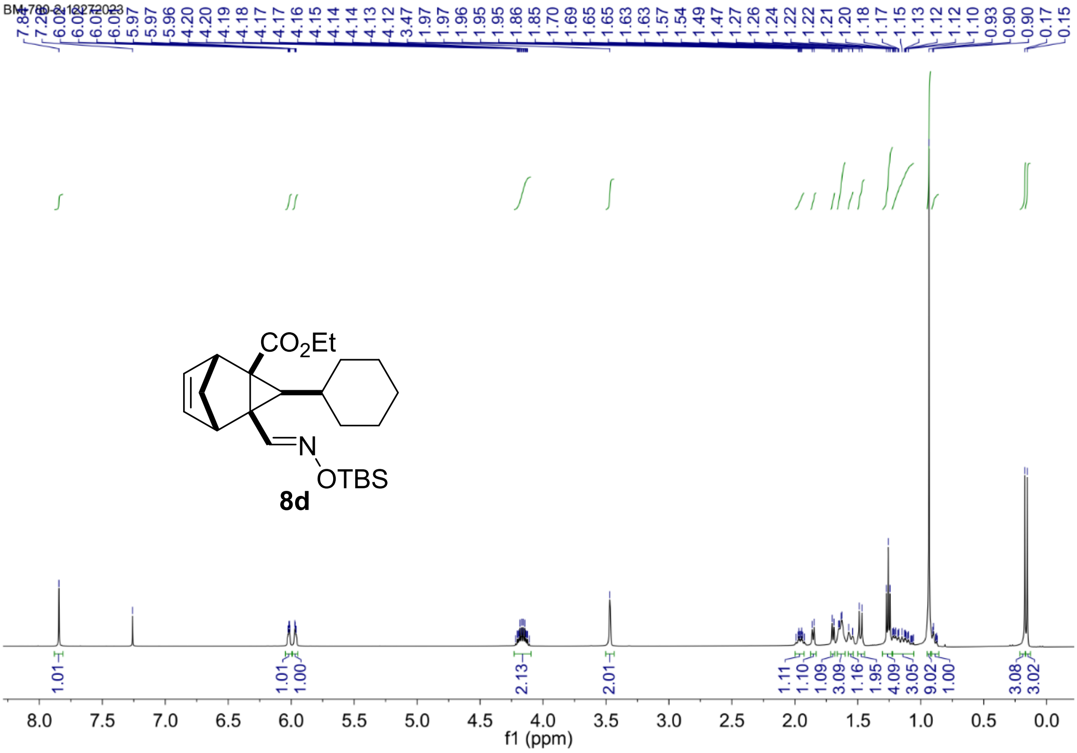
5.97
5.96
4.20
4.20
4.19
4.18
4.17
4.17
4.16
4.15
4.14
4.14
4.13
4.12
3.47
1.97
1.97
1.96
1.95
1.95
1.86
1.85
1.70
1.69
1.65
1.65
1.63
1.63
1.57
1.54
1.49
1.47
1.27
1.26
1.24
1.22
1.22
1.21
1.20
1.18
1.17
1.15
1.13
1.12
1.12
1.10
0.93
0.90
0.90
0.17
0.15
CO2Et
N
OTBS
8d
1.01
1.01
1.00
2.13
2.01
1.11
1.10
1.09
3.09
1.16
1.95
4.09
3.05
9.02
1.00
3.08
3.02
f1 (ppm)

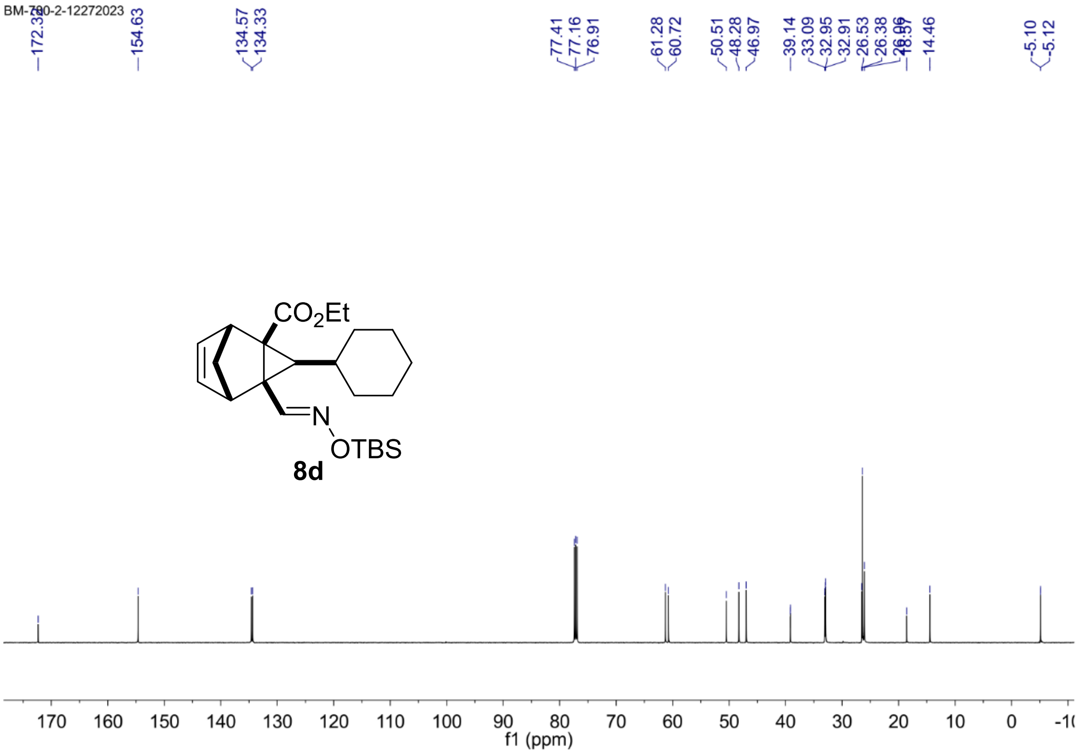
BM-7?0-2-12272023
154.63
134.57
134.33
77.41
77.16
76.91
61.28
60.72
50.51
48.28
46.97
39.14
33.09
32.95
32.91
26.53
26.38
14.46
-5.10
-5.12
CO2Et
N
OTBS
8d
f1 (ppm)

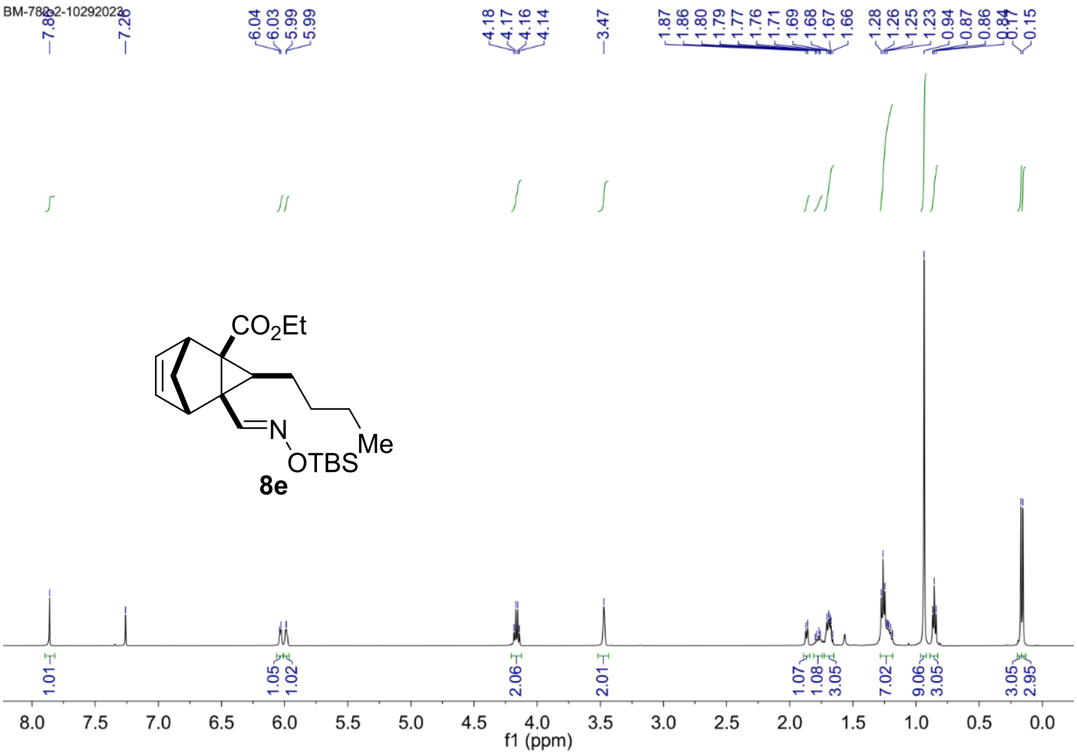
BM-782-2-10292023
CO2Et
N
OTBS
Me
8e
f1 (ppm)

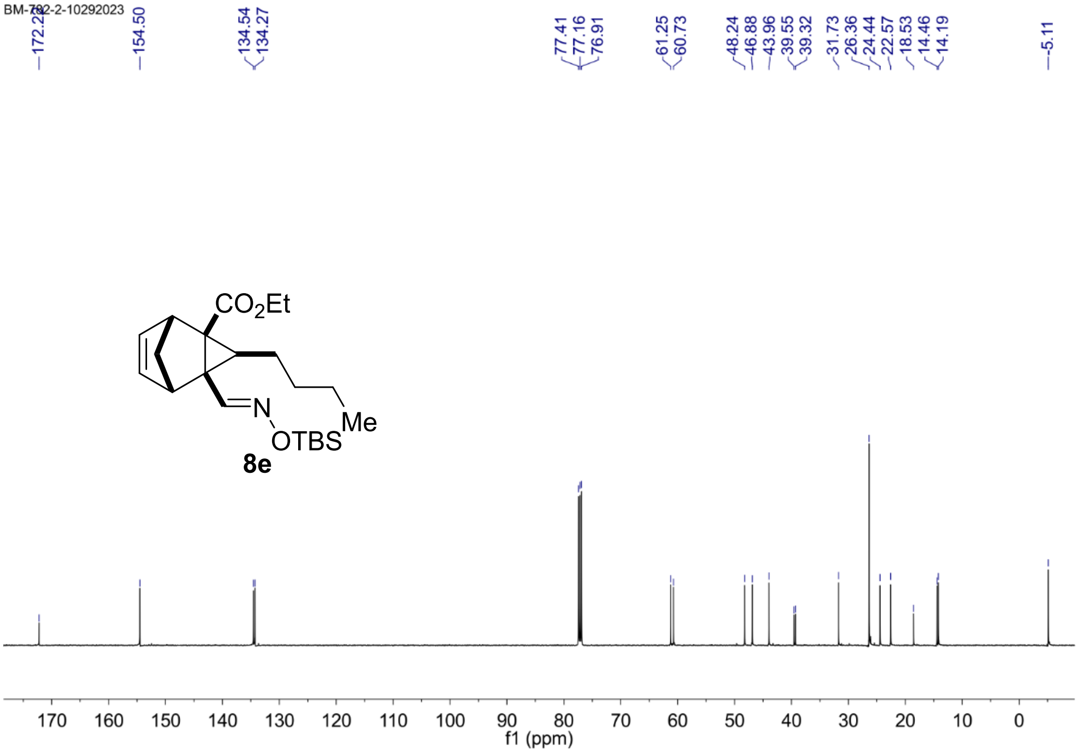
BM-782-2-10292023
CO2Et
N
OTBS
Me
8e
f1 (ppm)

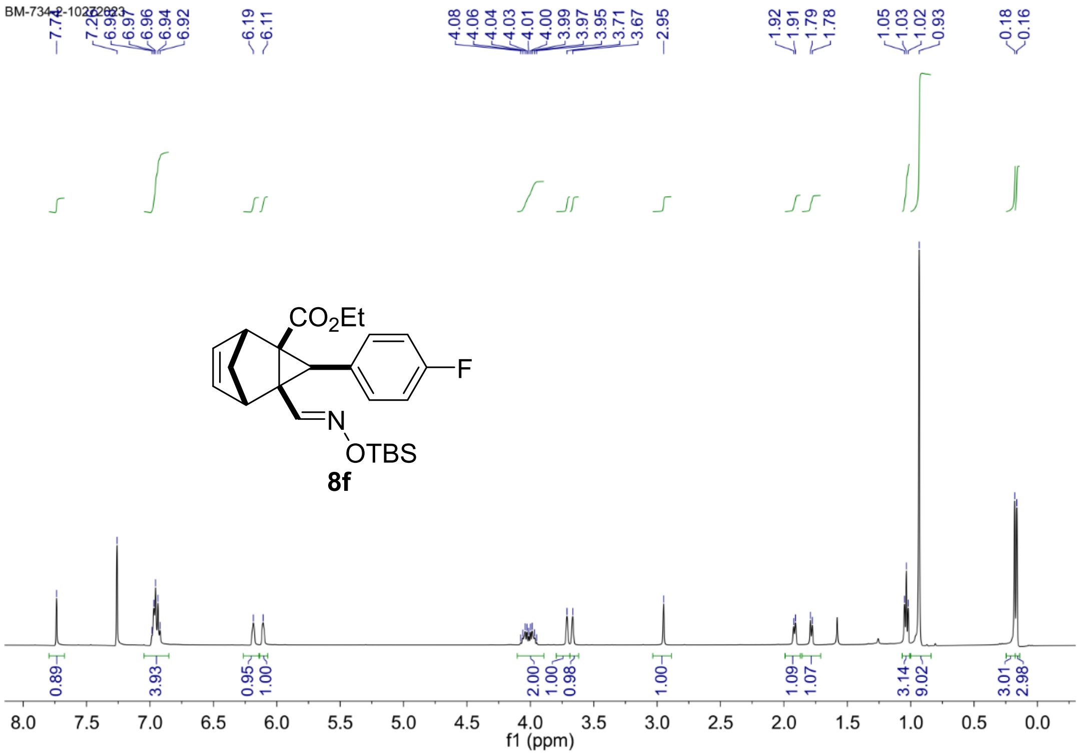

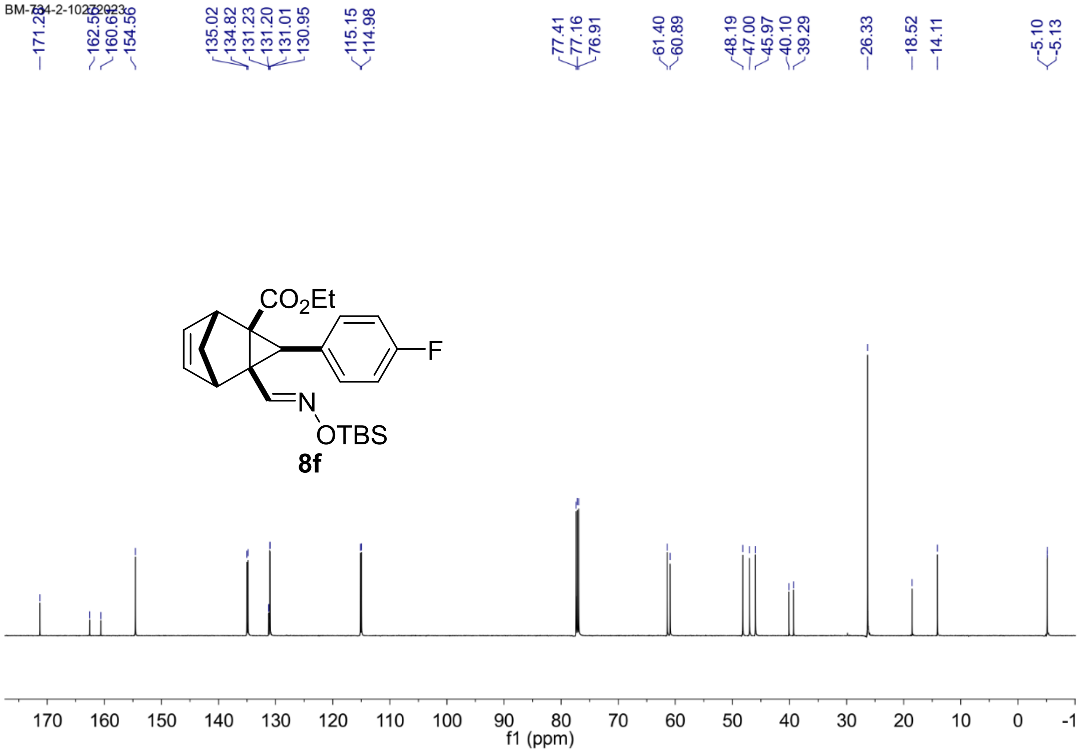

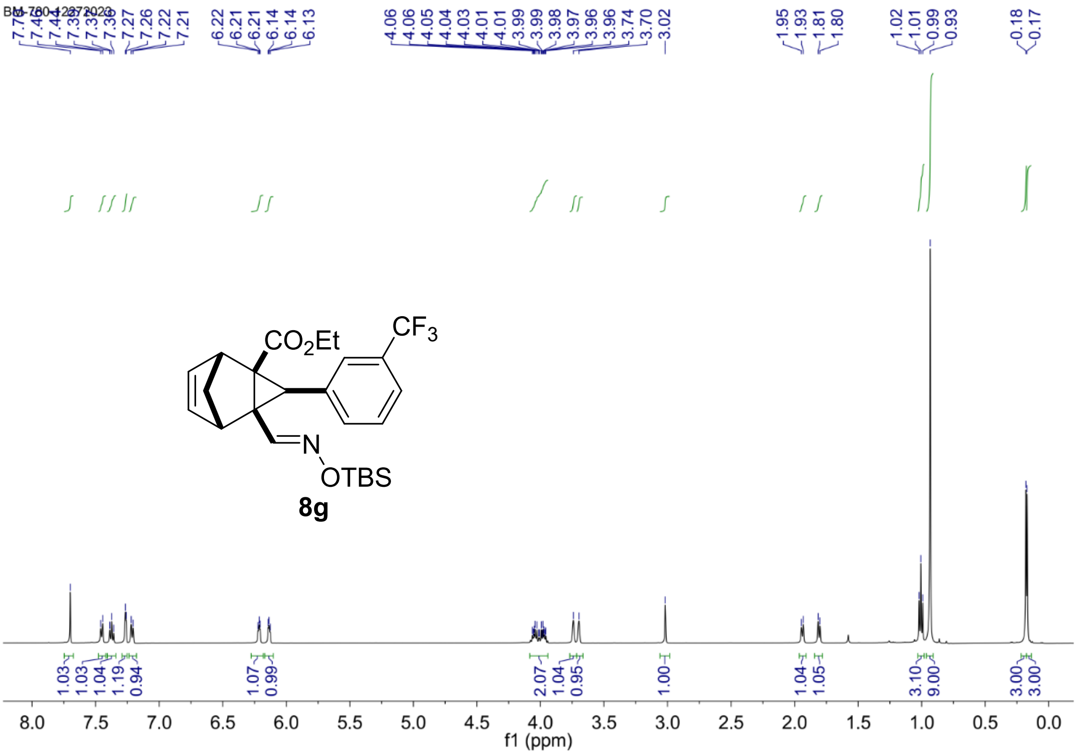
BM-780-12272023
CO2Et
CF3
N
OTBS
8g
f1 (ppm)

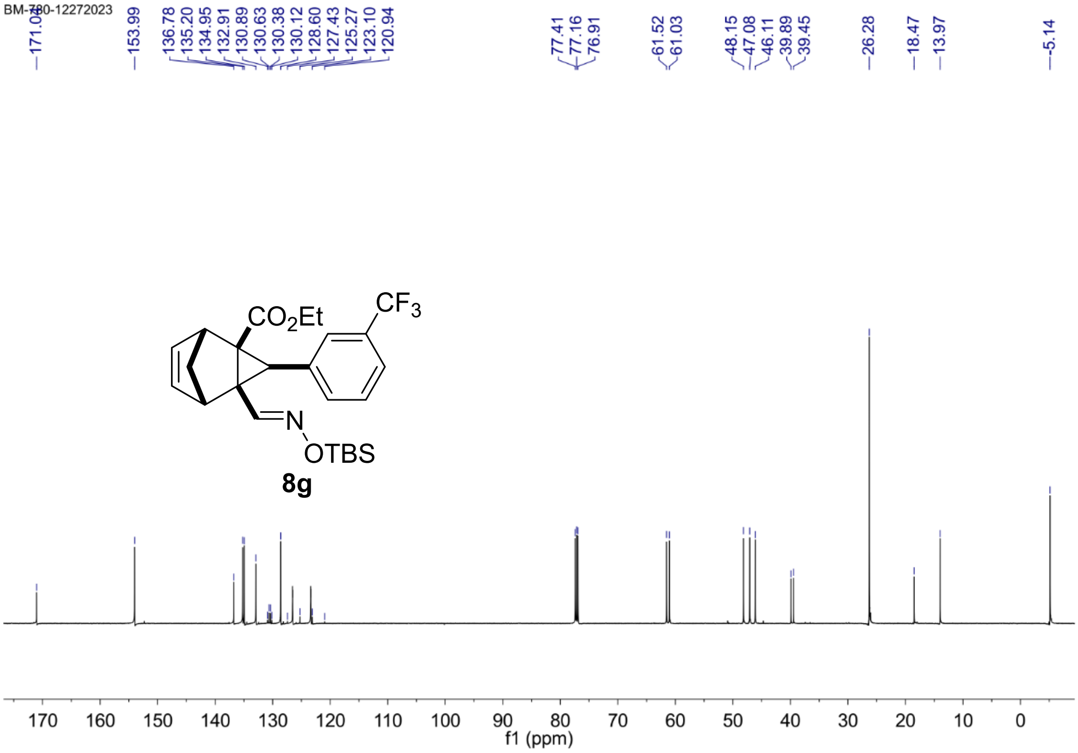
BM-780-12272023
CO2Et
CF3
N
OTBS
8g
f1 (ppm)

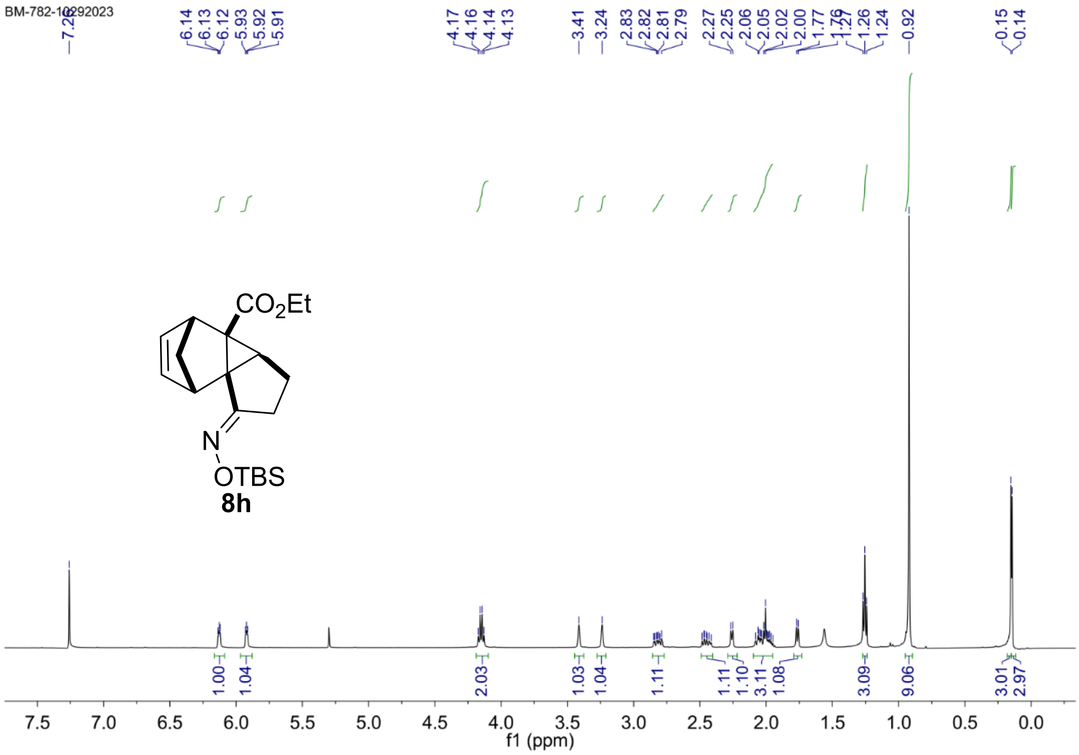
BM-782-10292023
8h
CO2Et
OTBS
f1 (ppm)

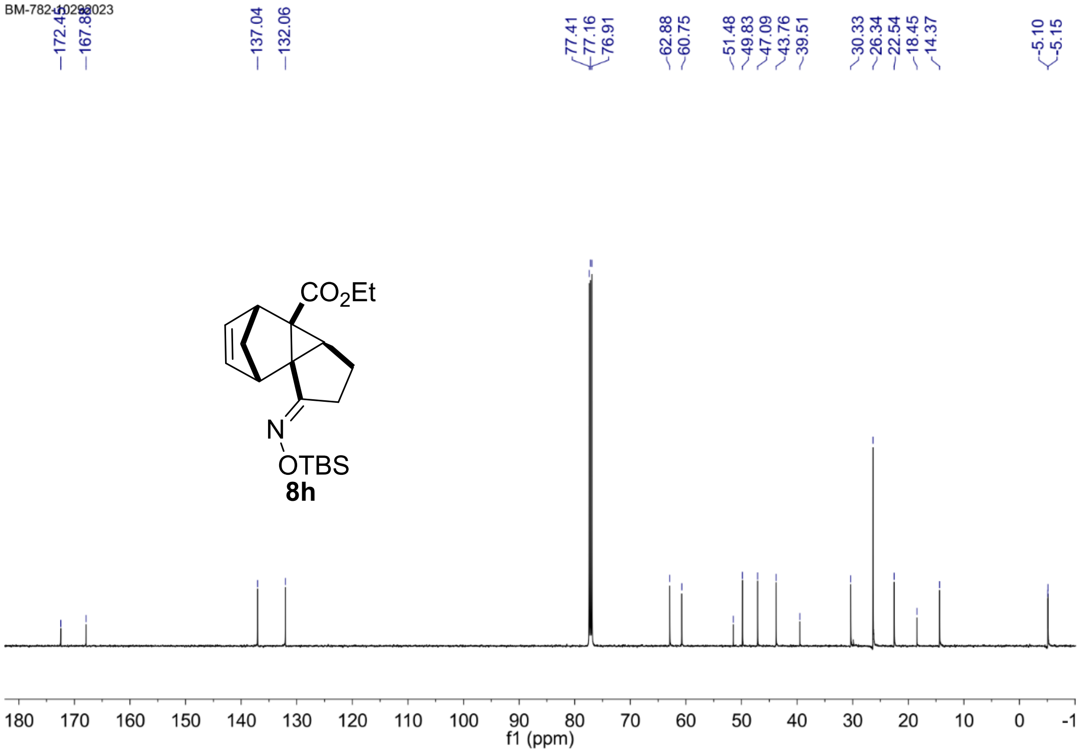
BM-782-10292023
8h
CO2Et
OTBS
f1 (ppm)

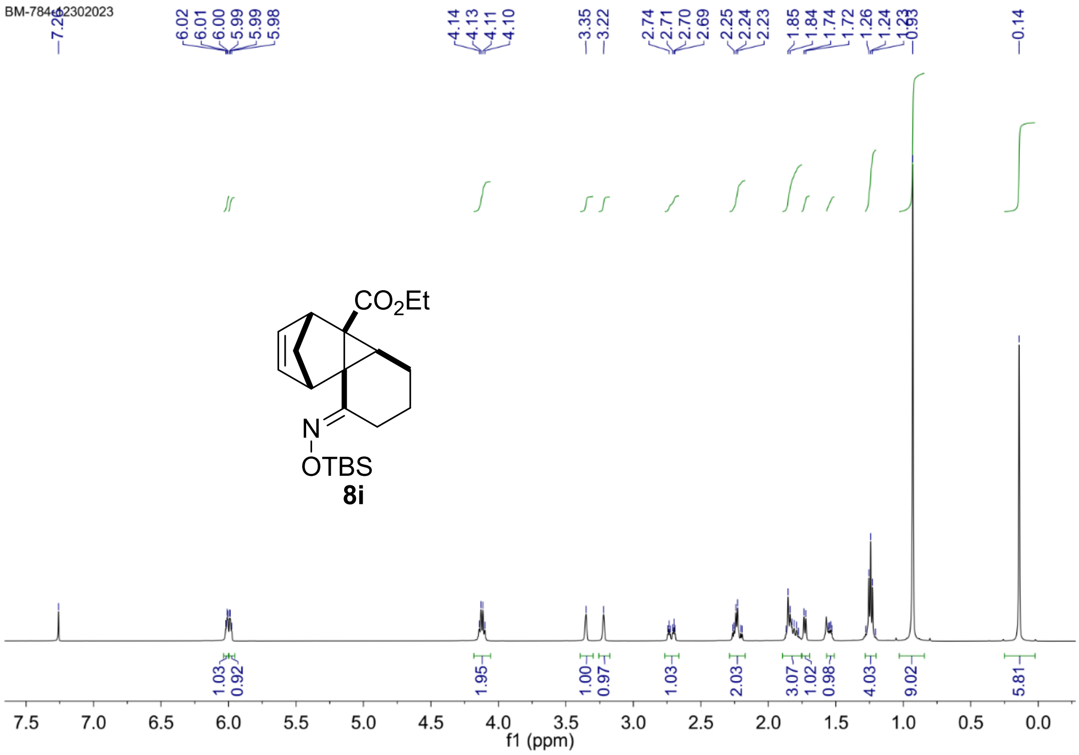
BM-784-12302023
CO2Et
N
OTBS
8i
f1 (ppm)

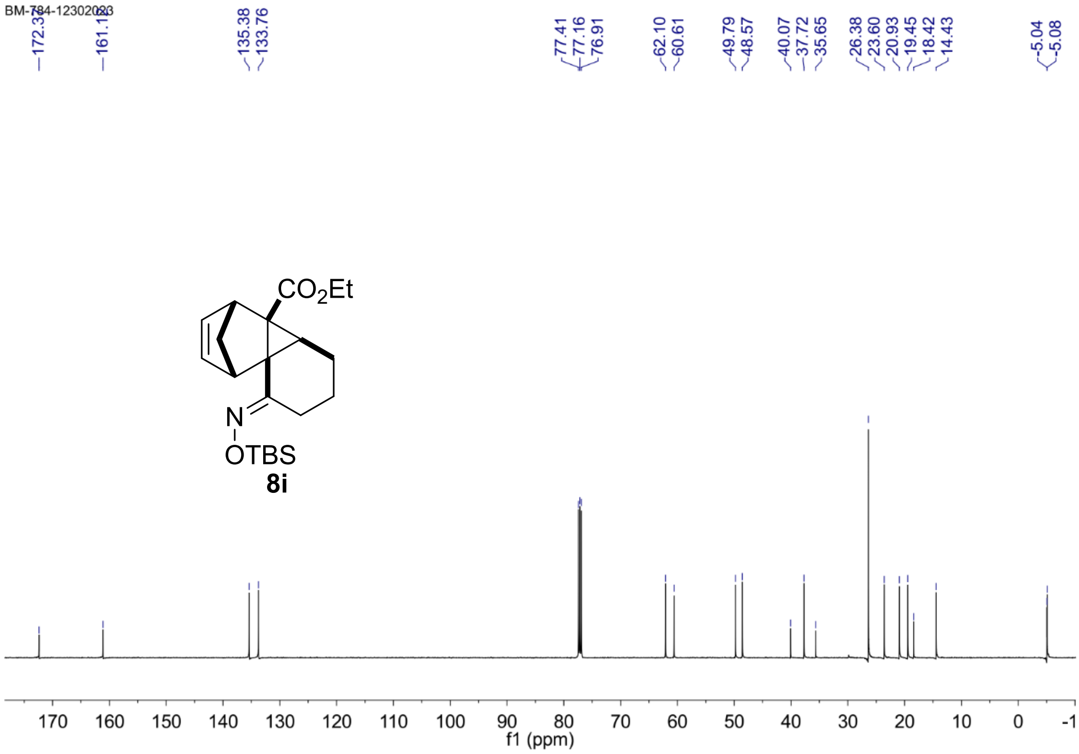
BM-784-12302023
CO2Et
N
OTBS
8i
f1 (ppm)

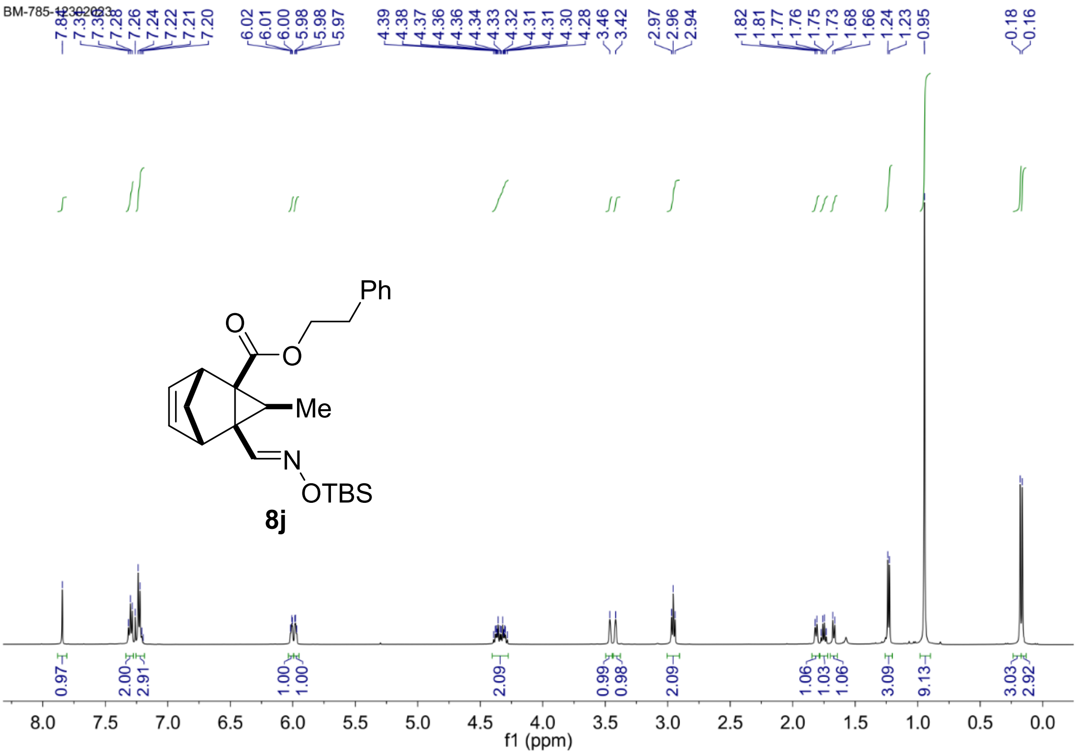
BM-785-12302023
7.33
7.28
7.26
7.24
7.22
7.21
7.20
6.02
6.01
6.00
5.98
5.98
5.97
4.39
4.38
4.37
4.36
4.36
4.34
4.33
4.32
4.31
4.31
4.30
4.28
3.46
3.42
2.97
2.96
2.94
1.82
1.81
1.77
1.76
1.75
1.73
1.68
1.66
1.24
1.23
0.95
0.18
0.16
Ph
O
O
Me
N
OTBS
8j
0.97
2.00
2.91
1.00
1.00
2.09
0.99
0.98
2.09
1.06
1.03
1.06
3.09
9.13
3.03
2.92
8.0
7.5
7.0
6.5
6.0
5.5
5.0
4.5
4.0
3.5
3.0
2.5
2.0
1.5
1.0
0.5
0.0
f1 (ppm)

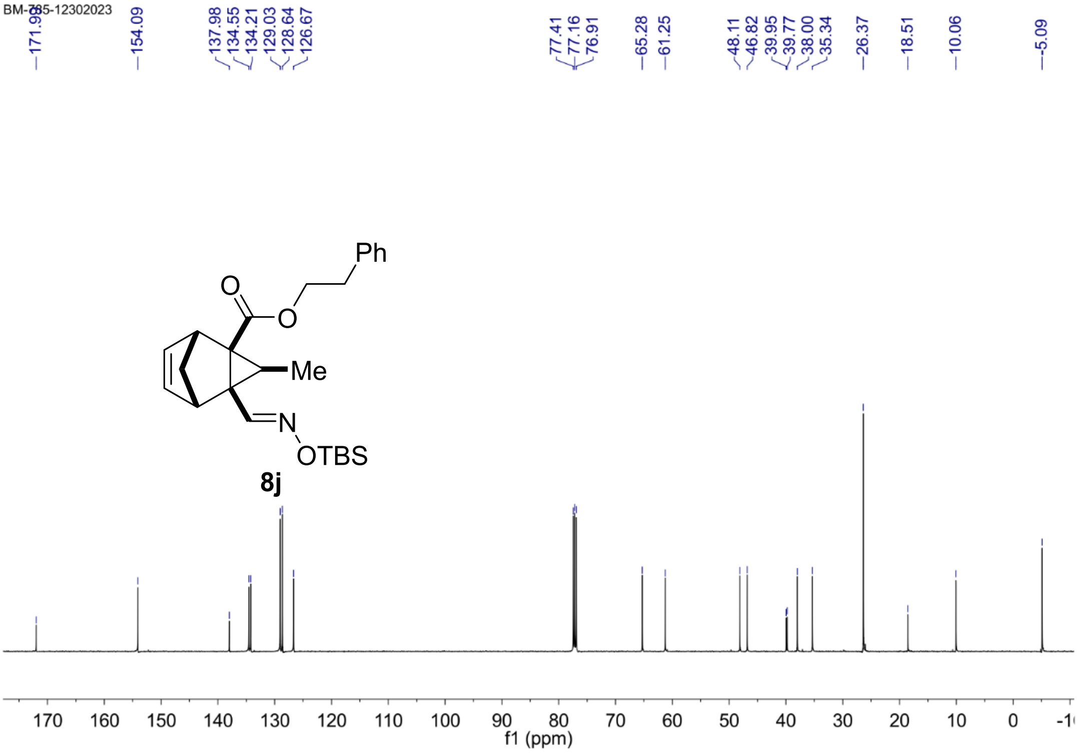
BM-785-12302023
154.09
137.98
134.55
134.21
129.03
128.64
126.67
77.41
77.16
76.91
65.28
61.25
48.11
46.82
39.95
39.77
38.00
35.34
26.37
18.51
10.06
-5.09
Ph
O
O
Me
N
OTBS
8j
170
160
150
140
130
120
110
100
90
80
70
60
50
40
30
20
10
0
f1 (ppm)

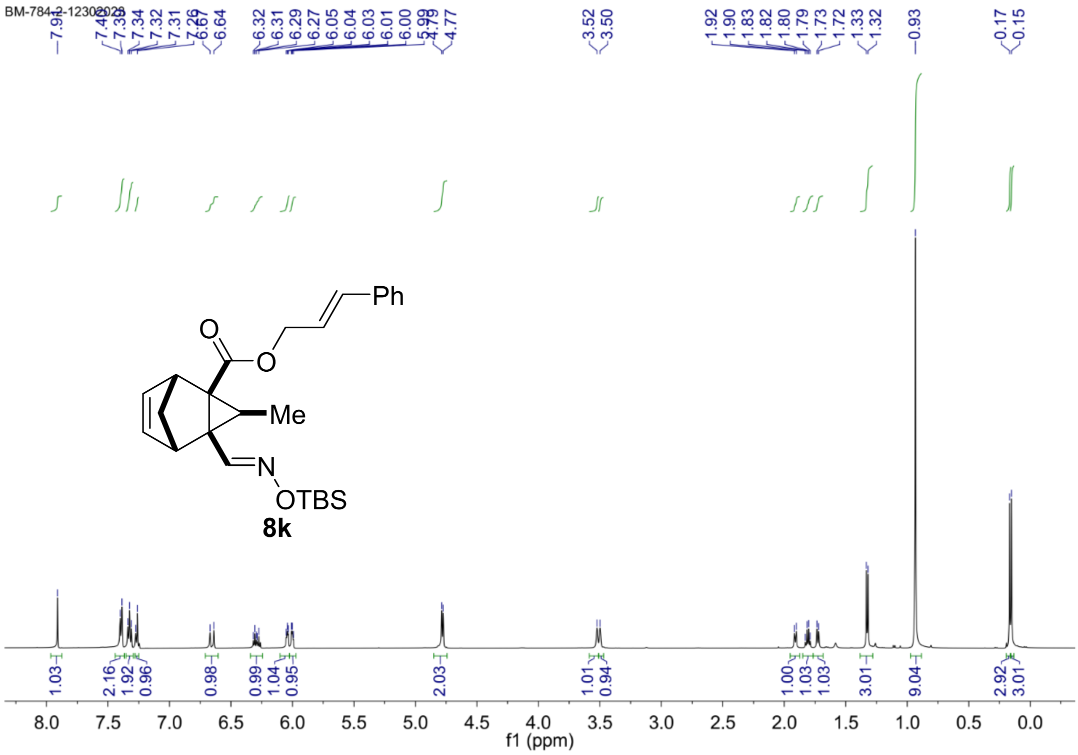
BM-784-2-12302023
Ph
O
O
Me
N
OTBS
8k
f1 (ppm)

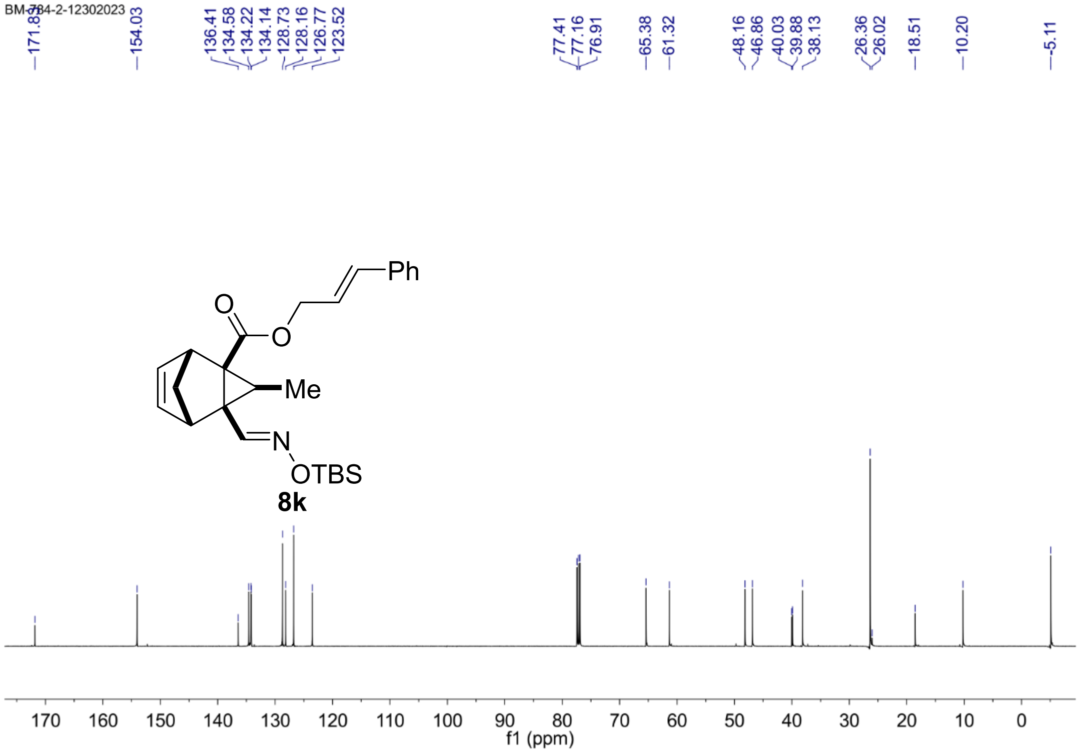
BM-784-2-12302023
Ph
O
O
Me
N
OTBS
8k
f1 (ppm)

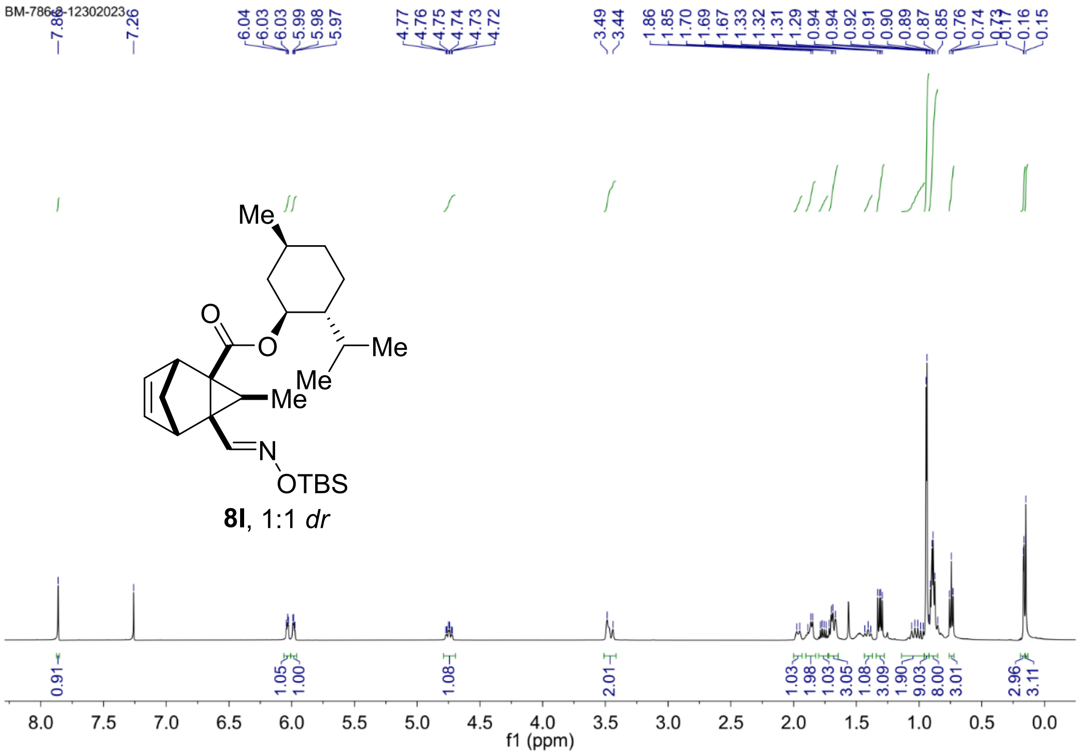
BM-786-2-12302023
Me
O
O
Me
Me
Me
N
OTBS
8l, 1:1 dr
f1 (ppm)

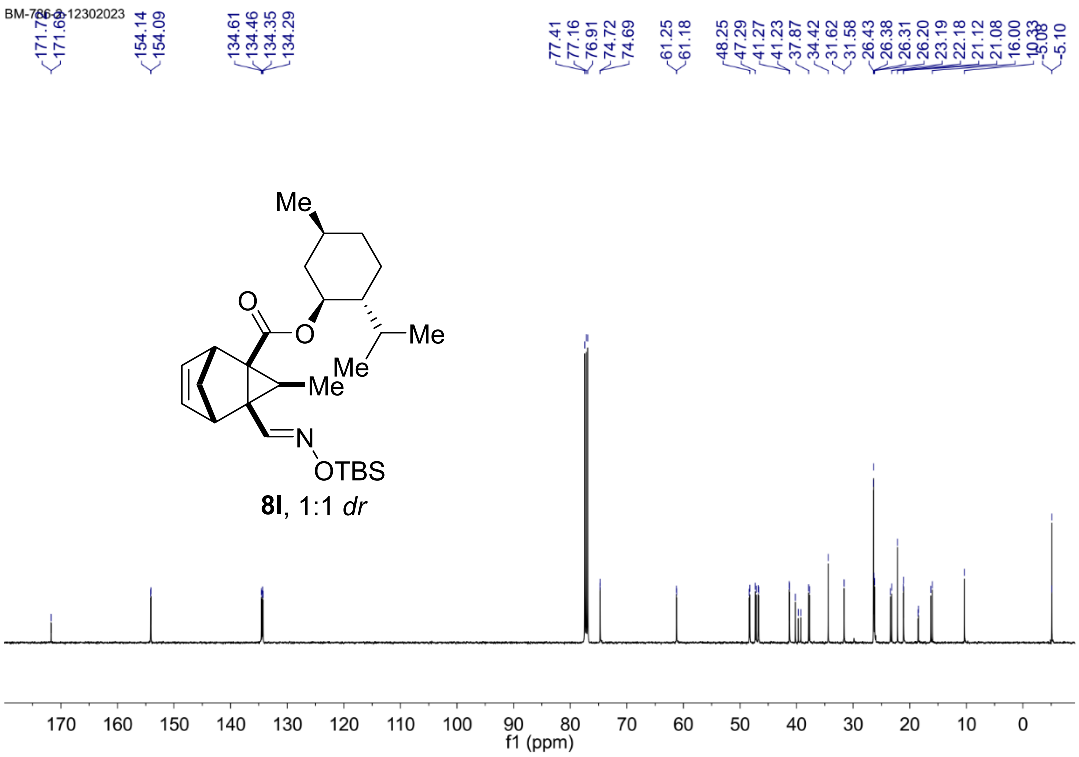
BM-786-2-12302023
Me
O
O
Me
Me
Me
N
OTBS
8l, 1:1 dr
f1 (ppm)

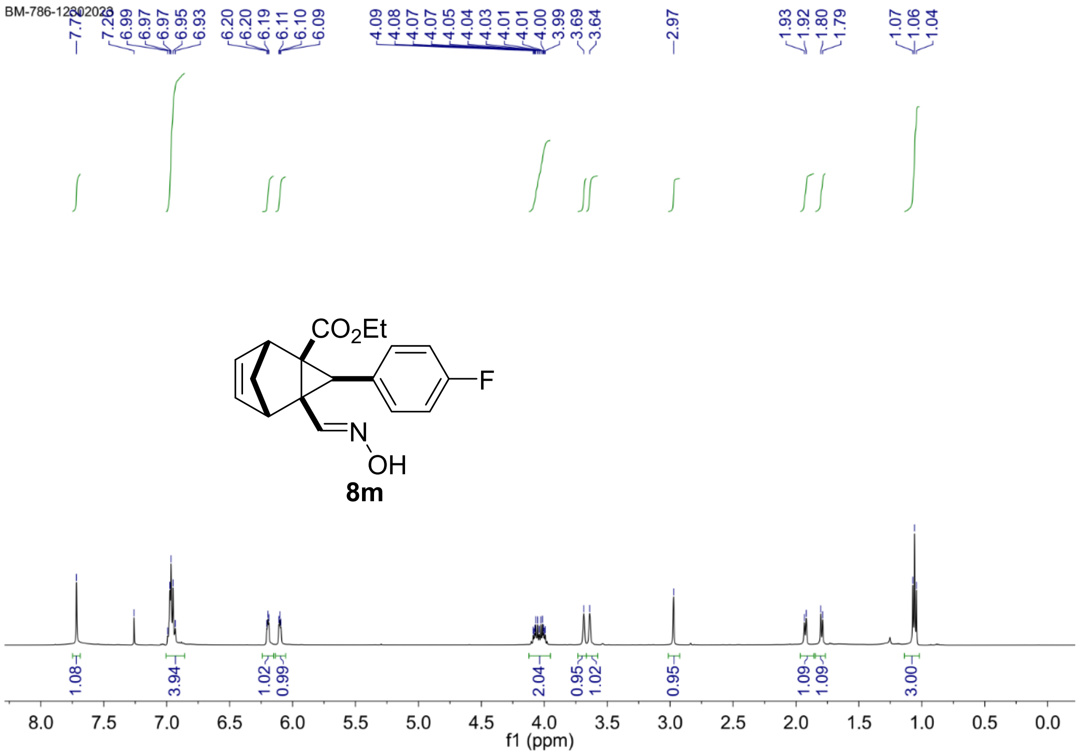
BM-786-12302023
CO2Et
F
N
OH
8m
f1 (ppm)

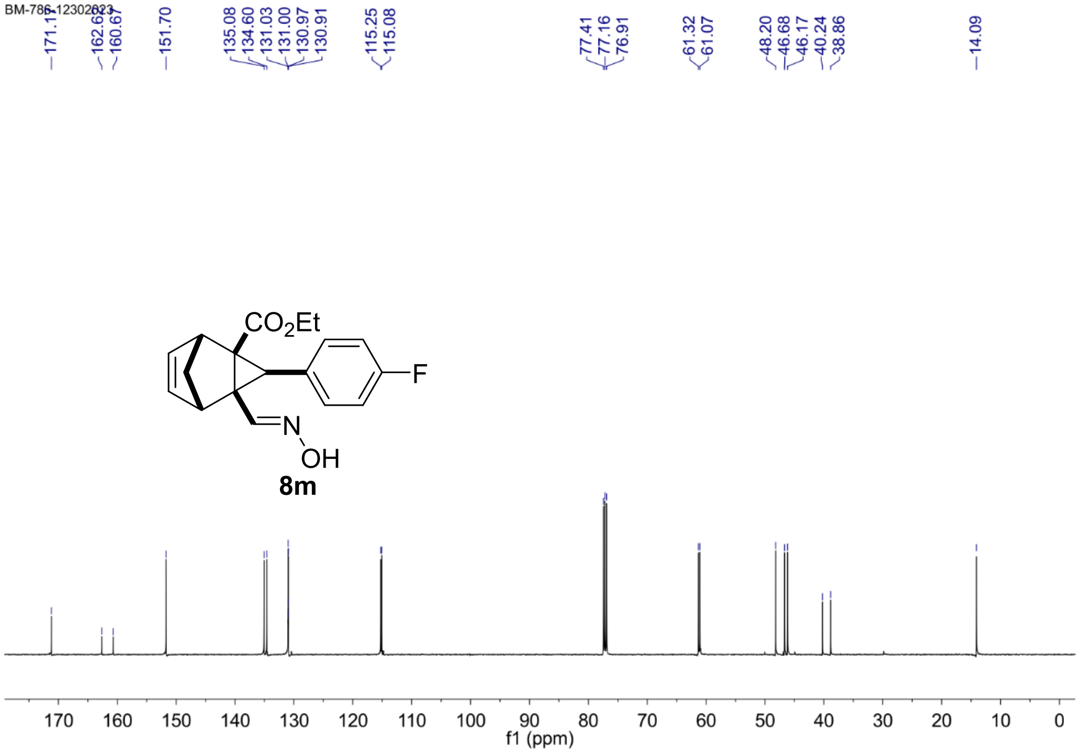
BM-786-12302023
CO2Et
F
N
OH
8m
f1 (ppm)

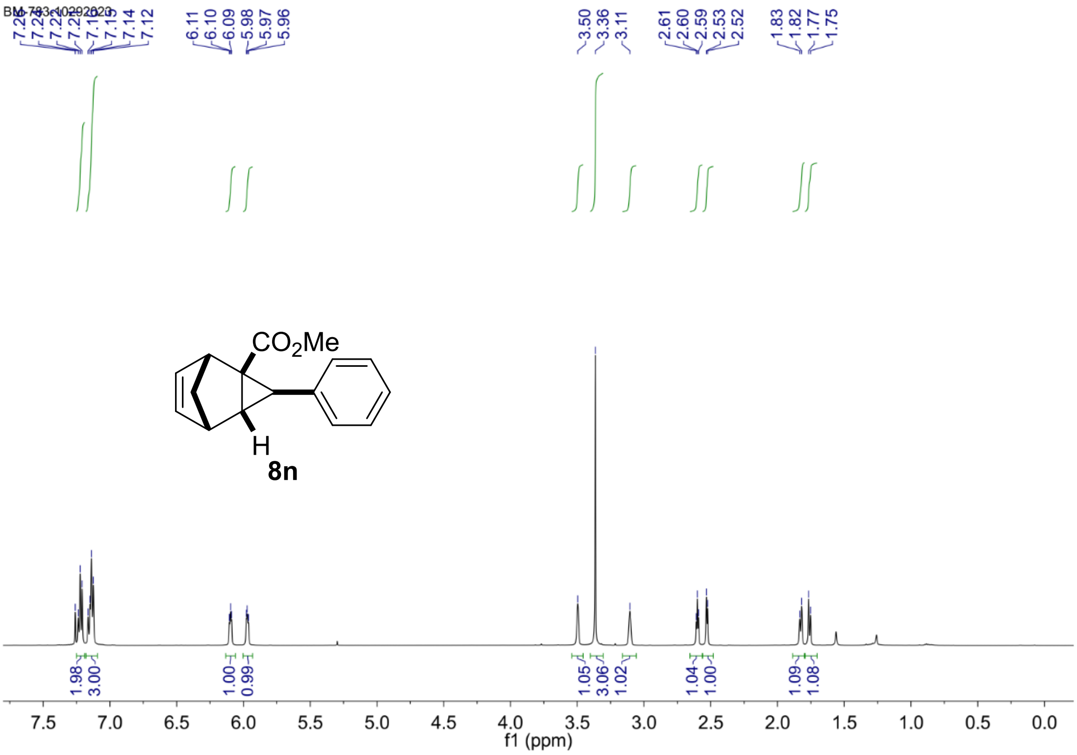
BM-783-10292023
6.11
6.10
6.09
5.98
5.97
5.96
3.50
3.36
3.11
2.61
2.60
2.59
2.53
2.52
1.83
1.82
1.77
1.75
CO2Me
H
8n
1.98
3.00
1.00
0.99
1.05
3.06
1.02
1.04
1.00
1.09
1.08
7.5
7.0
6.5
6.0
5.5
5.0
4.5
4.0
3.5
3.0
2.5
2.0
1.5
1.0
0.5
0.0
f1 (ppm)

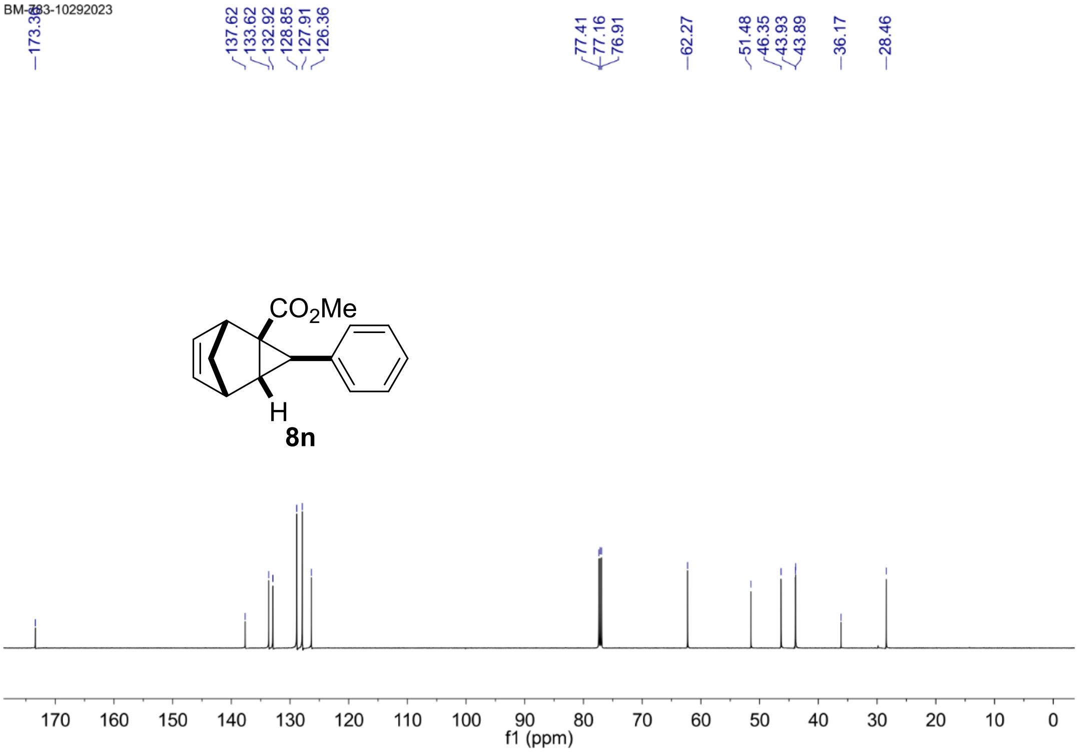
BM-783-10292023
173.38
137.62
133.62
132.92
128.85
127.91
126.36
77.41
77.16
76.91
62.27
51.48
46.35
43.93
43.89
36.17
28.46
CO2Me
H
8n
170
160
150
140
130
120
110
100
90
80
70
60
50
40
30
20
10
0
f1 (ppm)

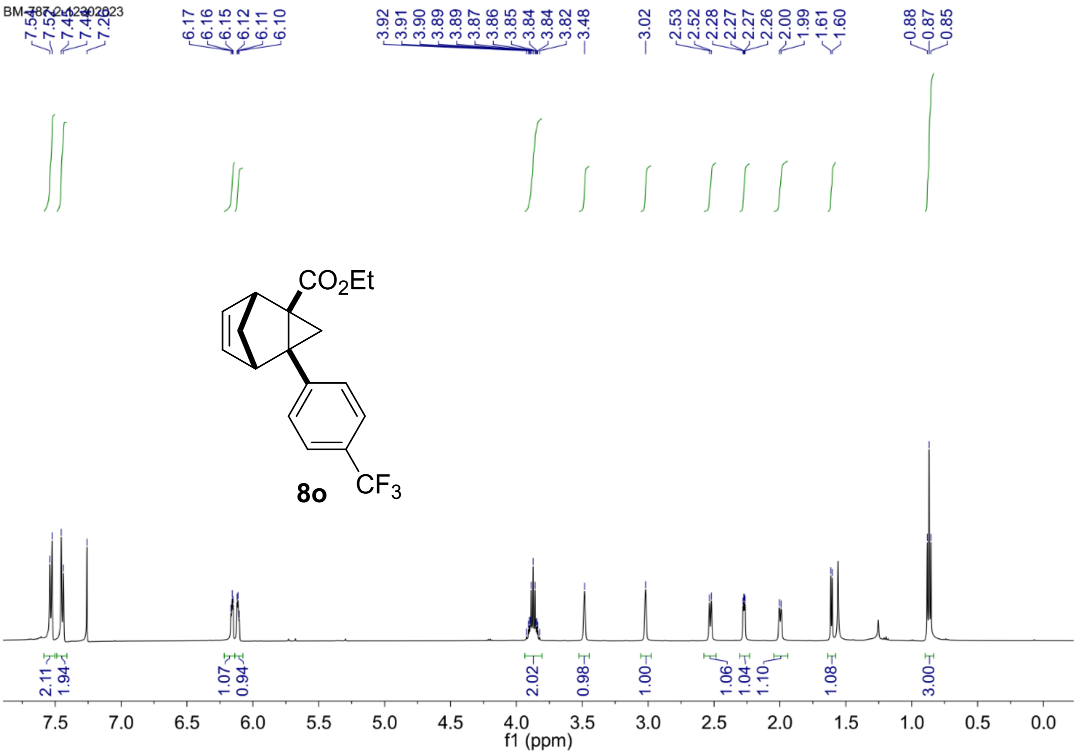
BM-787-2-12302023
7.54
7.52
7.44
7.42
7.26
6.17
6.16
6.15
6.12
6.11
6.10
3.92
3.91
3.90
3.89
3.89
3.87
3.86
3.85
3.84
3.84
3.82
3.48
3.02
2.53
2.52
2.28
2.27
2.27
2.26
2.00
1.99
1.61
1.60
0.88
0.87
0.85
CO2Et
8o
CF3
2.11
1.94
1.07
0.94
2.02
0.98
1.00
1.06
1.04
1.10
1.08
3.00
7.5
7.0
6.5
6.0
5.5
5.0
4.5
4.0
3.5
3.0
2.5
2.0
1.5
1.0
0.5
0.0
f1 (ppm)

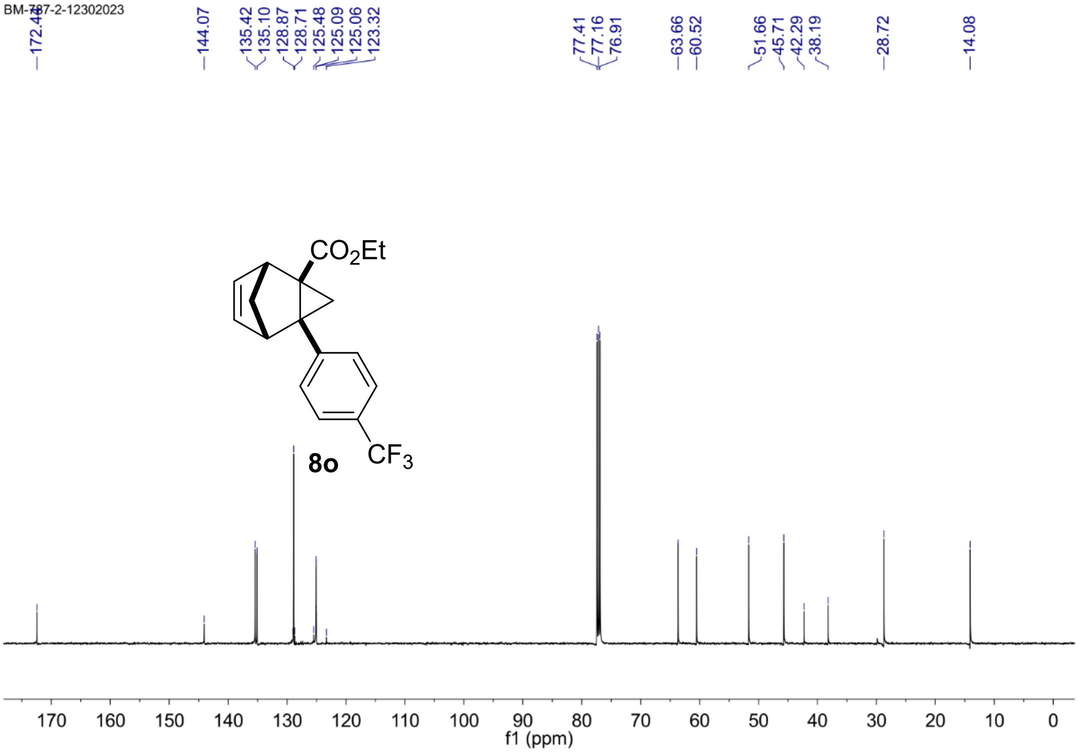
BM-787-2-12302023
172.48
144.07
135.42
135.10
128.87
128.71
125.48
125.09
125.06
123.32
77.41
77.16
76.91
63.66
60.52
51.66
45.71
42.29
38.19
28.72
14.08
CO2Et
8o
CF3
170
160
150
140
130
120
110
100
90
80
70
60
50
40
30
20
10
0
f1 (ppm)

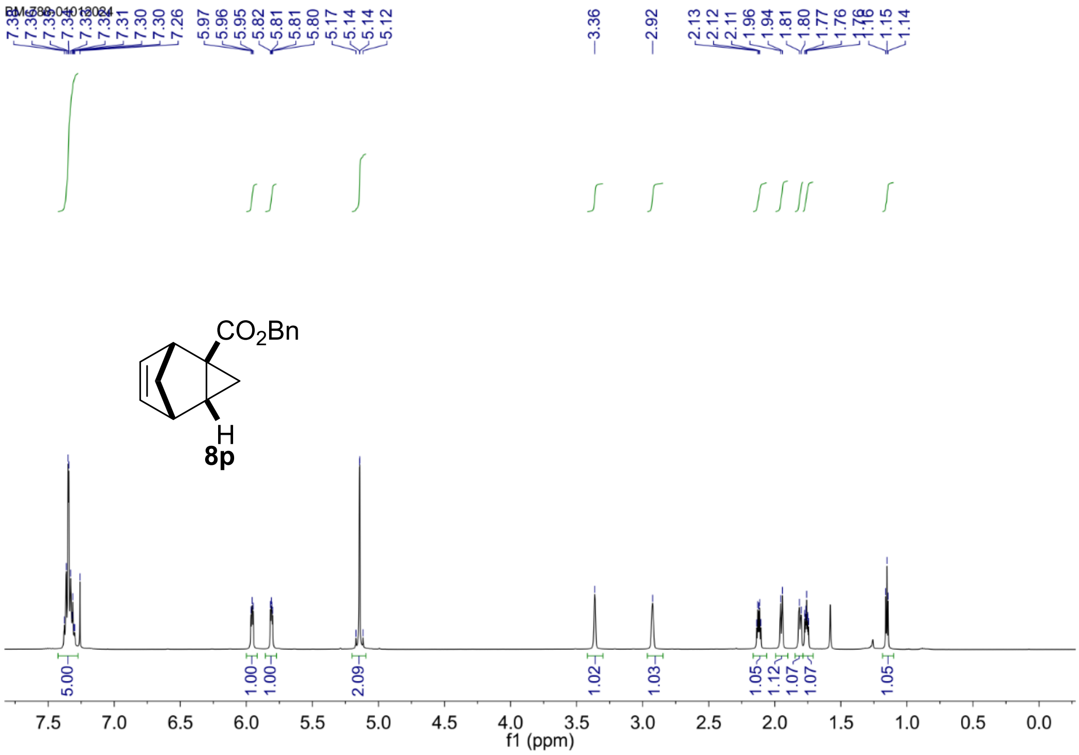

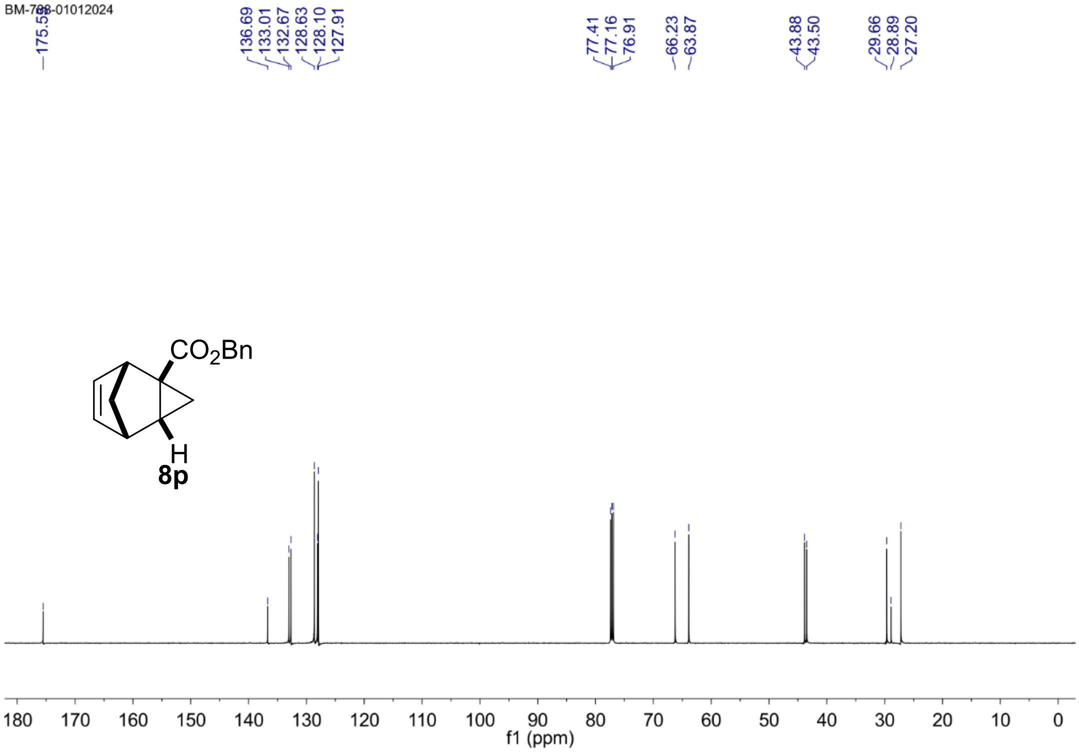

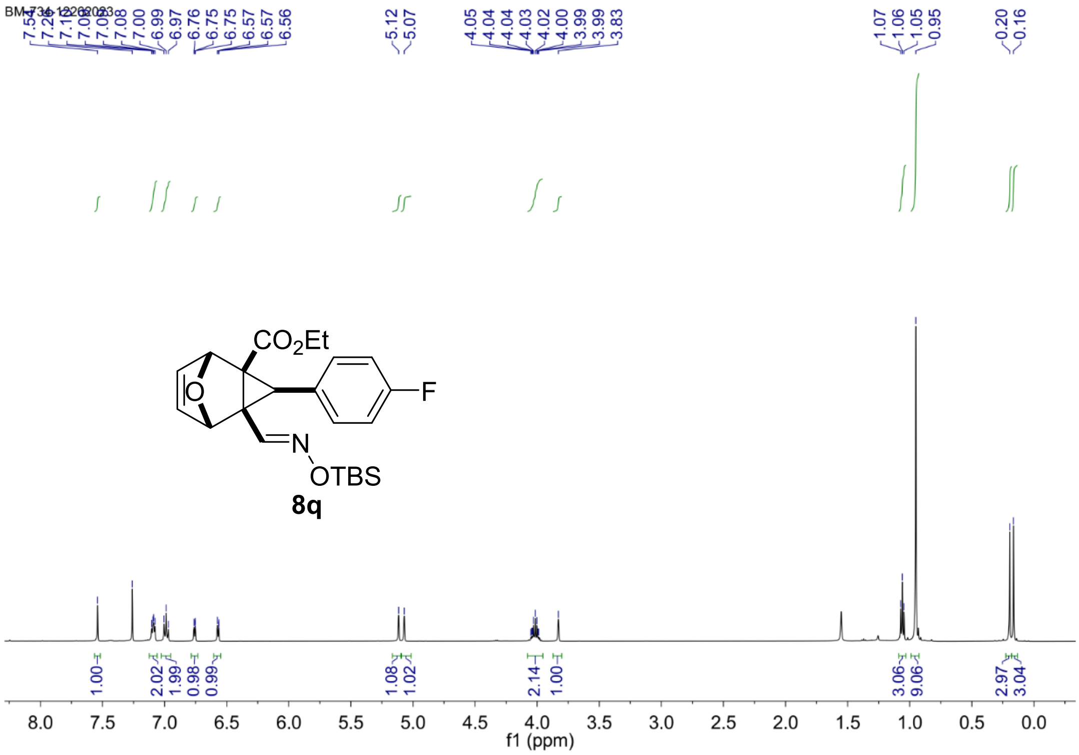
BM-734-12262023
8q
CO2Et
F
N
OTBS
O
f1 (ppm)

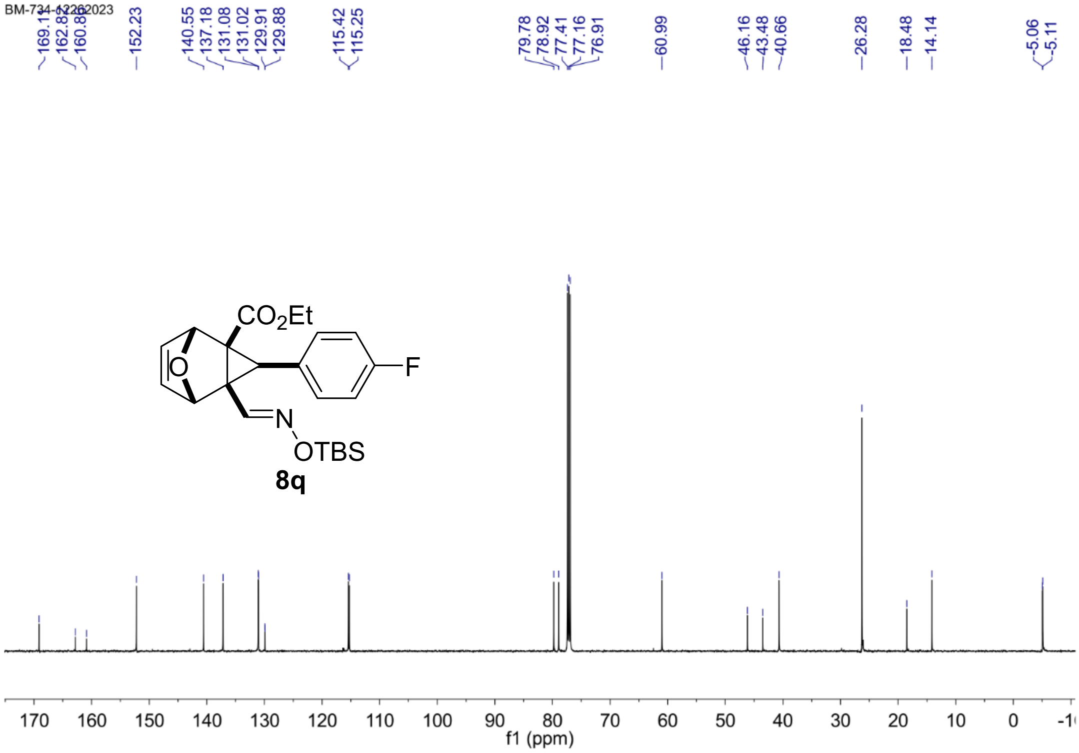
BM-734-12262023
8q
CO2Et
F
N
OTBS
O
f1 (ppm)

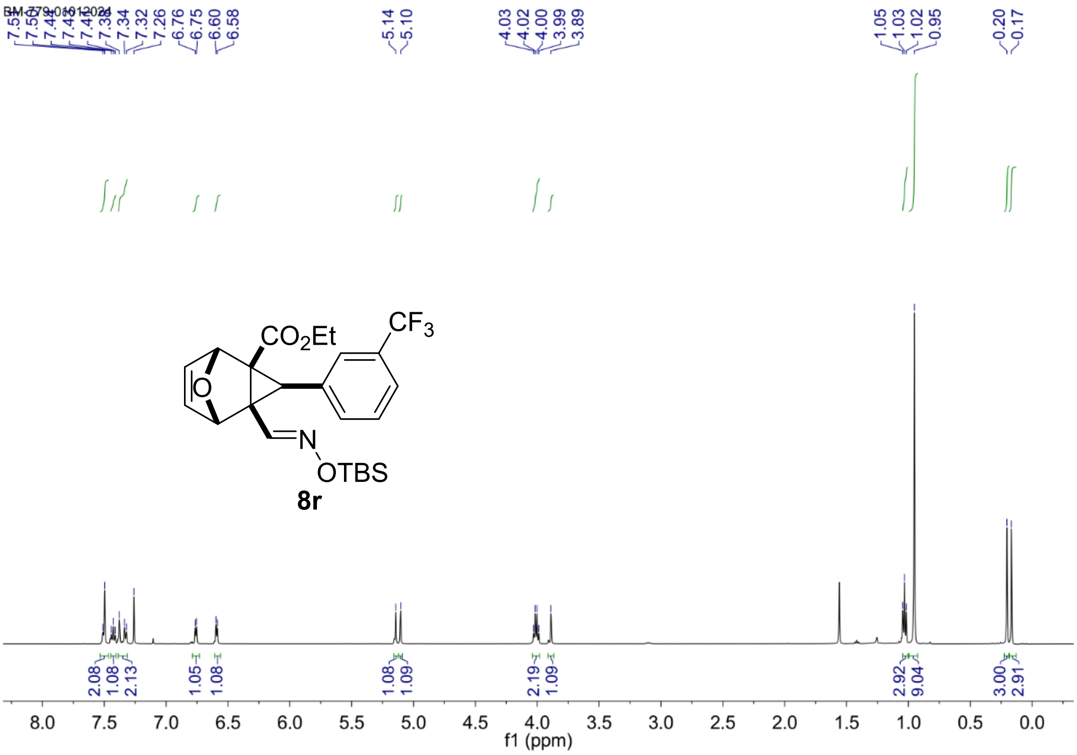
BM-779-01012024
8r
f1 (ppm)

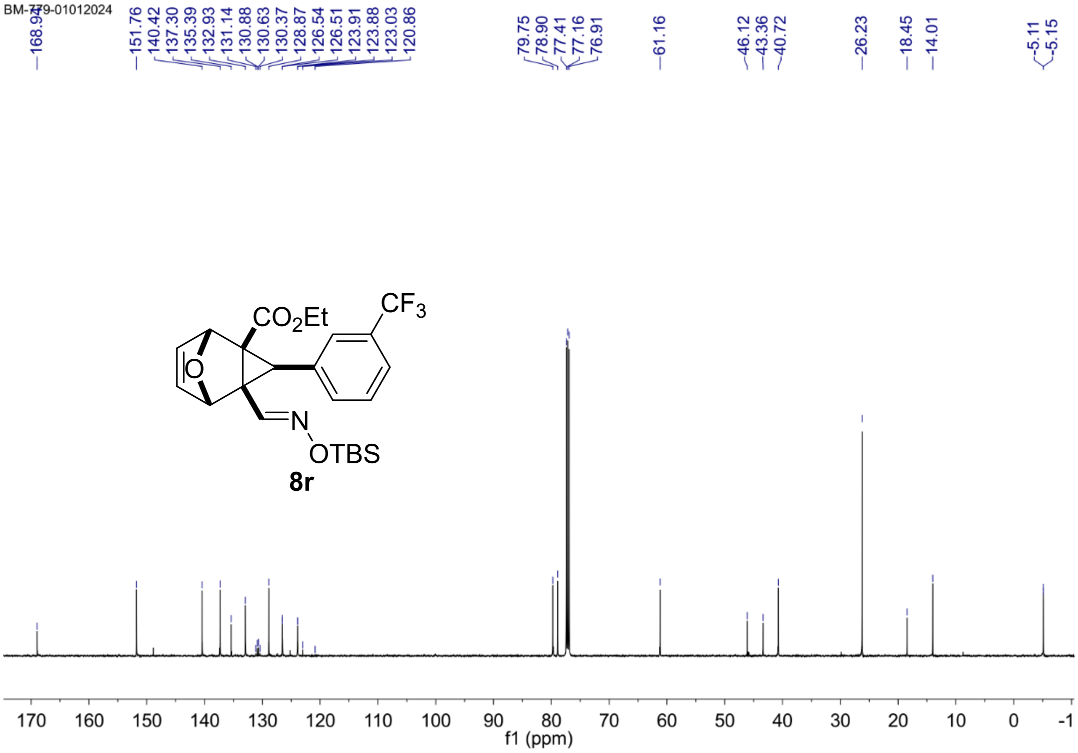
BM-779-01012024
8r
f1 (ppm)

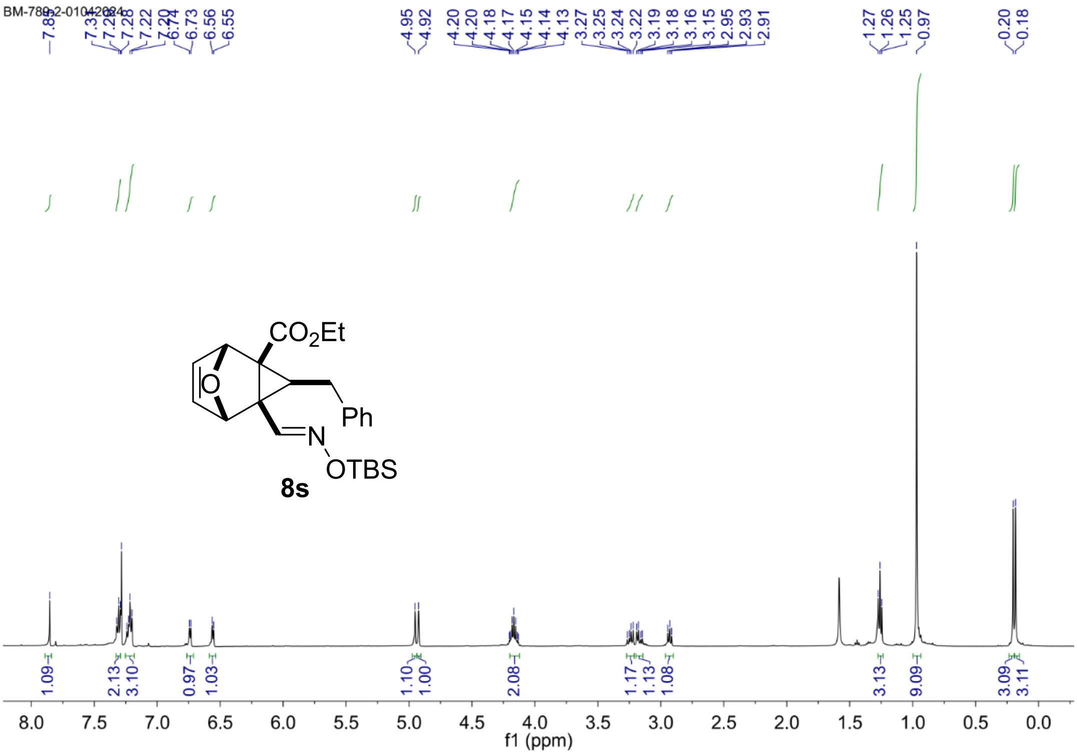

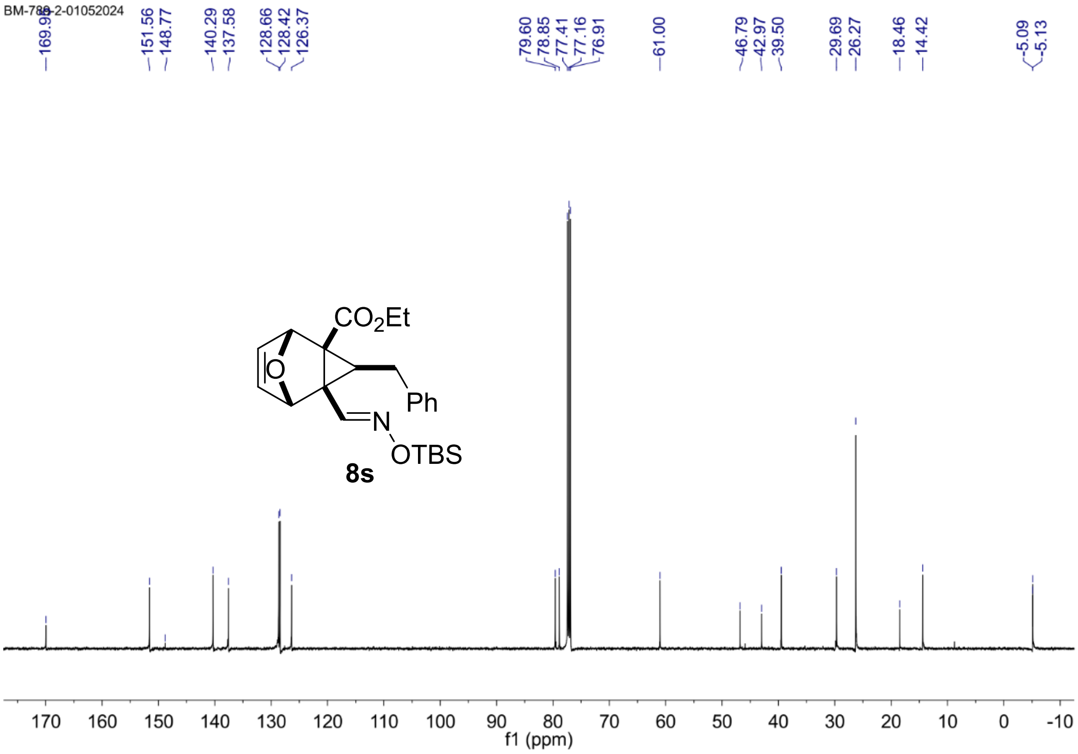

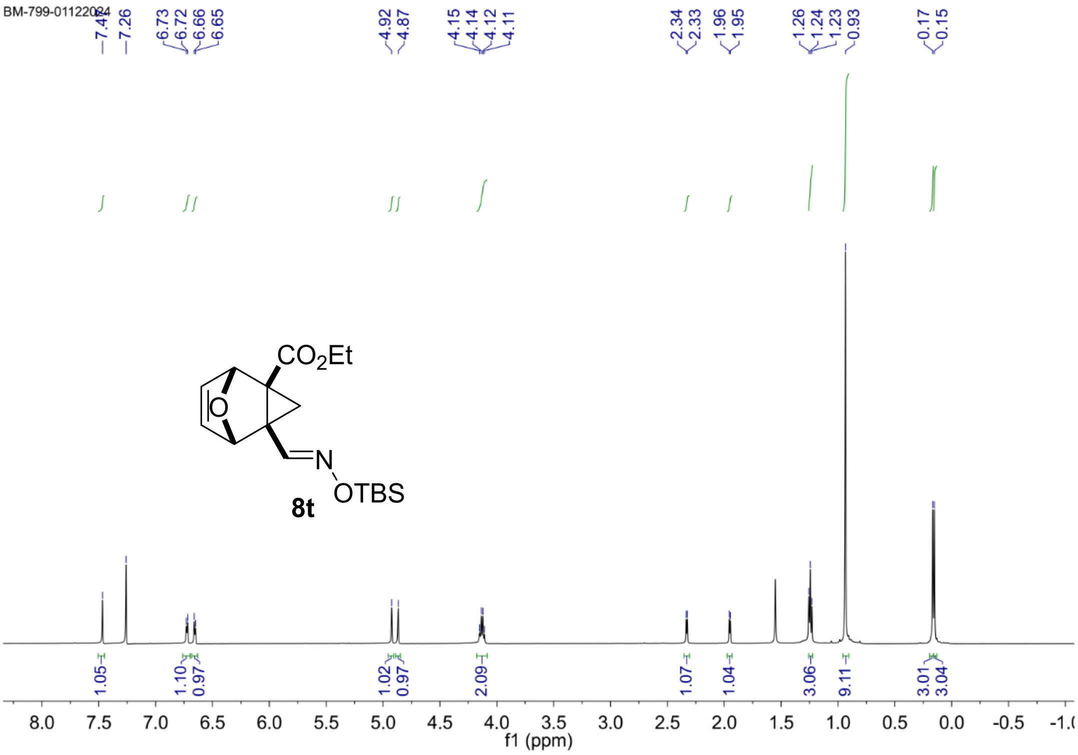

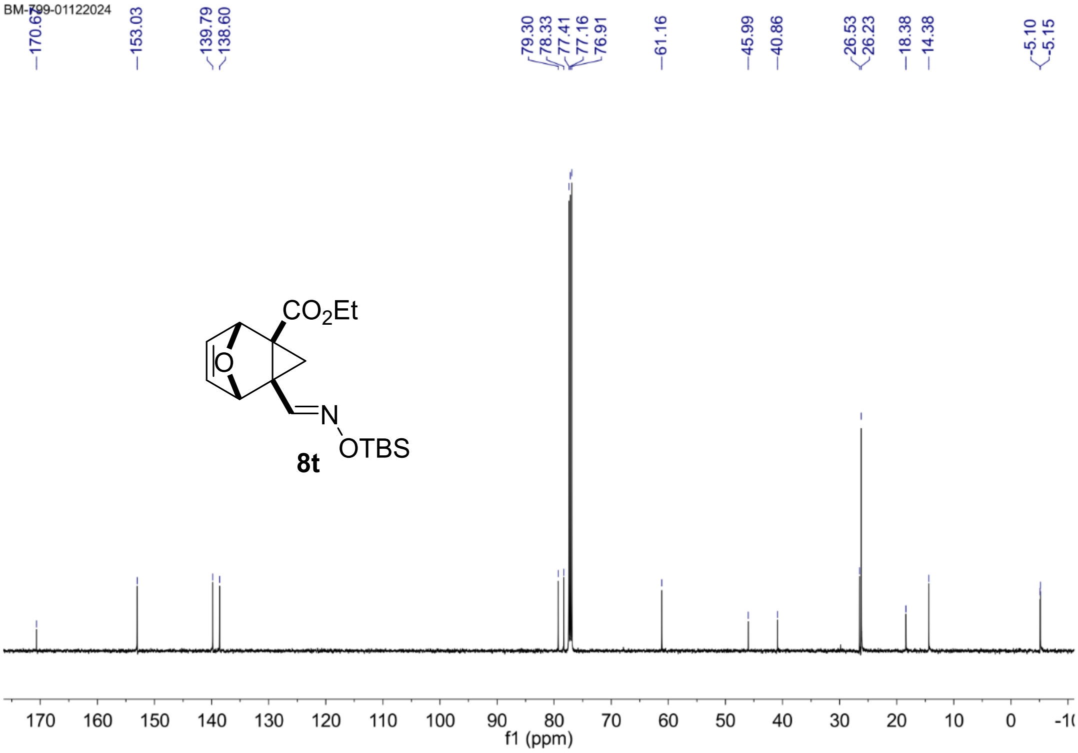

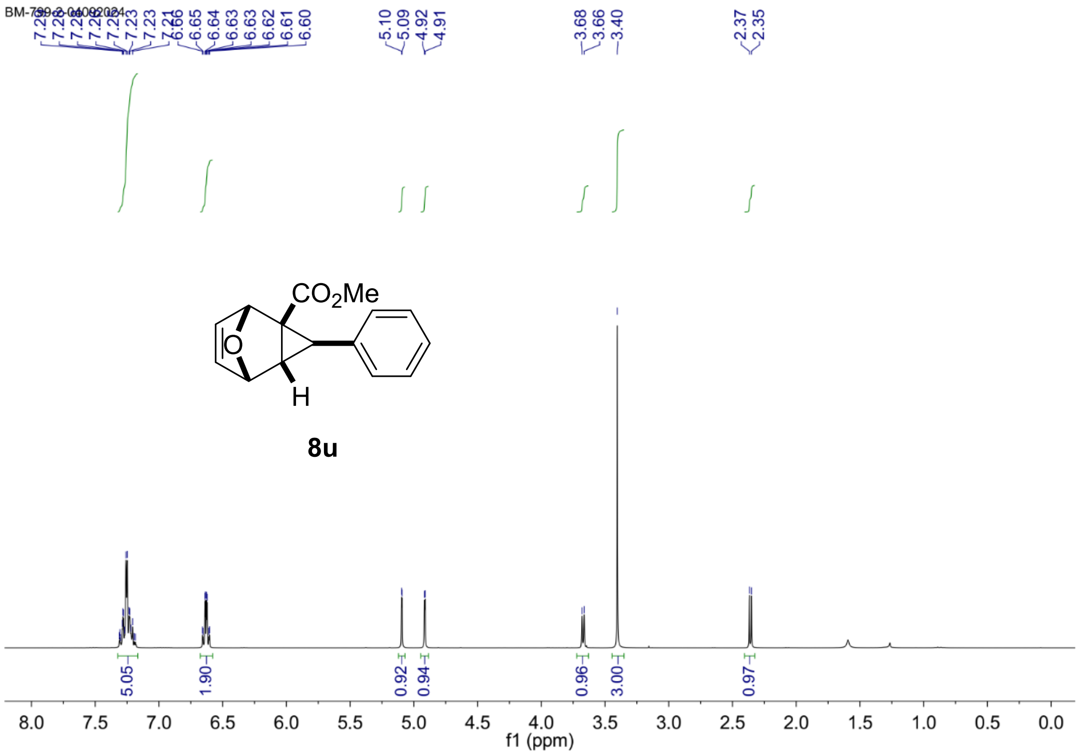

8u

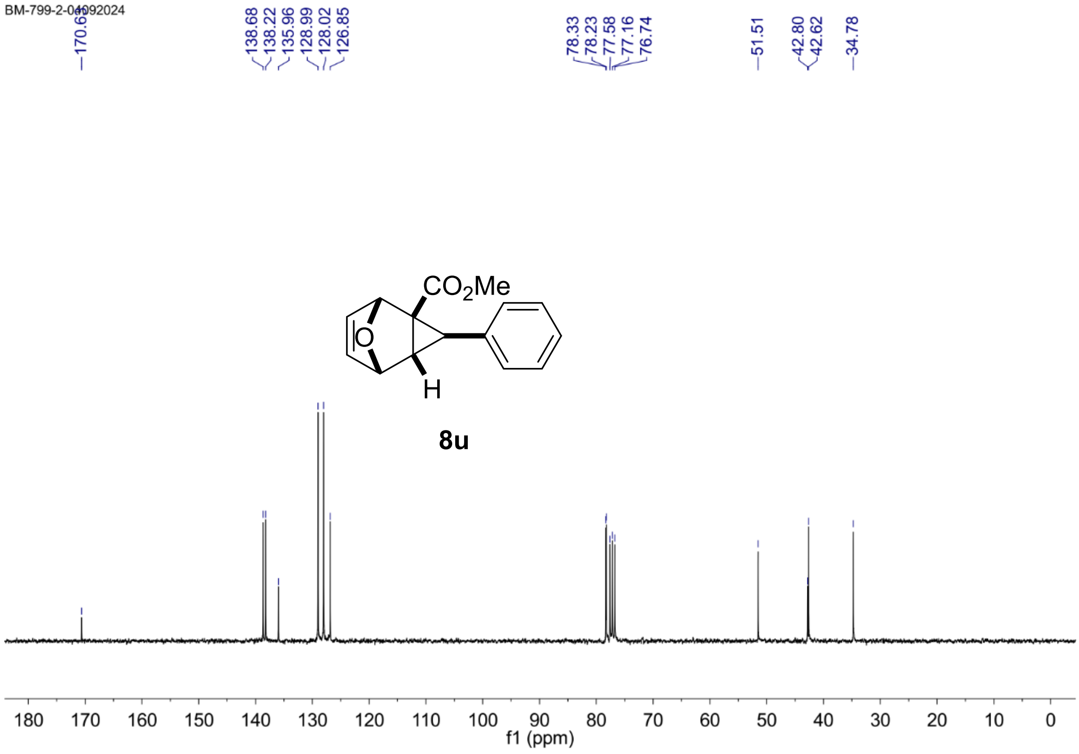

8u

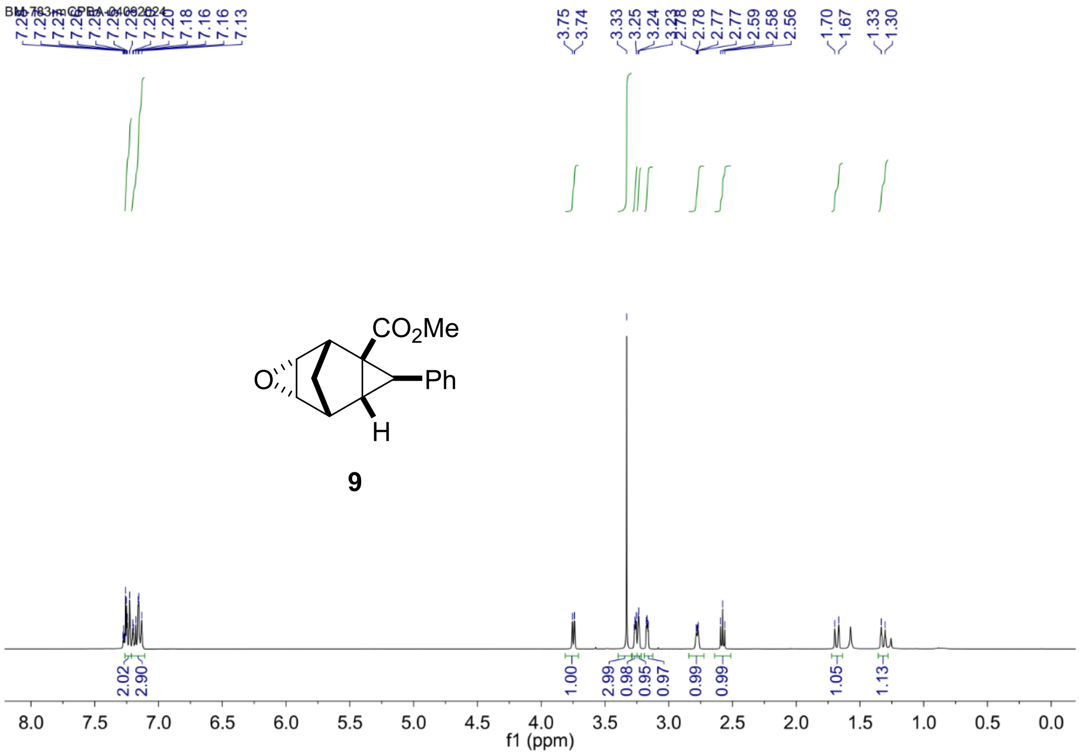

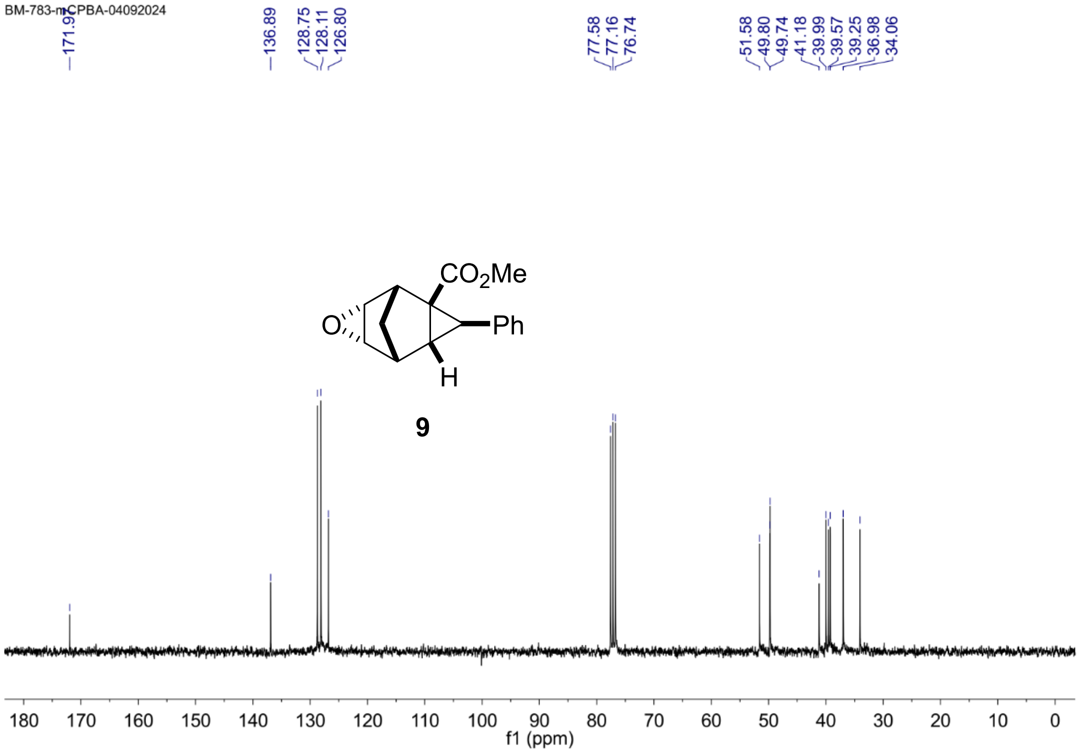

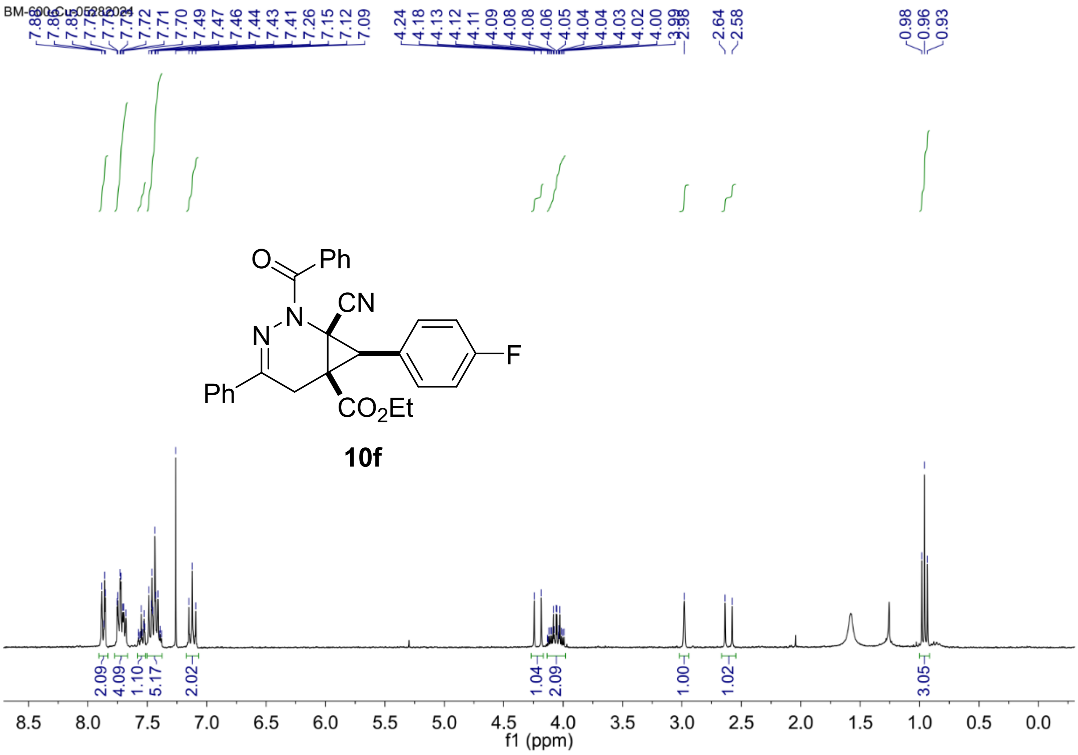
BM-600-Cu-05282024
10f
f1 (ppm)

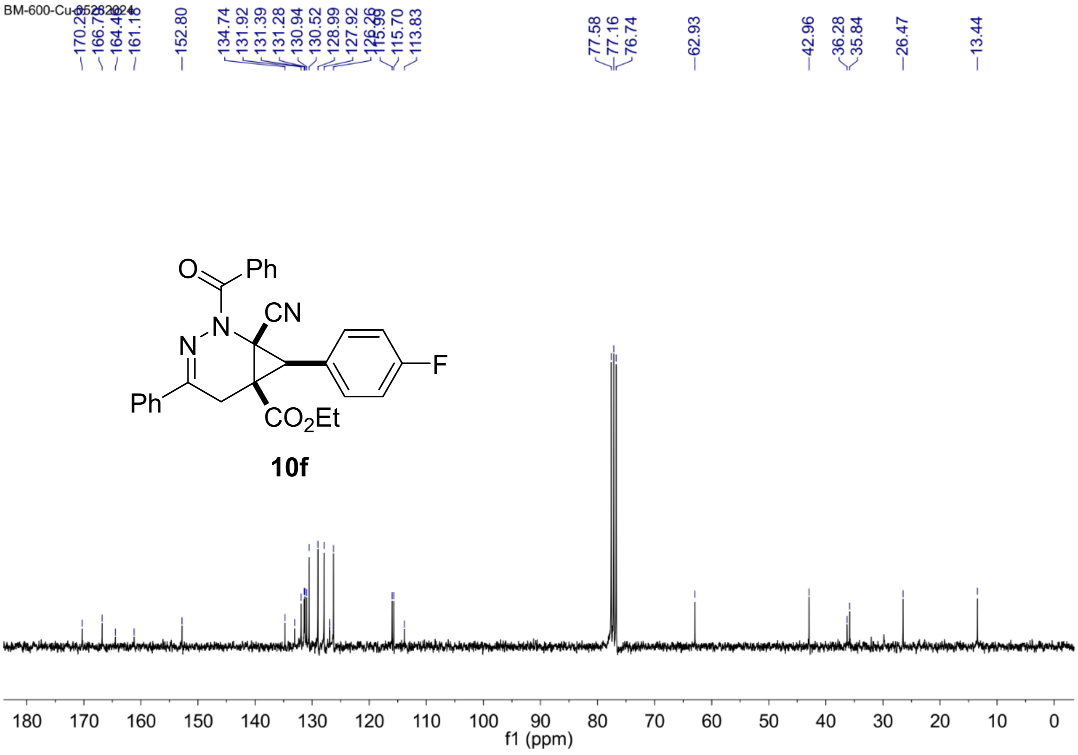
BM-600-Cu-05282024
10f
f1 (ppm)

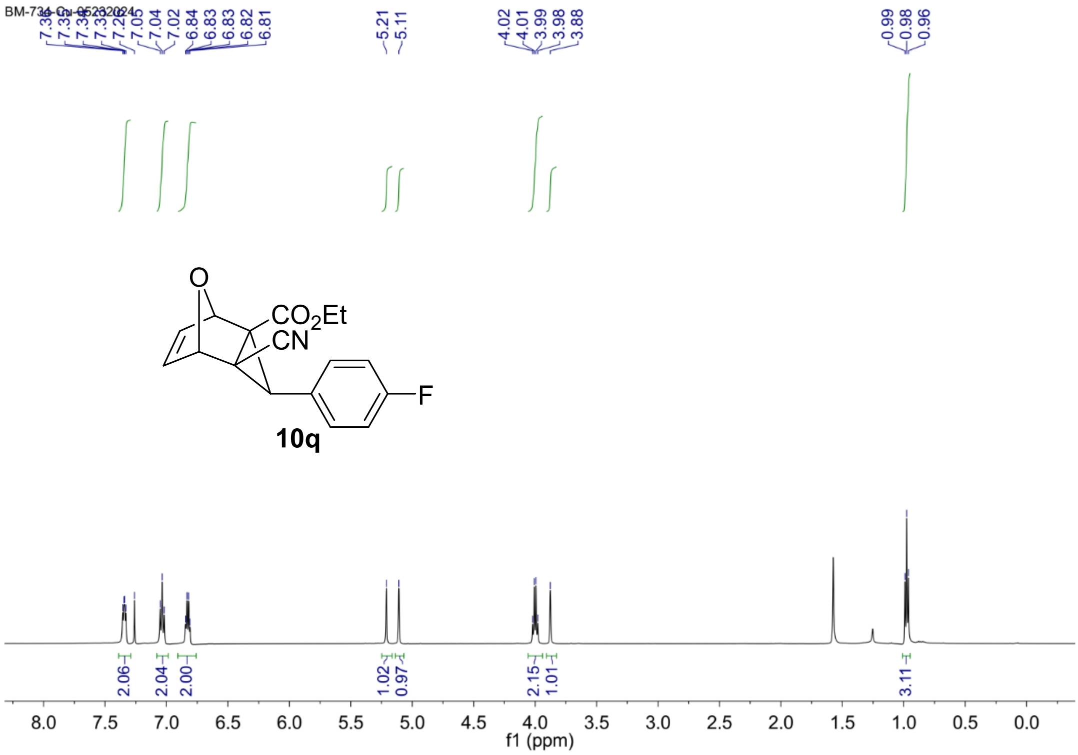
BM-734-Cu-05282024
7.05
7.04
7.02
6.84
6.83
6.82
6.81
5.21
5.11
4.02
4.01
3.99
3.98
3.88
0.99
0.98
0.96
O
CO_2Et
CN
F
10q
2.06
2.04
2.00
1.02
0.97
2.15
1.01
3.11
8.0 7.5 7.0 6.5 6.0 5.5 5.0 4.5 4.0 3.5 3.0 2.5 2.0 1.5 1.0 0.5 0.0
f1 (ppm)

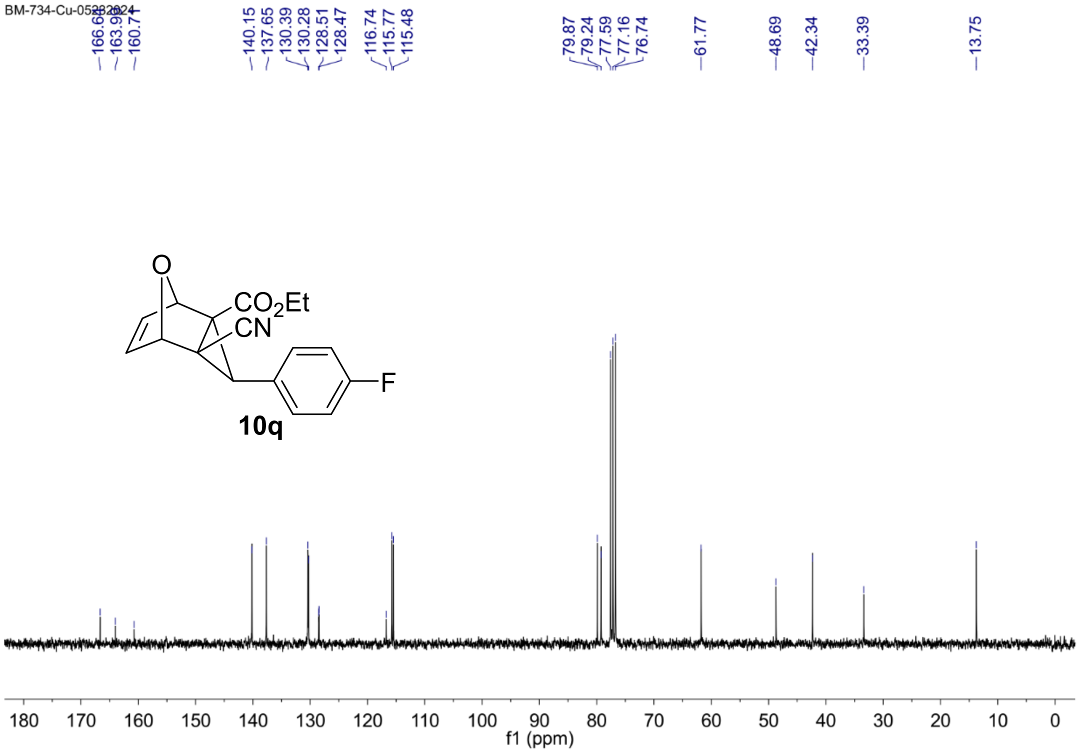
140.15
137.65
130.39
130.28
128.51
128.47
116.74
115.77
115.48
79.87
79.24
77.59
77.16
76.74
61.77
48.69
42.34
33.39
13.75
O
CO_2Et
CN
F
10q
180 170 160 150 140 130 120 110 100 90 80 70 60 50 40 30 20 10 0
f1 (ppm)

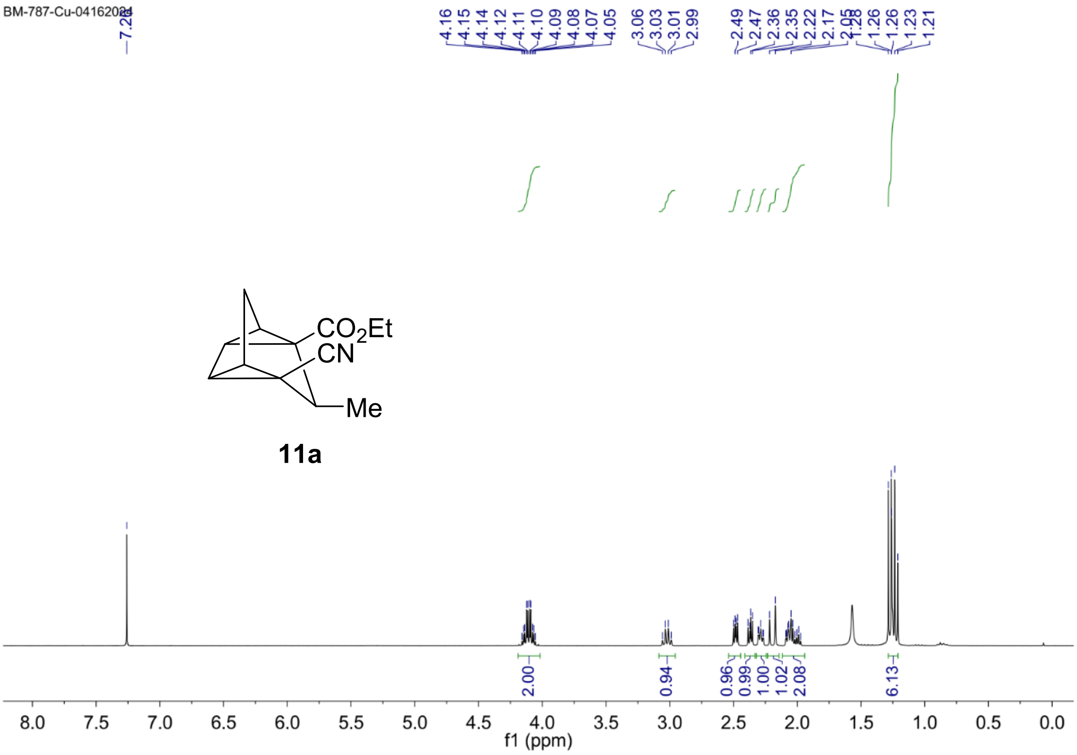
BM-787-Cu-04162024
7.26
4.16
4.15
4.14
4.12
4.11
4.10
4.09
4.08
4.07
4.05
3.06
3.03
3.01
2.99
2.49
2.47
2.36
2.35
2.22
2.17
1.28
1.26
1.26
1.23
1.21
CO2Et
CN
Me
11a
2.00
0.94
0.96
0.99
1.00
1.02
2.08
6.13
8.0 7.5 7.0 6.5 6.0 5.5 5.0 4.5 4.0 3.5 3.0 2.5 2.0 1.5 1.0 0.5 0.0
f1 (ppm)

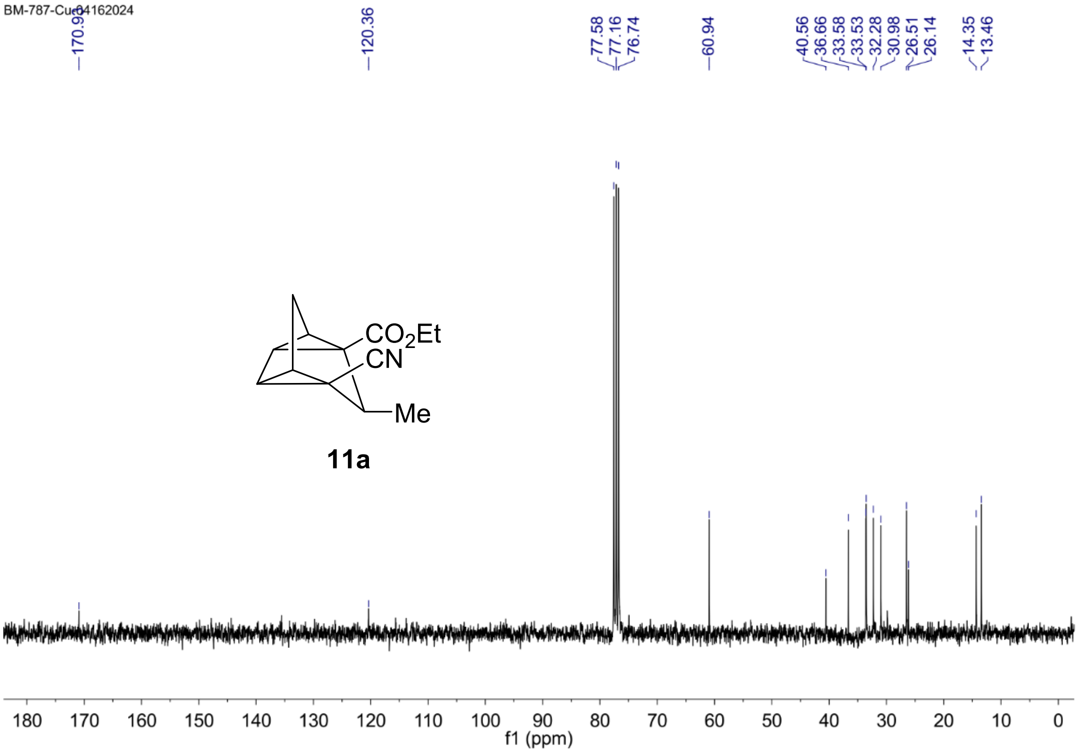
BM-787-Cu-04162024
170.90
120.36
77.58
77.16
76.74
60.94
40.56
36.66
33.58
33.53
32.28
30.98
26.51
26.14
14.35
13.46
CO2Et
CN
Me
11a
180 170 160 150 140 130 120 110 100 90 80 70 60 50 40 30 20 10 0
f1 (ppm)

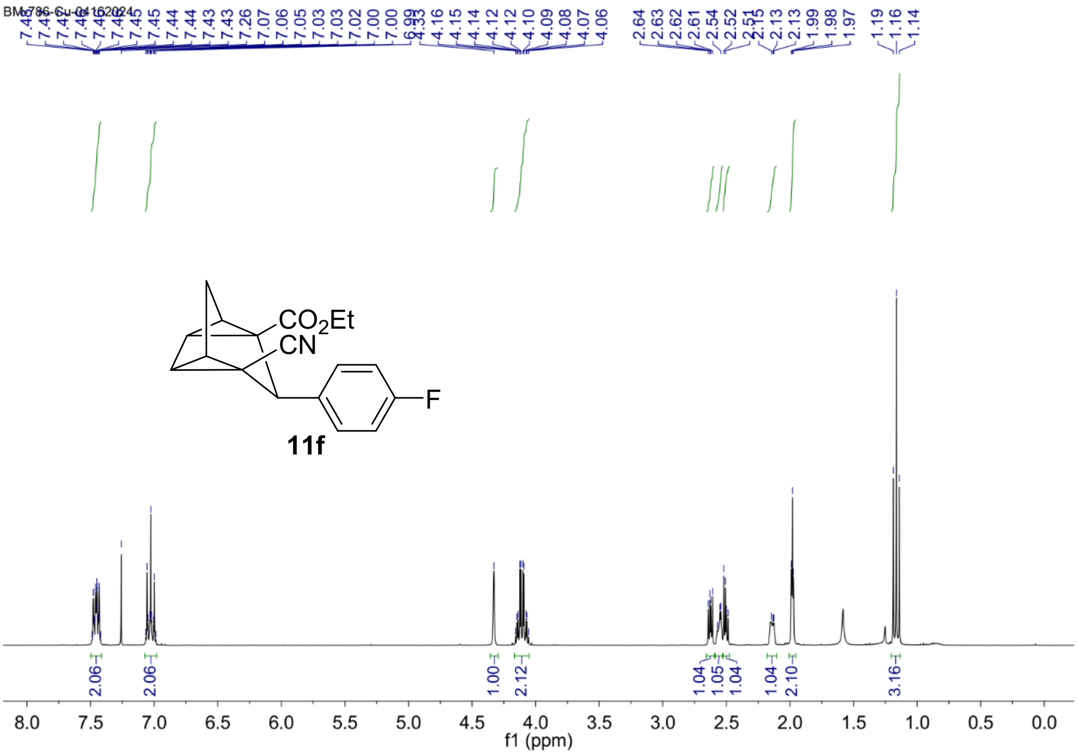
BM-786-Cu-04162024
CO2Et
CN
F
11f
2.06
2.06
1.00
2.12
1.04
1.05
1.04
1.04
2.10
3.16
f1 (ppm)

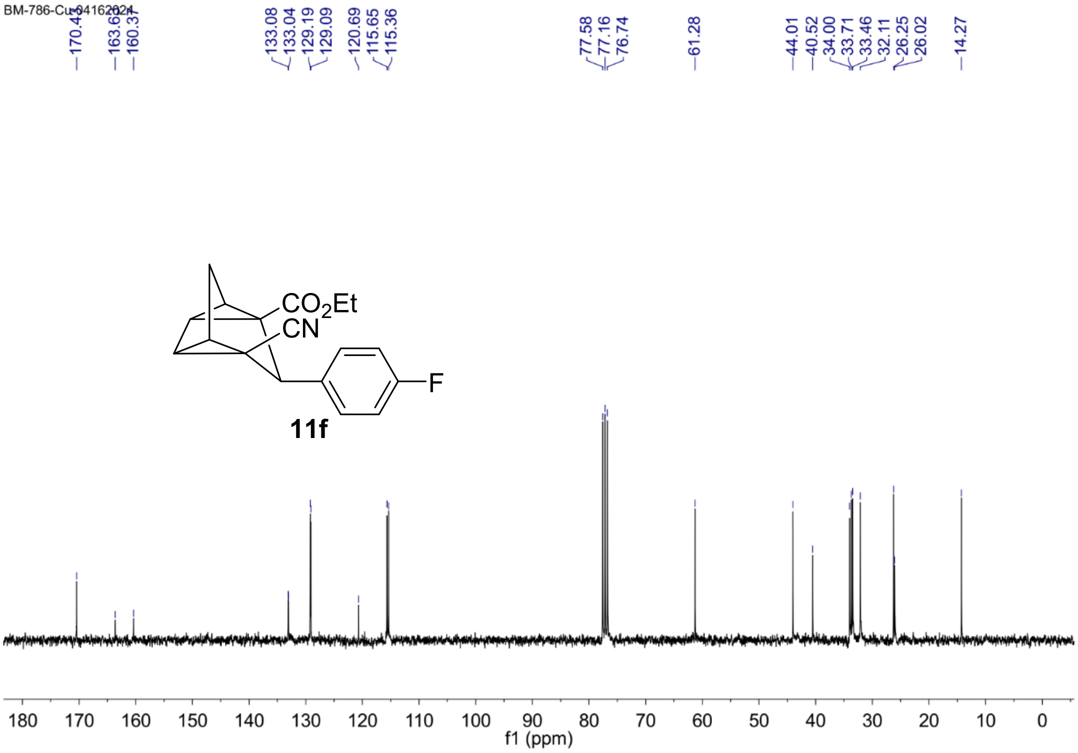
BM-786-Cu-04162024
170.41
163.63
160.37
133.08
133.04
129.19
129.09
120.69
115.65
115.36
77.58
77.16
76.74
61.28
44.01
40.52
34.00
33.71
33.46
32.11
26.25
26.02
14.27
CO2Et
CN
F
11f
f1 (ppm)

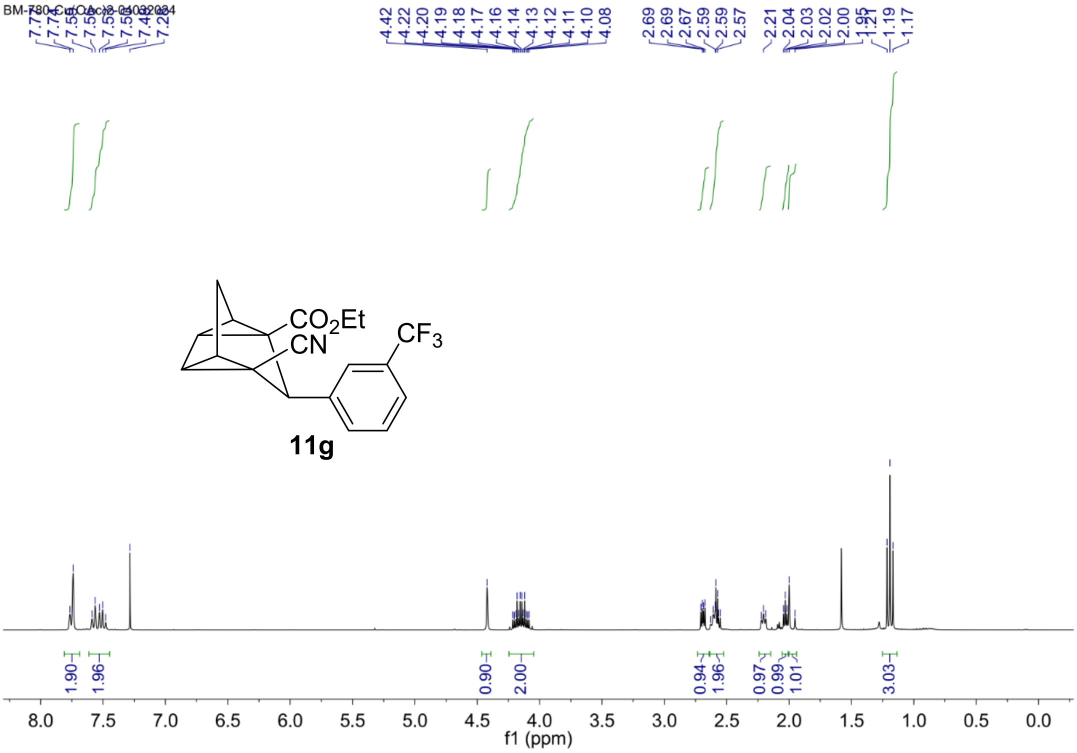
4.42
4.22
4.20
4.19
4.18
4.17
4.16
4.14
4.13
4.12
4.11
4.10
4.08
2.69
2.69
2.67
2.59
2.59
2.57
2.21
2.04
2.03
2.02
2.00
1.21
1.19
1.17
CO2Et
CN
CF3
11g
1.90
1.96
0.90
2.00
0.94
1.96
0.97
0.99
1.01
3.03
f1 (ppm)

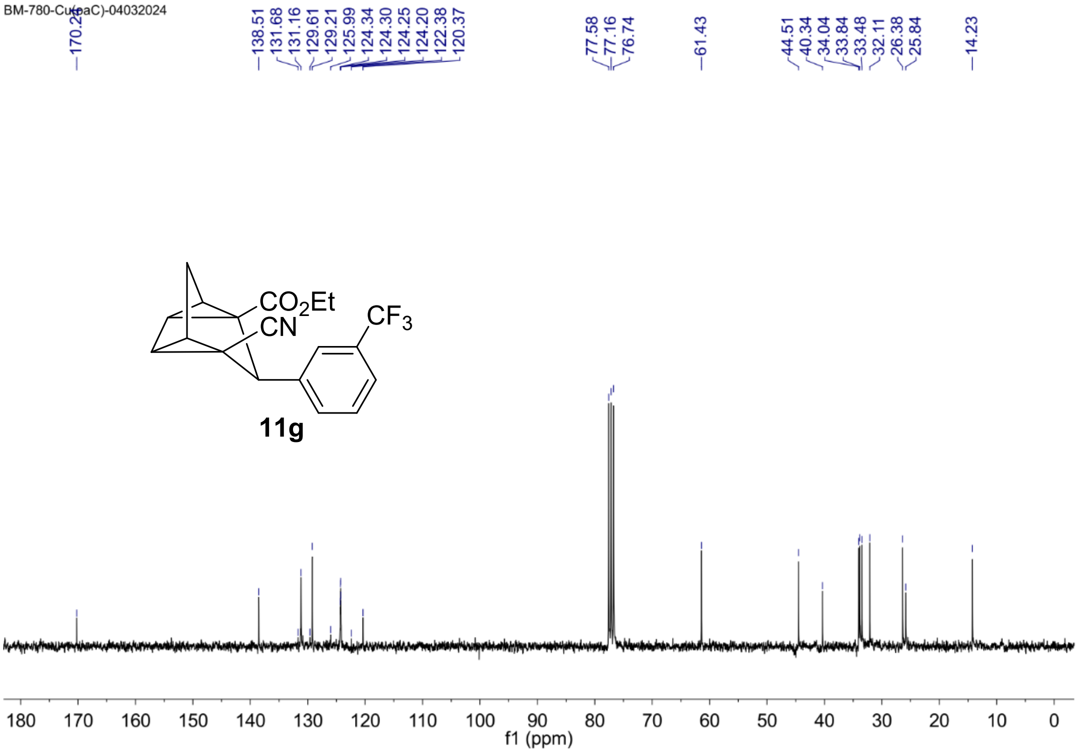
138.51
131.68
131.16
129.61
129.21
125.99
124.34
124.30
124.25
124.20
122.38
120.37
77.58
77.16
76.74
61.43
44.51
40.34
34.04
33.84
33.48
32.11
26.38
25.84
14.23
CO2Et
CN
CF3
11g
f1 (ppm)

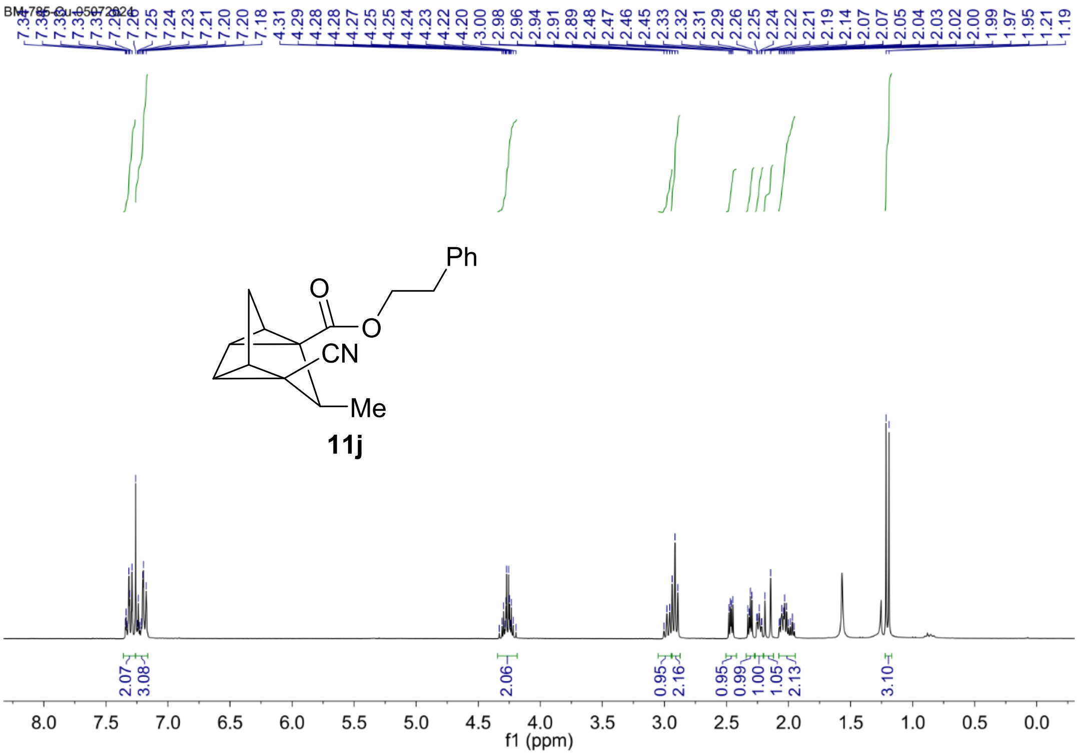
BM-785-Cu-05072024
7.25 7.24 7.23 7.21 7.20 7.20 7.18 4.31 4.29 4.28 4.28 4.27 4.25 4.25 4.24 4.23 4.22 4.20 3.00 2.98 2.96 2.94 2.91 2.89 2.48 2.47 2.46 2.45 2.33 2.32 2.31 2.29 2.26 2.25 2.24 2.22 2.21 2.19 2.14 2.07 2.07 2.05 2.04 2.03 2.02 2.00 1.99 1.97 1.95 1.21 1.19
Ph
O
O
CN
Me
11j
2.07 3.08 2.06 0.95 2.16 0.95 0.99 1.00 1.05 2.13 3.10
8.0 7.5 7.0 6.5 6.0 5.5 5.0 4.5 4.0 3.5 3.0 2.5 2.0 1.5 1.0 0.5 0.0
f1 (ppm)

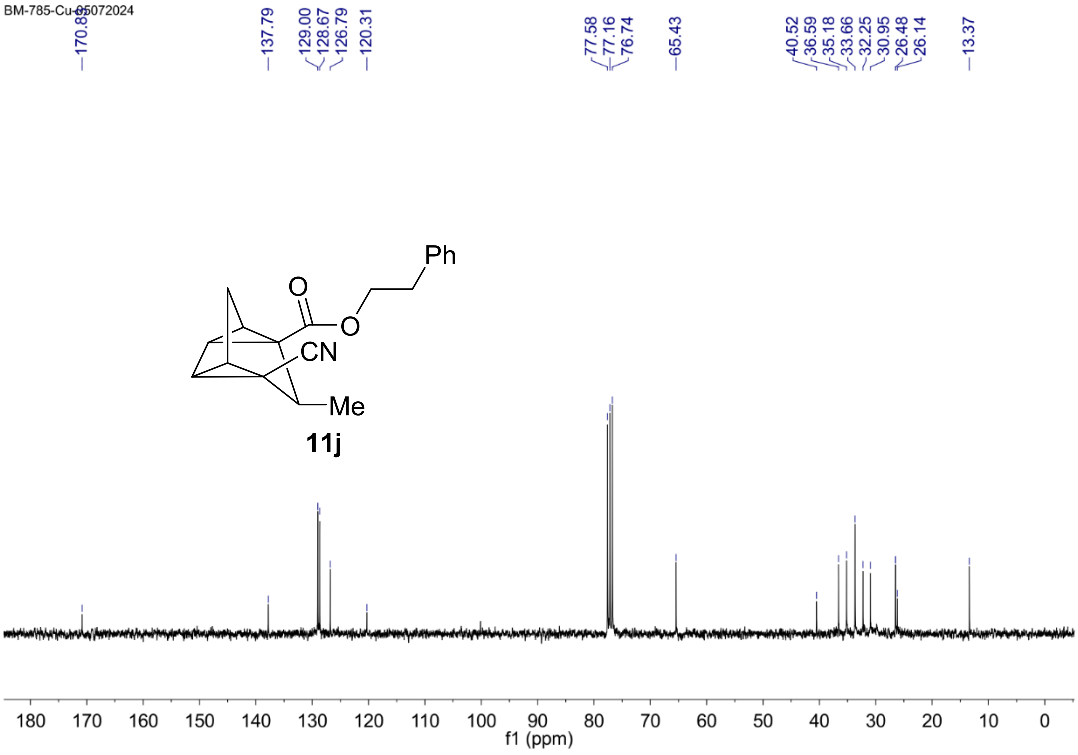
BM-785-Cu-05072024
170.89 137.79 129.00 128.67 126.79 120.31 77.58 77.16 76.74 65.43 40.52 36.59 35.18 33.66 32.25 30.95 26.48 26.14 13.37
Ph
O
O
CN
Me
11j
180 170 160 150 140 130 120 110 100 90 80 70 60 50 40 30 20 10 0
f1 (ppm)

Crystallographic Data for Compound 3i

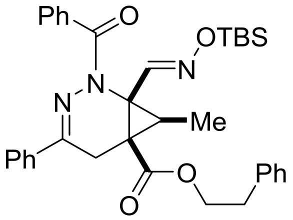

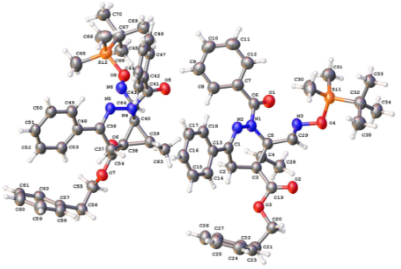

3i (CCDC: 2346069)

Figure S3. ORTEP drawing of **3i** showing thermal ellipsoids at the 50% probability level.

Crystallographic Data for Compound 8k

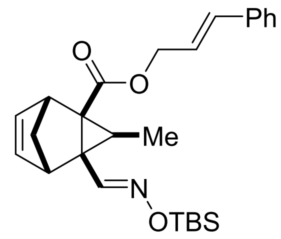

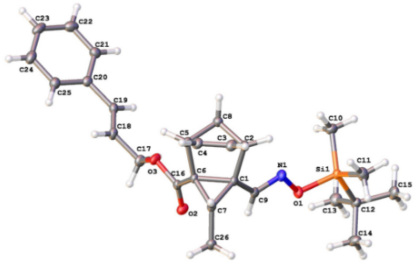

8k (CCDC: 2346068)

Figure S4. ORTEP drawing of **8k** showing thermal ellipsoids at the 50% probability level.

Single crystals of $C_{26}H_{35}NO_3Si$(**8k**) were prepared by slow evaporation of a dicholormethane/hexane solution. A suitable colorless plate-like crystal, with dimensions of 0.121 mm × 0.071 mm × 0.061 mm, was mounted in paratone oil onto a nylon loop. Single crystals of $C_{35}H_{41}N_3O_4Si$(**3i**) were prepared by slow evaporation of a dicholormethane/hexane solution. A suitable colorless plate-like crystal, with dimensions of 0.139 mm × 0.093 mm × 0.062 mm, was mounted in paratone oil onto a nylon loop. All data were collected at 100.0(1) K and 298(1) K for compounds 1 and 2 respectively, using a XtaLAB Synergy/ Dualflex, HyPix fitted with CuKα radiation (λ = 1.54184 Å). Data collection and unit cell refinement were performed using *CrysAlisPro* software.[3] The total number of data were measured in the $5.0° < 2\theta < 153.4°$ and $5.4° < 2\theta < 153.7°$ for compounds (1) and (2) respectively, using ω scans. Data processing and absorption correction, giving minimum and maximum transmission factors (0.796, 1.000 for compound (1), 0.007, 0.117 for compound (2)) were accomplished with *CrysAlisPro*[3] and *SCALE3 ABSPACK*,[4] respectively. The structure,

using Olex2,[5] was solved with the ShelXT[6] structure solution program using direct methods and refined (on F^2) with the ShelXL[7] refinement package using full-matrix, least-squares techniques. All non-hydrogen atoms were refined with anisotropic displacement parameters. All hydrogen atom positions were determined by geometry and refined by a riding model.

Table S1: Crystallographic data and structure refinement for Compounds **8k** and **3i**

Identification code	Hpd842(**8k**)	Hpd873(**3i**)
Empirical formula	$C_{26}H_{35}NO_3Si$	$C_{35}H_{41}N_3O_4Si$
Formula weight	437.64	595.80
Crystal system	Triclinic	Triclinic
Space group	*P-1*	*P-1*
a (Å)	6.0332(1)	14.0714(2)
b (Å)	11.3990(3)	14.8588(2)
c (Å)	17.7451(3)	16.3784(2)
α (°)	84.659 (2)	90.572(1)
β (°)	88.405(2)	92.785(1)
γ (°)	83.737(2)	107.376(1)
Volume (Å^3)	1207.63(4)	3263.25(8)
Z	2	4
ρ (calc.)	1.204	1.213
λ	1.54184	1.54184
Temp. (K)	100.0(1)	100.0(1)
F(000)	472	1272
μ (mm^{-1})	1.062	0.966
T_{min}, T_{max}	0.796, 1.000	0.007, 0.117
$2\theta_{range}$ (°)	5.0 to 153.4	5.4, 153.7
Reflections collected	22726	60696
Independent reflections	4794 [R(int) = 0.0373]	23211 [R(int) = 0.1006]
Completeness	99.9%	99.8%
Data / restraints / parameters	4794 / 0 / 286	23211 / 0 / 788
Observed data [I > 2σ(I)]	4388	20241
$wR(F^2$ all data)	0.0924	0.1432
$R(F$ obsd data)	0.0341	0.0669

Goodness-of-fit on F^2	0.992	1.002
largest diff. peak and hole (e Å^{-3})	0.29 / -0.30	0.90 / -0.57

$$wR_2 = \{ \Sigma [w(F_o^2 - F_c^2)^2] / \Sigma [w(F_o^2)^2] \}^{1/2}$$

$$R_1 = \Sigma ||F_o| - |F_c|| / \Sigma |F_o|$$

References:

1. (a) M. Bao, L. De Angeles, M. S. Rada, M. Baird, H. Arman, D. Wherritt and M. P. Doyle, *Chem. Catal.*, 2023, **3**, 100770; (b) H. Zheng, K. Wang, I. Faghihi, W. P. Griffith, H. Arman and M. P. Doyle, *ACS Catal.*, 2021, **11**, 9869-9874.
2. Y. Deng, C. Pei, H. Arman, K. Dong, X. Xu and M. P. Doyle, *Org. Lett.*, 2016, **18**, 5884-5887.
3. CrysAlisPro 1.171.40.63a (Rigaku Oxford Diffraction, 2019)
4. SCALE3 ABSPACK -An Oxford Diffraction program(1.0.4,gui:1.0.3) (C) 2005 Oxford Diffraction Ltd.
5. O. V. Dolomanov, L. J. Bourhis, R. J. Gildea, J. A. K. Howard and H. Puschmann, *J. Appl. Cryst.*, 2009, **42**, 339-341.
6. G. M. Sheldrick, *Acta Cryst.*, 2015, **A71**, 3.
7. G. M. Sheldrick, *Acta Cryst.*, 2008, **A64**, 112.
